# Supplementary figures and images for: Whole-body replacement of larval myofibers generates permanent adult myofibers in zebrafish (part 1 of 4)
Source: EMBO J. 2024 Jun 5;43(15):2. doi: 10.1038/s44318-024-00136-y (PMC11294464; doi:10.1038/s44318-024-00136-y)

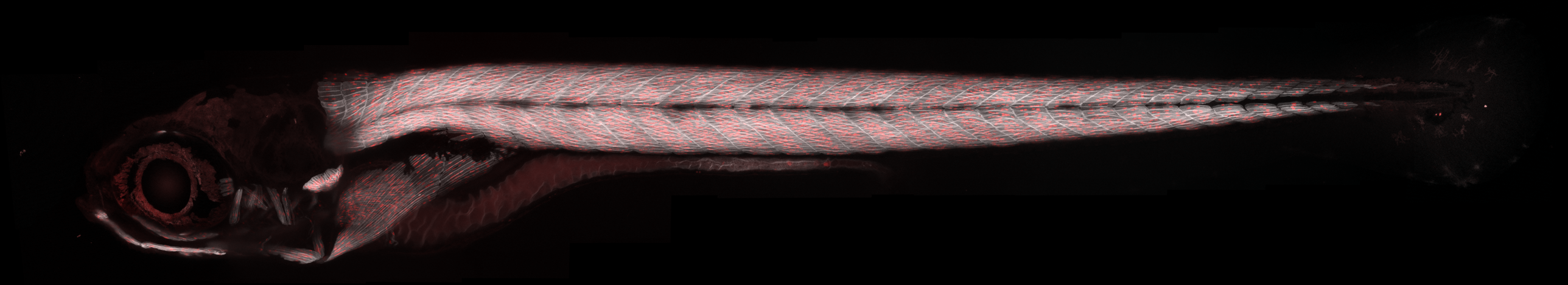

Supplement: Supplementary file 10 — Source data Fig. 1 [file 44318_2024_136_MOESM10_ESM.zip › Figure 1A/palmuscle-Dual-Stitched.tiff]

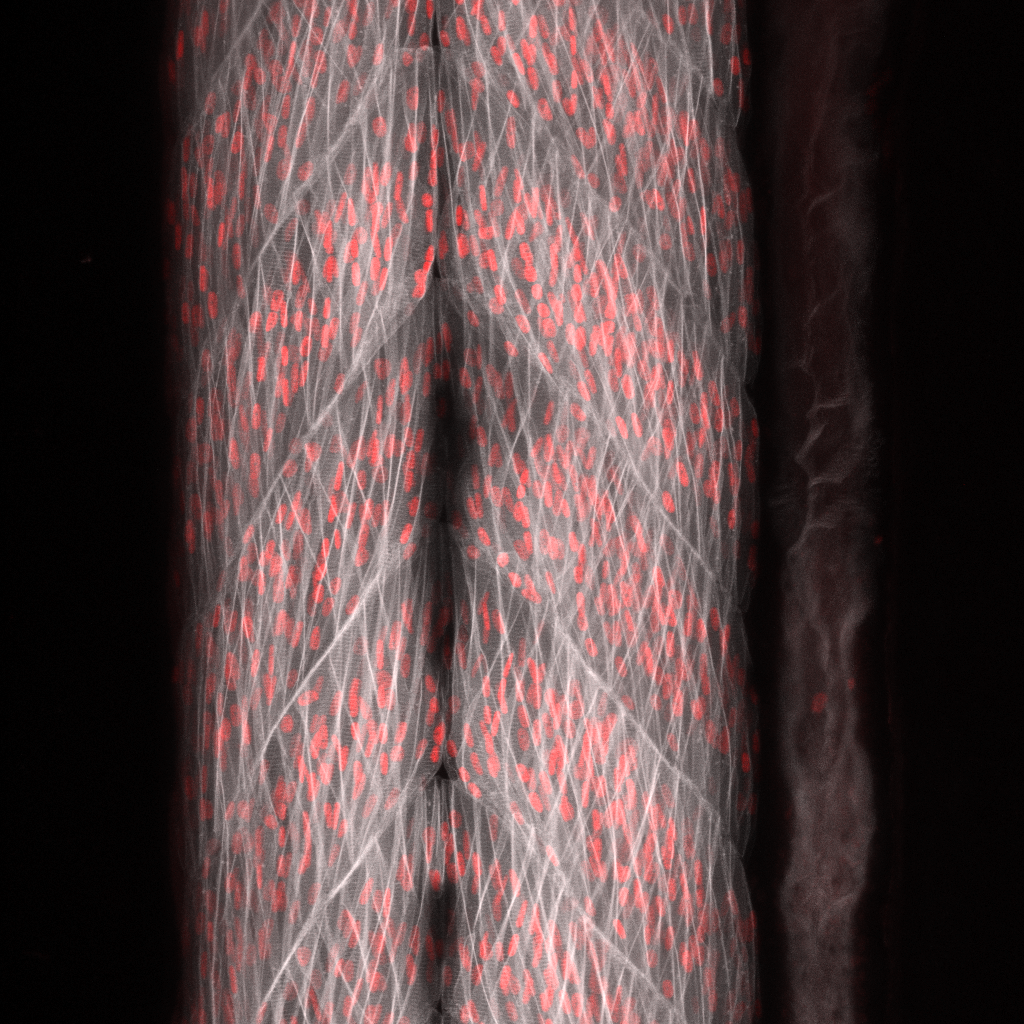

Supplement: Supplementary file 10 — Source data Fig. 1 [file 44318_2024_136_MOESM10_ESM.zip › Figure 1B/palmuscle-Dual-Trunk.tif]

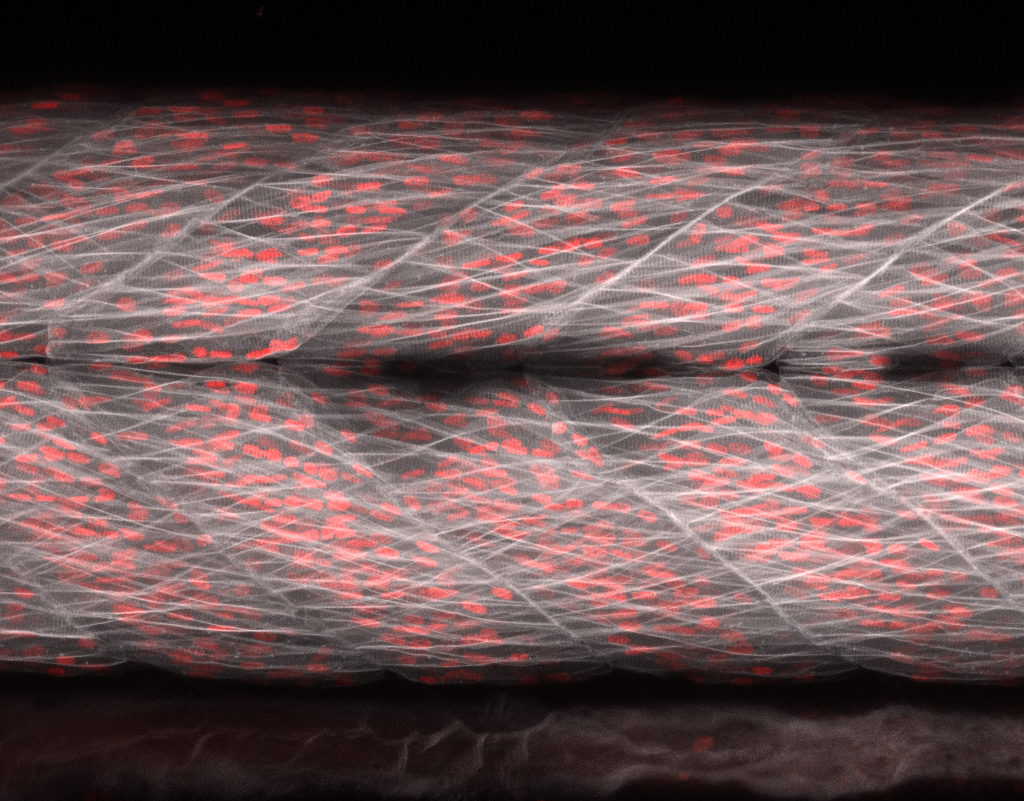

Supplement: Supplementary file 10 — Source data Fig. 1 [file 44318_2024_136_MOESM10_ESM.zip › Figure 1B/palmuscle-Dual-Trunk-Crop.tif]

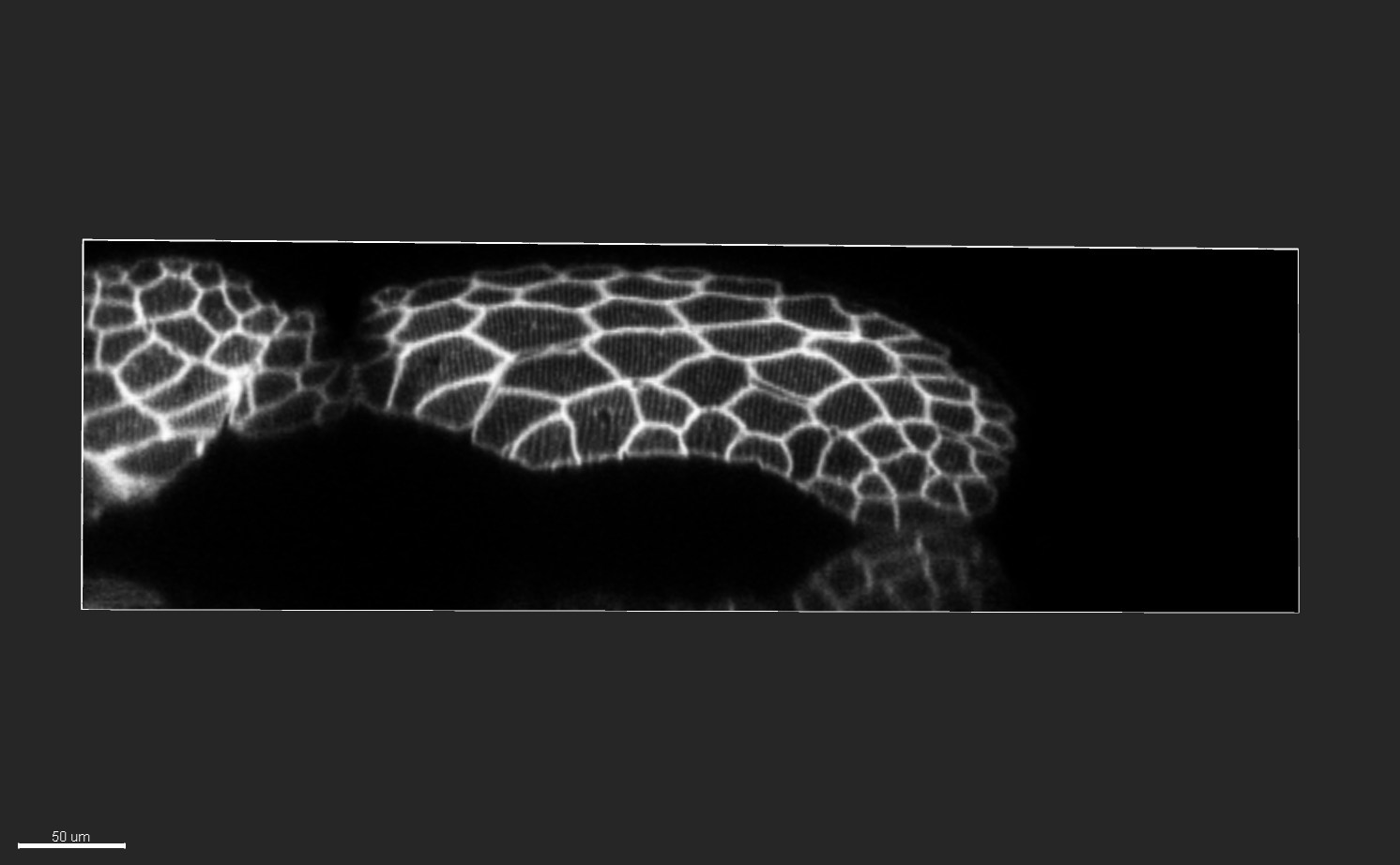

Supplement: Supplementary file 10 — Source data Fig. 1 [file 44318_2024_136_MOESM10_ESM.zip › Figure 1C/Dorsal.tif]

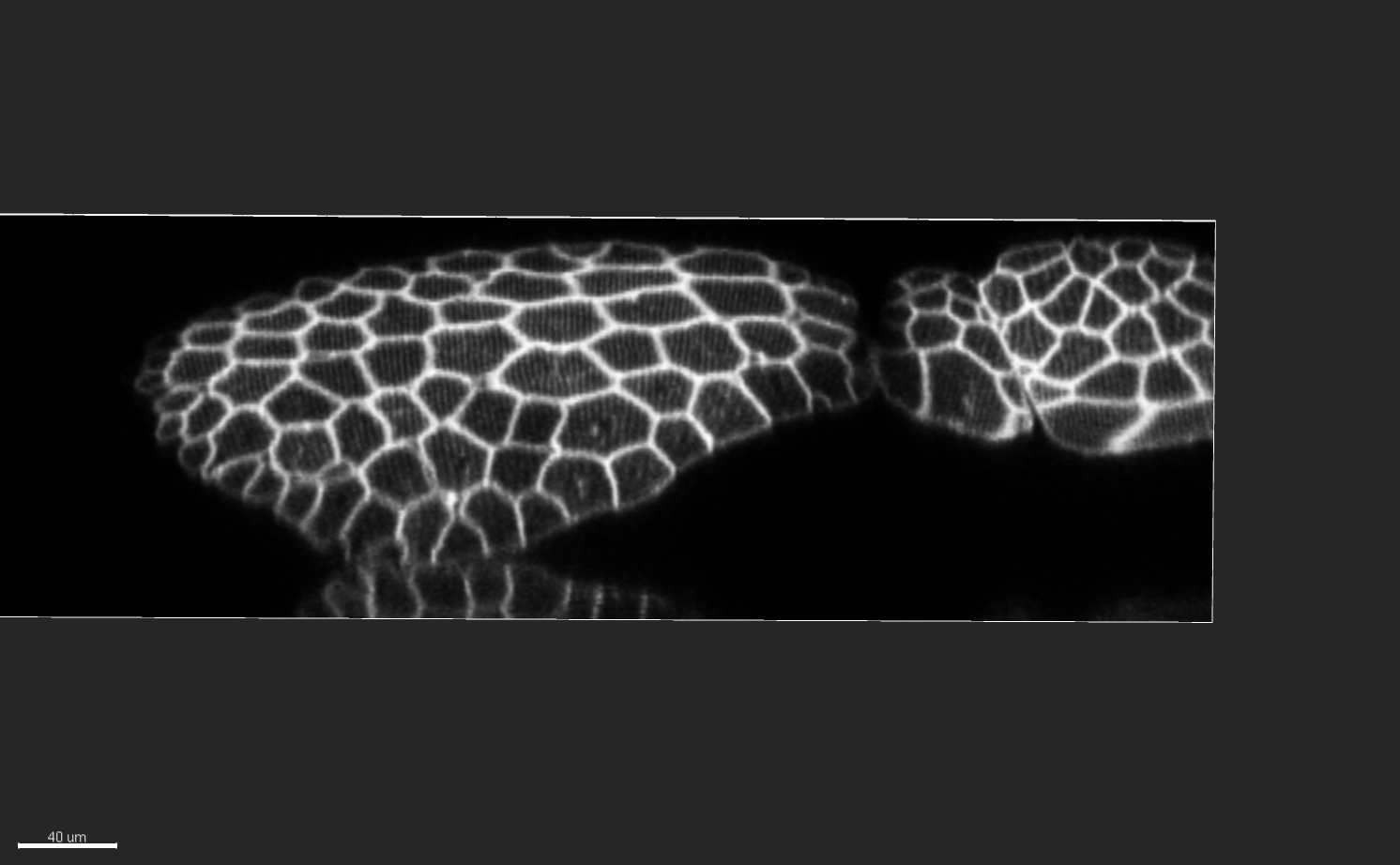

Supplement: Supplementary file 10 — Source data Fig. 1 [file 44318_2024_136_MOESM10_ESM.zip › Figure 1C/Ventral.tif]

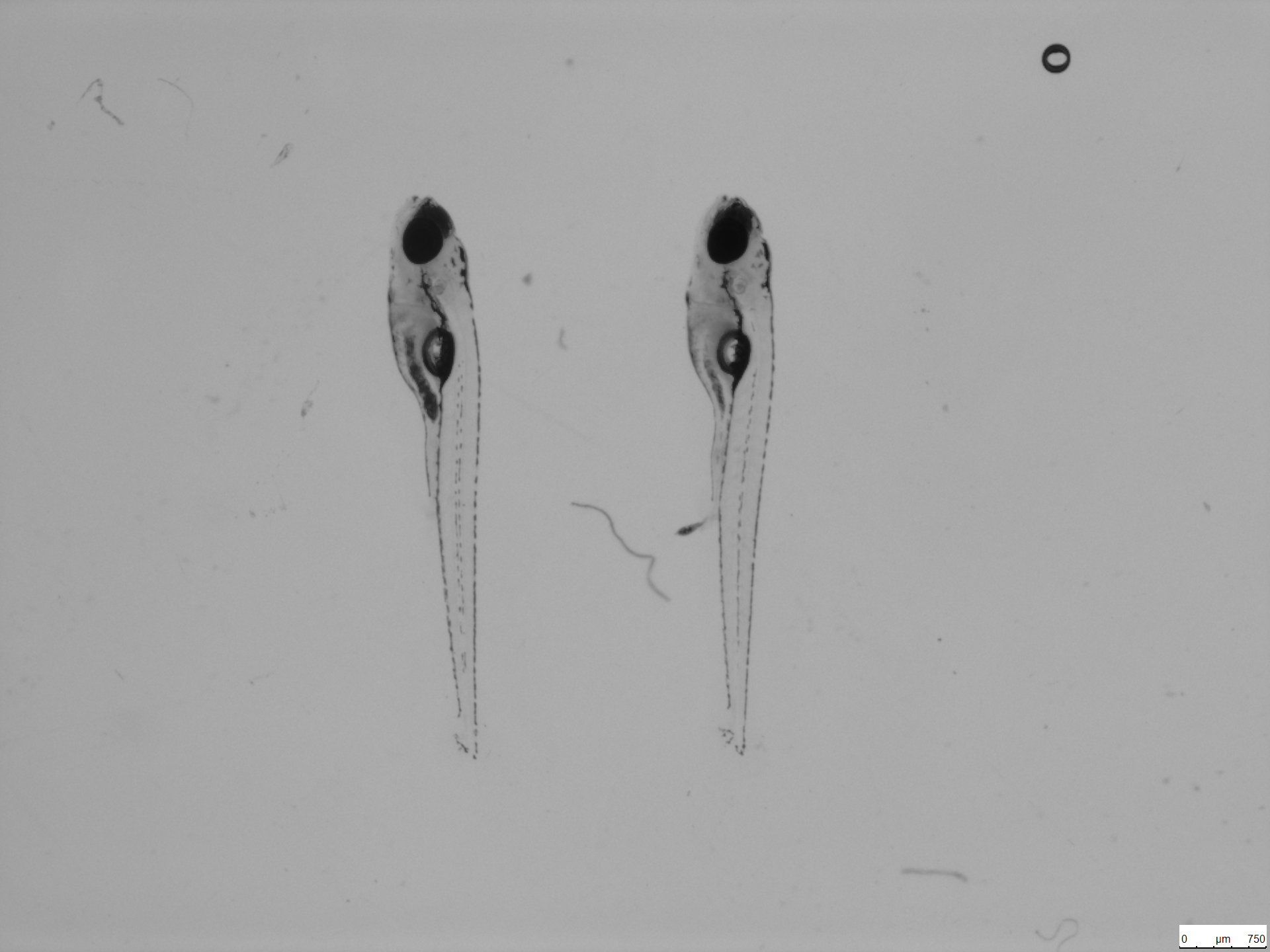

Supplement: Supplementary file 10 — Source data Fig. 1 [file 44318_2024_136_MOESM10_ESM.zip › Figure 1D-E/10 dpf-Standard length and Trunk surface area-Fish 1 & 2.tif]

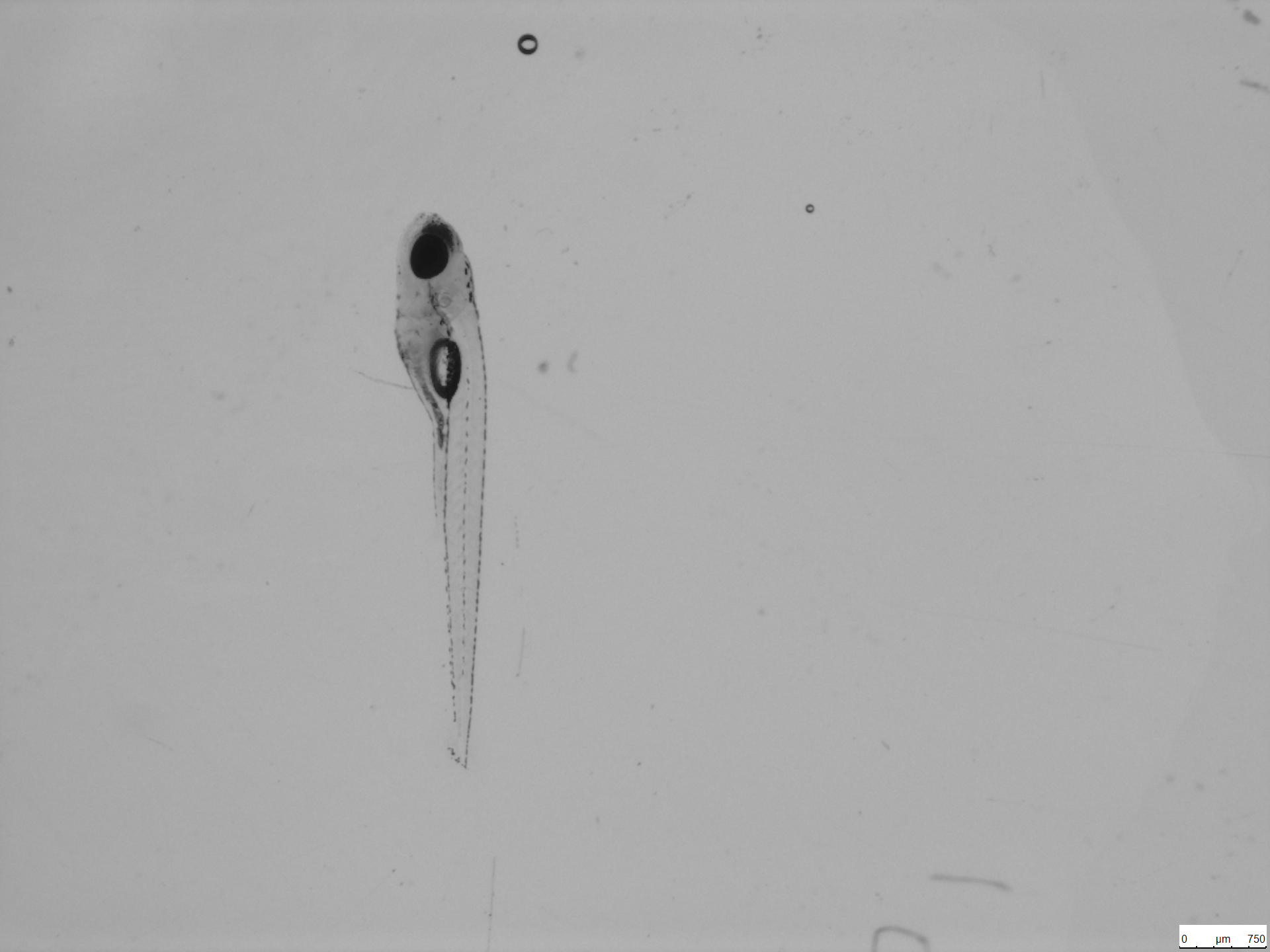

Supplement: Supplementary file 10 — Source data Fig. 1 [file 44318_2024_136_MOESM10_ESM.zip › Figure 1D-E/10 dpf-Standard length and Trunk surface area-Fish 3.tif]

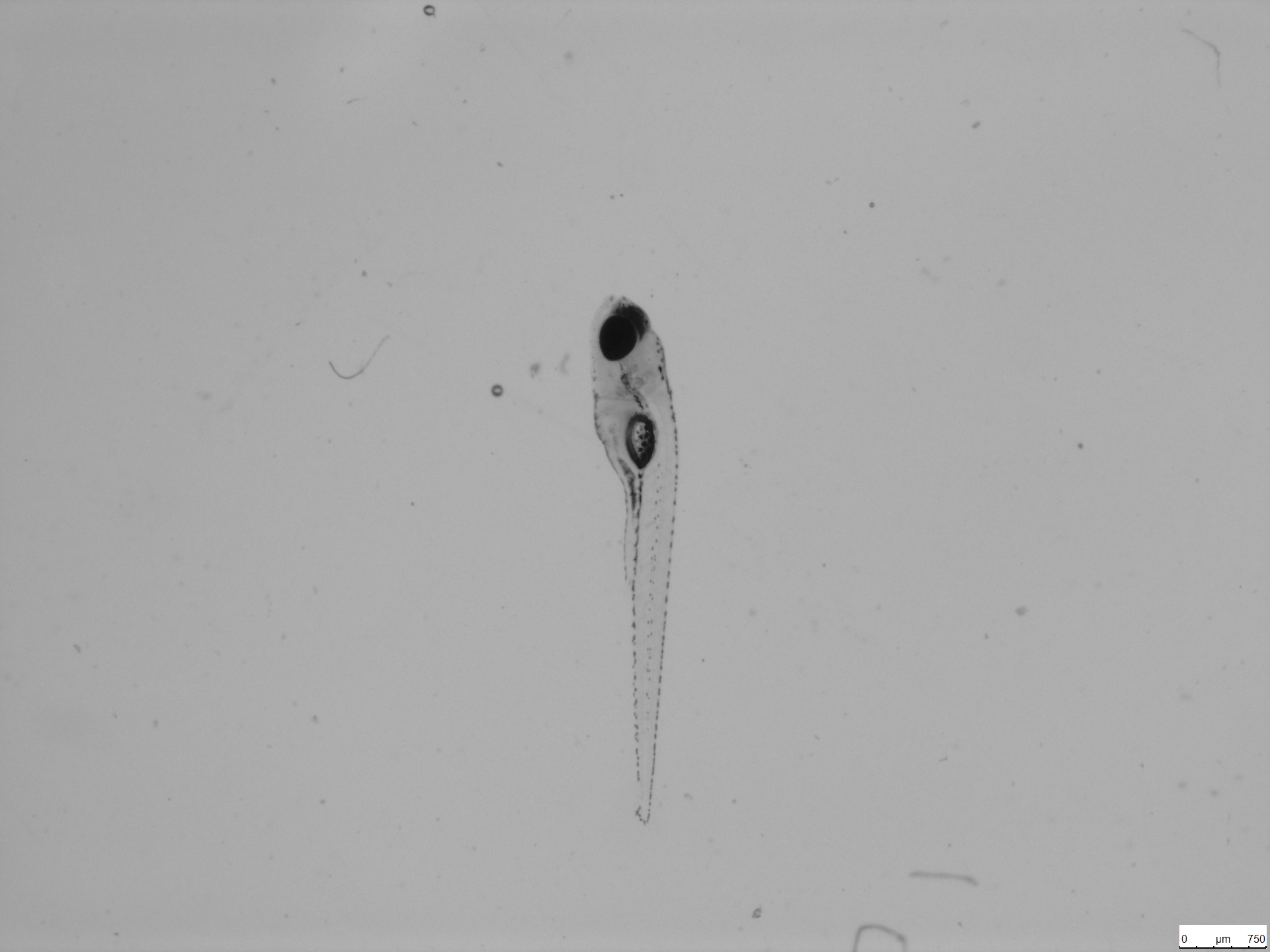

Supplement: Supplementary file 10 — Source data Fig. 1 [file 44318_2024_136_MOESM10_ESM.zip › Figure 1D-E/10 dpf-Standard length and Trunk surface area-Fish 4.tif]

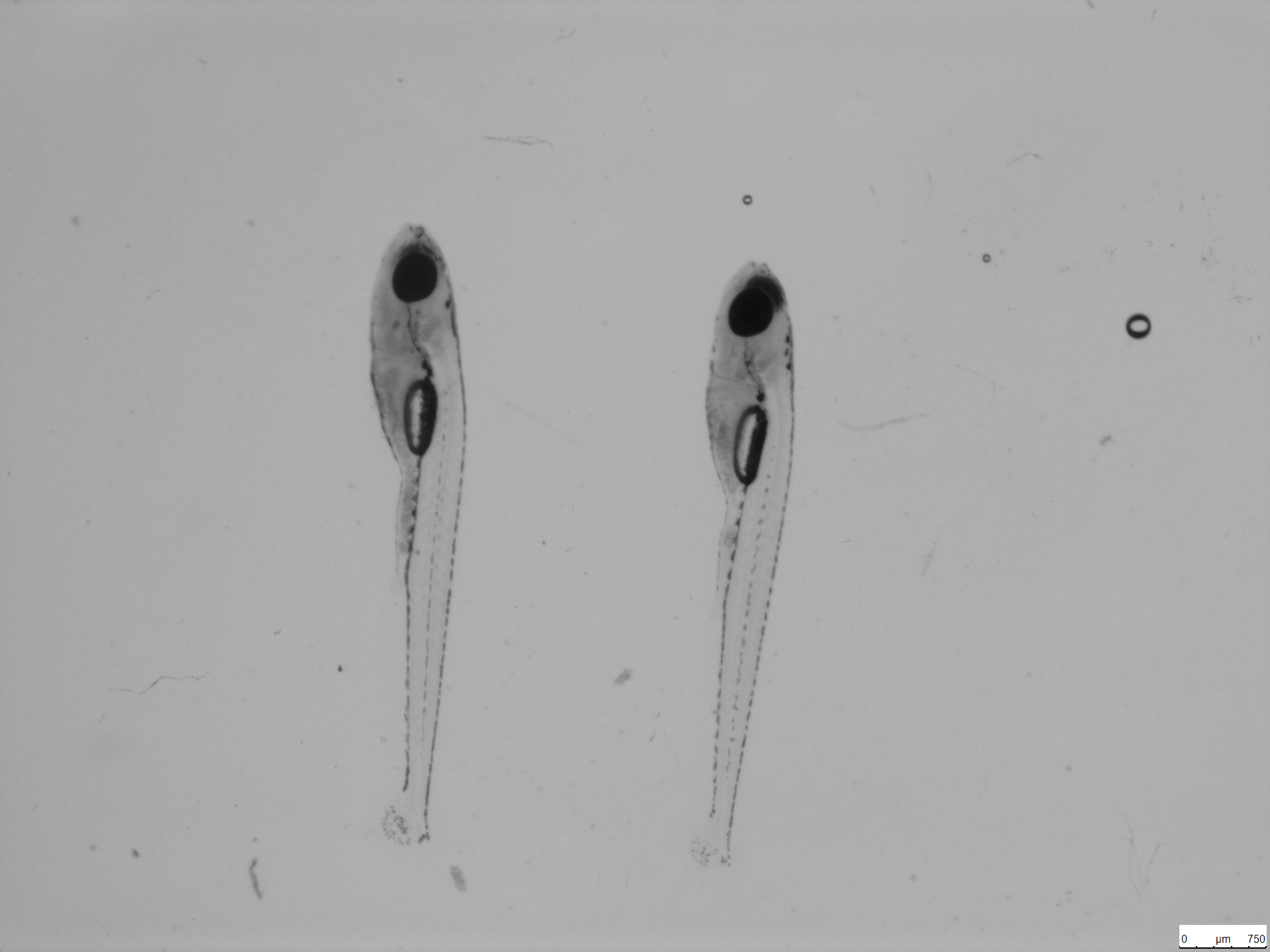

Supplement: Supplementary file 10 — Source data Fig. 1 [file 44318_2024_136_MOESM10_ESM.zip › Figure 1D-E/14 dpf-Standard length and Trunk surface area-Fish 1 & 2.tif]

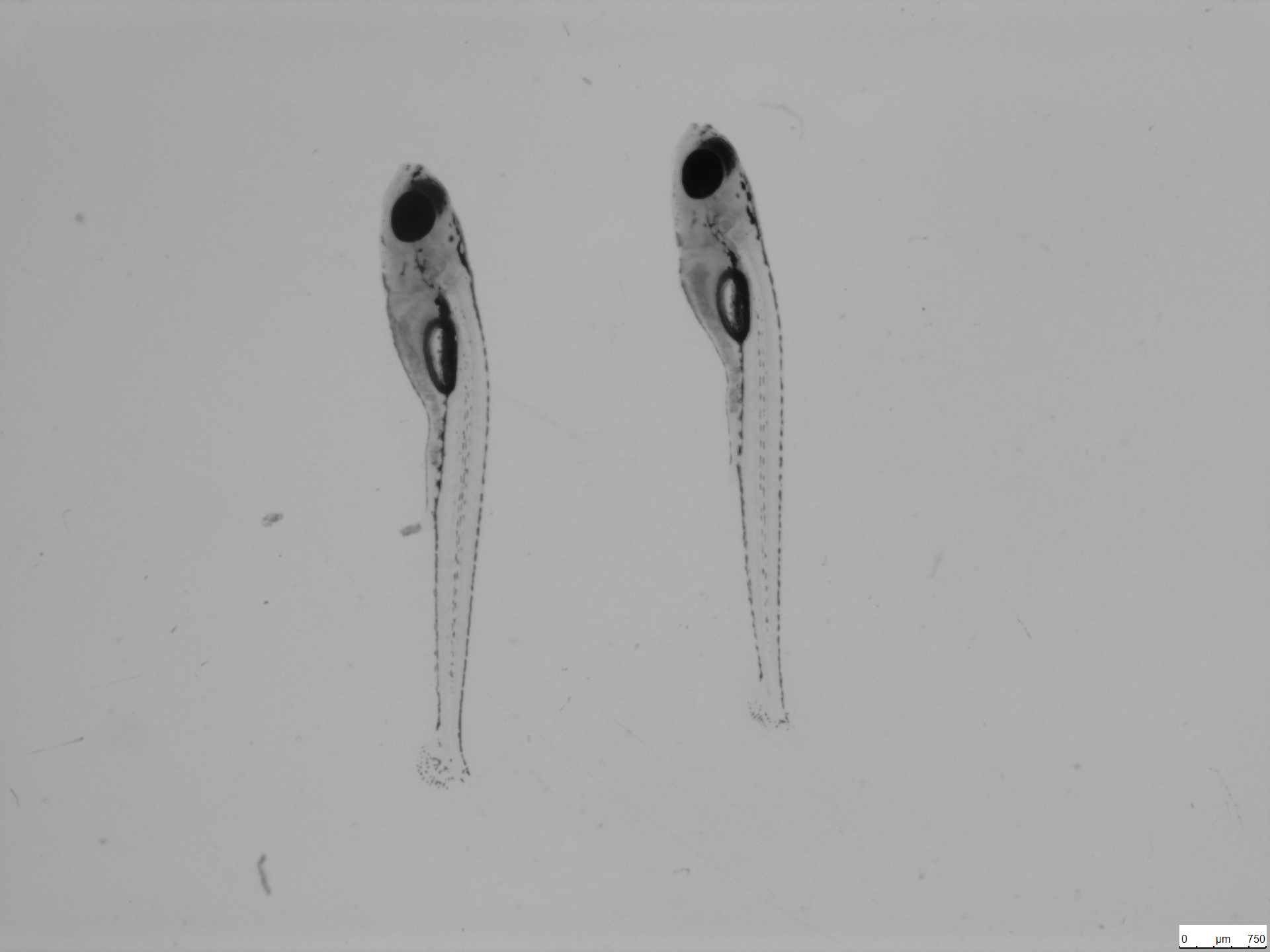

Supplement: Supplementary file 10 — Source data Fig. 1 [file 44318_2024_136_MOESM10_ESM.zip › Figure 1D-E/14 dpf-Standard length and Trunk surface area-Fish 3 & 4.tif]

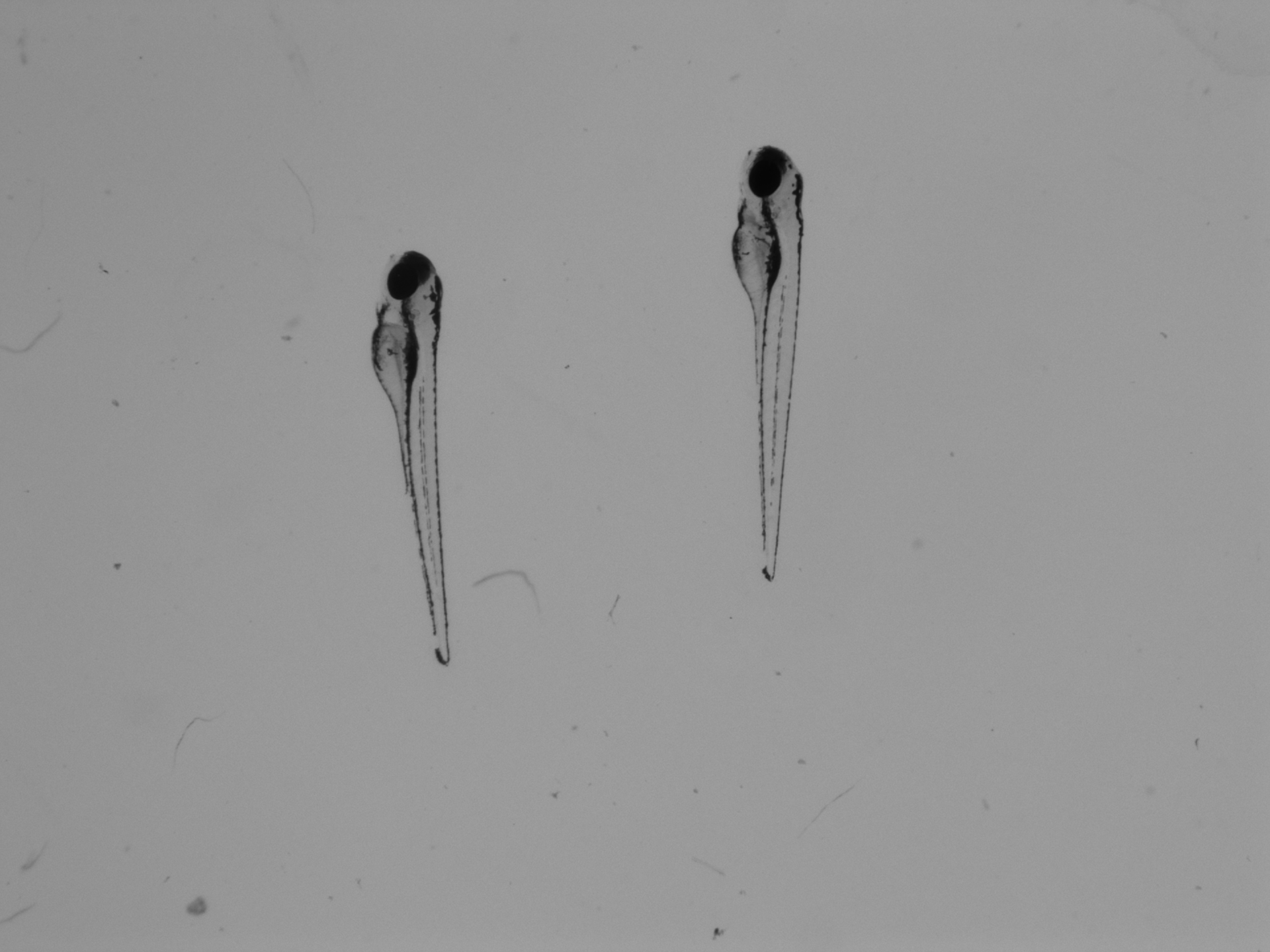

Supplement: Supplementary file 10 — Source data Fig. 1 [file 44318_2024_136_MOESM10_ESM.zip › Figure 1D-E/6 dpf-Standard length and Trunk surface area-Fish 1 & 2.tif]

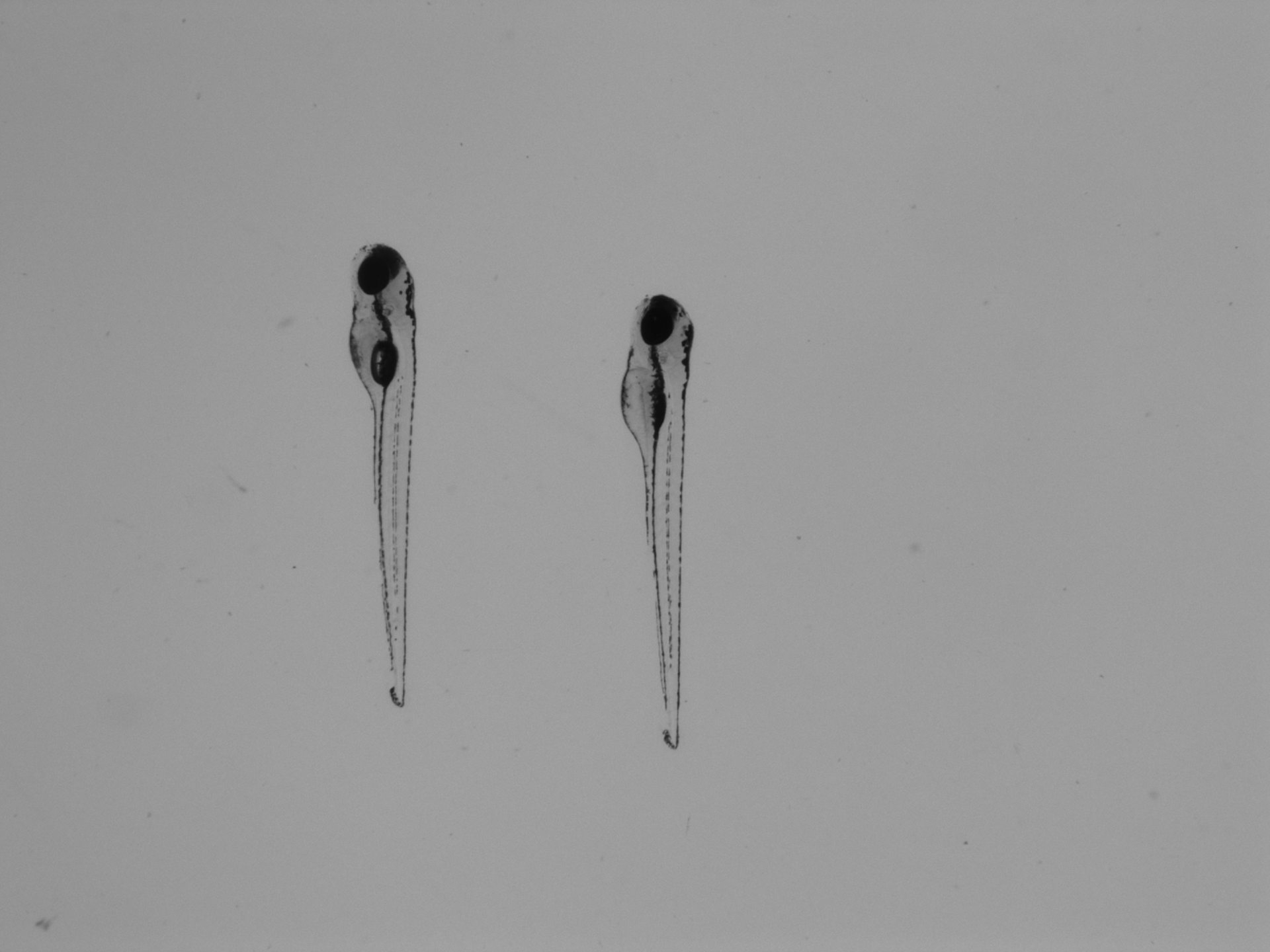

Supplement: Supplementary file 10 — Source data Fig. 1 [file 44318_2024_136_MOESM10_ESM.zip › Figure 1D-E/6 dpf-Standard length and Trunk surface area-Fish 3 & 4.tif]

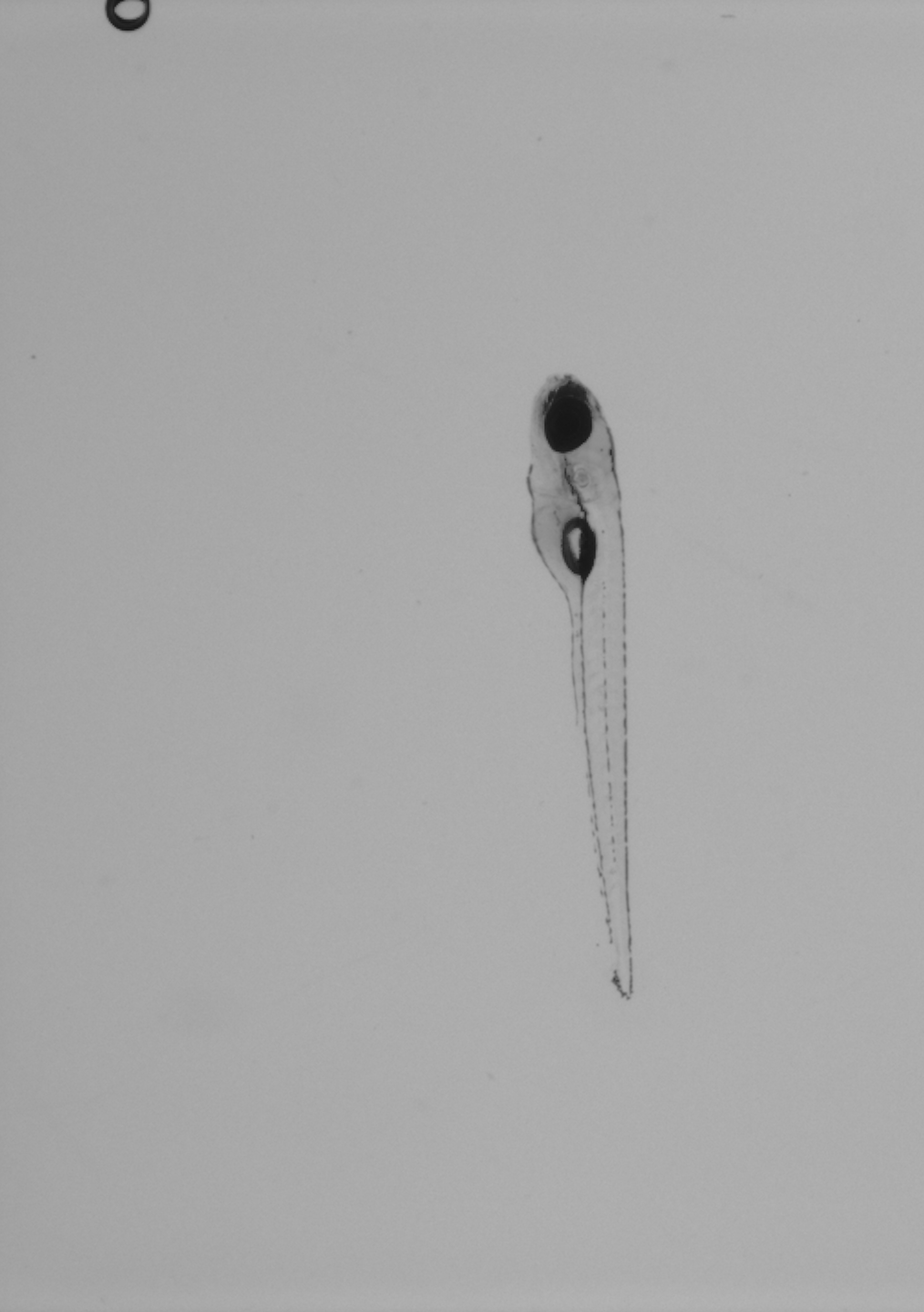

Supplement: Supplementary file 10 — Source data Fig. 1 [file 44318_2024_136_MOESM10_ESM.zip › Figure 1D-E/8 dpf-Standard length and Trunk surface area-Fish 1.tif]

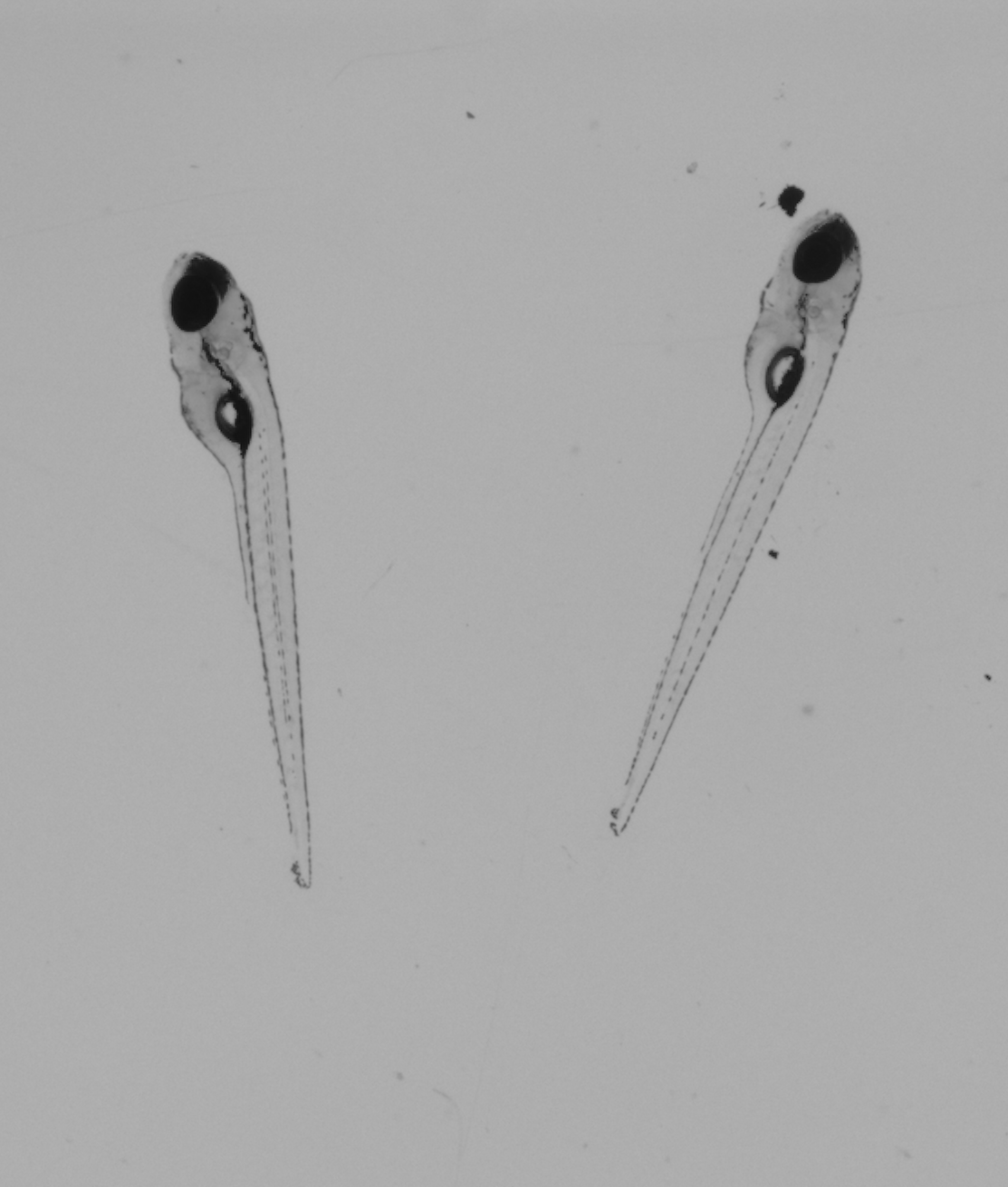

Supplement: Supplementary file 10 — Source data Fig. 1 [file 44318_2024_136_MOESM10_ESM.zip › Figure 1D-E/8 dpf-Standard length and Trunk surface area-Fish 2 & 3.tif]

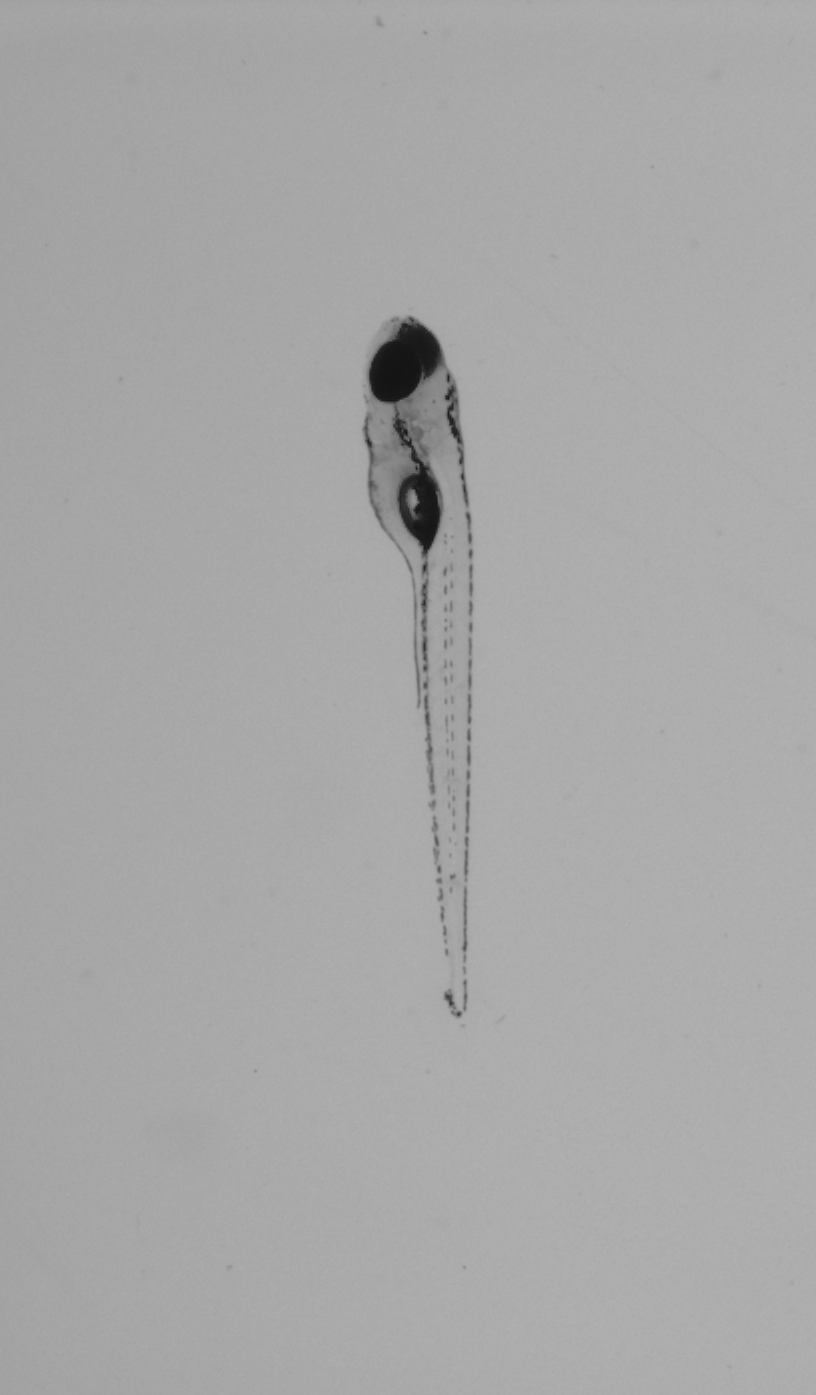

Supplement: Supplementary file 10 — Source data Fig. 1 [file 44318_2024_136_MOESM10_ESM.zip › Figure 1D-E/8 dpf-Standard length and Trunk surface area-Fish 4.tif]

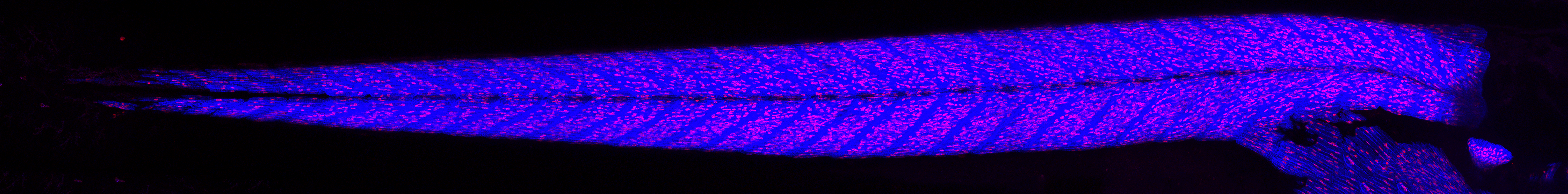

Supplement: Supplementary file 10 — Source data Fig. 1 [file 44318_2024_136_MOESM10_ESM.zip › Figure 1F/10 dpf-Fish 1.tiff]

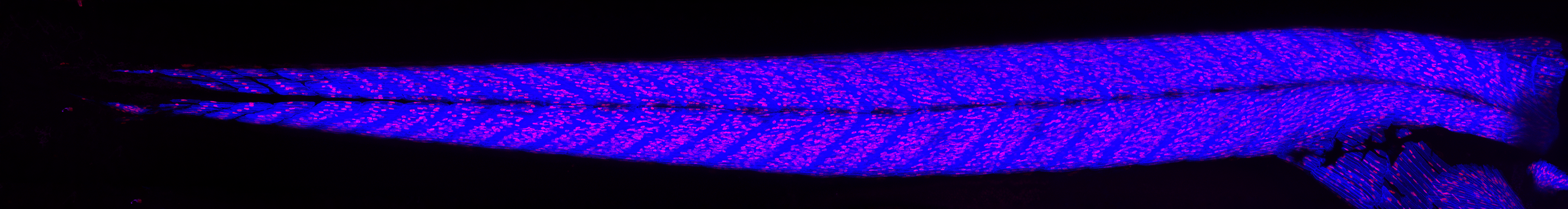

Supplement: Supplementary file 10 — Source data Fig. 1 [file 44318_2024_136_MOESM10_ESM.zip › Figure 1F/10 dpf-Fish 2.tiff]

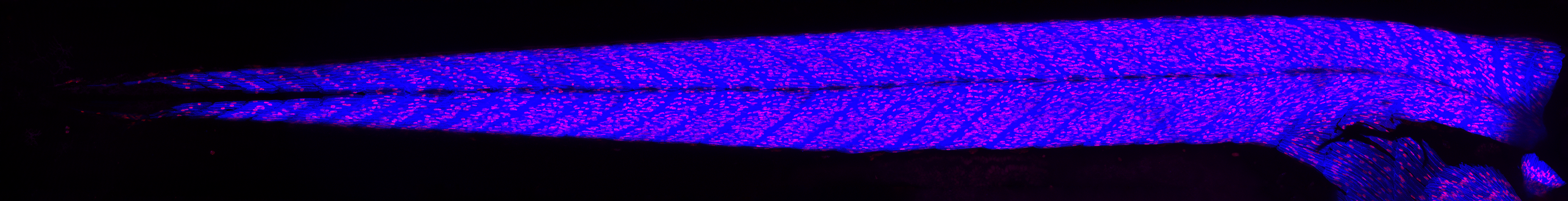

Supplement: Supplementary file 10 — Source data Fig. 1 [file 44318_2024_136_MOESM10_ESM.zip › Figure 1F/10 dpf-Fish 3.tiff]

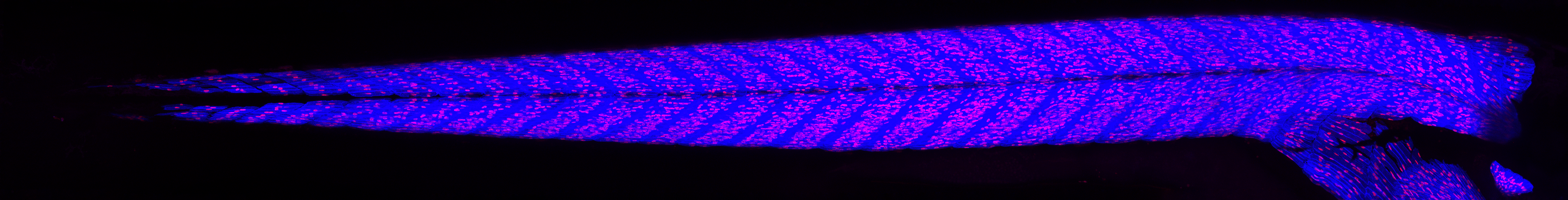

Supplement: Supplementary file 10 — Source data Fig. 1 [file 44318_2024_136_MOESM10_ESM.zip › Figure 1F/10 dpf-Fish 4.tiff]

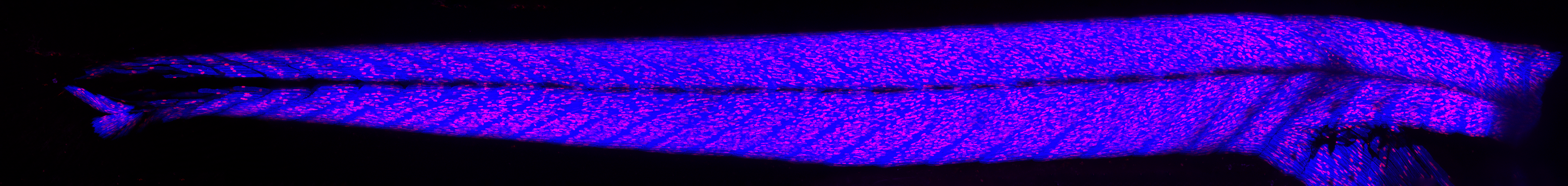

Supplement: Supplementary file 10 — Source data Fig. 1 [file 44318_2024_136_MOESM10_ESM.zip › Figure 1F/14 dpf-Fish 1.tiff]

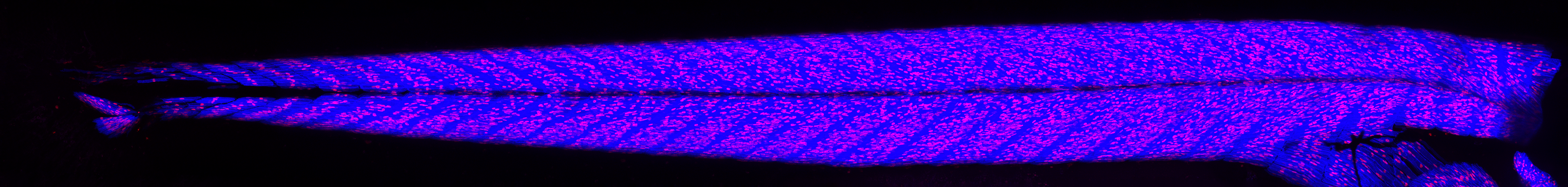

Supplement: Supplementary file 10 — Source data Fig. 1 [file 44318_2024_136_MOESM10_ESM.zip › Figure 1F/14 dpf-Fish 2.tiff]

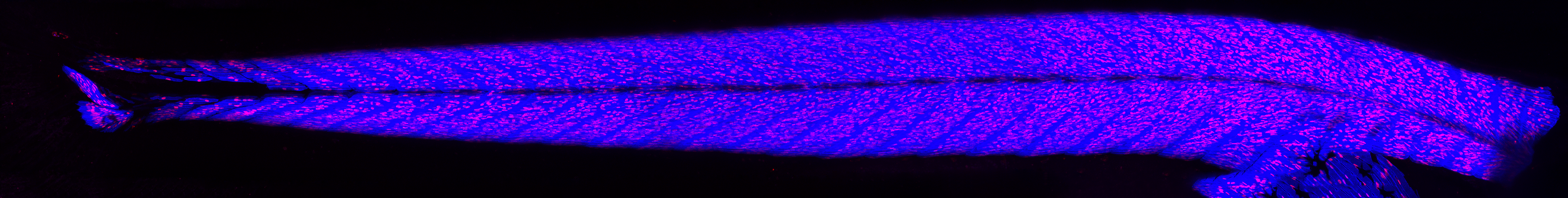

Supplement: Supplementary file 10 — Source data Fig. 1 [file 44318_2024_136_MOESM10_ESM.zip › Figure 1F/14 dpf-Fish 3.tiff]

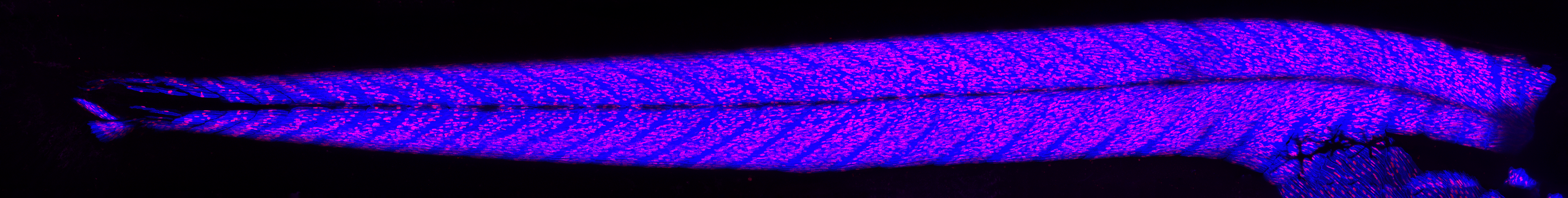

Supplement: Supplementary file 10 — Source data Fig. 1 [file 44318_2024_136_MOESM10_ESM.zip › Figure 1F/14 dpf-Fish 4.tiff]

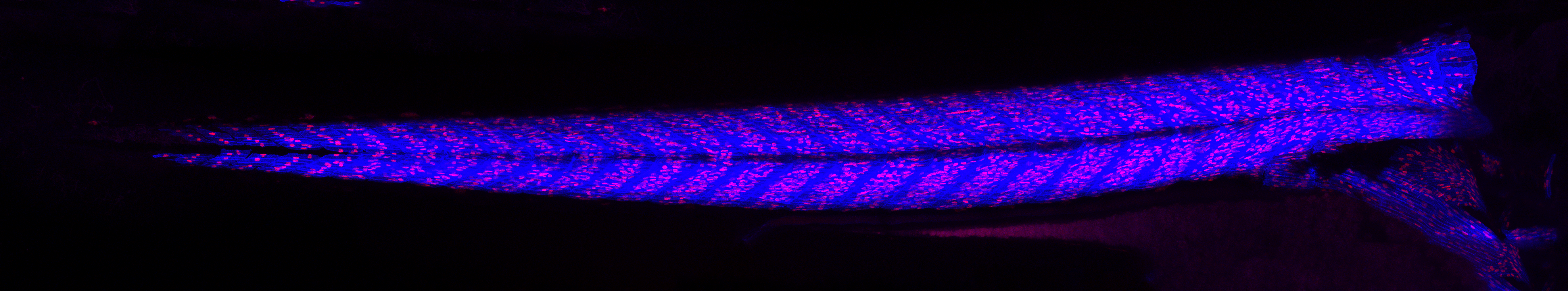

Supplement: Supplementary file 10 — Source data Fig. 1 [file 44318_2024_136_MOESM10_ESM.zip › Figure 1F/6 dpf-Fish 1.tiff]

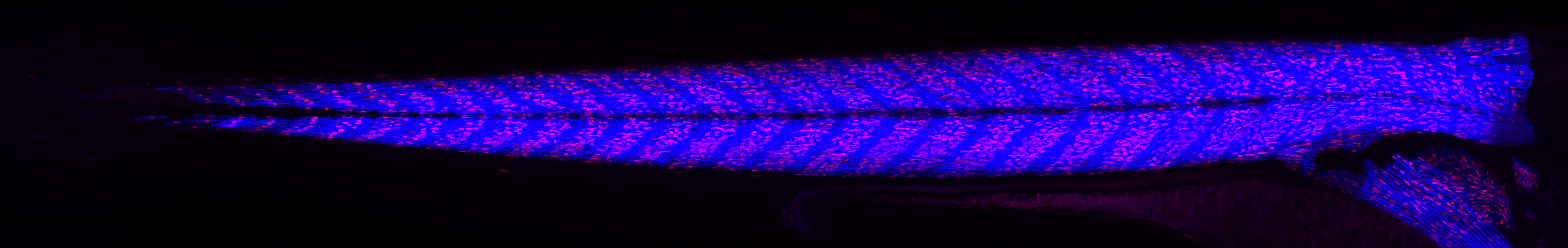

Supplement: Supplementary file 10 — Source data Fig. 1 [file 44318_2024_136_MOESM10_ESM.zip › Figure 1F/6 dpf-Fish 2.tiff]

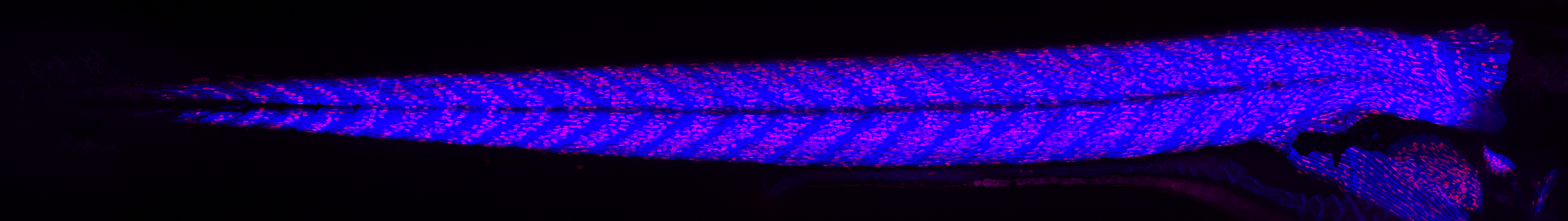

Supplement: Supplementary file 10 — Source data Fig. 1 [file 44318_2024_136_MOESM10_ESM.zip › Figure 1F/6 dpf-Fish 3.tiff]

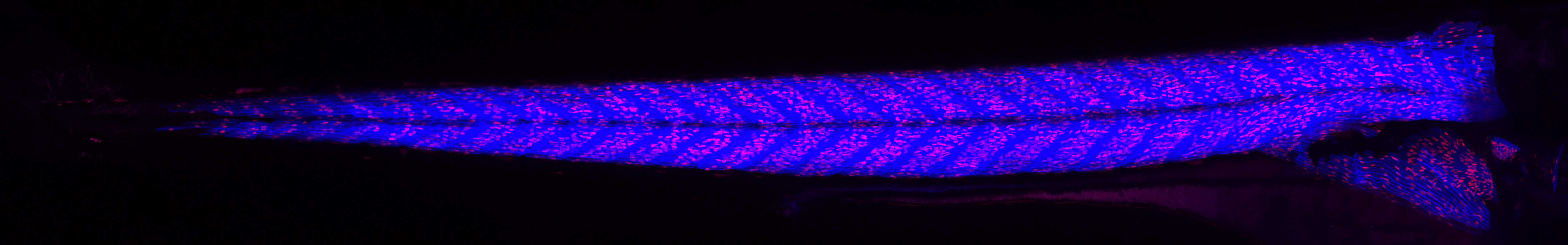

Supplement: Supplementary file 10 — Source data Fig. 1 [file 44318_2024_136_MOESM10_ESM.zip › Figure 1F/6 dpf-Fish 4.tiff]

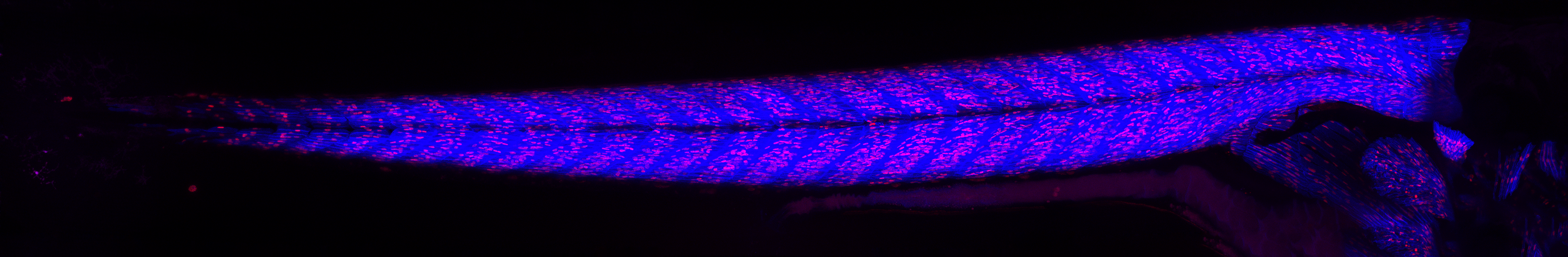

Supplement: Supplementary file 10 — Source data Fig. 1 [file 44318_2024_136_MOESM10_ESM.zip › Figure 1F/8 dpf-Fish 1.tiff]

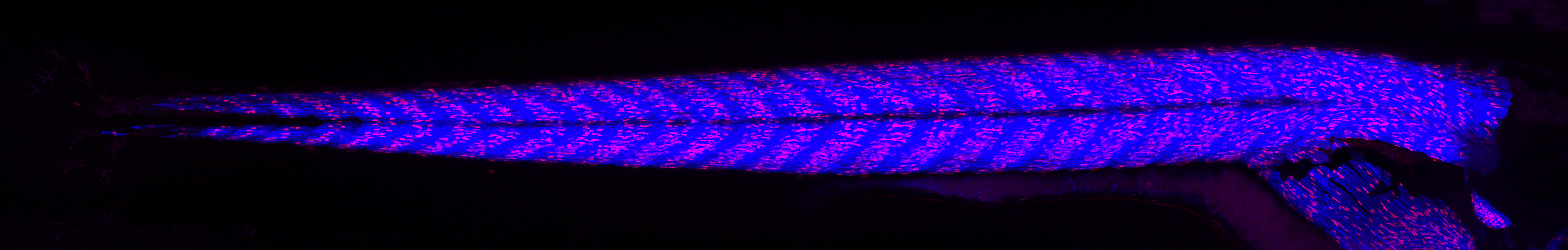

Supplement: Supplementary file 10 — Source data Fig. 1 [file 44318_2024_136_MOESM10_ESM.zip › Figure 1F/8 dpf-Fish 2.tiff]

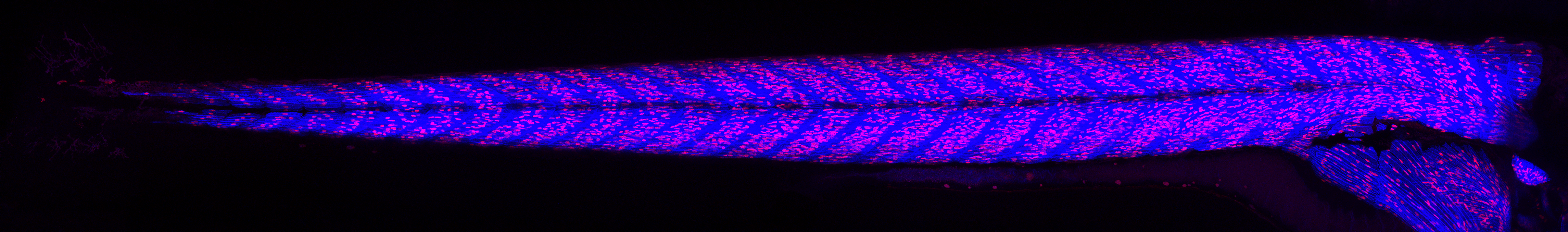

Supplement: Supplementary file 10 — Source data Fig. 1 [file 44318_2024_136_MOESM10_ESM.zip › Figure 1F/8 dpf-Fish 3.tiff]

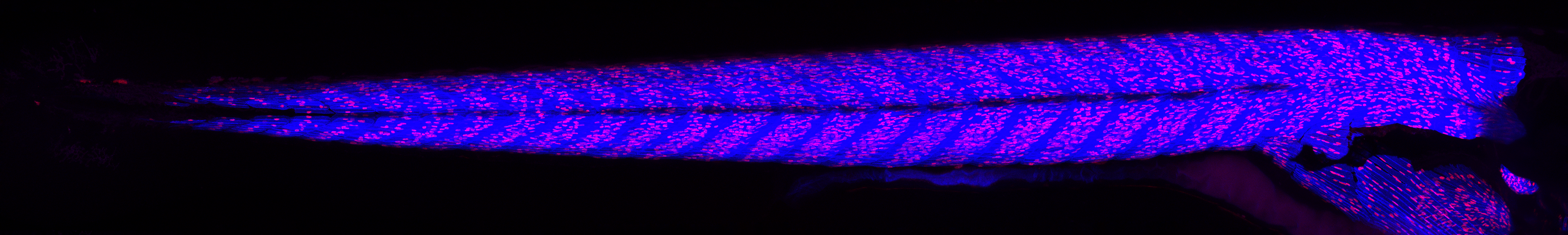

Supplement: Supplementary file 10 — Source data Fig. 1 [file 44318_2024_136_MOESM10_ESM.zip › Figure 1F/8 dpf-Fish 4.tiff]

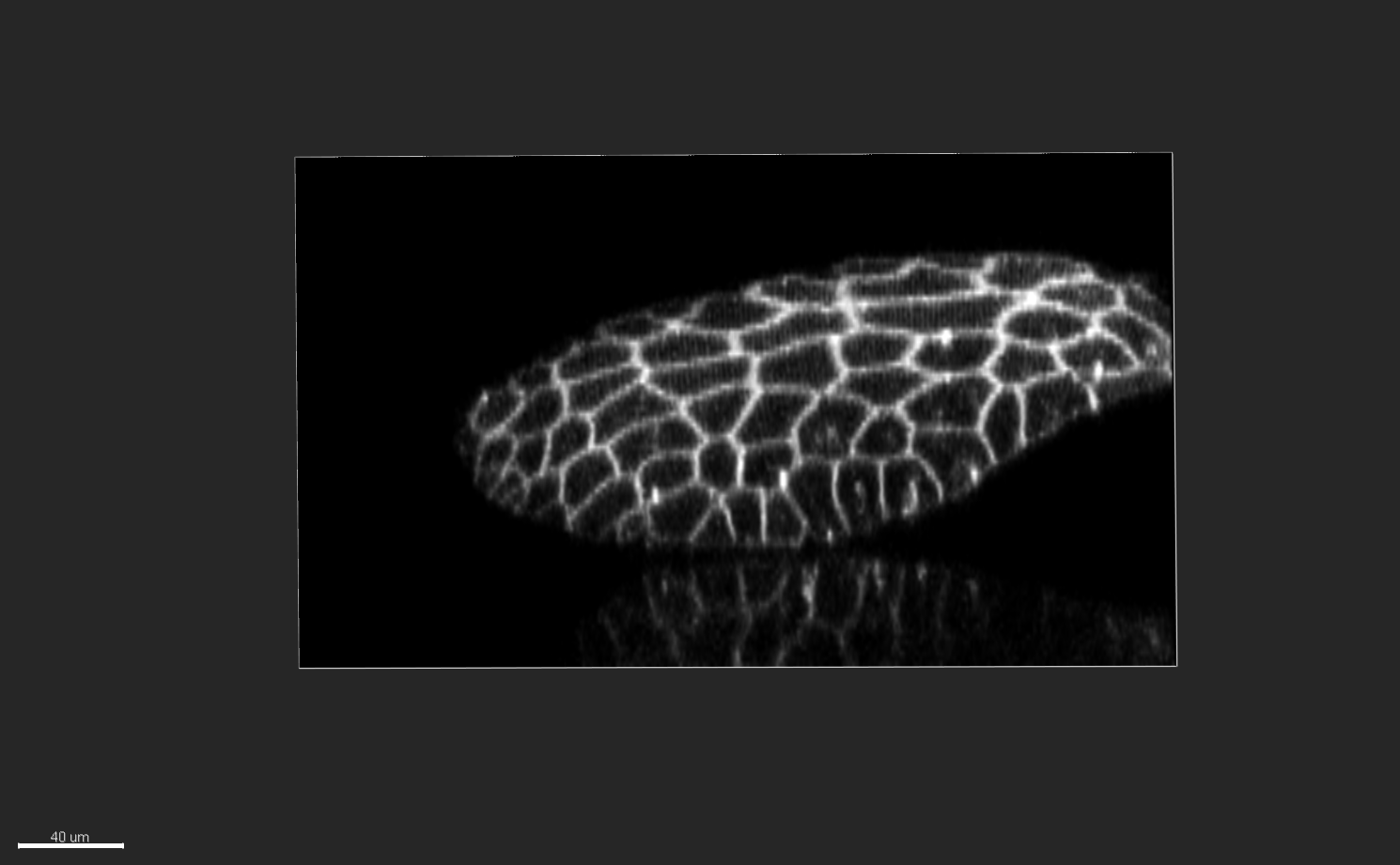

Supplement: Supplementary file 10 — Source data Fig. 1 [file 44318_2024_136_MOESM10_ESM.zip › Figure 1H/10 dpf.tif]

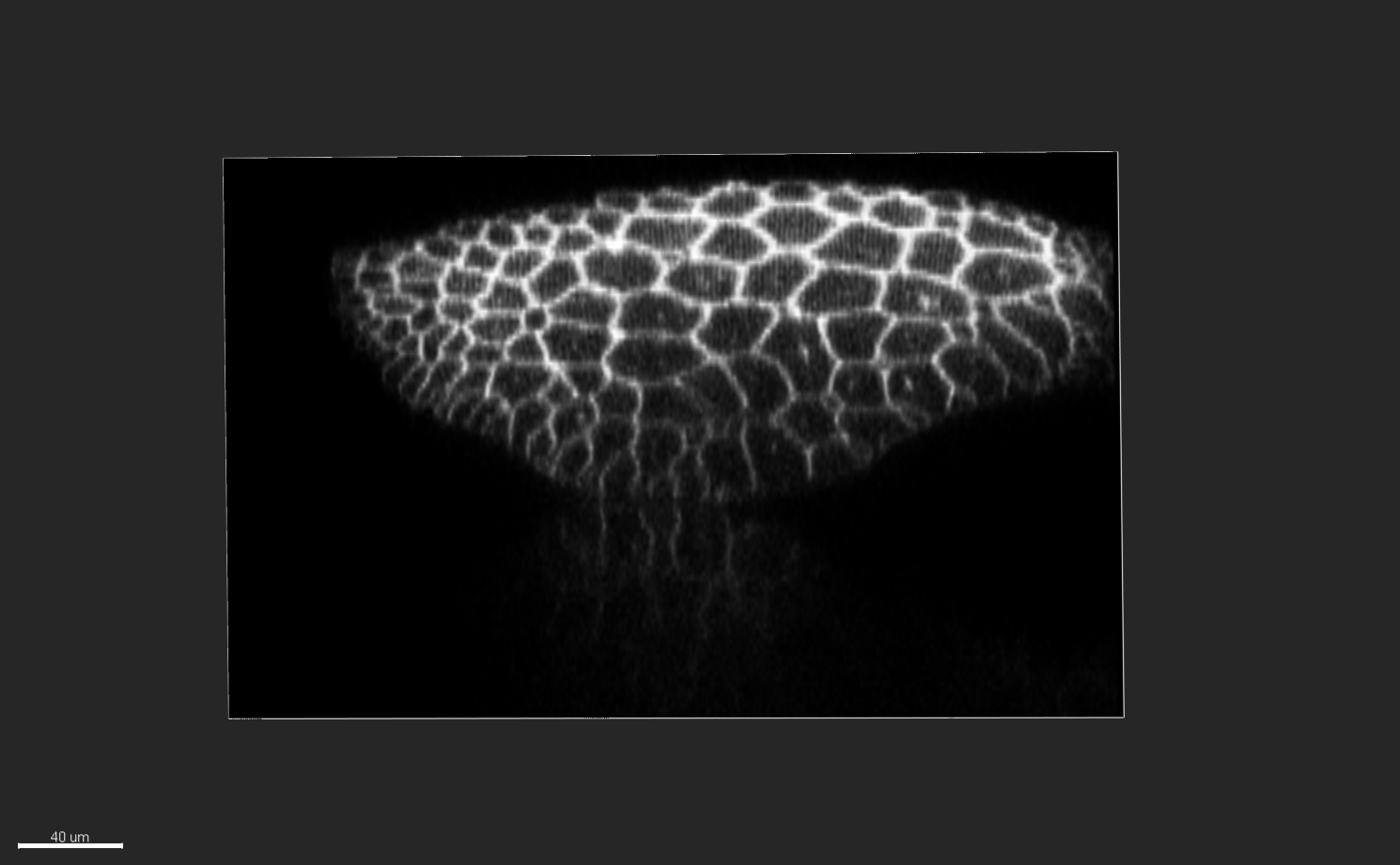

Supplement: Supplementary file 10 — Source data Fig. 1 [file 44318_2024_136_MOESM10_ESM.zip › Figure 1H/14 dpf.tif]

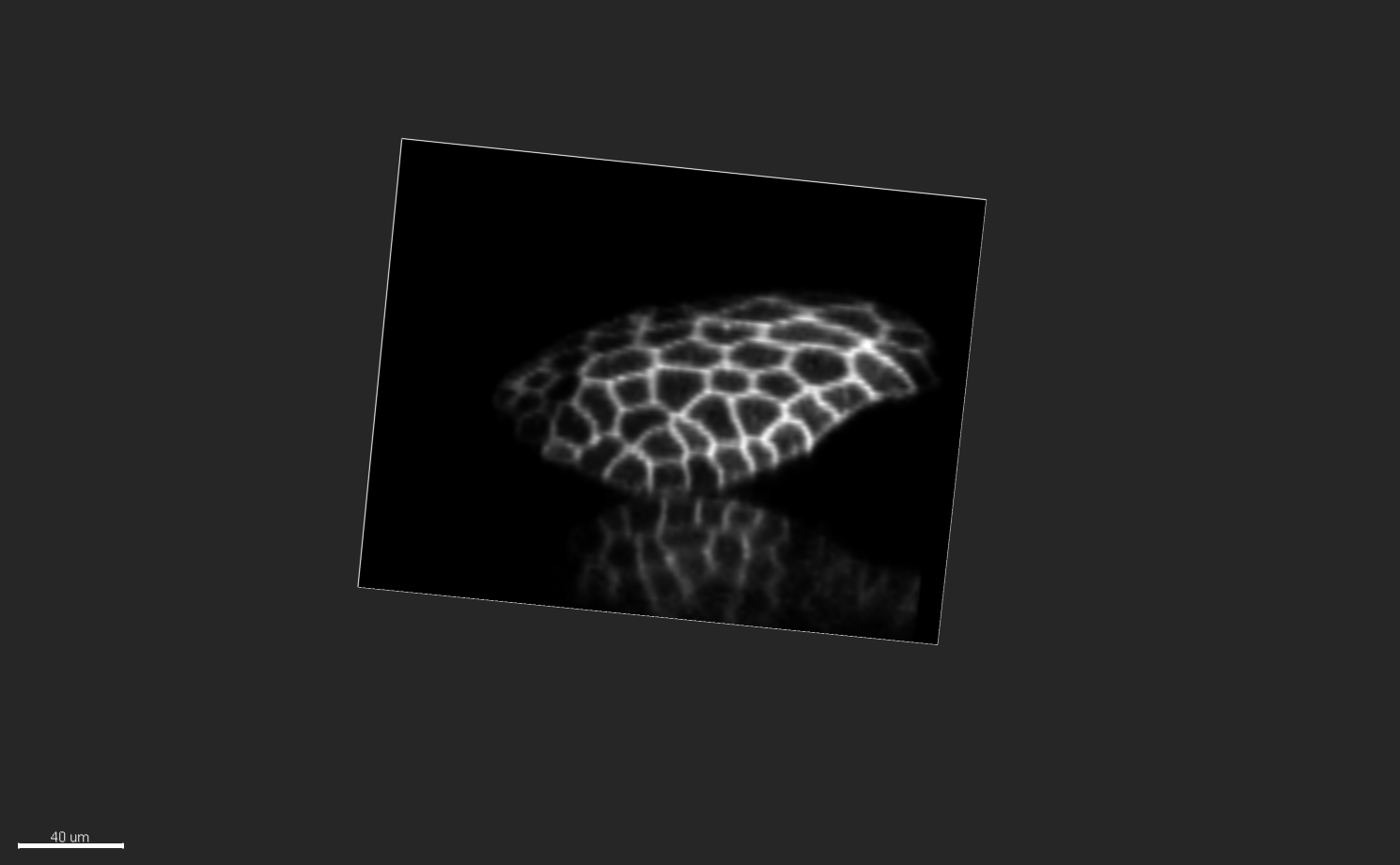

Supplement: Supplementary file 10 — Source data Fig. 1 [file 44318_2024_136_MOESM10_ESM.zip › Figure 1H/6 dpf.tif]

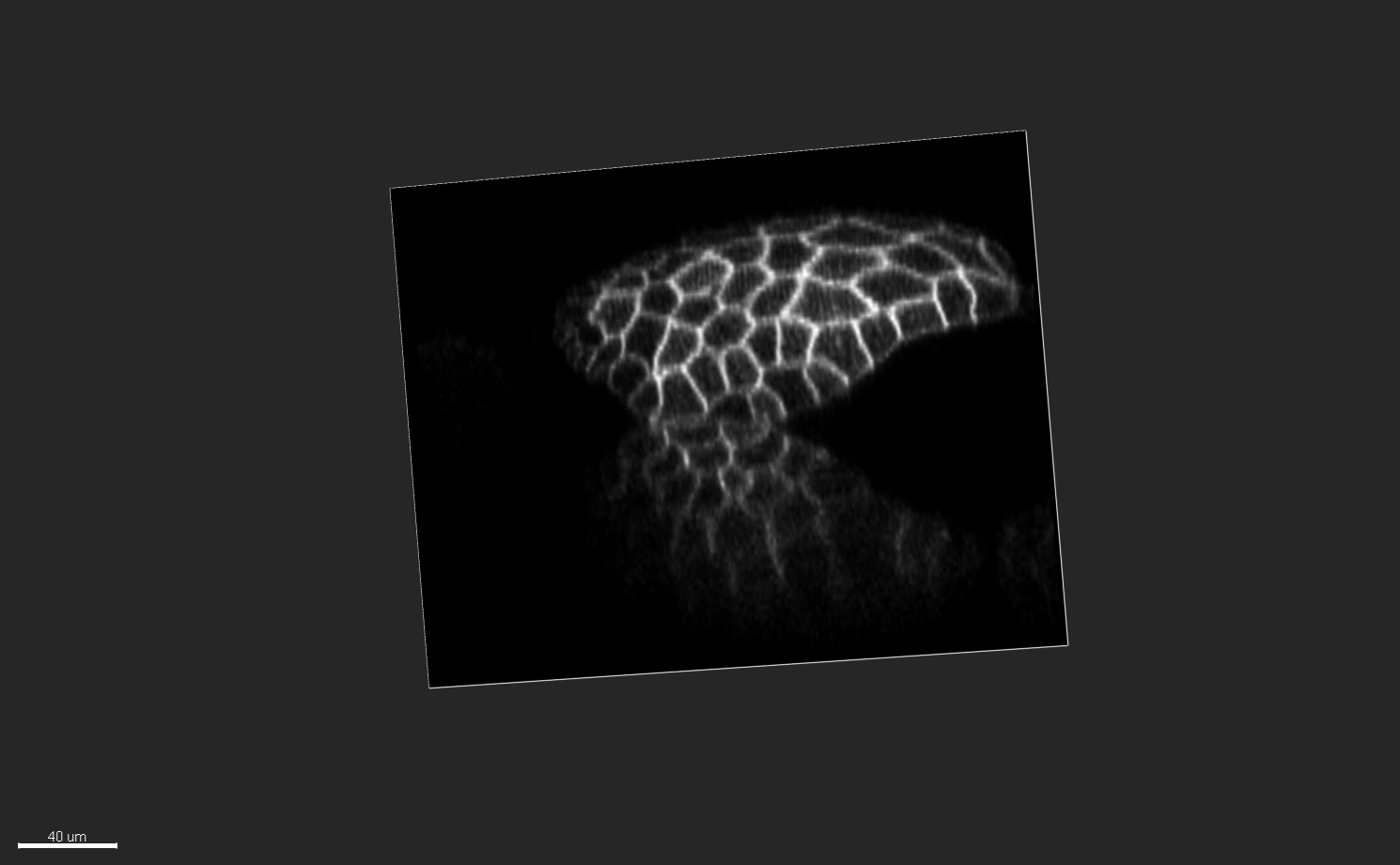

Supplement: Supplementary file 10 — Source data Fig. 1 [file 44318_2024_136_MOESM10_ESM.zip › Figure 1H/8 dpf.tif]

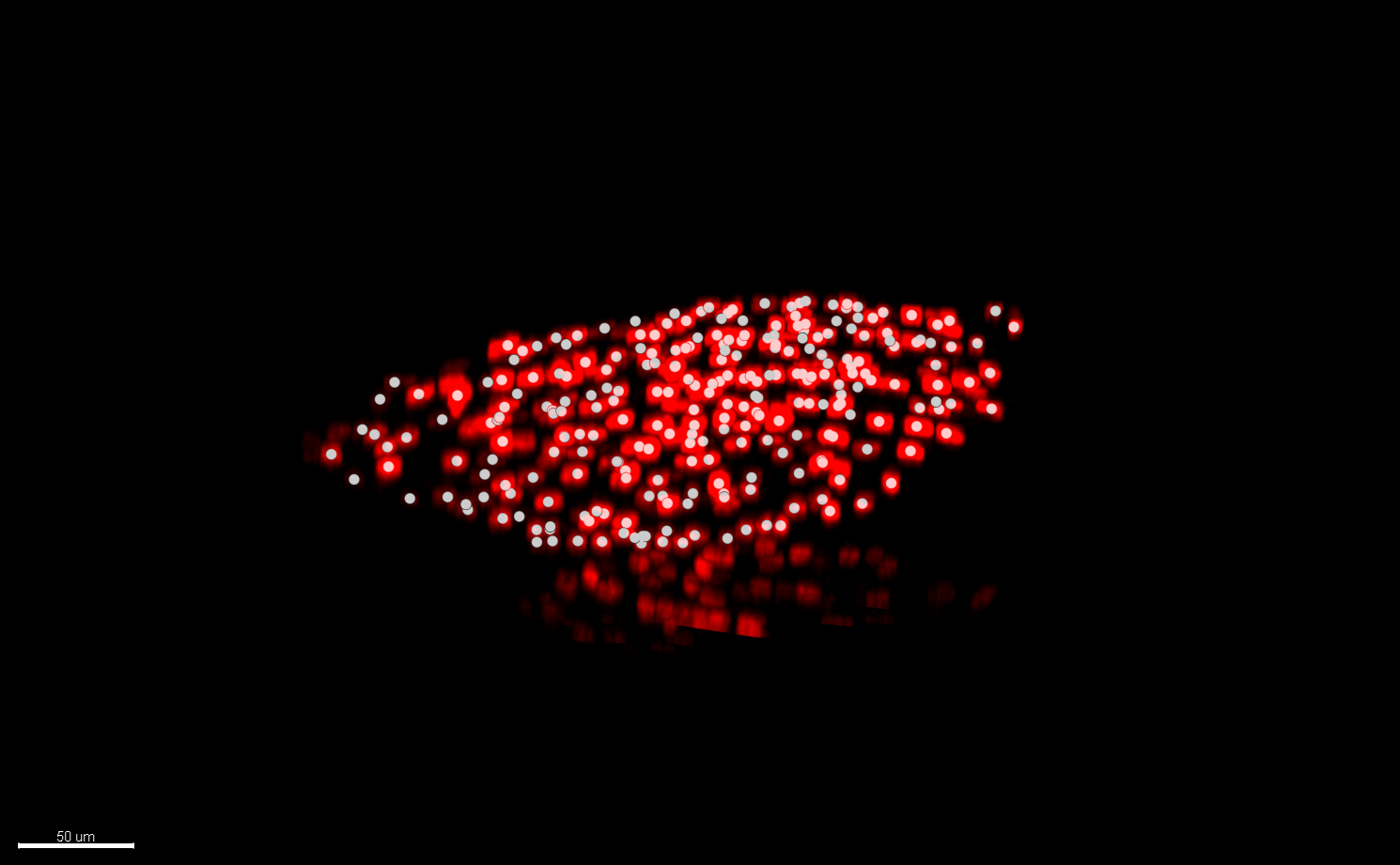

Supplement: Supplementary file 10 — Source data Fig. 1 [file 44318_2024_136_MOESM10_ESM.zip › Figure 1I/10 dpf.tif]

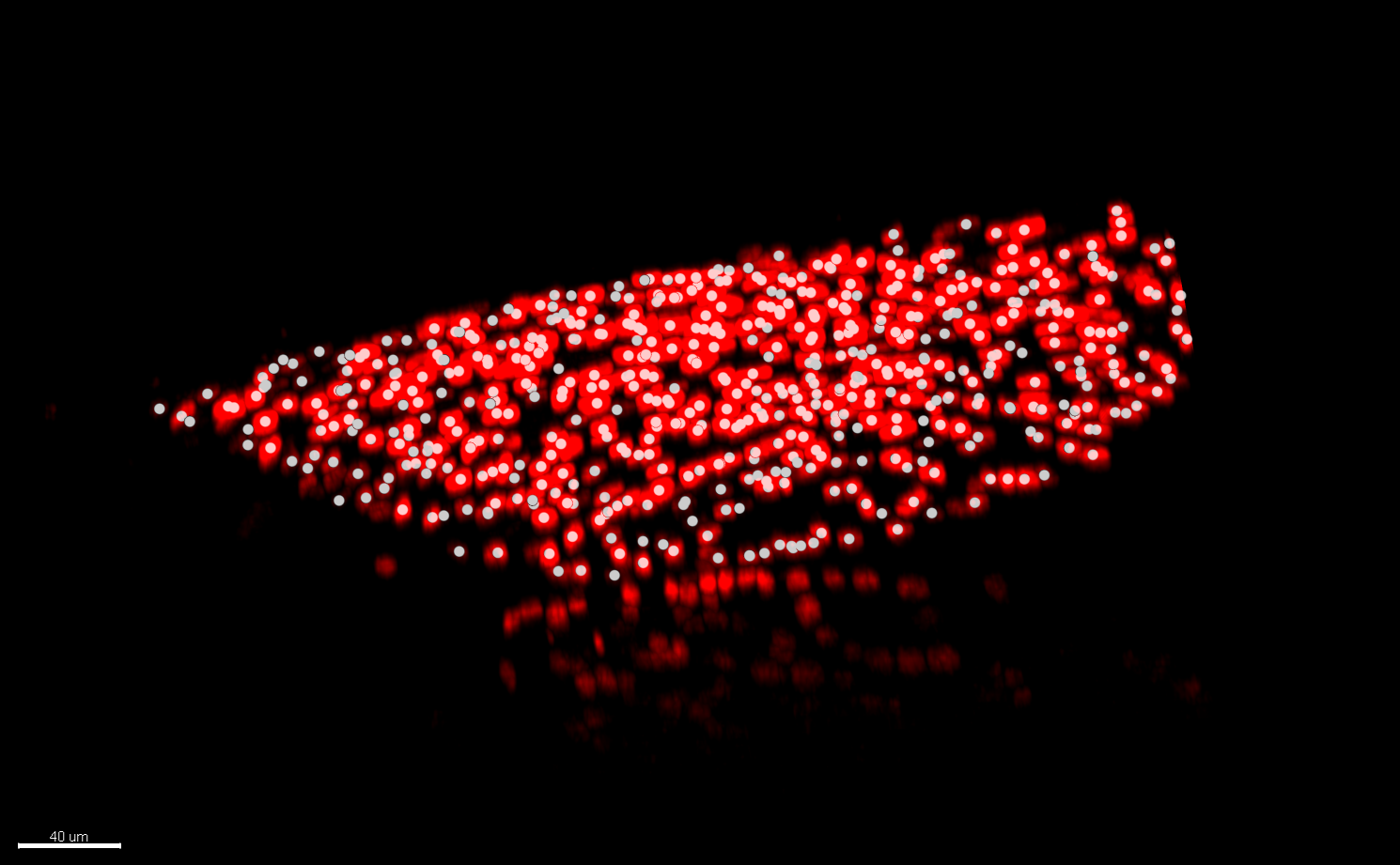

Supplement: Supplementary file 10 — Source data Fig. 1 [file 44318_2024_136_MOESM10_ESM.zip › Figure 1I/14 dpf.tif]

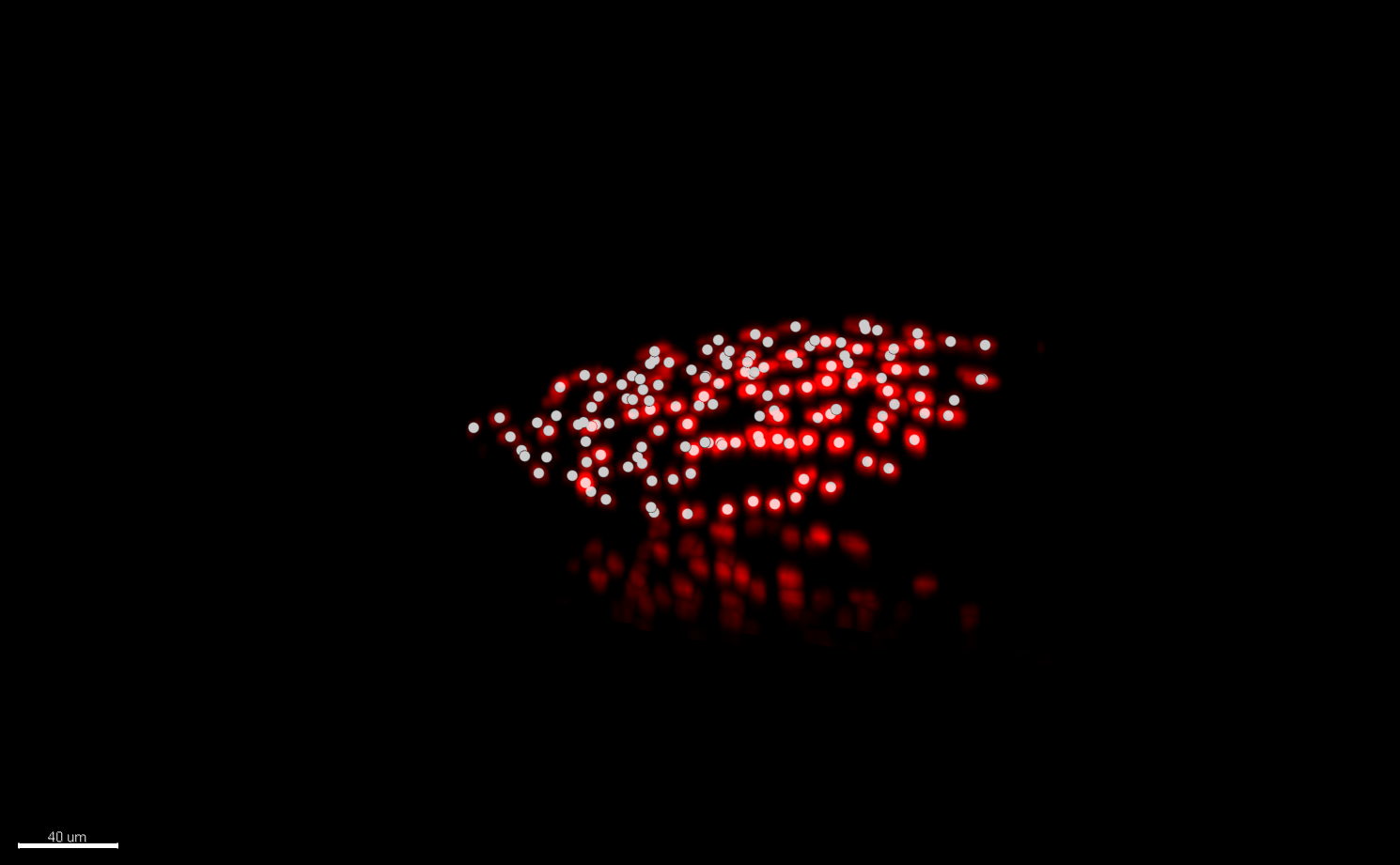

Supplement: Supplementary file 10 — Source data Fig. 1 [file 44318_2024_136_MOESM10_ESM.zip › Figure 1I/6 dpf.tif]

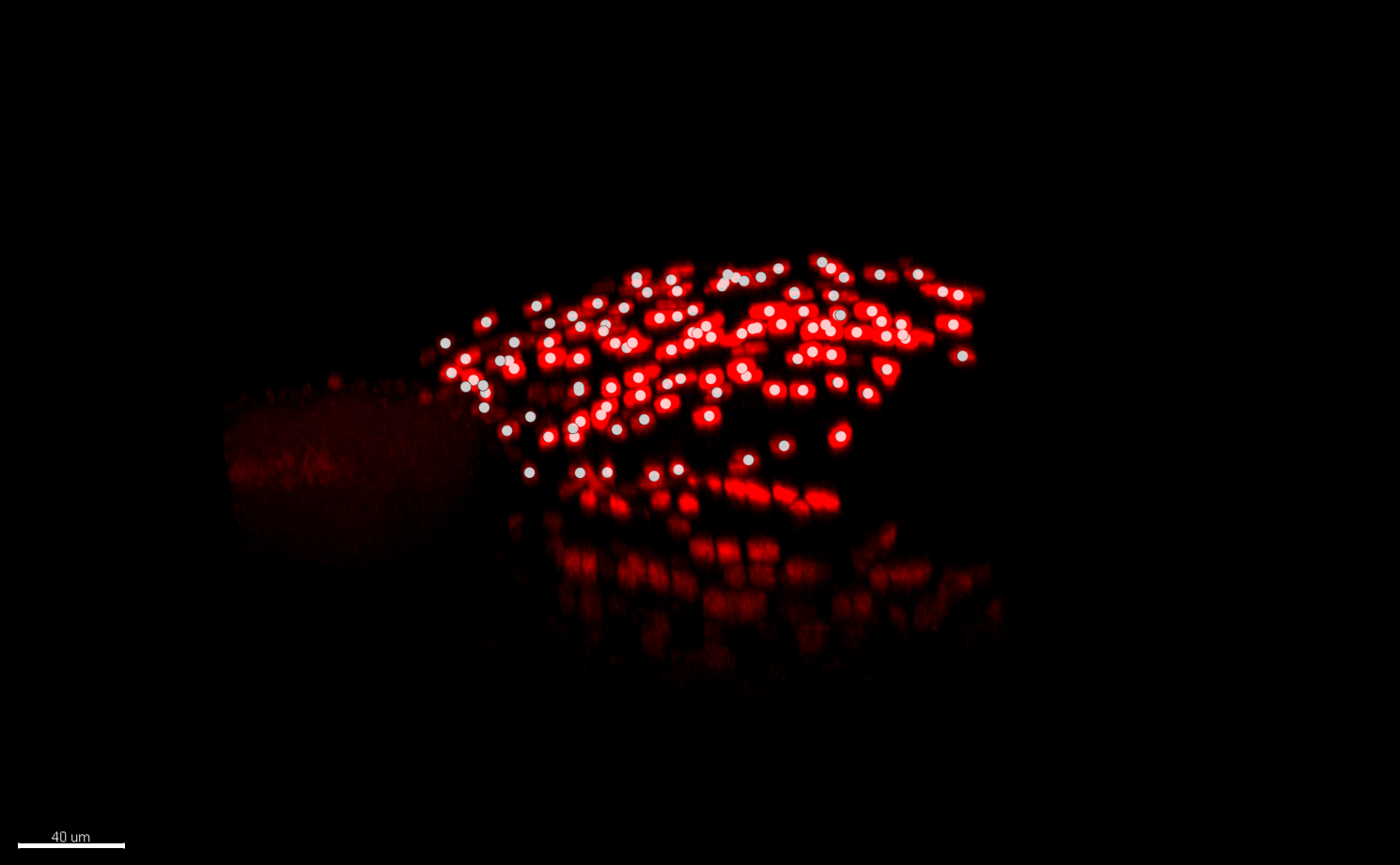

Supplement: Supplementary file 10 — Source data Fig. 1 [file 44318_2024_136_MOESM10_ESM.zip › Figure 1I/8 dpf.tif]

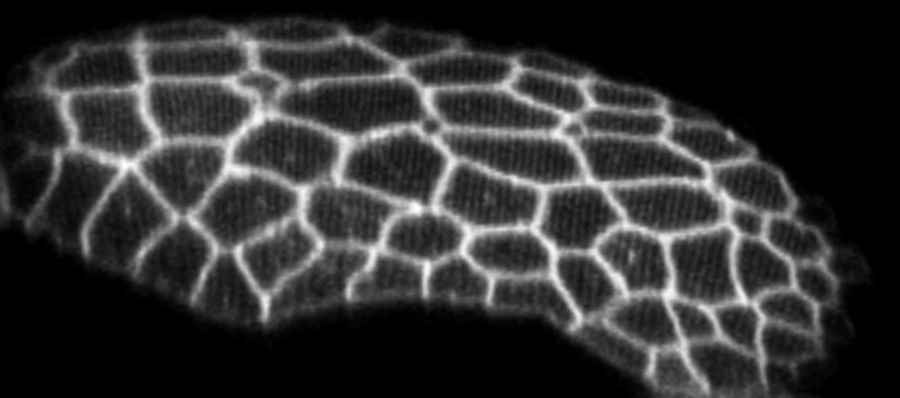

Supplement: Supplementary file 11 — Source data Fig. 2 [file 44318_2024_136_MOESM11_ESM.zip › Figure 2B/Dorsal myotome-FG-10 dpf.tif]

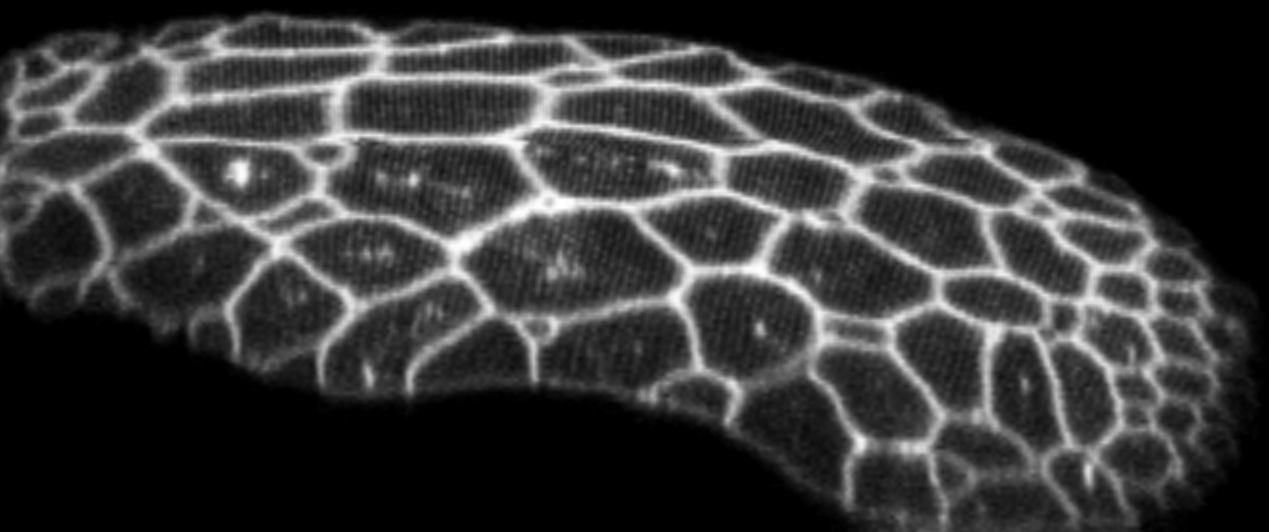

Supplement: Supplementary file 11 — Source data Fig. 2 [file 44318_2024_136_MOESM11_ESM.zip › Figure 2B/Dorsal myotome-FG-14 dpf.tif]

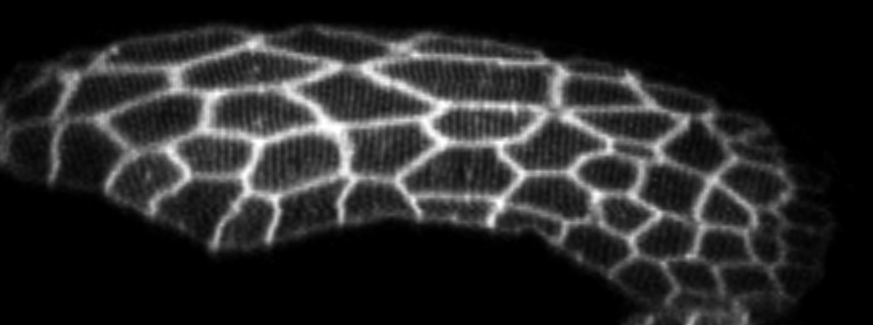

Supplement: Supplementary file 11 — Source data Fig. 2 [file 44318_2024_136_MOESM11_ESM.zip › Figure 2B/Dorsal myotome-SG-10 dpf.tif]

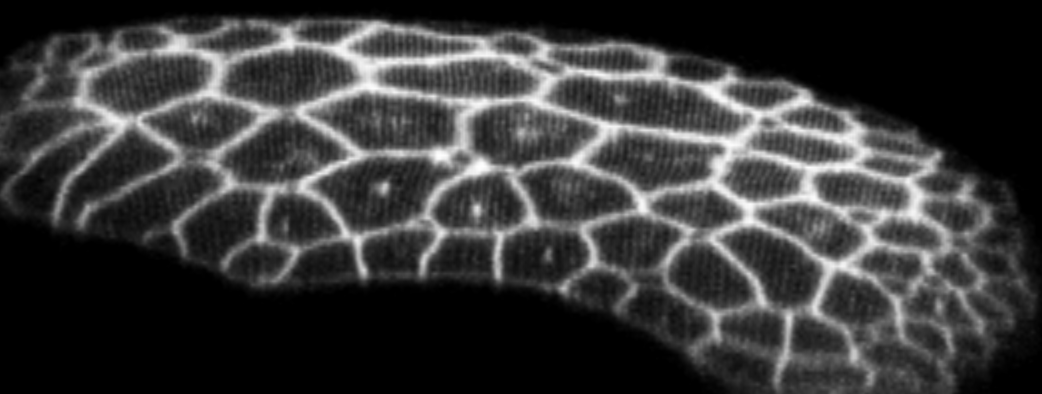

Supplement: Supplementary file 11 — Source data Fig. 2 [file 44318_2024_136_MOESM11_ESM.zip › Figure 2B/Dorsal myotome-SG-14 dpf.tif]

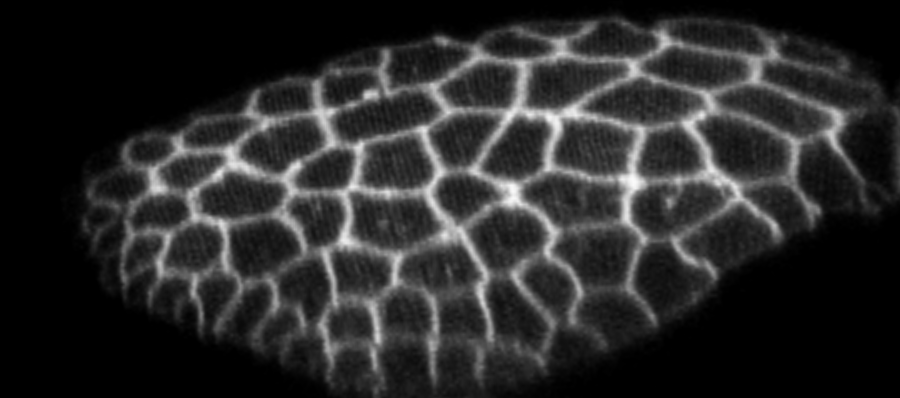

Supplement: Supplementary file 11 — Source data Fig. 2 [file 44318_2024_136_MOESM11_ESM.zip › Figure 2B/Ventral myotome-FG-10 dpf.tif]

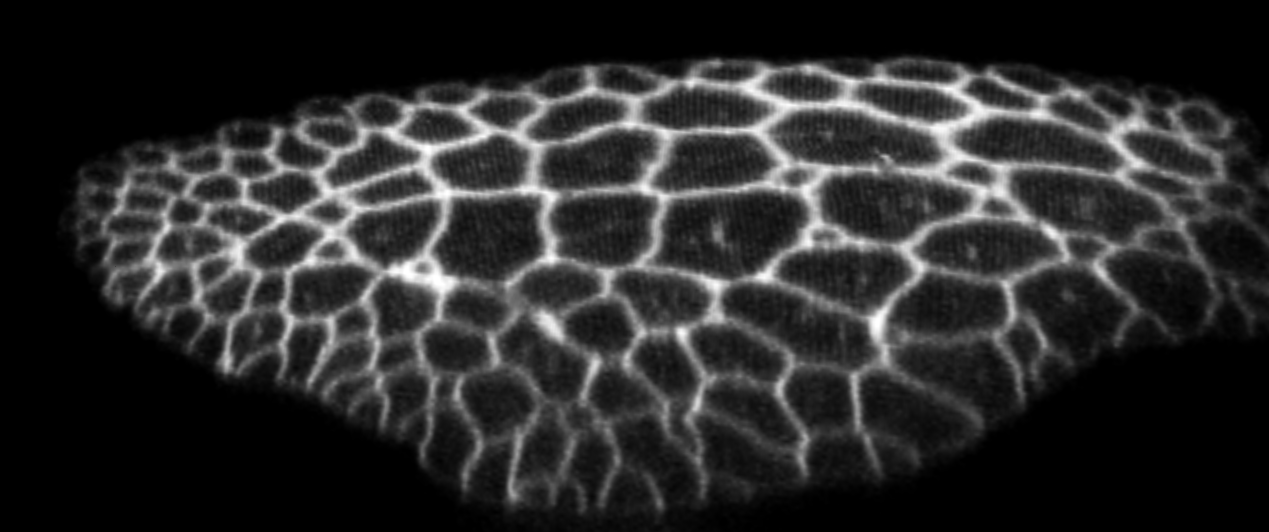

Supplement: Supplementary file 11 — Source data Fig. 2 [file 44318_2024_136_MOESM11_ESM.zip › Figure 2B/Ventral myotome-FG-14 dpf.tif]

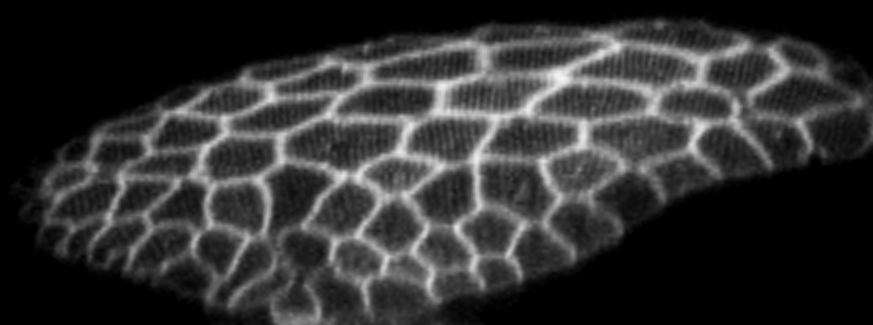

Supplement: Supplementary file 11 — Source data Fig. 2 [file 44318_2024_136_MOESM11_ESM.zip › Figure 2B/Ventral myotome-SG-10 dpf.tif]

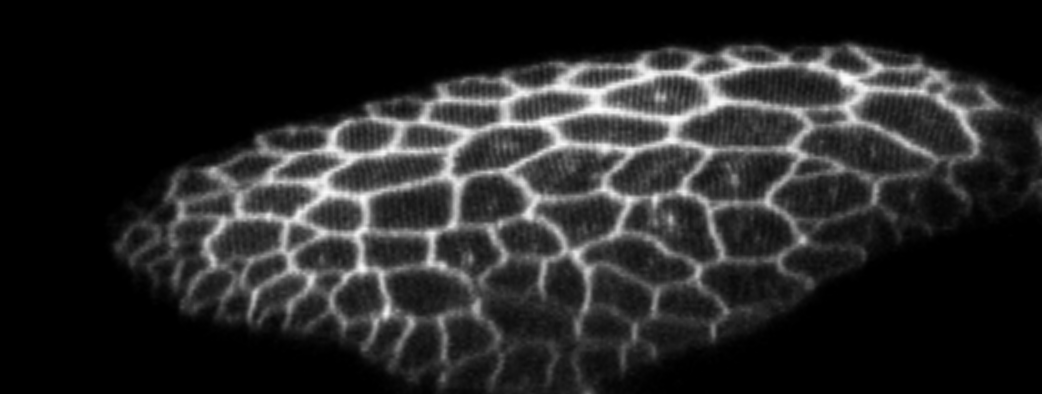

Supplement: Supplementary file 11 — Source data Fig. 2 [file 44318_2024_136_MOESM11_ESM.zip › Figure 2B/Ventral myotome-SG-14 dpf.tif]

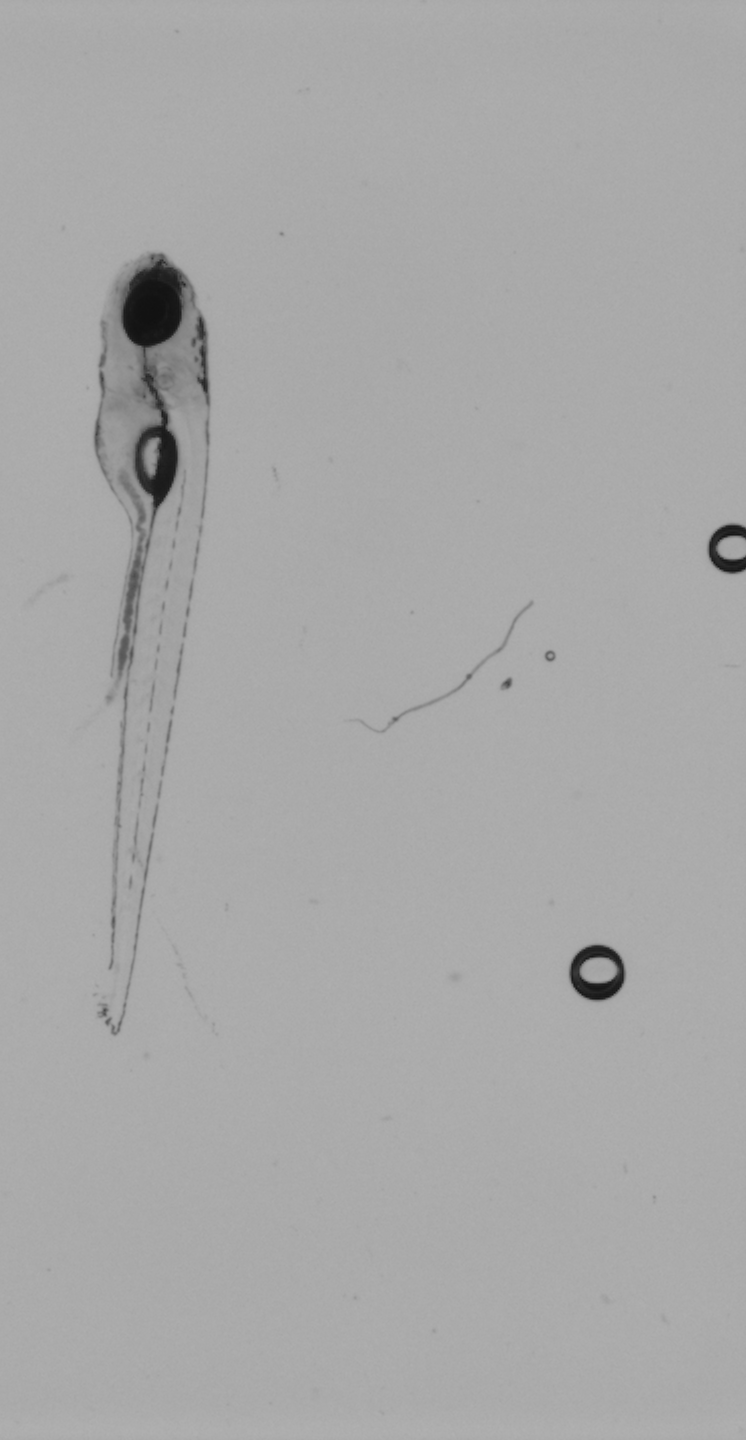

Supplement: Supplementary file 11 — Source data Fig. 2 [file 44318_2024_136_MOESM11_ESM.zip › Figure 2C-D/10 dpf-Standard length and Trunk surface area-Fish 10_SG.tif]

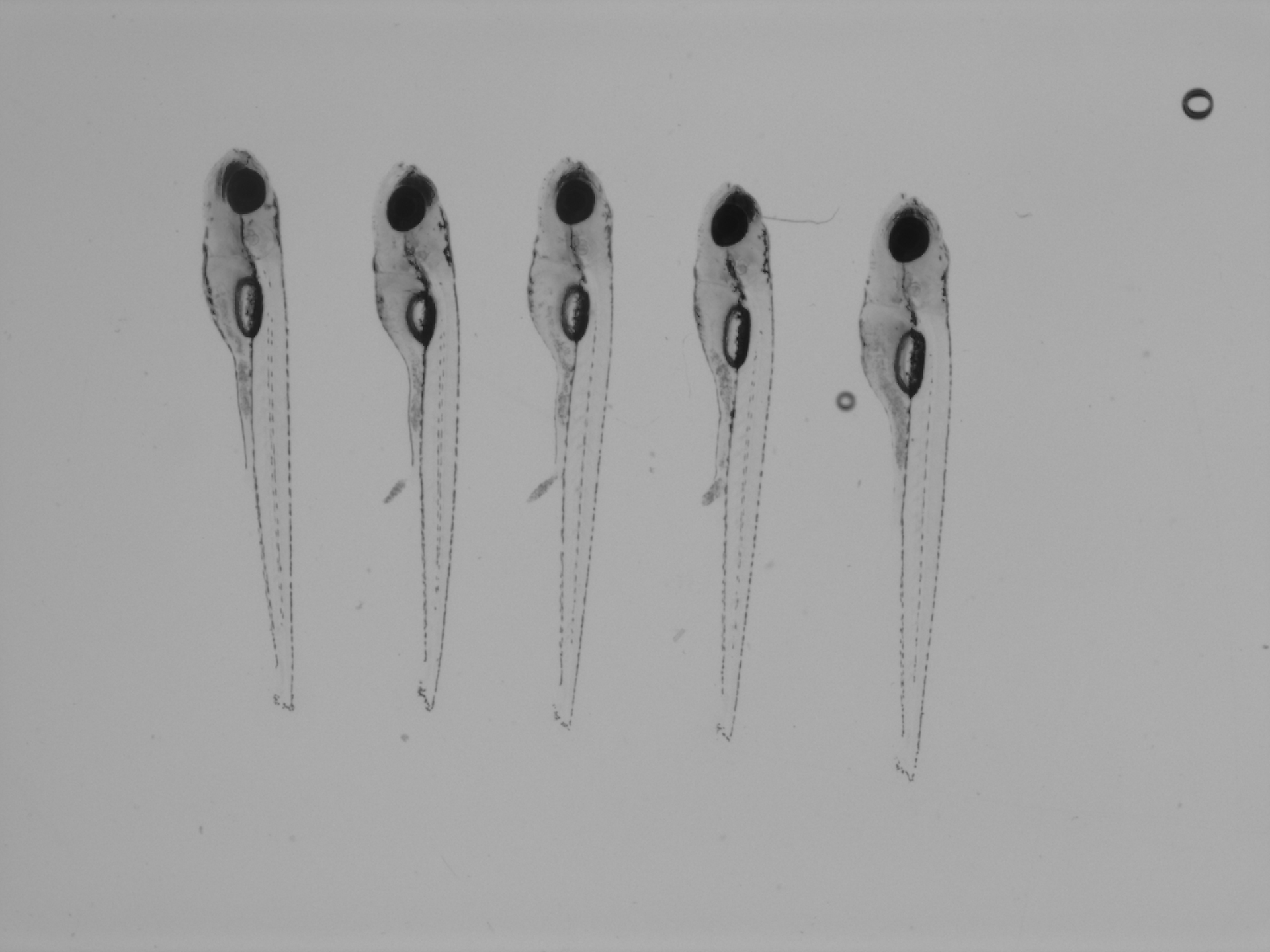

Supplement: Supplementary file 11 — Source data Fig. 2 [file 44318_2024_136_MOESM11_ESM.zip › Figure 2C-D/10 dpf-Standard length and Trunk surface area-Fish 1-5_FG.tif]

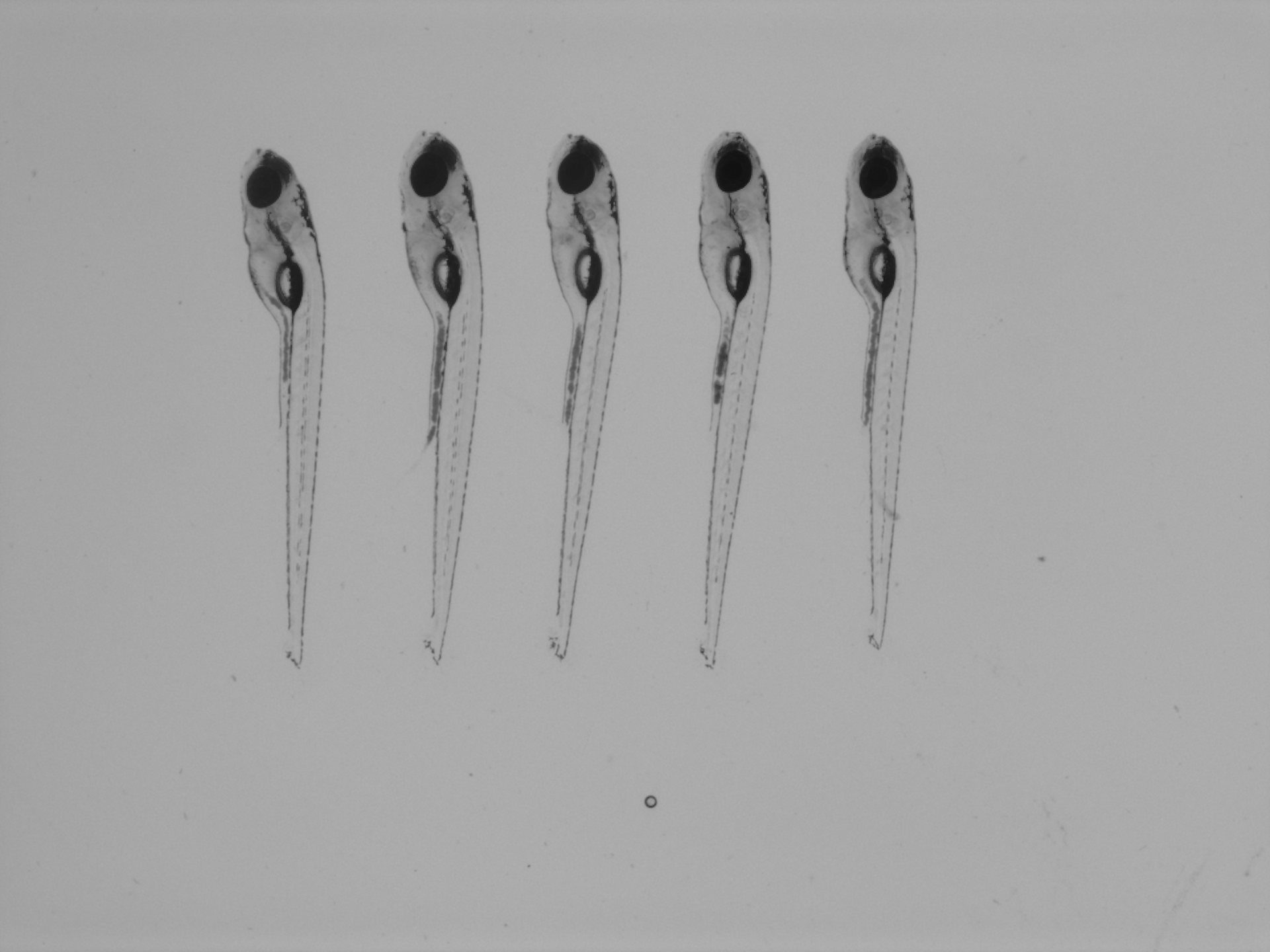

Supplement: Supplementary file 11 — Source data Fig. 2 [file 44318_2024_136_MOESM11_ESM.zip › Figure 2C-D/10 dpf-Standard length and Trunk surface area-Fish 1-5_SG.tif]

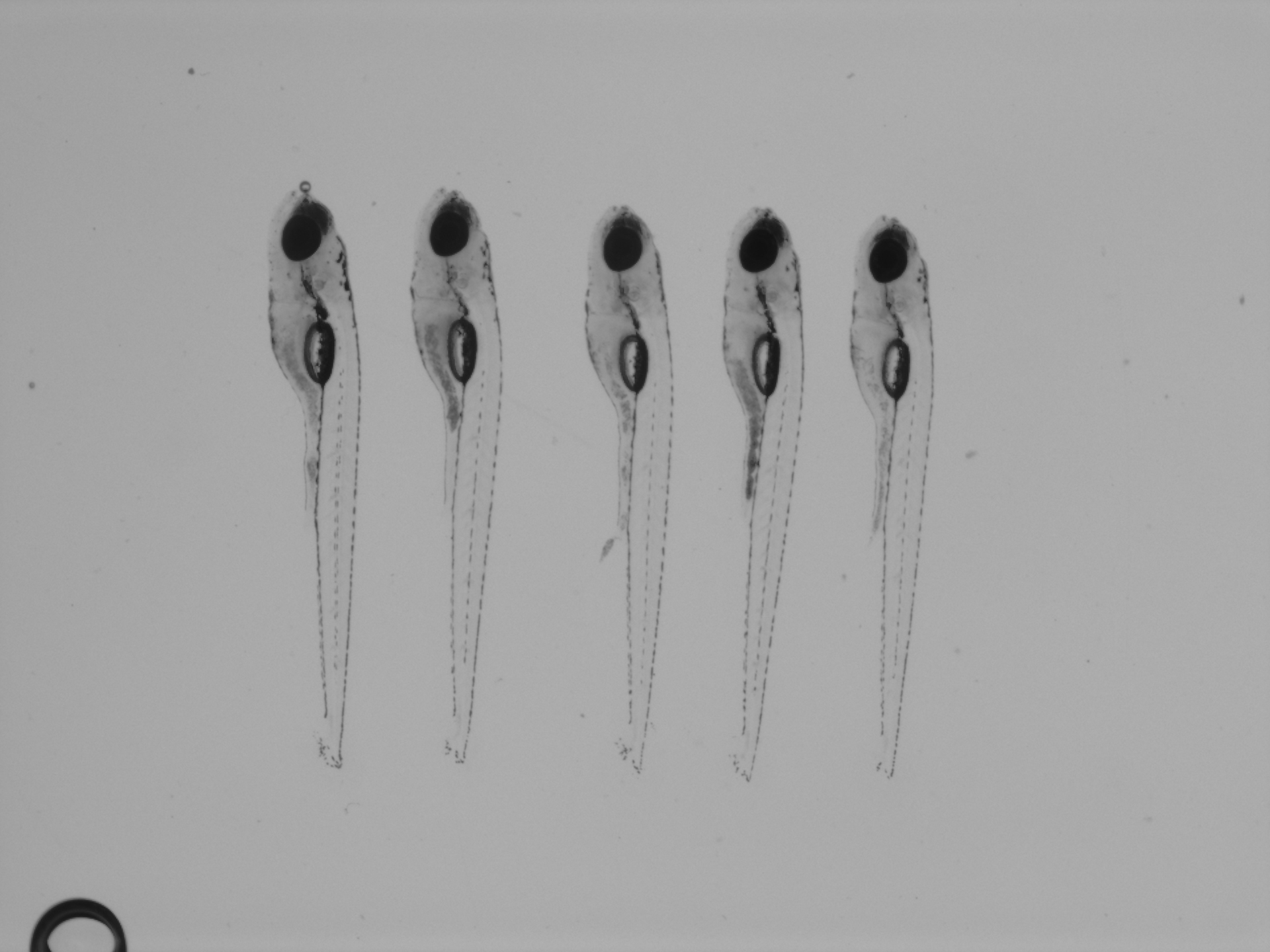

Supplement: Supplementary file 11 — Source data Fig. 2 [file 44318_2024_136_MOESM11_ESM.zip › Figure 2C-D/10 dpf-Standard length and Trunk surface area-Fish 6-10_FG.tif]

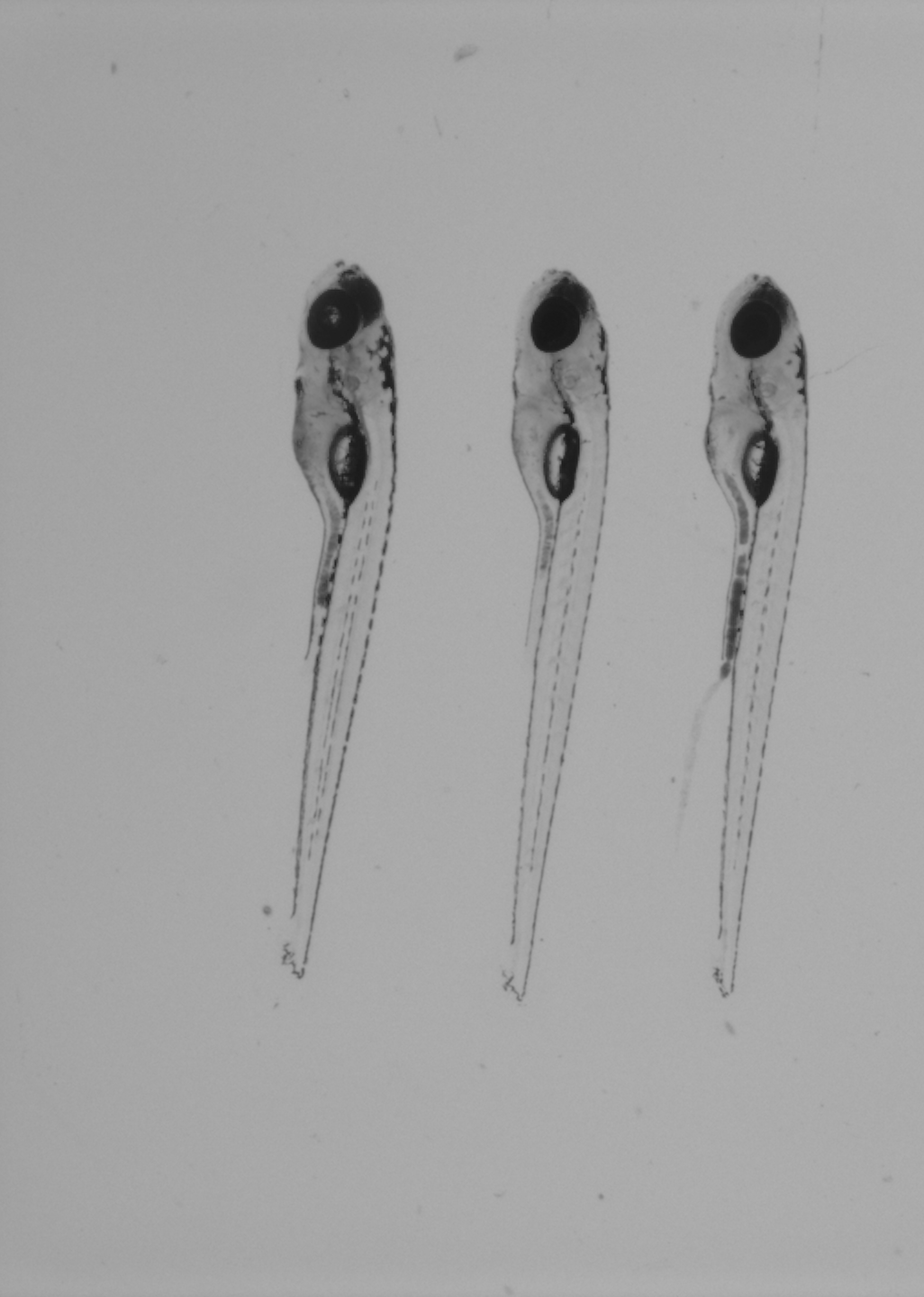

Supplement: Supplementary file 11 — Source data Fig. 2 [file 44318_2024_136_MOESM11_ESM.zip › Figure 2C-D/10 dpf-Standard length and Trunk surface area-Fish 6-8_SG.tif]

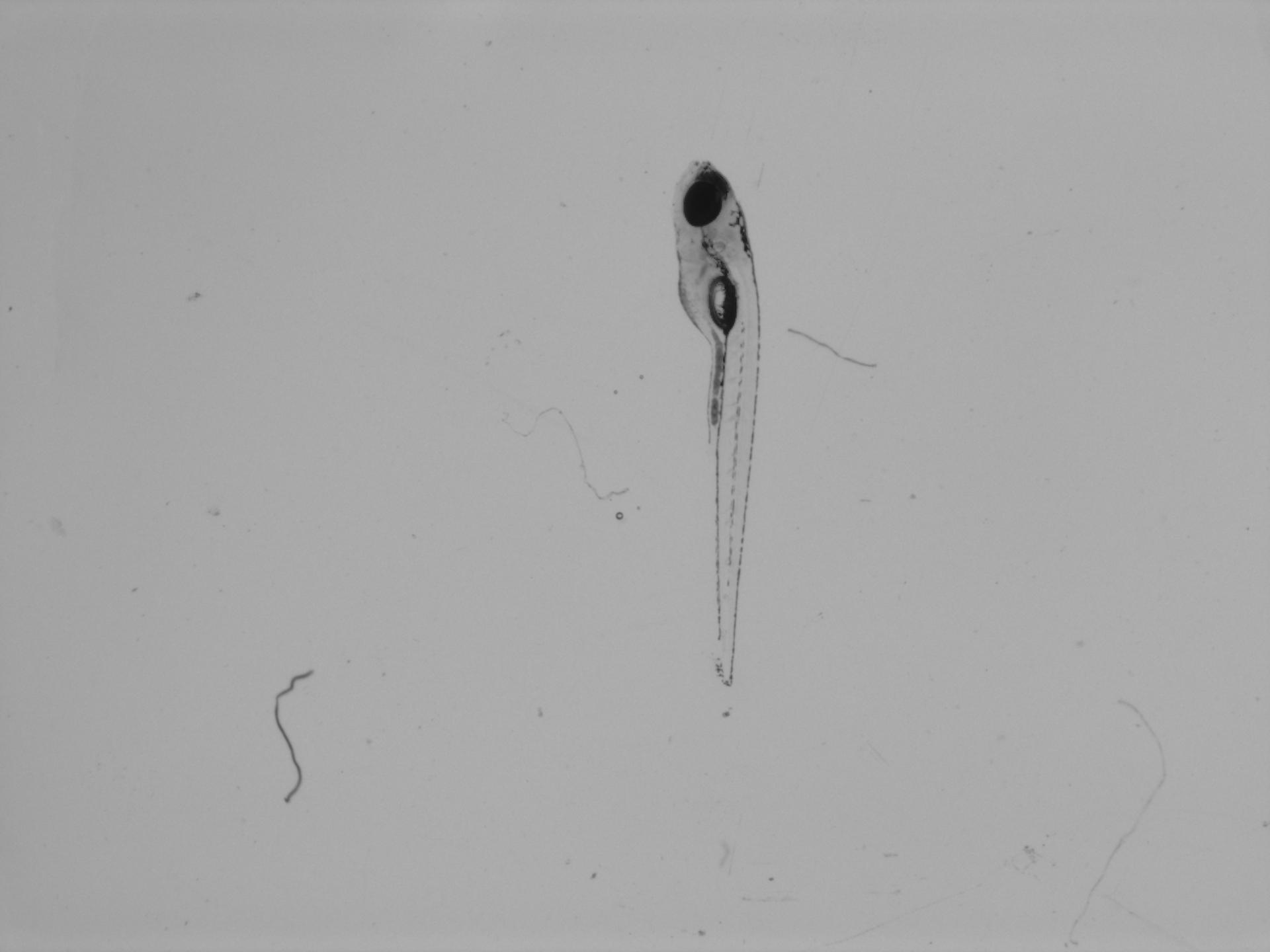

Supplement: Supplementary file 11 — Source data Fig. 2 [file 44318_2024_136_MOESM11_ESM.zip › Figure 2C-D/10 dpf-Standard length and Trunk surface area-Fish 9_SG.tif]

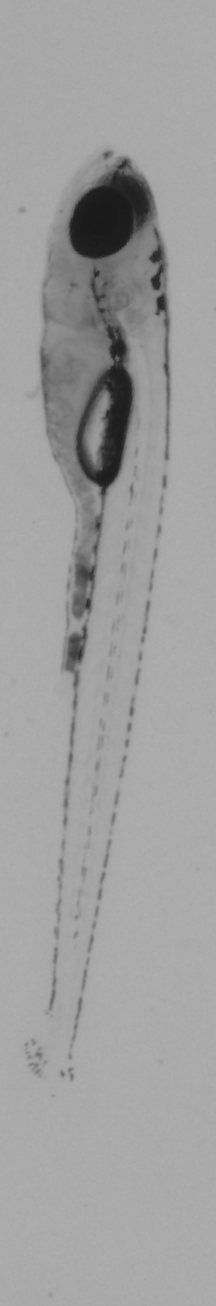

Supplement: Supplementary file 11 — Source data Fig. 2 [file 44318_2024_136_MOESM11_ESM.zip › Figure 2C-D/14 dpf-Standard length and Trunk surface area-Fish 1_SG.tif]

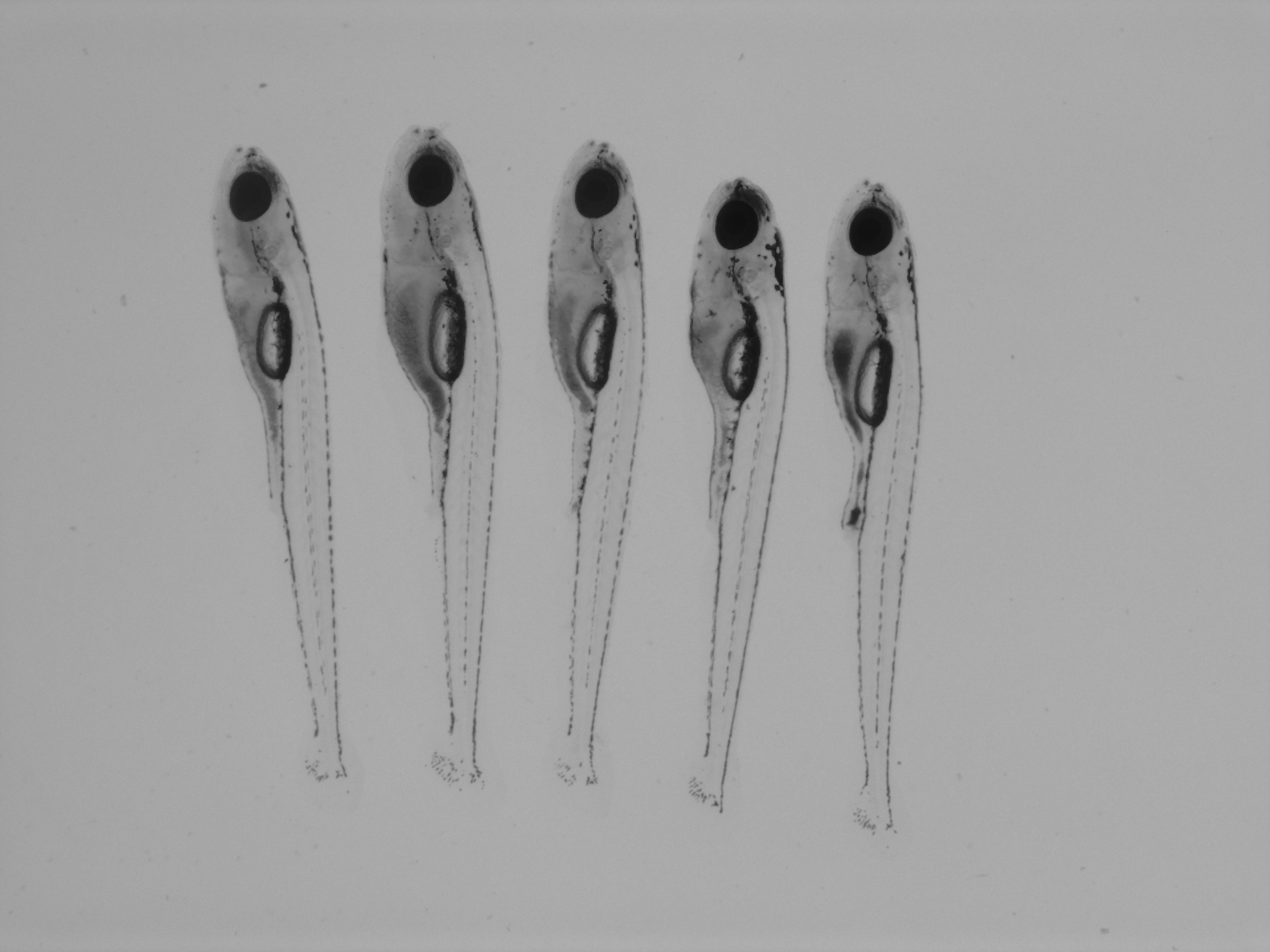

Supplement: Supplementary file 11 — Source data Fig. 2 [file 44318_2024_136_MOESM11_ESM.zip › Figure 2C-D/14 dpf-Standard length and Trunk surface area-Fish 1-5_FG.tif]

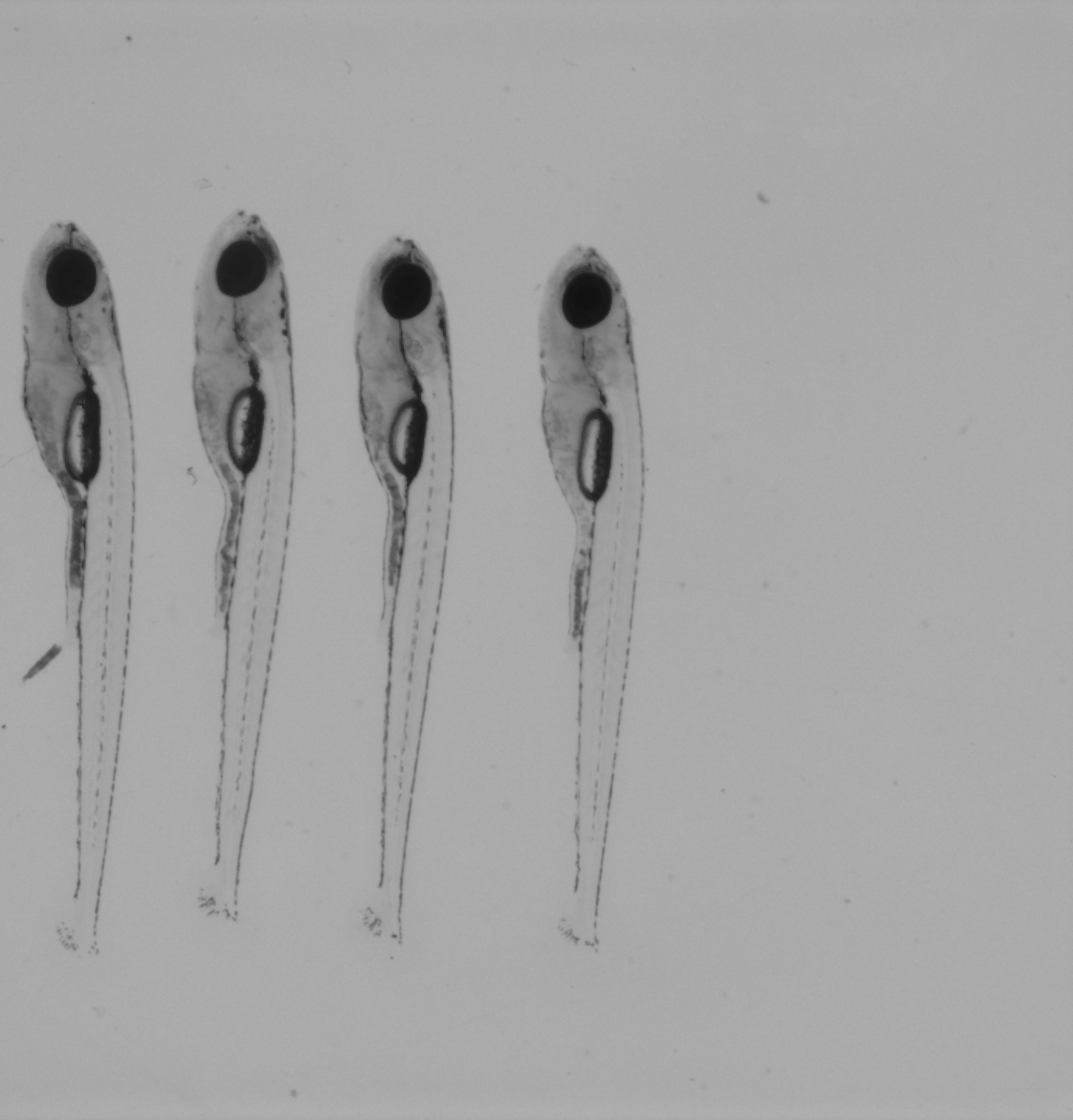

Supplement: Supplementary file 11 — Source data Fig. 2 [file 44318_2024_136_MOESM11_ESM.zip › Figure 2C-D/14 dpf-Standard length and Trunk surface area-Fish 2-5_SG.tif]

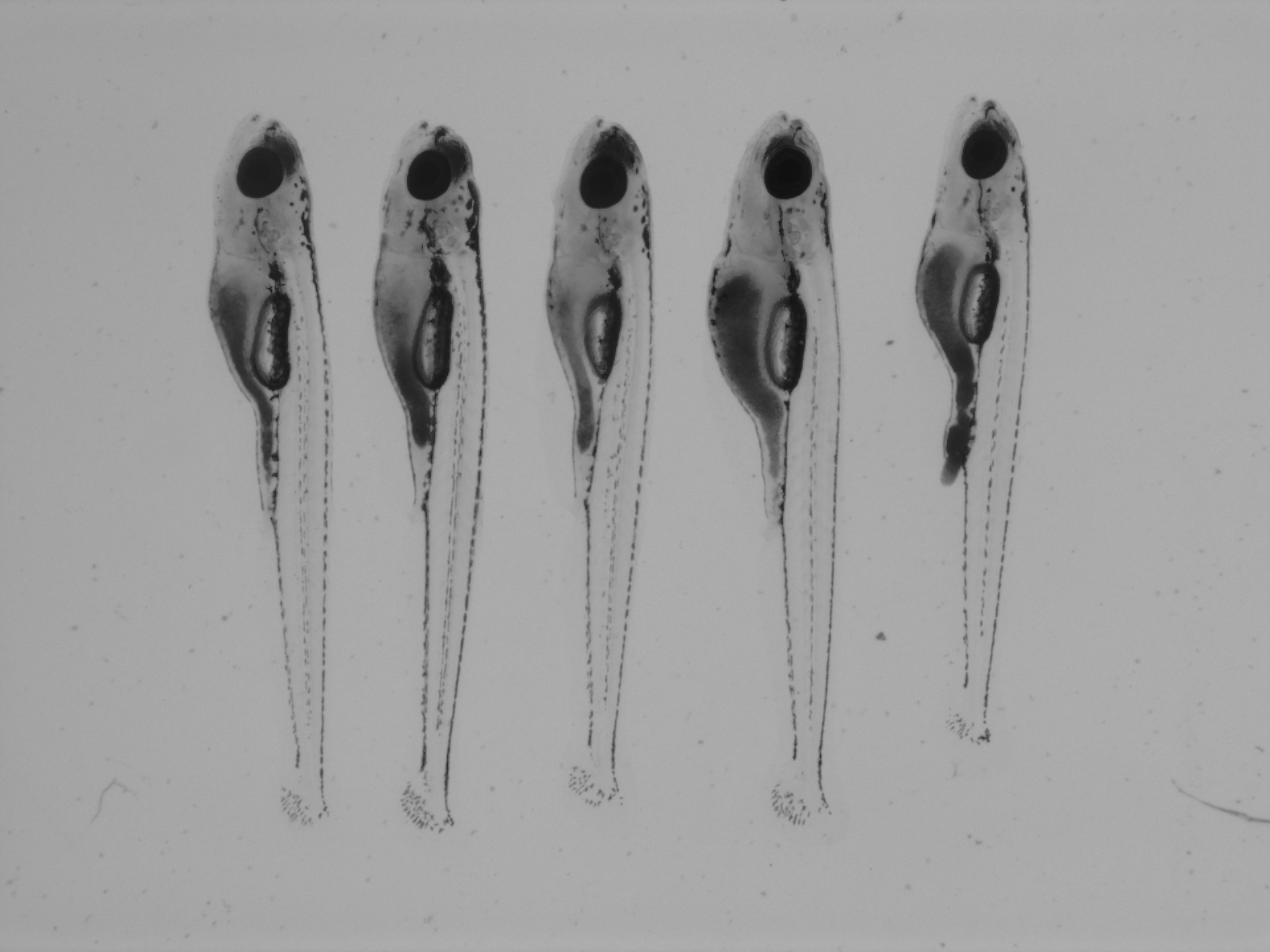

Supplement: Supplementary file 11 — Source data Fig. 2 [file 44318_2024_136_MOESM11_ESM.zip › Figure 2C-D/14 dpf-Standard length and Trunk surface area-Fish 6-10_FG.tif]

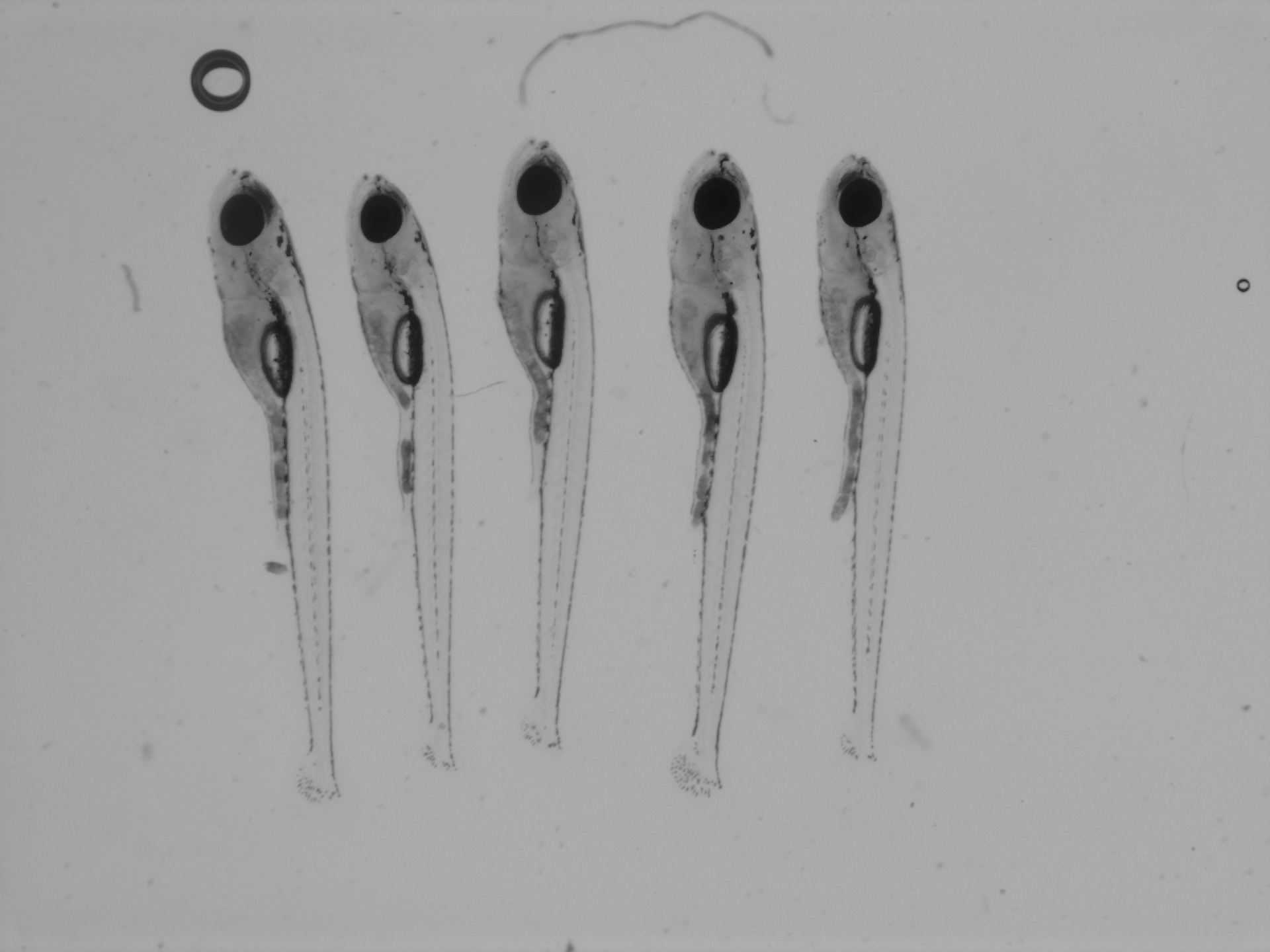

Supplement: Supplementary file 11 — Source data Fig. 2 [file 44318_2024_136_MOESM11_ESM.zip › Figure 2C-D/14 dpf-Standard length and Trunk surface area-Fish 6-10_SG.tif]

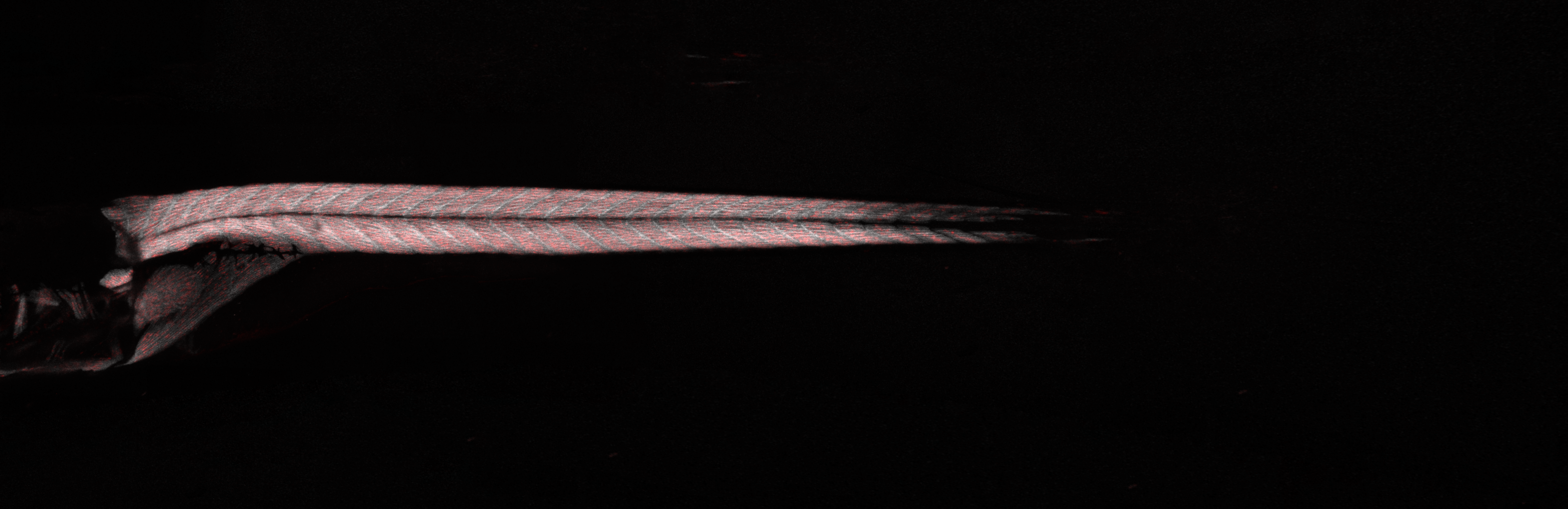

Supplement: Supplementary file 11 — Source data Fig. 2 [file 44318_2024_136_MOESM11_ESM.zip › Figure 2E/10 dpf-Fish 1-FG.tiff]

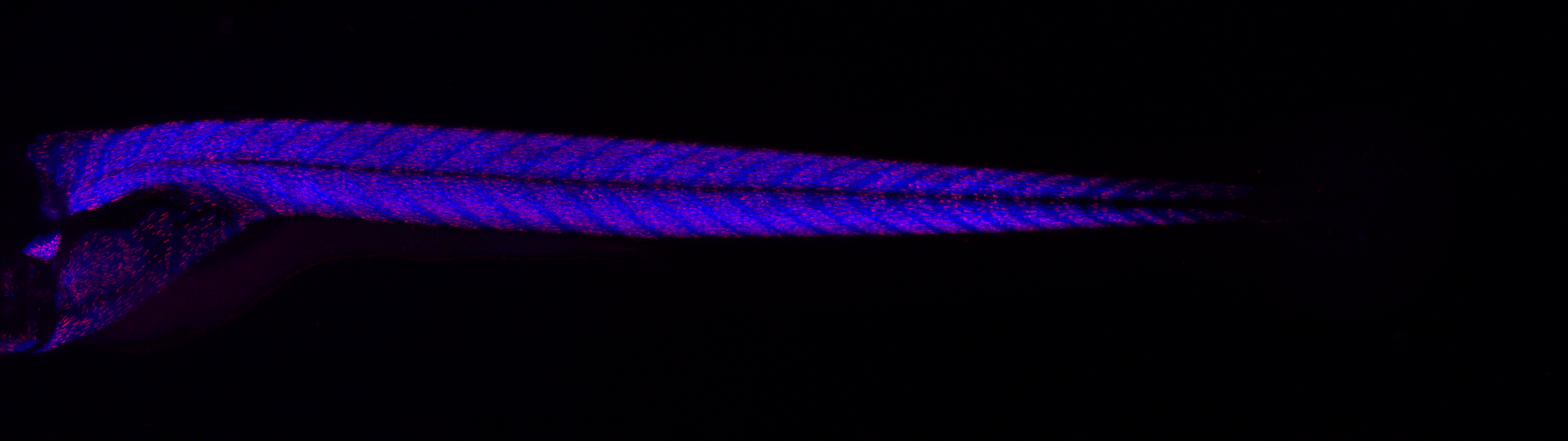

Supplement: Supplementary file 11 — Source data Fig. 2 [file 44318_2024_136_MOESM11_ESM.zip › Figure 2E/10 dpf-Fish 1-SG.tiff]

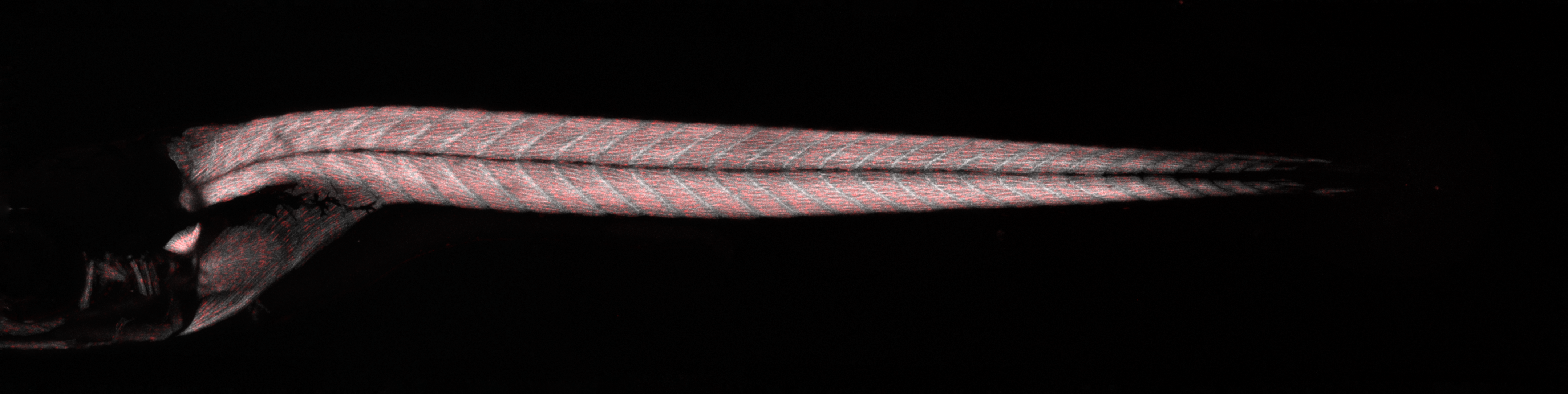

Supplement: Supplementary file 11 — Source data Fig. 2 [file 44318_2024_136_MOESM11_ESM.zip › Figure 2E/10 dpf-Fish 2-FG.tiff]

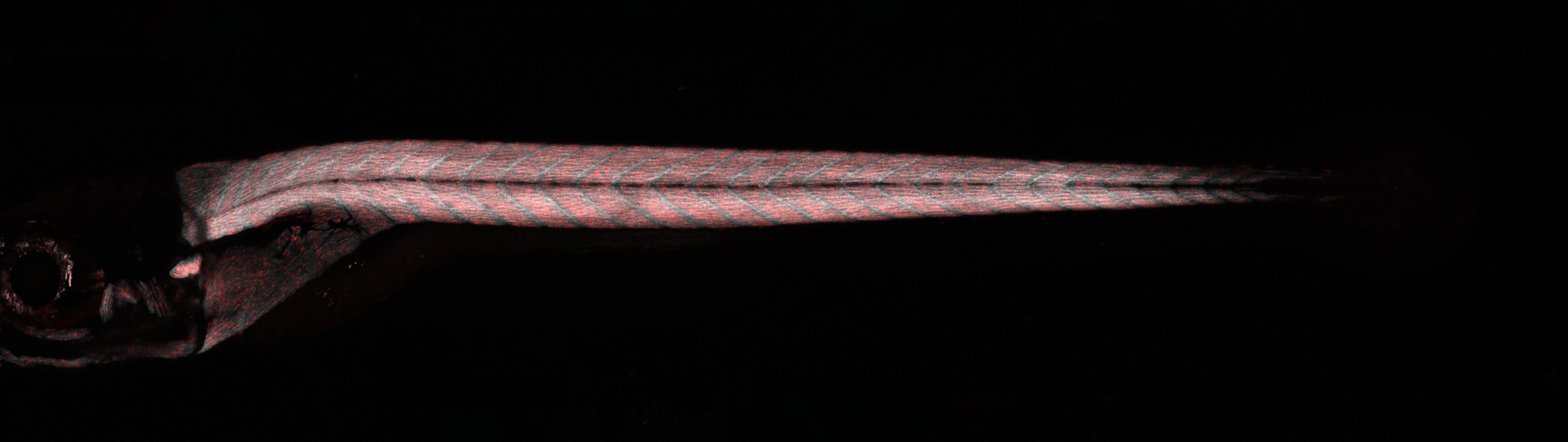

Supplement: Supplementary file 11 — Source data Fig. 2 [file 44318_2024_136_MOESM11_ESM.zip › Figure 2E/10 dpf-Fish 2-SG.tiff]

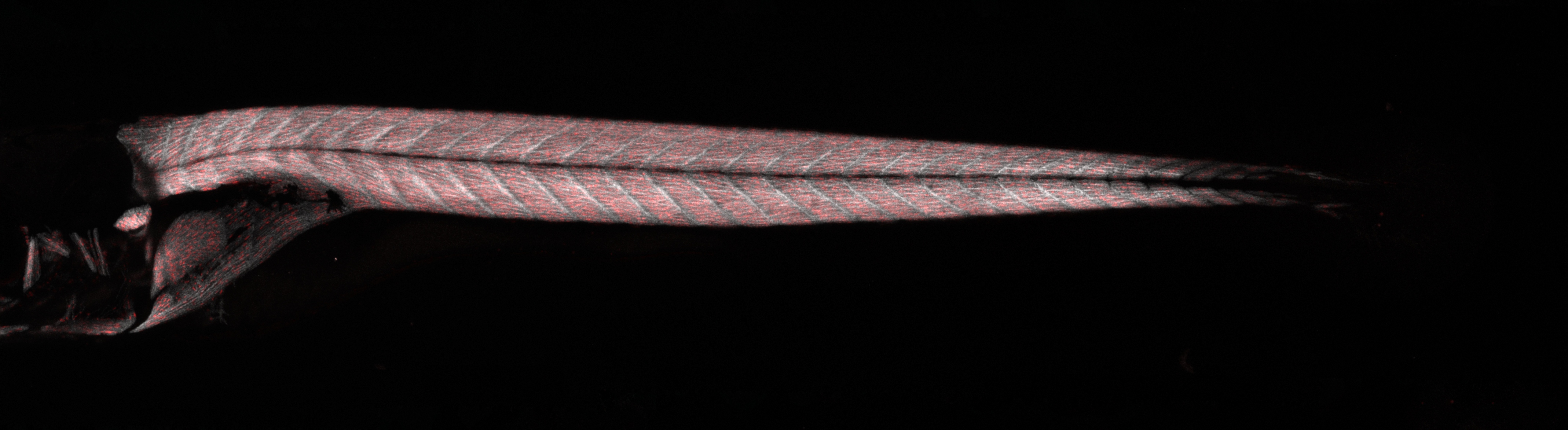

Supplement: Supplementary file 11 — Source data Fig. 2 [file 44318_2024_136_MOESM11_ESM.zip › Figure 2E/10 dpf-Fish 3-FG.tiff]

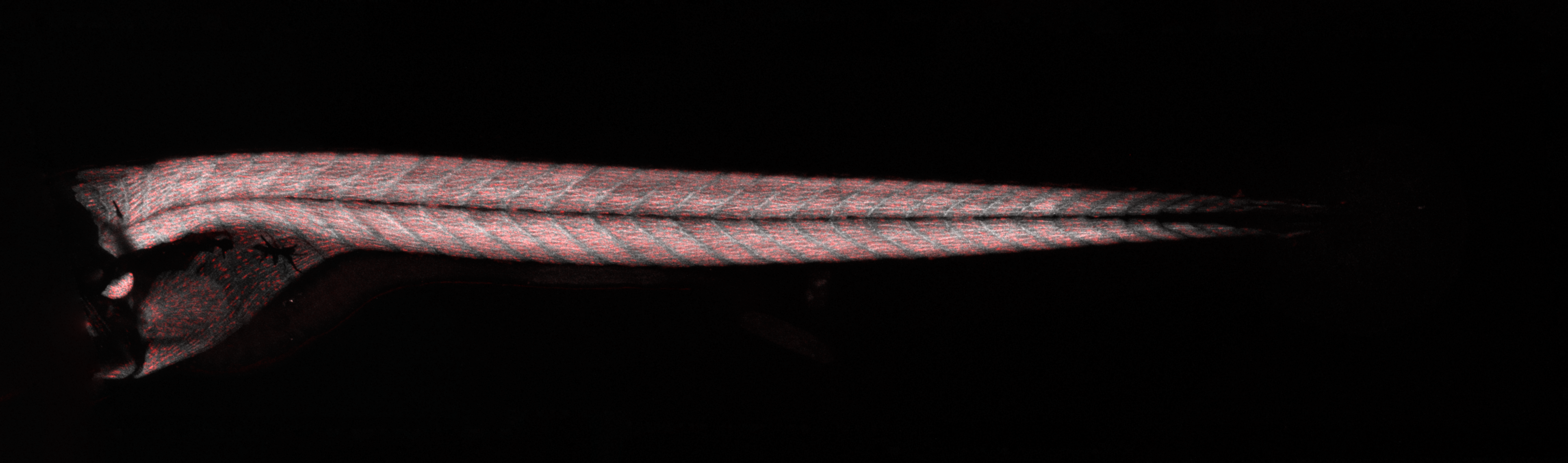

Supplement: Supplementary file 11 — Source data Fig. 2 [file 44318_2024_136_MOESM11_ESM.zip › Figure 2E/10 dpf-Fish 3-SG.tiff]

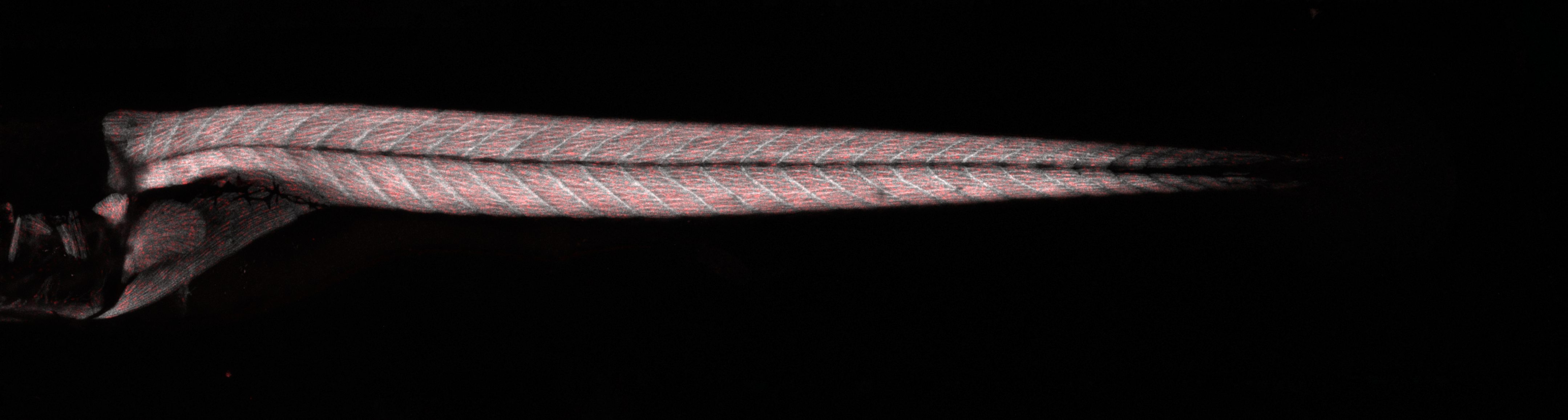

Supplement: Supplementary file 11 — Source data Fig. 2 [file 44318_2024_136_MOESM11_ESM.zip › Figure 2E/10 dpf-Fish 4-FG.tiff]

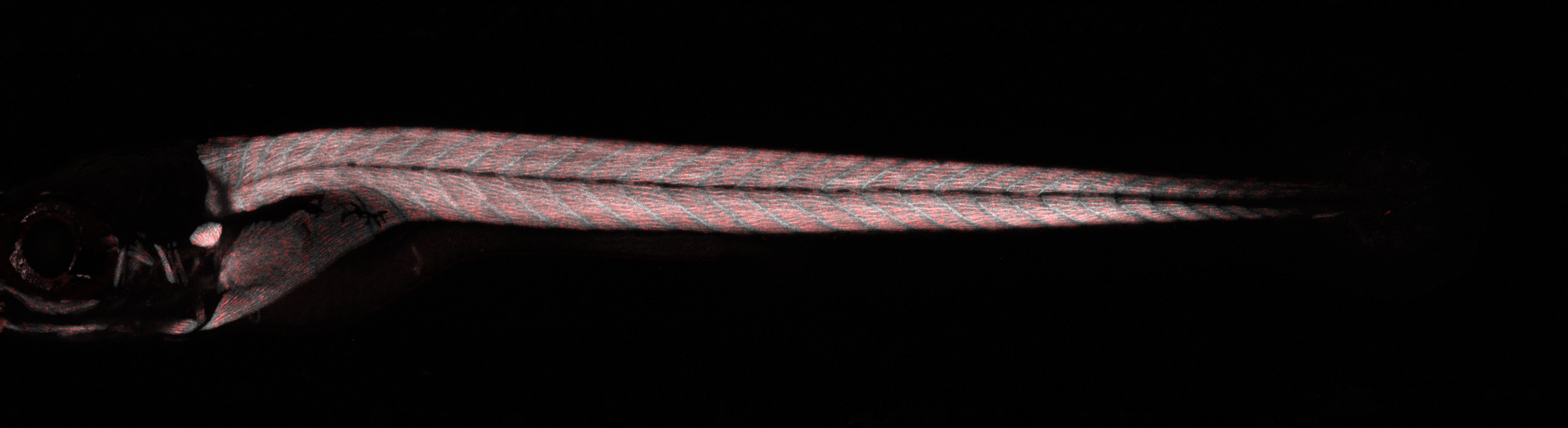

Supplement: Supplementary file 11 — Source data Fig. 2 [file 44318_2024_136_MOESM11_ESM.zip › Figure 2E/10 dpf-Fish 4-SG.tiff]

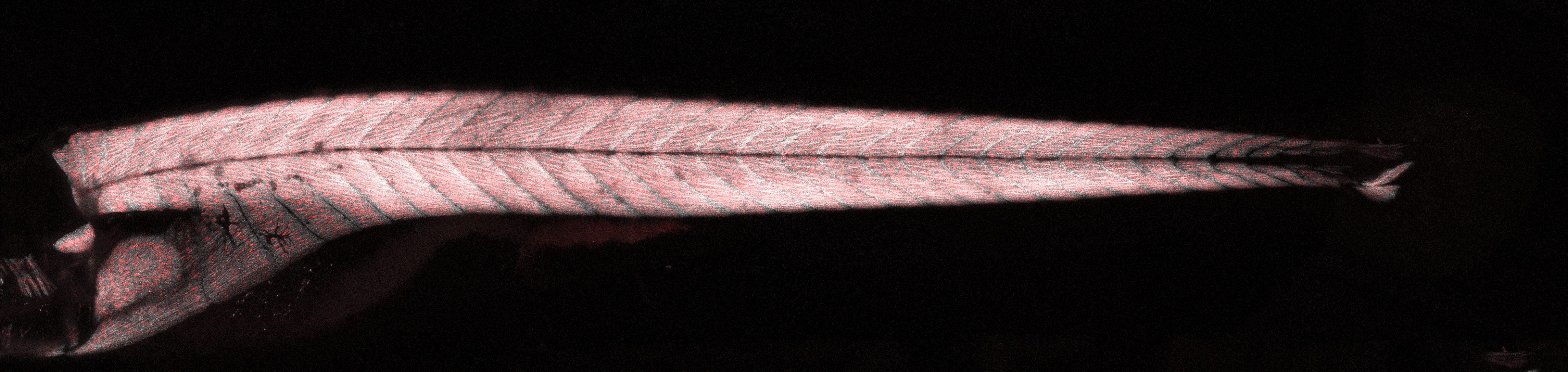

Supplement: Supplementary file 11 — Source data Fig. 2 [file 44318_2024_136_MOESM11_ESM.zip › Figure 2E/14 dpf-Fish 1-FG.tif]

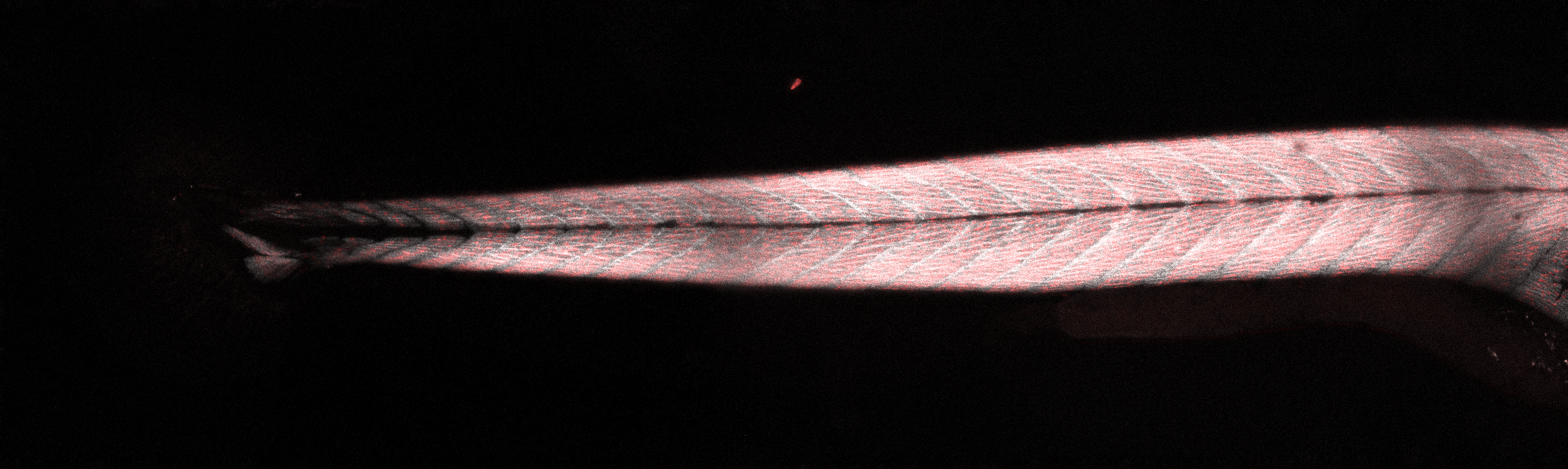

Supplement: Supplementary file 11 — Source data Fig. 2 [file 44318_2024_136_MOESM11_ESM.zip › Figure 2E/14 dpf-Fish 1-SG.tif]

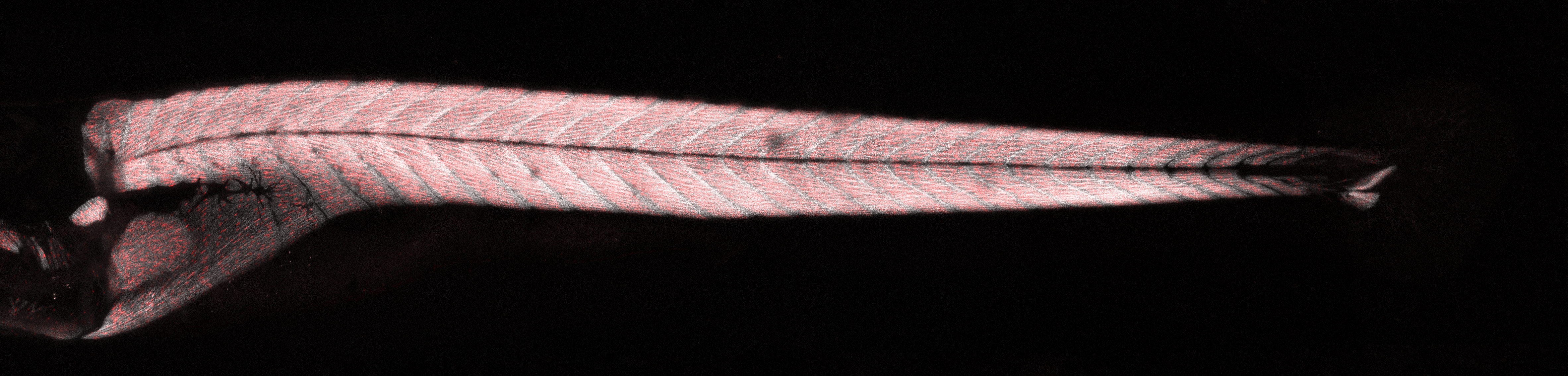

Supplement: Supplementary file 11 — Source data Fig. 2 [file 44318_2024_136_MOESM11_ESM.zip › Figure 2E/14 dpf-Fish 2-FG.tif]

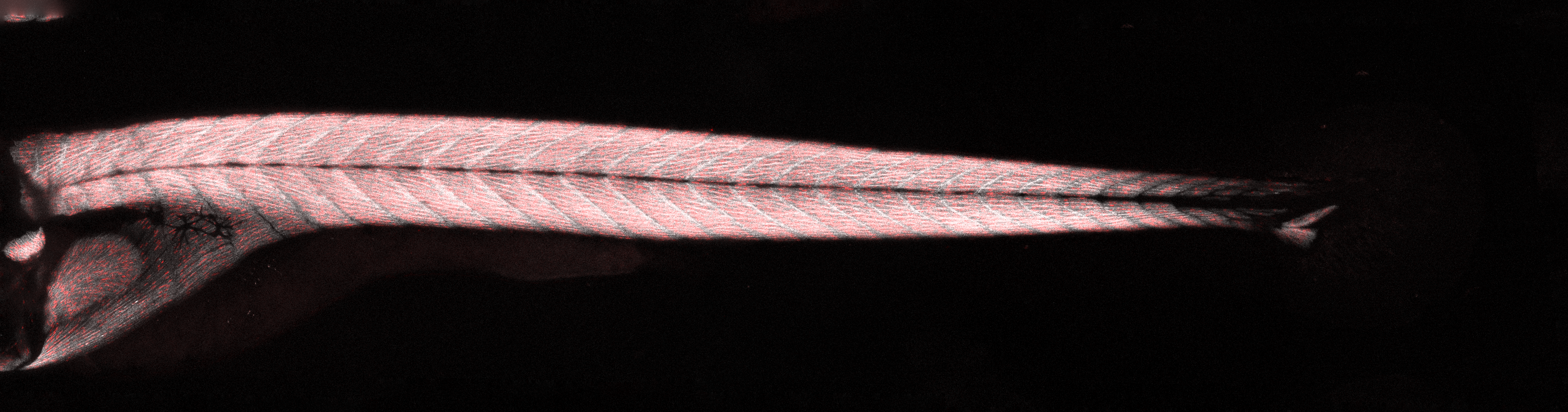

Supplement: Supplementary file 11 — Source data Fig. 2 [file 44318_2024_136_MOESM11_ESM.zip › Figure 2E/14 dpf-Fish 2-SG.tif]

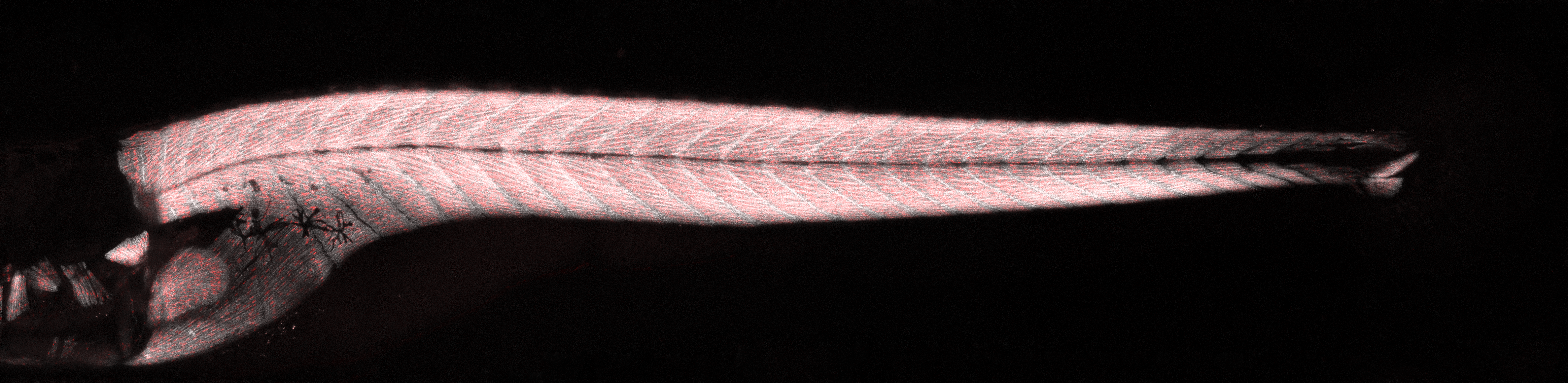

Supplement: Supplementary file 11 — Source data Fig. 2 [file 44318_2024_136_MOESM11_ESM.zip › Figure 2E/14 dpf-Fish 3-FG.tif]

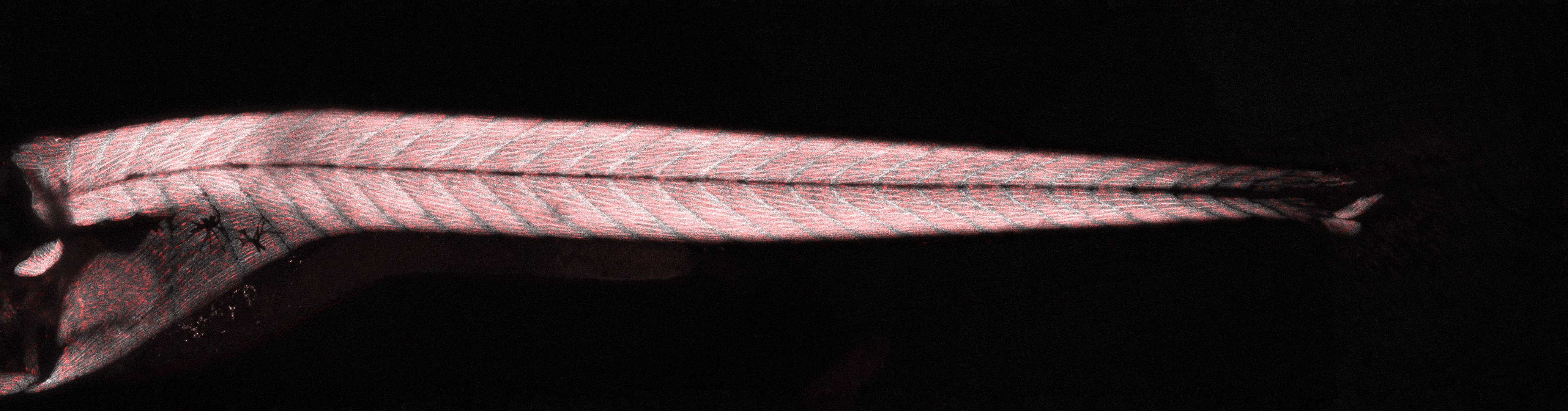

Supplement: Supplementary file 11 — Source data Fig. 2 [file 44318_2024_136_MOESM11_ESM.zip › Figure 2E/14 dpf-Fish 3-SG.tif]

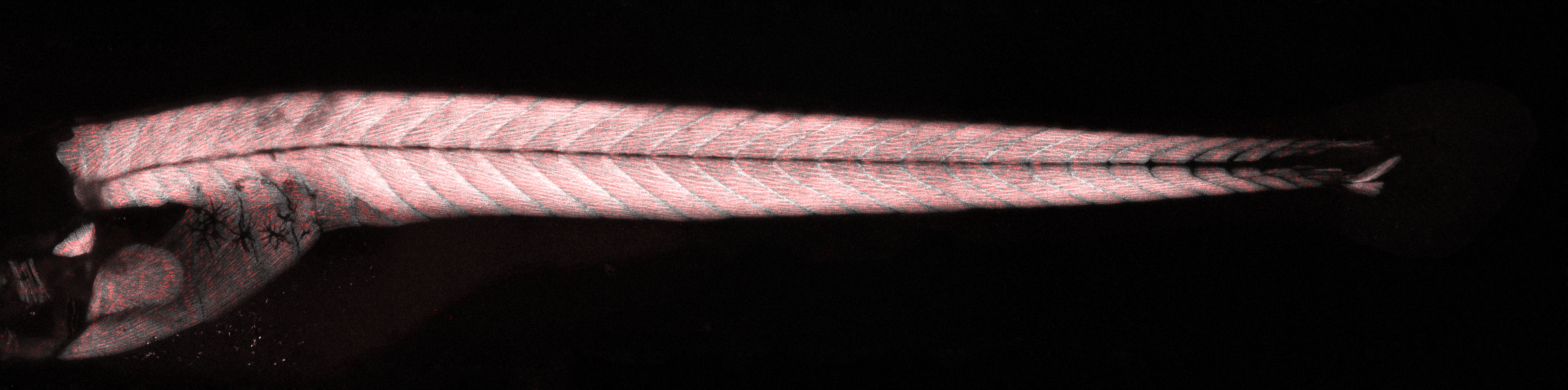

Supplement: Supplementary file 11 — Source data Fig. 2 [file 44318_2024_136_MOESM11_ESM.zip › Figure 2E/14 dpf-Fish 4-FG.tif]

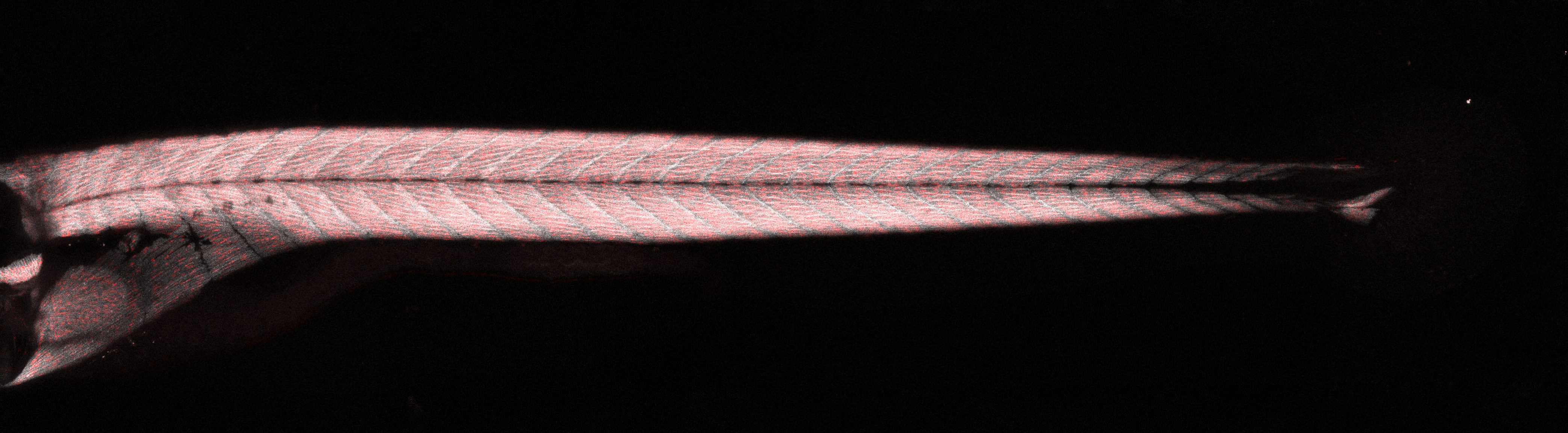

Supplement: Supplementary file 11 — Source data Fig. 2 [file 44318_2024_136_MOESM11_ESM.zip › Figure 2E/14 dpf-Fish 4-SG.tif]

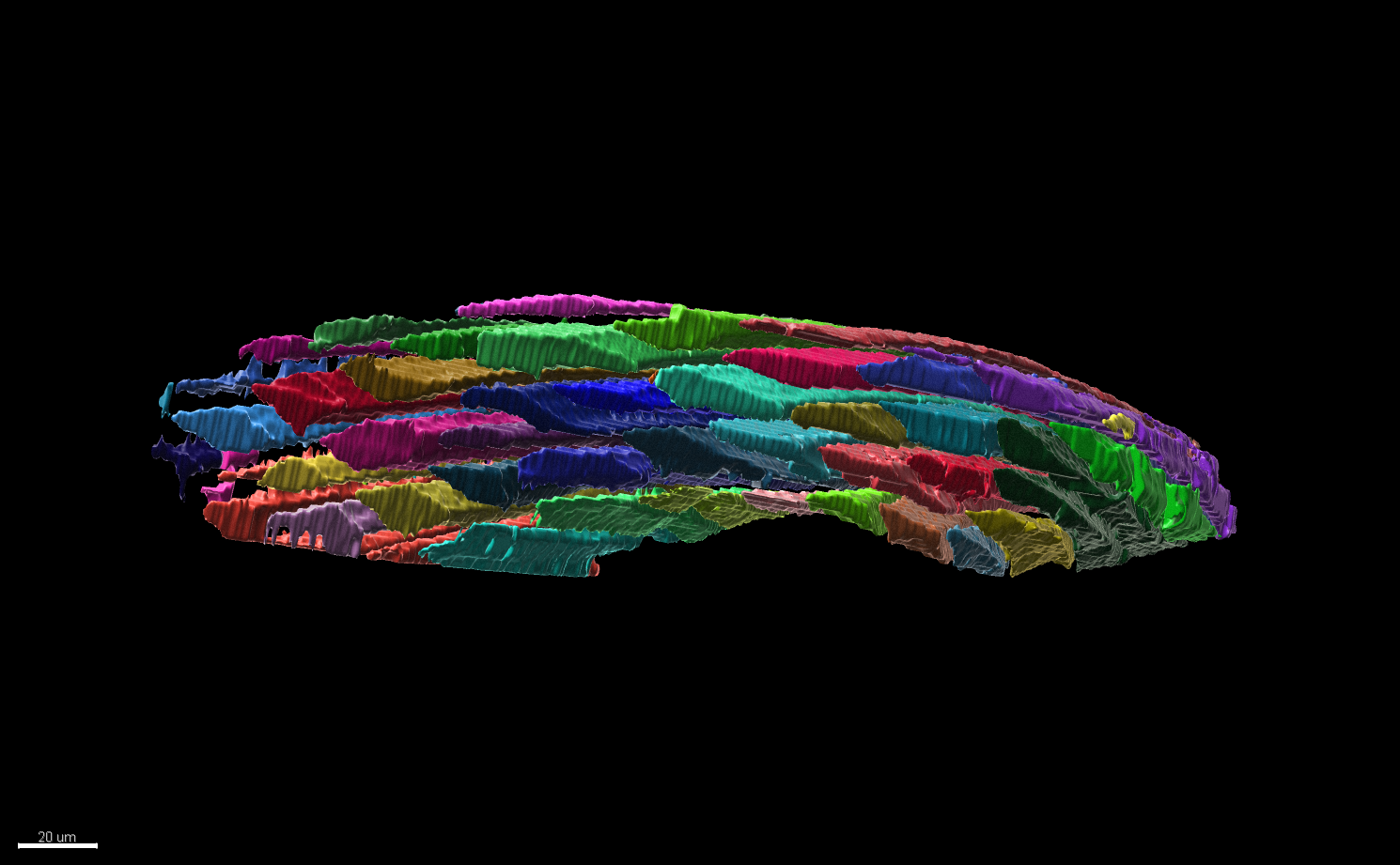

Supplement: Supplementary file 11 — Source data Fig. 2 [file 44318_2024_136_MOESM11_ESM.zip › Figure 2H/Dorsal myotome-3D image.tif]

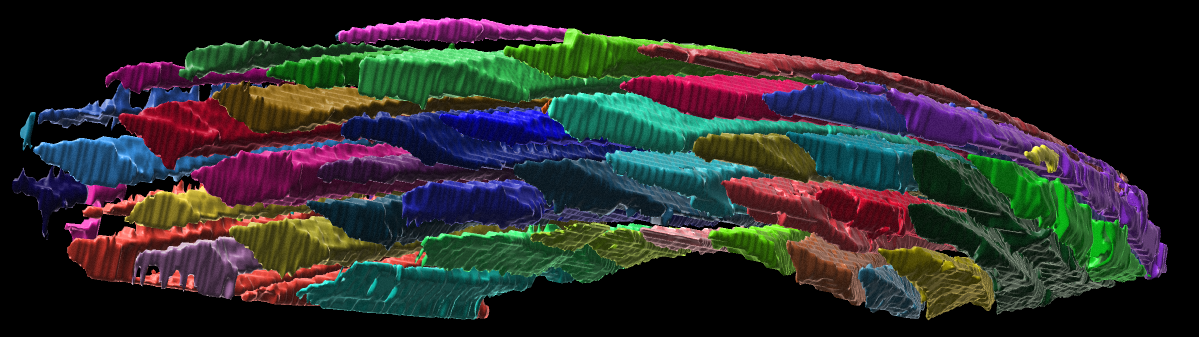

Supplement: Supplementary file 11 — Source data Fig. 2 [file 44318_2024_136_MOESM11_ESM.zip › Figure 2H/Dorsal myotome-3D image-crop.tif]

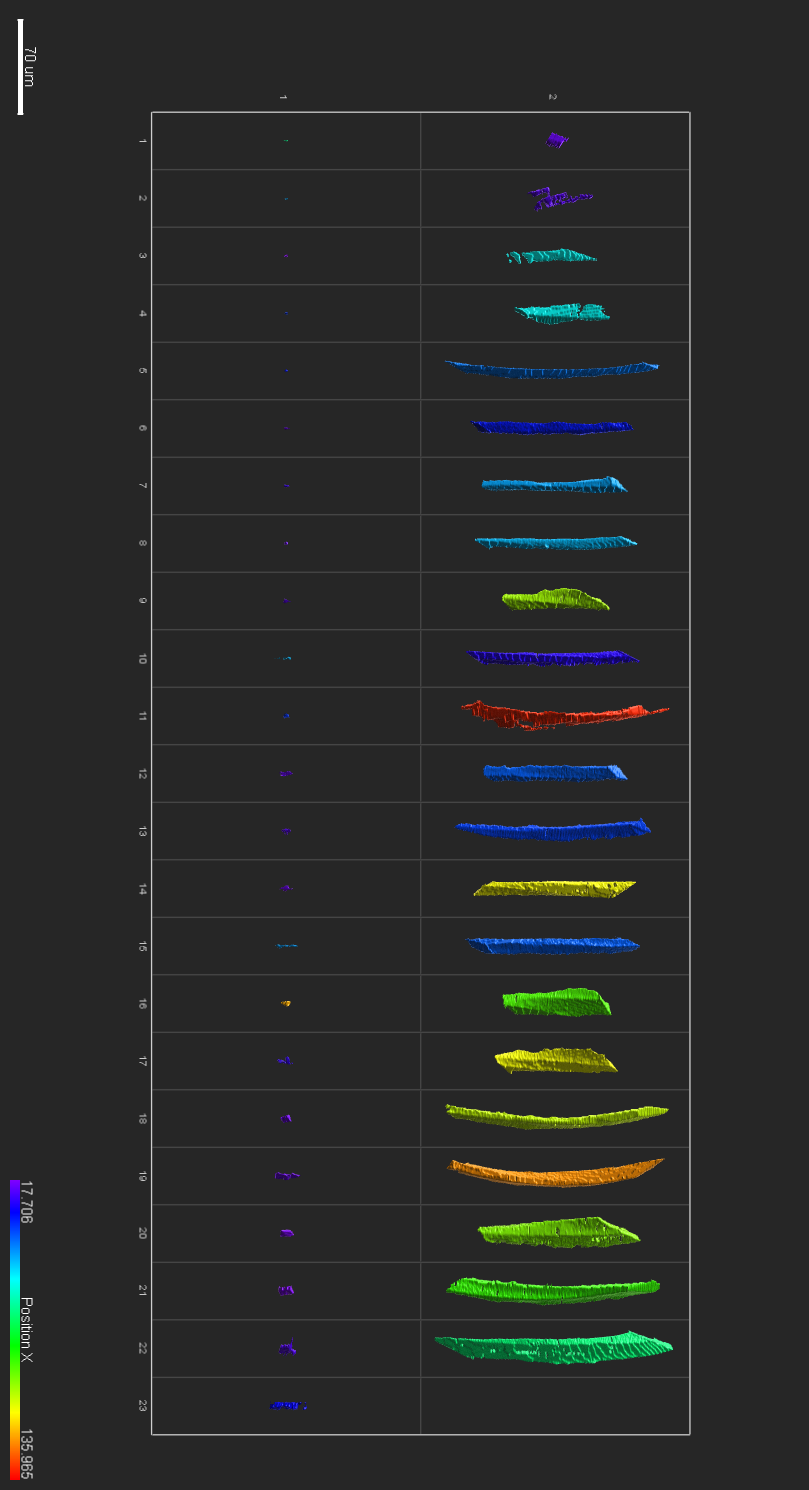

Supplement: Supplementary file 11 — Source data Fig. 2 [file 44318_2024_136_MOESM11_ESM.zip › Figure 2H/Myofiber vintage-3D image.tif]

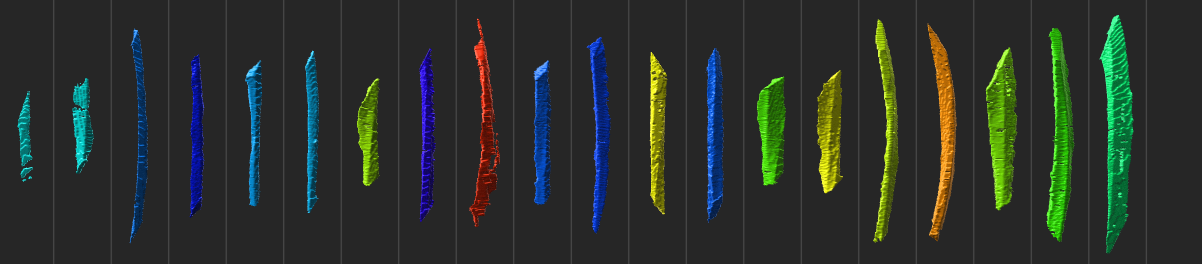

Supplement: Supplementary file 11 — Source data Fig. 2 [file 44318_2024_136_MOESM11_ESM.zip › Figure 2H/Myofiber vintage-3D image-crop.tif]

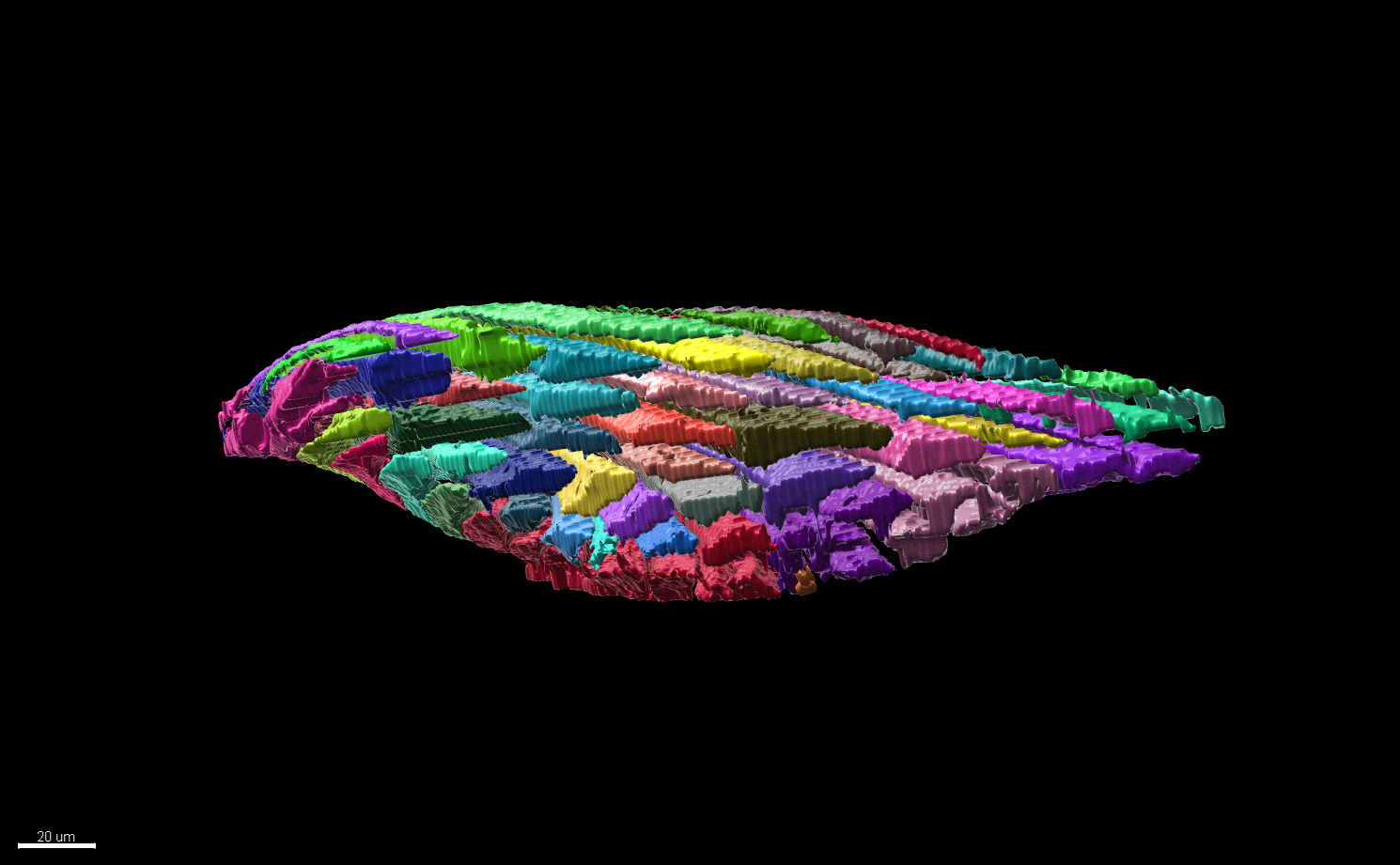

Supplement: Supplementary file 11 — Source data Fig. 2 [file 44318_2024_136_MOESM11_ESM.zip › Figure 2H/Ventral myotome-3D image.tif]

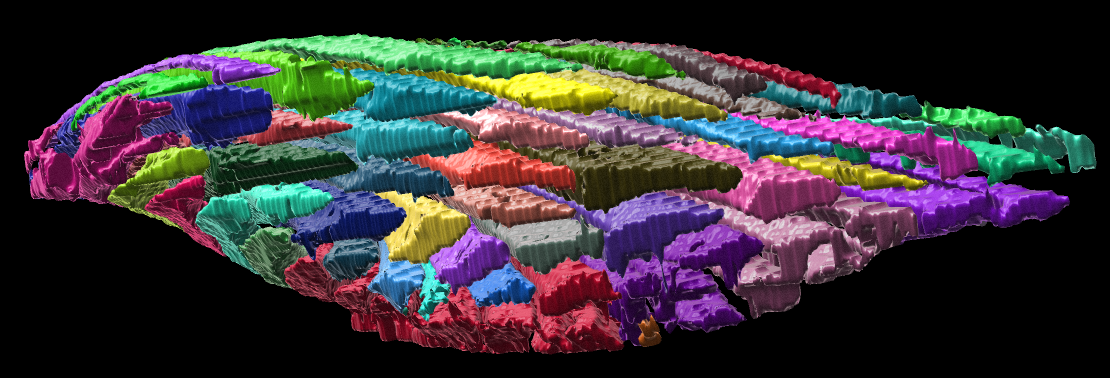

Supplement: Supplementary file 11 — Source data Fig. 2 [file 44318_2024_136_MOESM11_ESM.zip › Figure 2H/Ventral myotome-3D image-crop.tif]

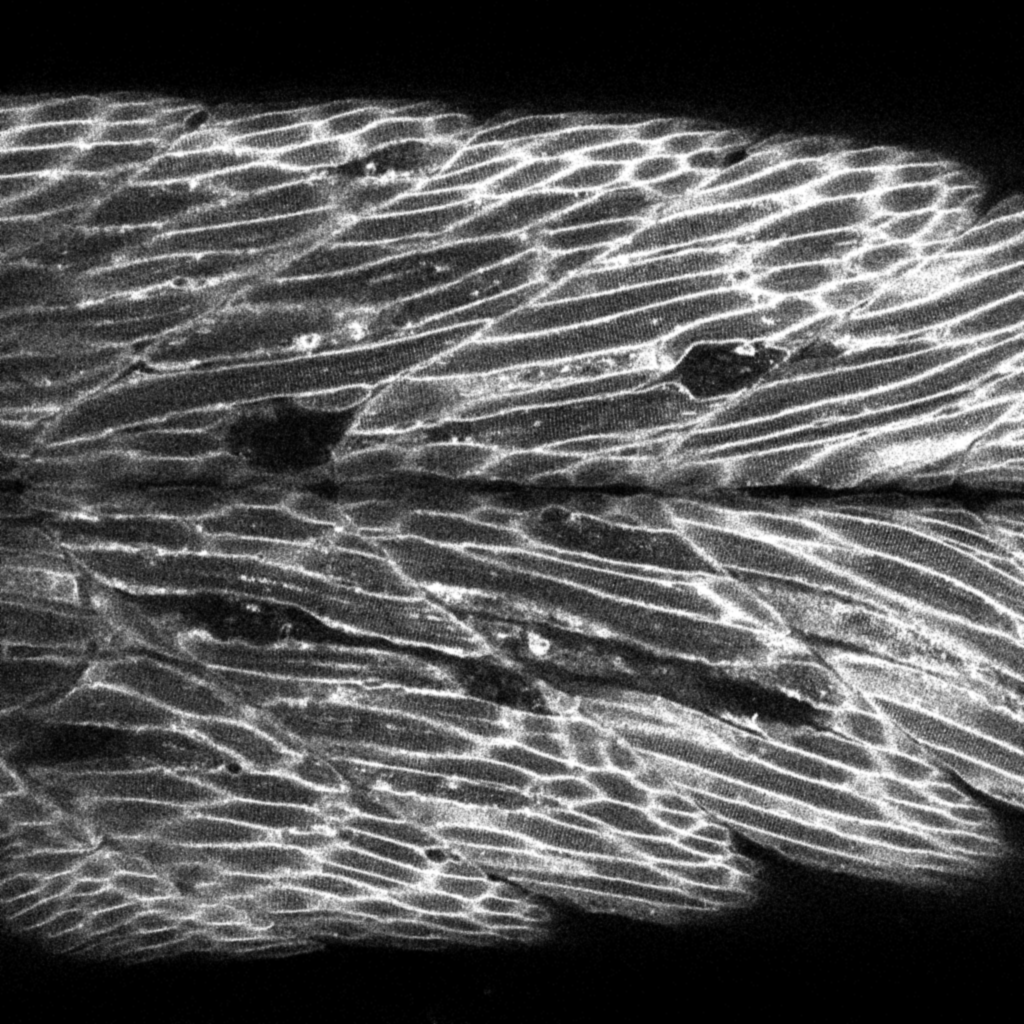

Supplement: Supplementary file 12 — Source data Fig. 3 [file 44318_2024_136_MOESM12_ESM.zip › Figure 3A/Transverse view-trunk myofiber with deformation-single z.tif]

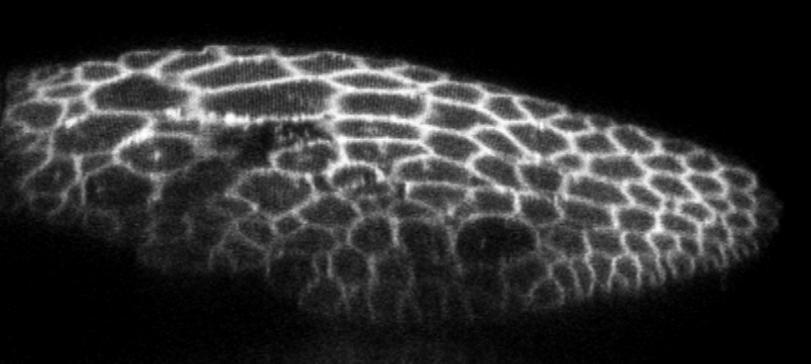

Supplement: Supplementary file 12 — Source data Fig. 3 [file 44318_2024_136_MOESM12_ESM.zip › Figure 3B/Cross-sectional view-trunk myofiber- deformed.tif]

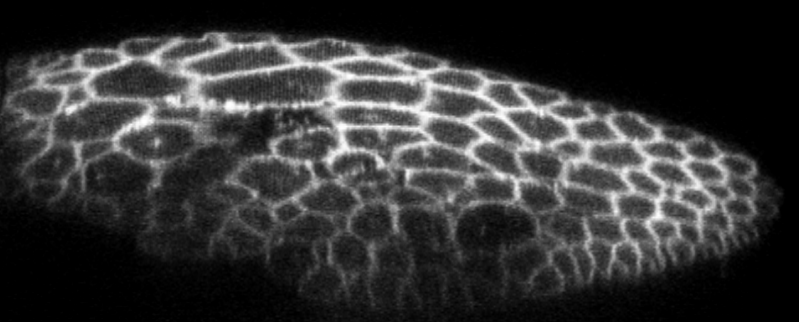

Supplement: Supplementary file 12 — Source data Fig. 3 [file 44318_2024_136_MOESM12_ESM.zip › Figure 3B/Cross-sectional view-trunk myofiber- defprmed-crop.tif]

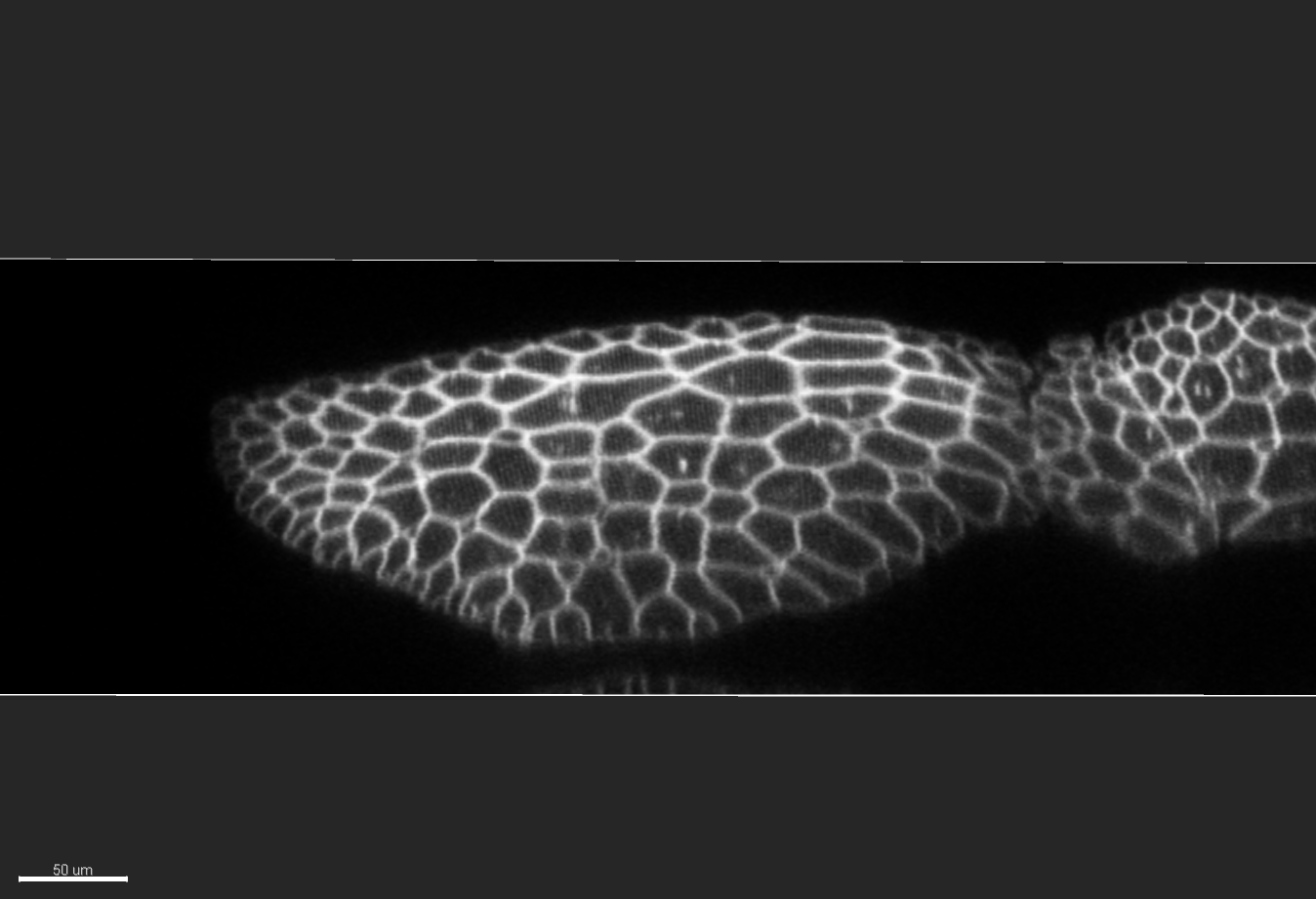

Supplement: Supplementary file 12 — Source data Fig. 3 [file 44318_2024_136_MOESM12_ESM.zip › Figure 3B/Cross-sectional view-trunk myofiber-normal.tif]

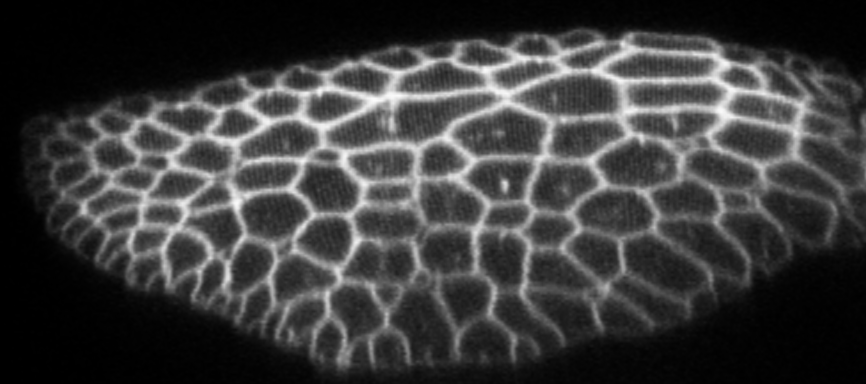

Supplement: Supplementary file 12 — Source data Fig. 3 [file 44318_2024_136_MOESM12_ESM.zip › Figure 3B/Cross-sectional view-trunk myofiber-normal-crop.tif]

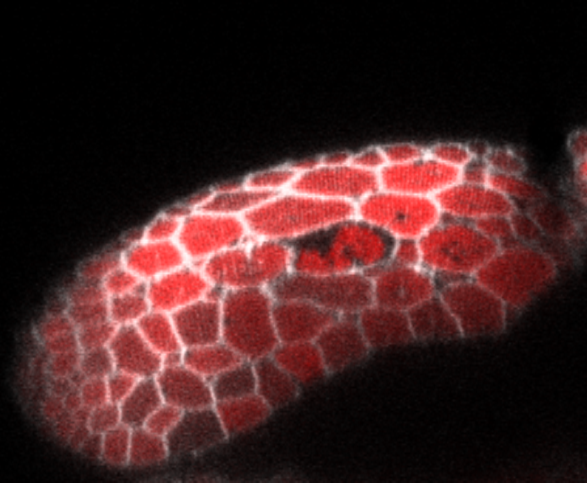

Supplement: Supplementary file 12 — Source data Fig. 3 [file 44318_2024_136_MOESM12_ESM.zip › Figure 3C/palmuscle-F-actin-cross-sectional view-deform.tif]

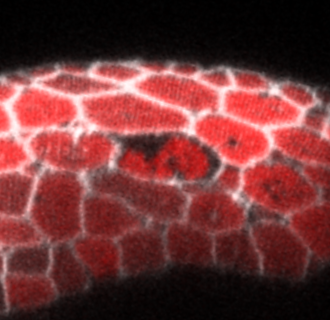

Supplement: Supplementary file 12 — Source data Fig. 3 [file 44318_2024_136_MOESM12_ESM.zip › Figure 3C/palmuscle-F-actin-cross-sectional view-deform-crop.tif]

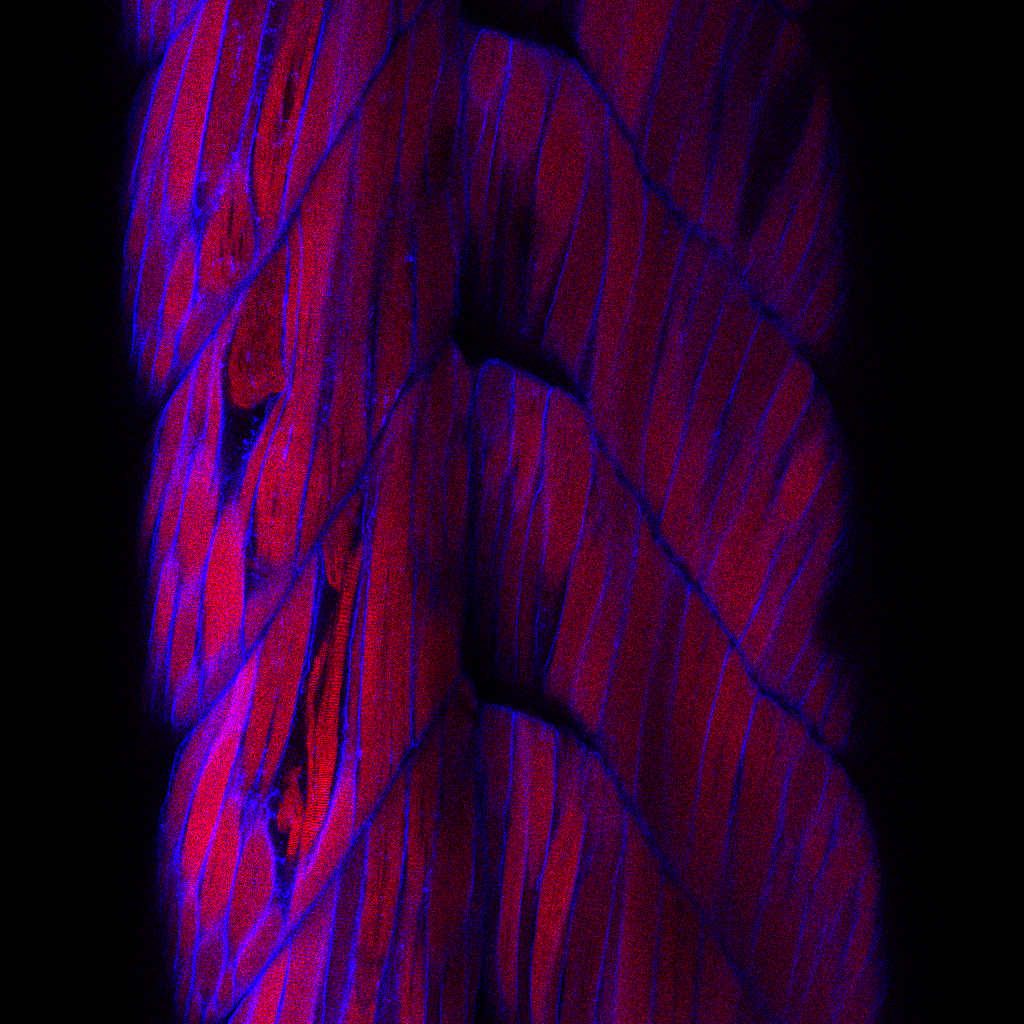

Supplement: Supplementary file 12 — Source data Fig. 3 [file 44318_2024_136_MOESM12_ESM.zip › Figure 3C/palmuscle-F-actin-transverse view-deform.tif]

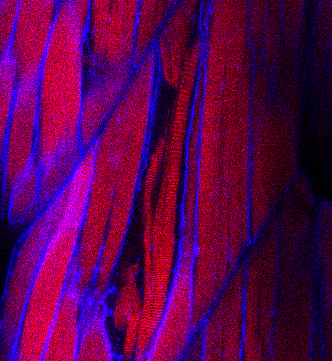

Supplement: Supplementary file 12 — Source data Fig. 3 [file 44318_2024_136_MOESM12_ESM.zip › Figure 3C/palmuscle-F-actin-transverse view-deform-crop.tif]

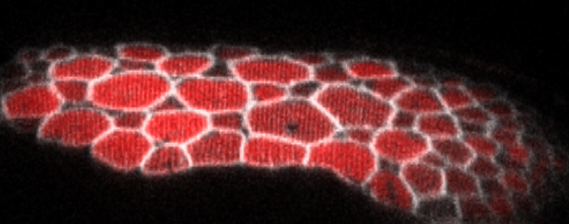

Supplement: Supplementary file 12 — Source data Fig. 3 [file 44318_2024_136_MOESM12_ESM.zip › Figure 3D/palmuscle-F-actin-13 dpf.tif]

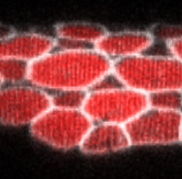

Supplement: Supplementary file 12 — Source data Fig. 3 [file 44318_2024_136_MOESM12_ESM.zip › Figure 3D/palmuscle-F-actin-13 dpf-crop.tif]

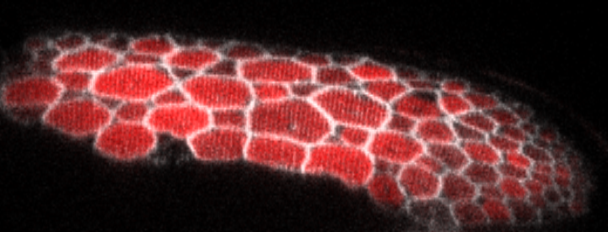

Supplement: Supplementary file 12 — Source data Fig. 3 [file 44318_2024_136_MOESM12_ESM.zip › Figure 3D/palmuscle-F-actin-14 dpf.tif]

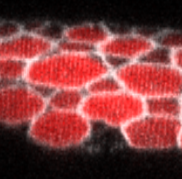

Supplement: Supplementary file 12 — Source data Fig. 3 [file 44318_2024_136_MOESM12_ESM.zip › Figure 3D/palmuscle-F-actin-14 dpf-crop.tif]

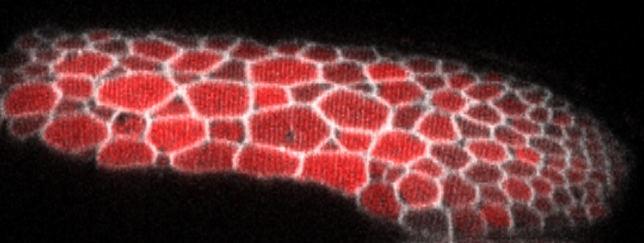

Supplement: Supplementary file 12 — Source data Fig. 3 [file 44318_2024_136_MOESM12_ESM.zip › Figure 3D/palmuscle-F-actin-15 dpf.tif]

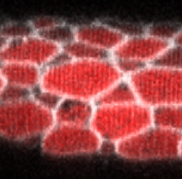

Supplement: Supplementary file 12 — Source data Fig. 3 [file 44318_2024_136_MOESM12_ESM.zip › Figure 3D/palmuscle-F-actin-15 dpf-crop.tif]

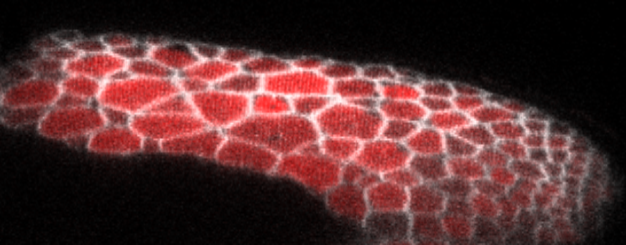

Supplement: Supplementary file 12 — Source data Fig. 3 [file 44318_2024_136_MOESM12_ESM.zip › Figure 3D/palmuscle-F-actin-16 dpf.tif]

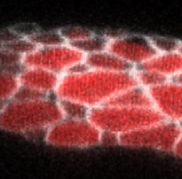

Supplement: Supplementary file 12 — Source data Fig. 3 [file 44318_2024_136_MOESM12_ESM.zip › Figure 3D/palmuscle-F-actin-16 dpf-crop.tif]

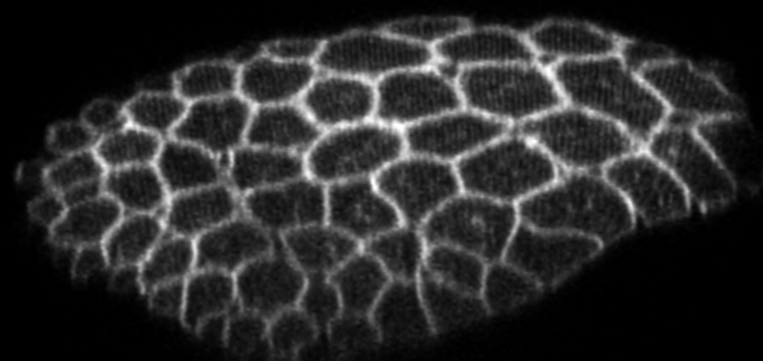

Supplement: Supplementary file 12 — Source data Fig. 3 [file 44318_2024_136_MOESM12_ESM.zip › Figure 3F/Cross-sectional view-10 dpf.tif]

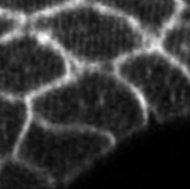

Supplement: Supplementary file 12 — Source data Fig. 3 [file 44318_2024_136_MOESM12_ESM.zip › Figure 3F/Cross-sectional view-10 dpf-crop.tif]

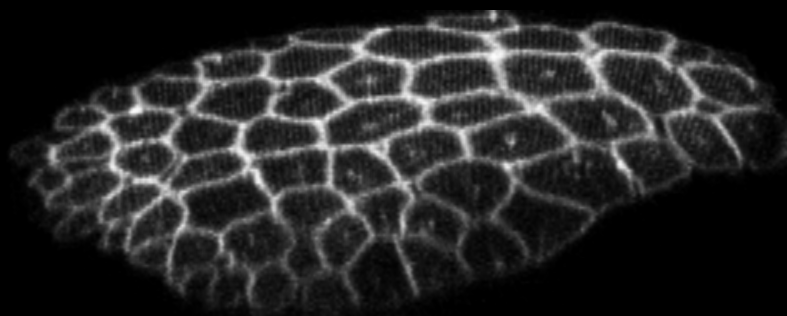

Supplement: Supplementary file 12 — Source data Fig. 3 [file 44318_2024_136_MOESM12_ESM.zip › Figure 3F/Cross-sectional view-10.5 dpf.tif]
