# Supplementary figures and images for: Whole-body replacement of larval myofibers generates permanent adult myofibers in zebrafish (part 2 of 4)
Source: EMBO J. 2024 Jun 5;43(15):2. doi: 10.1038/s44318-024-00136-y (PMC11294464; doi:10.1038/s44318-024-00136-y)

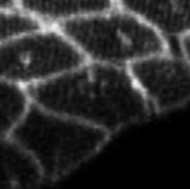

Supplement: Supplementary file 12 — Source data Fig. 3 [file 44318_2024_136_MOESM12_ESM.zip › Figure 3F/Cross-sectional view-10.5 dpf-crop.tif]

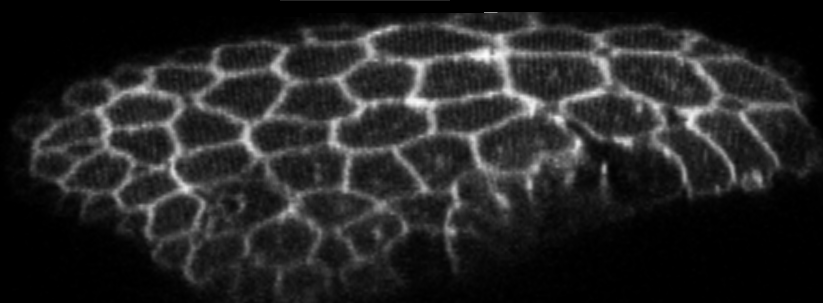

Supplement: Supplementary file 12 — Source data Fig. 3 [file 44318_2024_136_MOESM12_ESM.zip › Figure 3F/Cross-sectional view-11 dpf.tif]

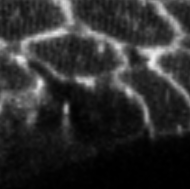

Supplement: Supplementary file 12 — Source data Fig. 3 [file 44318_2024_136_MOESM12_ESM.zip › Figure 3F/Cross-sectional view-11 dpf-crop.tif]

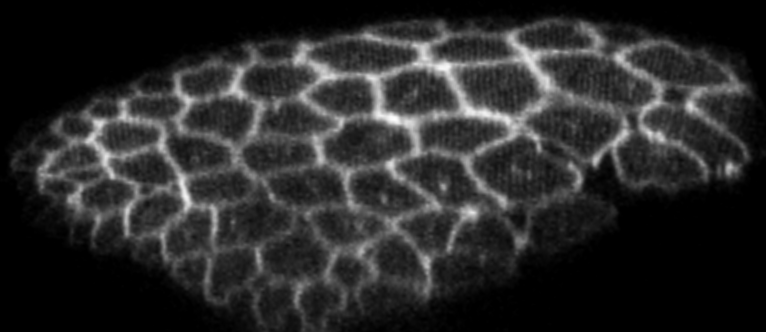

Supplement: Supplementary file 12 — Source data Fig. 3 [file 44318_2024_136_MOESM12_ESM.zip › Figure 3F/Cross-sectional view-11.5 dpf.tif]

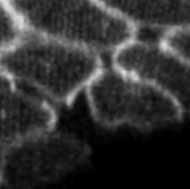

Supplement: Supplementary file 12 — Source data Fig. 3 [file 44318_2024_136_MOESM12_ESM.zip › Figure 3F/Cross-sectional view-11.5 dpf-crop.tif]

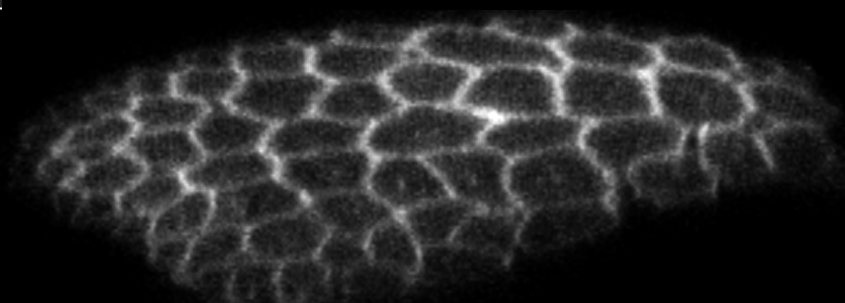

Supplement: Supplementary file 12 — Source data Fig. 3 [file 44318_2024_136_MOESM12_ESM.zip › Figure 3F/Cross-sectional view-12 dpf.tif]

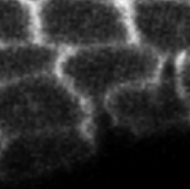

Supplement: Supplementary file 12 — Source data Fig. 3 [file 44318_2024_136_MOESM12_ESM.zip › Figure 3F/Cross-sectional view-12 dpf-crop.tif]

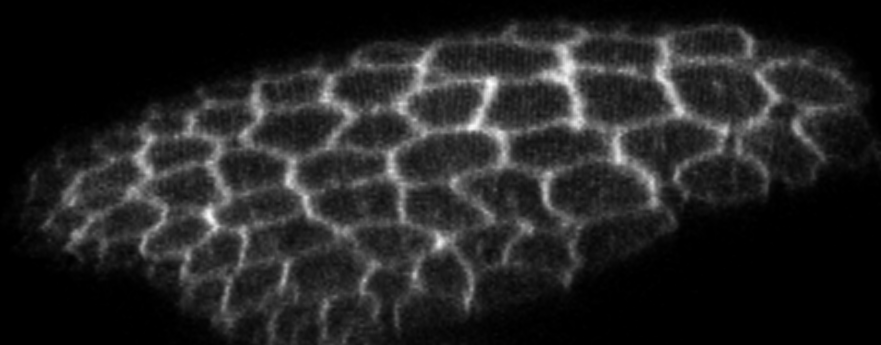

Supplement: Supplementary file 12 — Source data Fig. 3 [file 44318_2024_136_MOESM12_ESM.zip › Figure 3F/Cross-sectional view-12.5 dpf.tif]

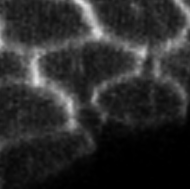

Supplement: Supplementary file 12 — Source data Fig. 3 [file 44318_2024_136_MOESM12_ESM.zip › Figure 3F/Cross-sectional view-12.5 dpf-crop.tif]

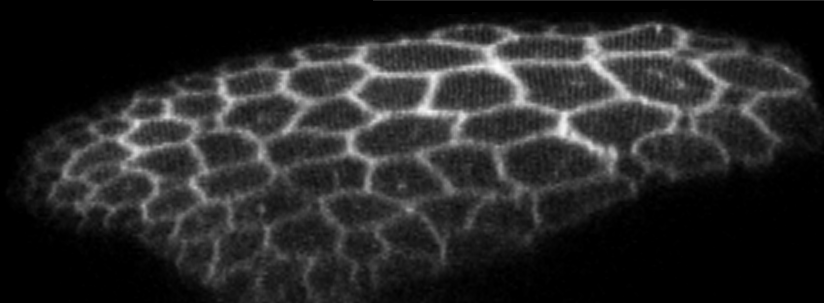

Supplement: Supplementary file 12 — Source data Fig. 3 [file 44318_2024_136_MOESM12_ESM.zip › Figure 3F/Cross-sectional view-13 dpf.tif]

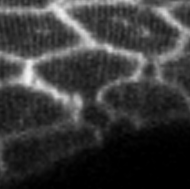

Supplement: Supplementary file 12 — Source data Fig. 3 [file 44318_2024_136_MOESM12_ESM.zip › Figure 3F/Cross-sectional view-13 dpf-crop.tif]

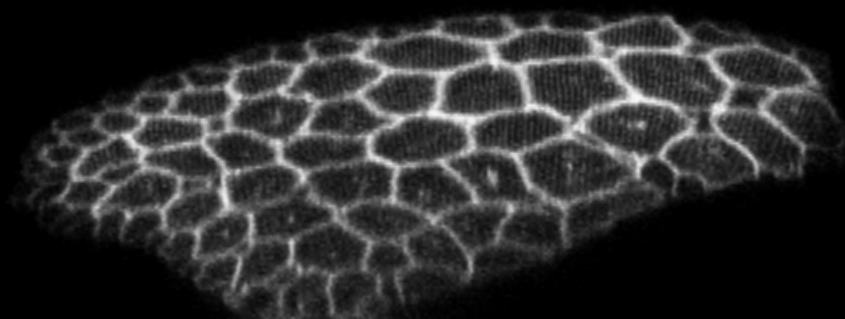

Supplement: Supplementary file 12 — Source data Fig. 3 [file 44318_2024_136_MOESM12_ESM.zip › Figure 3F/Cross-sectional view-13.5 dpf.tif]

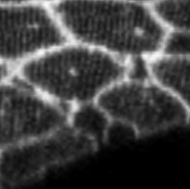

Supplement: Supplementary file 12 — Source data Fig. 3 [file 44318_2024_136_MOESM12_ESM.zip › Figure 3F/Cross-sectional view-13.5 dpf-crop.tif]

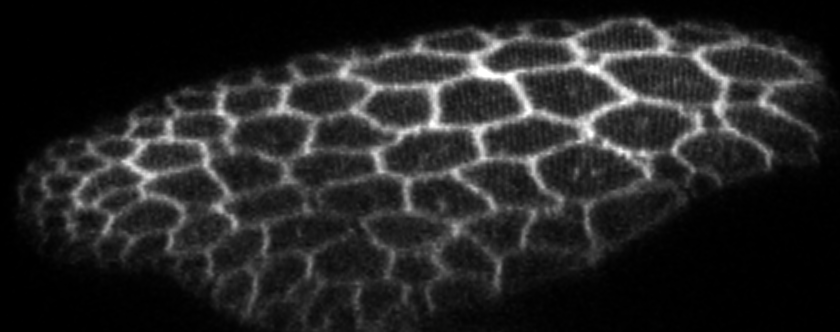

Supplement: Supplementary file 12 — Source data Fig. 3 [file 44318_2024_136_MOESM12_ESM.zip › Figure 3F/Cross-sectional view-14 dpf.tif]

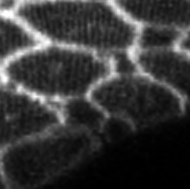

Supplement: Supplementary file 12 — Source data Fig. 3 [file 44318_2024_136_MOESM12_ESM.zip › Figure 3F/Cross-sectional view-14 dpf-crop.tif]

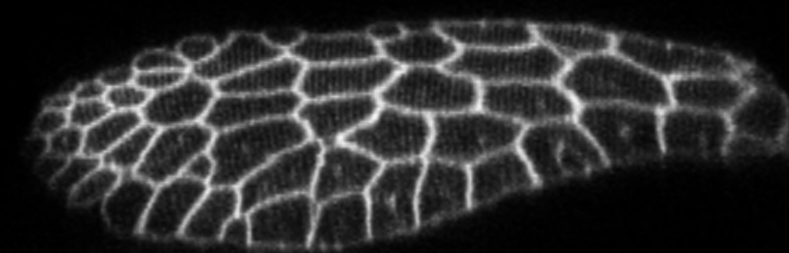

Supplement: Supplementary file 12 — Source data Fig. 3 [file 44318_2024_136_MOESM12_ESM.zip › Figure 3G/Cross-sectional view-1 to 0 myofiber-10 dpf.tif]

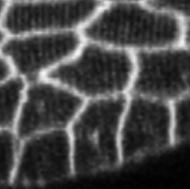

Supplement: Supplementary file 12 — Source data Fig. 3 [file 44318_2024_136_MOESM12_ESM.zip › Figure 3G/Cross-sectional view-1 to 0 myofiber-10 dpf-crop.tif]

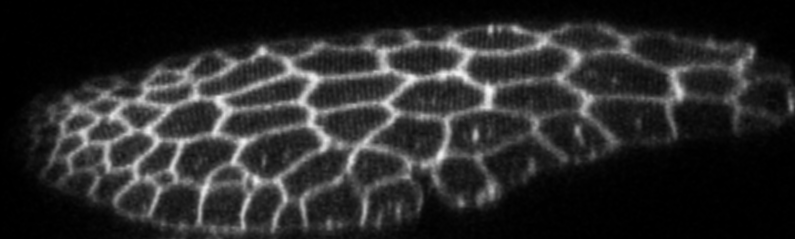

Supplement: Supplementary file 12 — Source data Fig. 3 [file 44318_2024_136_MOESM12_ESM.zip › Figure 3G/Cross-sectional view-1 to 0 myofiber-11 dpf.tif]

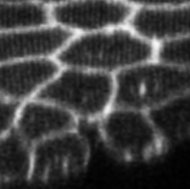

Supplement: Supplementary file 12 — Source data Fig. 3 [file 44318_2024_136_MOESM12_ESM.zip › Figure 3G/Cross-sectional view-1 to 0 myofiber-11 dpf-crop.tif]

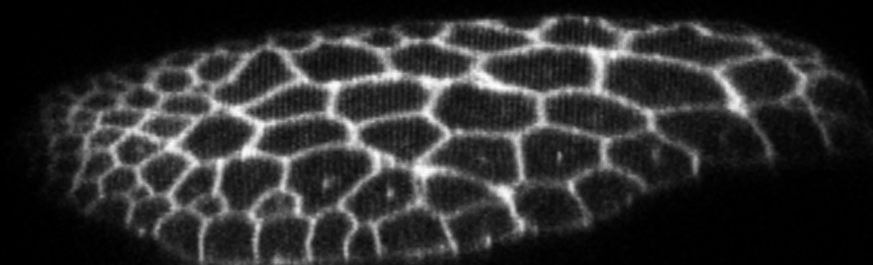

Supplement: Supplementary file 12 — Source data Fig. 3 [file 44318_2024_136_MOESM12_ESM.zip › Figure 3G/Cross-sectional view-1 to 0 myofiber-12 dpf.tif]

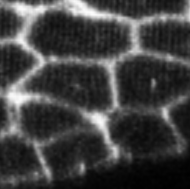

Supplement: Supplementary file 12 — Source data Fig. 3 [file 44318_2024_136_MOESM12_ESM.zip › Figure 3G/Cross-sectional view-1 to 0 myofiber-12 dpf-crop.tif]

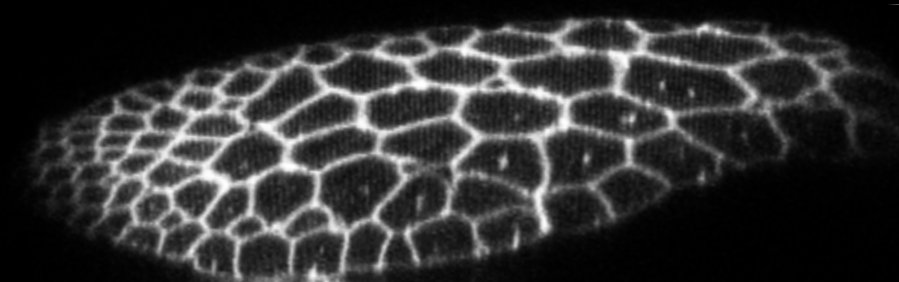

Supplement: Supplementary file 12 — Source data Fig. 3 [file 44318_2024_136_MOESM12_ESM.zip › Figure 3G/Cross-sectional view-1 to 0 myofiber-13 dpf.tif]

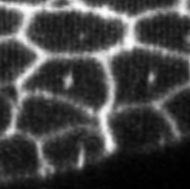

Supplement: Supplementary file 12 — Source data Fig. 3 [file 44318_2024_136_MOESM12_ESM.zip › Figure 3G/Cross-sectional view-1 to 0 myofiber-13 dpf-crop.tif]

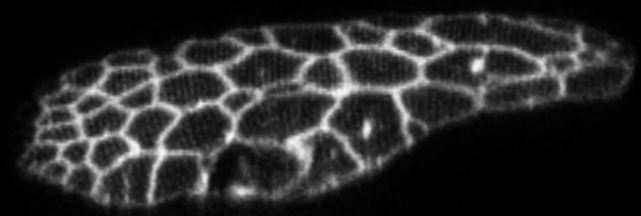

Supplement: Supplementary file 12 — Source data Fig. 3 [file 44318_2024_136_MOESM12_ESM.zip › Figure 3H/Cross-sectional view-1 to 1 myofiber-10 dpf.tif]

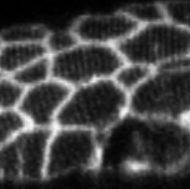

Supplement: Supplementary file 12 — Source data Fig. 3 [file 44318_2024_136_MOESM12_ESM.zip › Figure 3H/Cross-sectional view-1 to 1 myofiber-10 dpf-crop.tif]

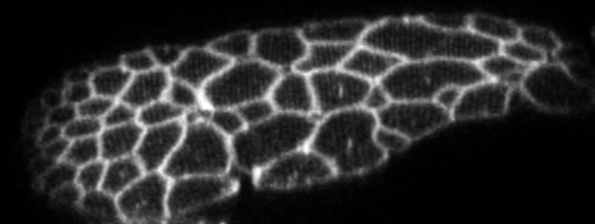

Supplement: Supplementary file 12 — Source data Fig. 3 [file 44318_2024_136_MOESM12_ESM.zip › Figure 3H/Cross-sectional view-1 to 1 myofiber-11 dpf.tif]

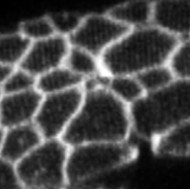

Supplement: Supplementary file 12 — Source data Fig. 3 [file 44318_2024_136_MOESM12_ESM.zip › Figure 3H/Cross-sectional view-1 to 1 myofiber-11 dpf-crop.tif]

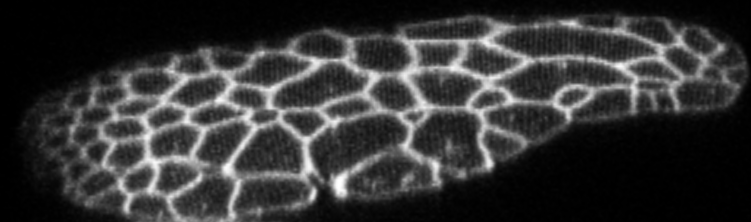

Supplement: Supplementary file 12 — Source data Fig. 3 [file 44318_2024_136_MOESM12_ESM.zip › Figure 3H/Cross-sectional view-1 to 1 myofiber-12 dpf.tif]

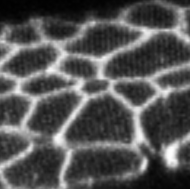

Supplement: Supplementary file 12 — Source data Fig. 3 [file 44318_2024_136_MOESM12_ESM.zip › Figure 3H/Cross-sectional view-1 to 1 myofiber-12 dpf-crop.tif]

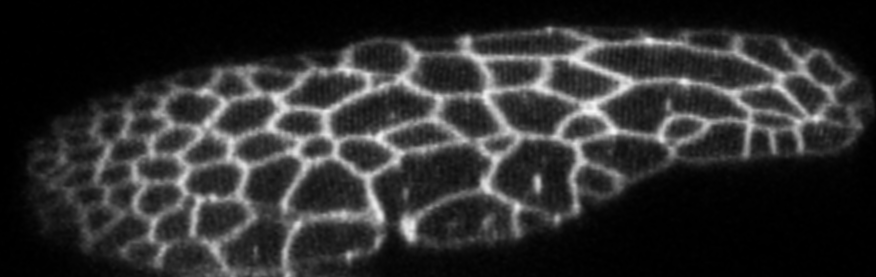

Supplement: Supplementary file 12 — Source data Fig. 3 [file 44318_2024_136_MOESM12_ESM.zip › Figure 3H/Cross-sectional view-1 to 1 myofiber-13 dpf.tif]

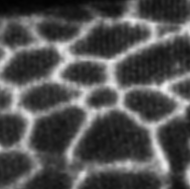

Supplement: Supplementary file 12 — Source data Fig. 3 [file 44318_2024_136_MOESM12_ESM.zip › Figure 3H/Cross-sectional view-1 to 1 myofiber-13 dpf-crop.tif]

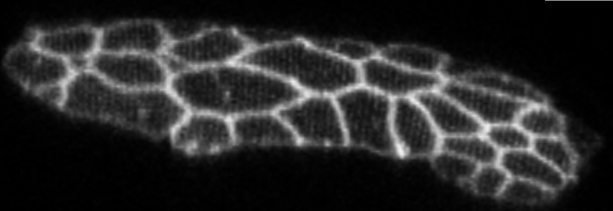

Supplement: Supplementary file 12 — Source data Fig. 3 [file 44318_2024_136_MOESM12_ESM.zip › Figure 3I/Cross-sectional view-1 to 2 myofiber-10 dpf.tif]

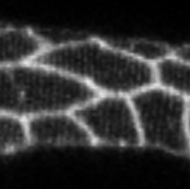

Supplement: Supplementary file 12 — Source data Fig. 3 [file 44318_2024_136_MOESM12_ESM.zip › Figure 3I/Cross-sectional view-1 to 2 myofiber-10 dpf-crop.tif]

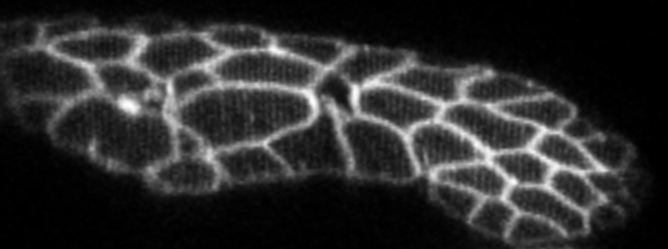

Supplement: Supplementary file 12 — Source data Fig. 3 [file 44318_2024_136_MOESM12_ESM.zip › Figure 3I/Cross-sectional view-1 to 2 myofiber-11 dpf.tif]

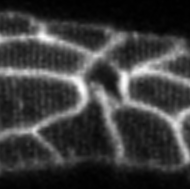

Supplement: Supplementary file 12 — Source data Fig. 3 [file 44318_2024_136_MOESM12_ESM.zip › Figure 3I/Cross-sectional view-1 to 2 myofiber-11 dpf-crop.tif]

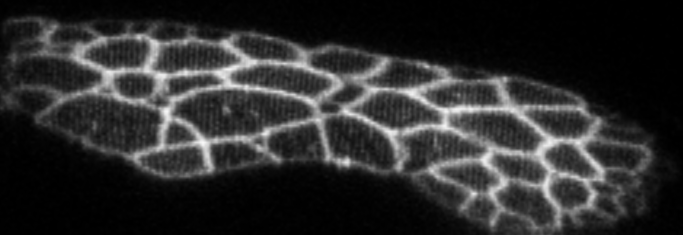

Supplement: Supplementary file 12 — Source data Fig. 3 [file 44318_2024_136_MOESM12_ESM.zip › Figure 3I/Cross-sectional view-1 to 2 myofiber-12 dpf.tif]

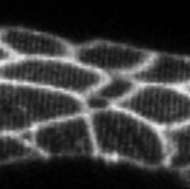

Supplement: Supplementary file 12 — Source data Fig. 3 [file 44318_2024_136_MOESM12_ESM.zip › Figure 3I/Cross-sectional view-1 to 2 myofiber-12 dpf-crop.tif]

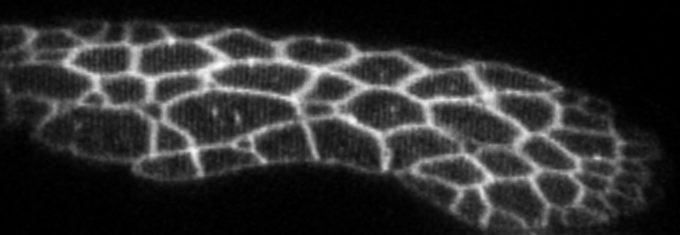

Supplement: Supplementary file 12 — Source data Fig. 3 [file 44318_2024_136_MOESM12_ESM.zip › Figure 3I/Cross-sectional view-1 to 2 myofiber-13 dpf.tif]

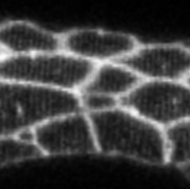

Supplement: Supplementary file 12 — Source data Fig. 3 [file 44318_2024_136_MOESM12_ESM.zip › Figure 3I/Cross-sectional view-1 to 2 myofiber-13 dpf-crop.tif]

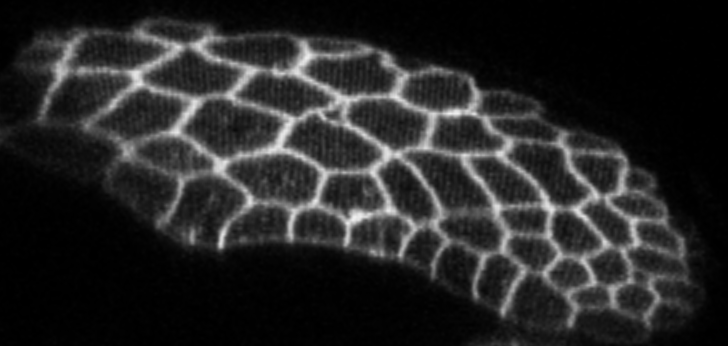

Supplement: Supplementary file 12 — Source data Fig. 3 [file 44318_2024_136_MOESM12_ESM.zip › Figure 3J/Cross-sectional view-1 to 3 myofiber-10 dpf.tif]

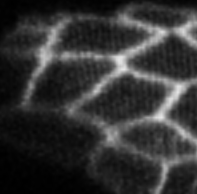

Supplement: Supplementary file 12 — Source data Fig. 3 [file 44318_2024_136_MOESM12_ESM.zip › Figure 3J/Cross-sectional view-1 to 3 myofiber-10 dpf-crop.tif]

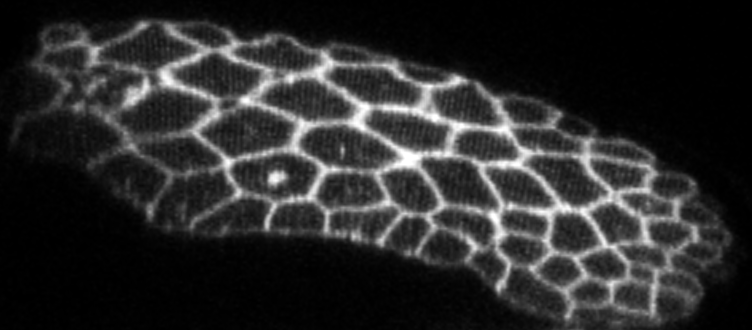

Supplement: Supplementary file 12 — Source data Fig. 3 [file 44318_2024_136_MOESM12_ESM.zip › Figure 3J/Cross-sectional view-1 to 3 myofiber-11 dpf.tif]

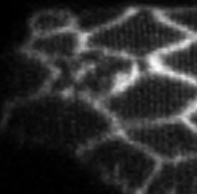

Supplement: Supplementary file 12 — Source data Fig. 3 [file 44318_2024_136_MOESM12_ESM.zip › Figure 3J/Cross-sectional view-1 to 3 myofiber-11 dpf-crop.tif]

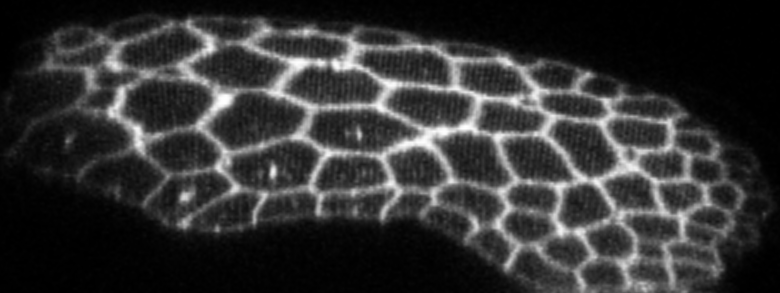

Supplement: Supplementary file 12 — Source data Fig. 3 [file 44318_2024_136_MOESM12_ESM.zip › Figure 3J/Cross-sectional view-1 to 3 myofiber-12 dpf.tif]

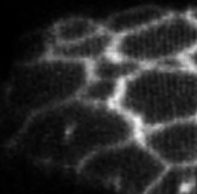

Supplement: Supplementary file 12 — Source data Fig. 3 [file 44318_2024_136_MOESM12_ESM.zip › Figure 3J/Cross-sectional view-1 to 3 myofiber-12 dpf-crop.tif]

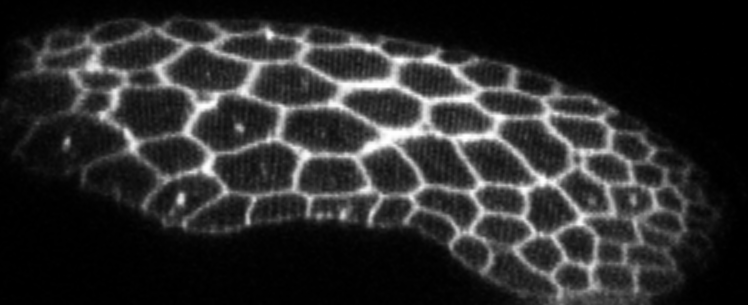

Supplement: Supplementary file 12 — Source data Fig. 3 [file 44318_2024_136_MOESM12_ESM.zip › Figure 3J/Cross-sectional view-1 to 3 myofiber-13 dpf.tif]

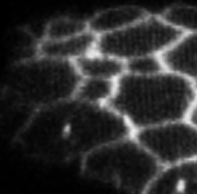

Supplement: Supplementary file 12 — Source data Fig. 3 [file 44318_2024_136_MOESM12_ESM.zip › Figure 3J/Cross-sectional view-1 to 3 myofiber-14 dpf-crop.tif]

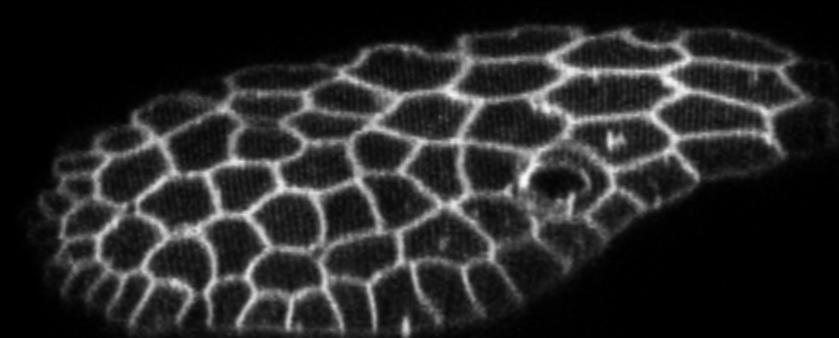

Supplement: Supplementary file 12 — Source data Fig. 3 [file 44318_2024_136_MOESM12_ESM.zip › Figure 3K/Cross-sectional view-1 to 4 myofiber-10 dpf.tif]

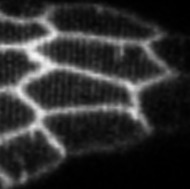

Supplement: Supplementary file 12 — Source data Fig. 3 [file 44318_2024_136_MOESM12_ESM.zip › Figure 3K/Cross-sectional view-1 to 4 myofiber-10 dpf-crop.tif]

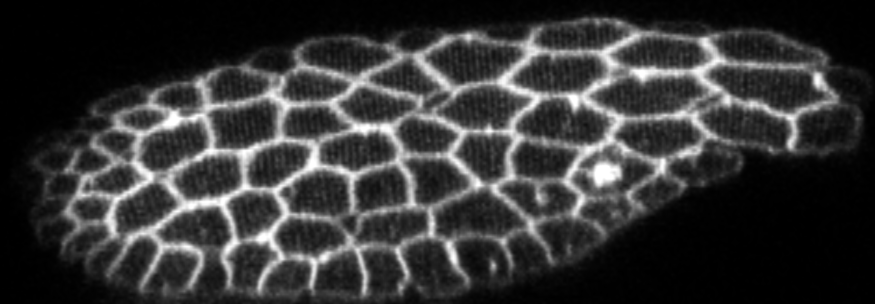

Supplement: Supplementary file 12 — Source data Fig. 3 [file 44318_2024_136_MOESM12_ESM.zip › Figure 3K/Cross-sectional view-1 to 4 myofiber-11 dpf.tif]

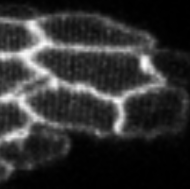

Supplement: Supplementary file 12 — Source data Fig. 3 [file 44318_2024_136_MOESM12_ESM.zip › Figure 3K/Cross-sectional view-1 to 4 myofiber-11 dpf-crop.tif]

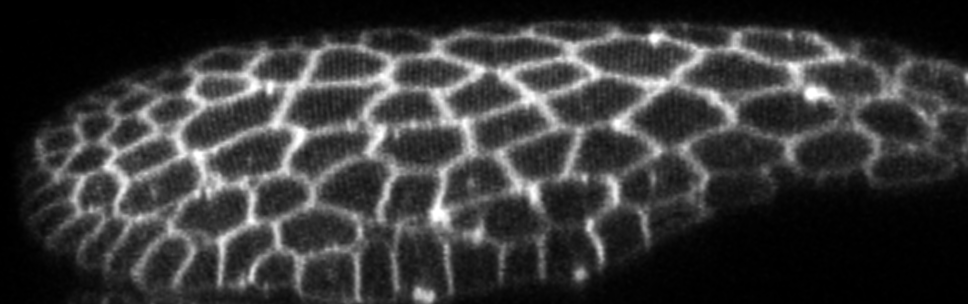

Supplement: Supplementary file 12 — Source data Fig. 3 [file 44318_2024_136_MOESM12_ESM.zip › Figure 3K/Cross-sectional view-1 to 4 myofiber-12 dpf.tif]

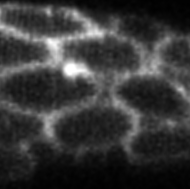

Supplement: Supplementary file 12 — Source data Fig. 3 [file 44318_2024_136_MOESM12_ESM.zip › Figure 3K/Cross-sectional view-1 to 4 myofiber-12 dpf-crop.tif]

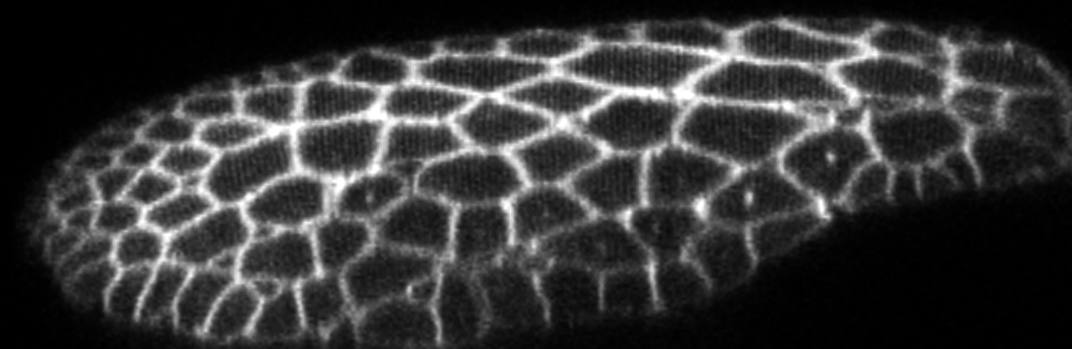

Supplement: Supplementary file 12 — Source data Fig. 3 [file 44318_2024_136_MOESM12_ESM.zip › Figure 3K/Cross-sectional view-1 to 4 myofiber-13 dpf.tif]

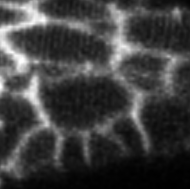

Supplement: Supplementary file 12 — Source data Fig. 3 [file 44318_2024_136_MOESM12_ESM.zip › Figure 3K/Cross-sectional view-1 to 4 myofiber-13 dpf-crop.tif]

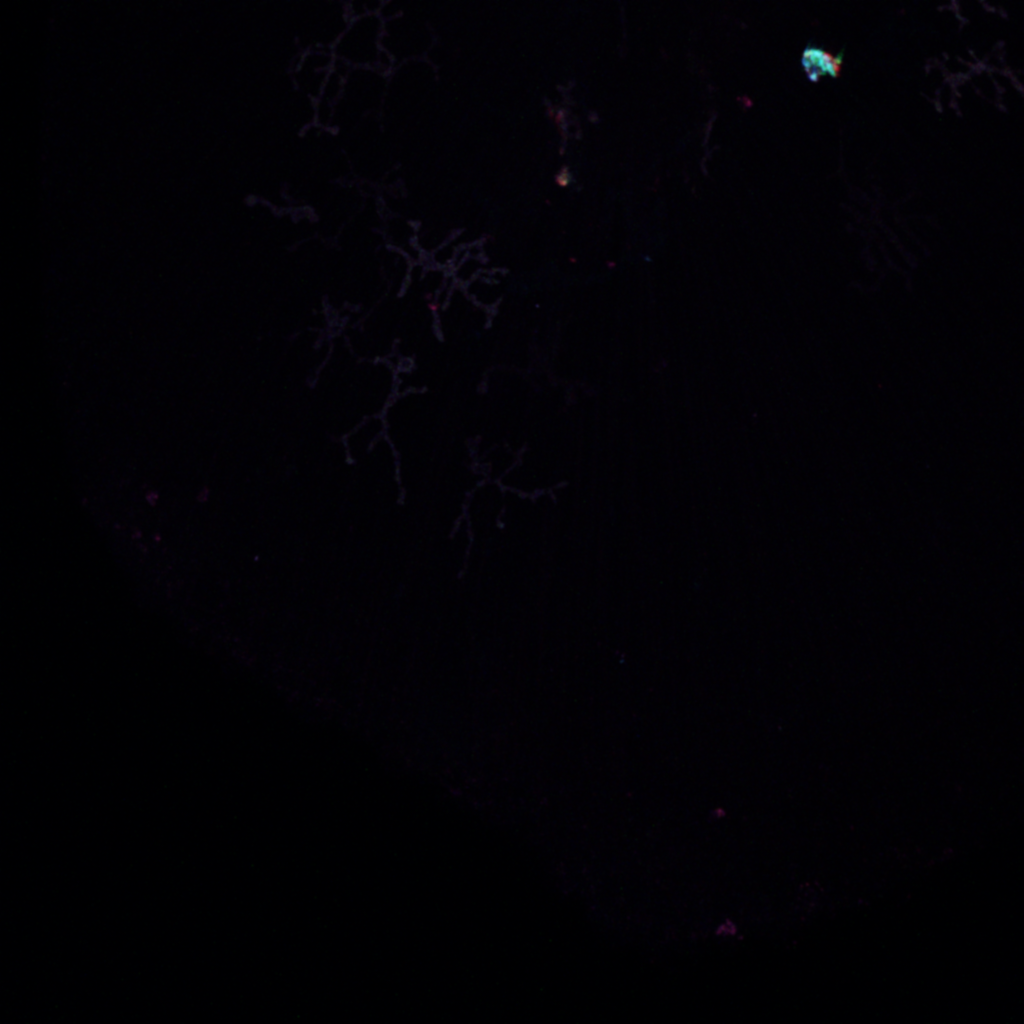

Supplement: Supplementary file 13 — Source data Fig. 4 [file 44318_2024_136_MOESM13_ESM.zip › Figure 4C/palmuscle-Multi-10 dpf-1.tif]

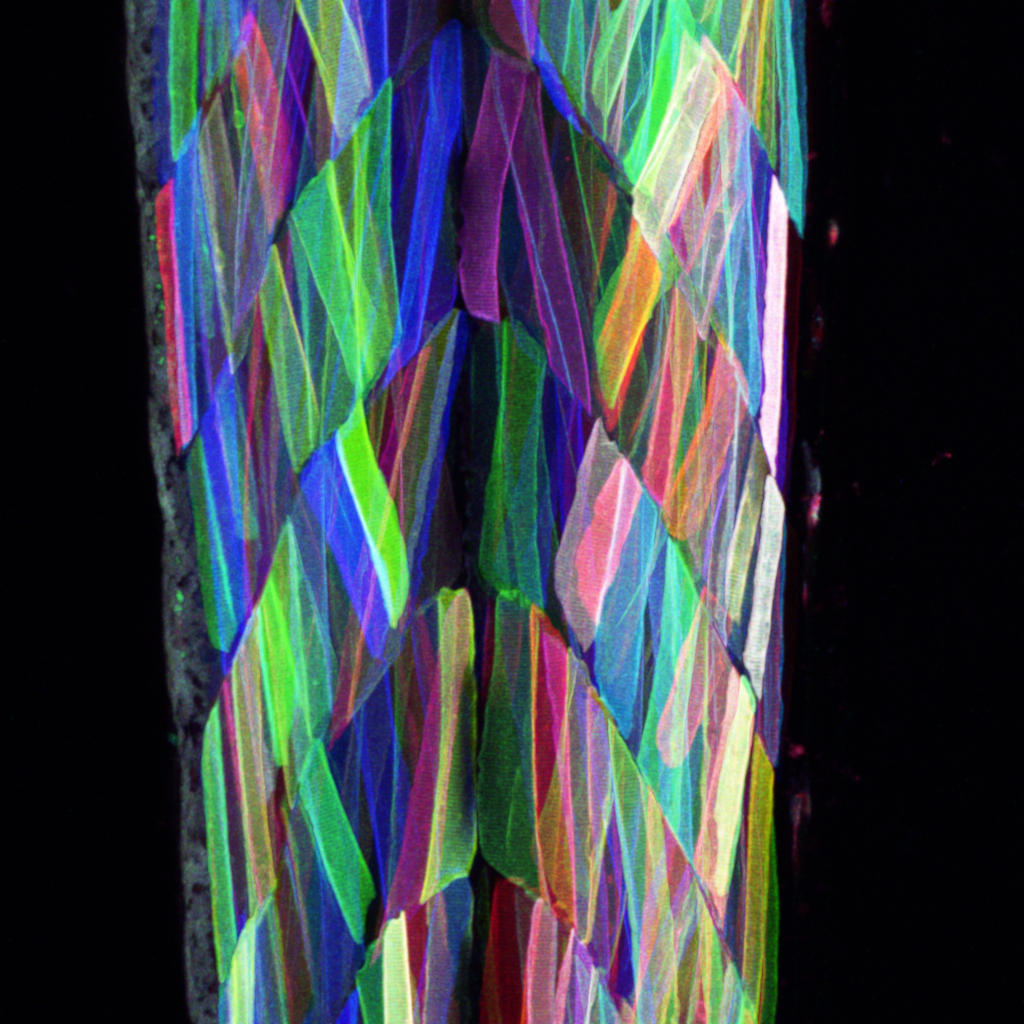

Supplement: Supplementary file 13 — Source data Fig. 4 [file 44318_2024_136_MOESM13_ESM.zip › Figure 4C/palmuscle-Multi-10 dpf-10.tif]

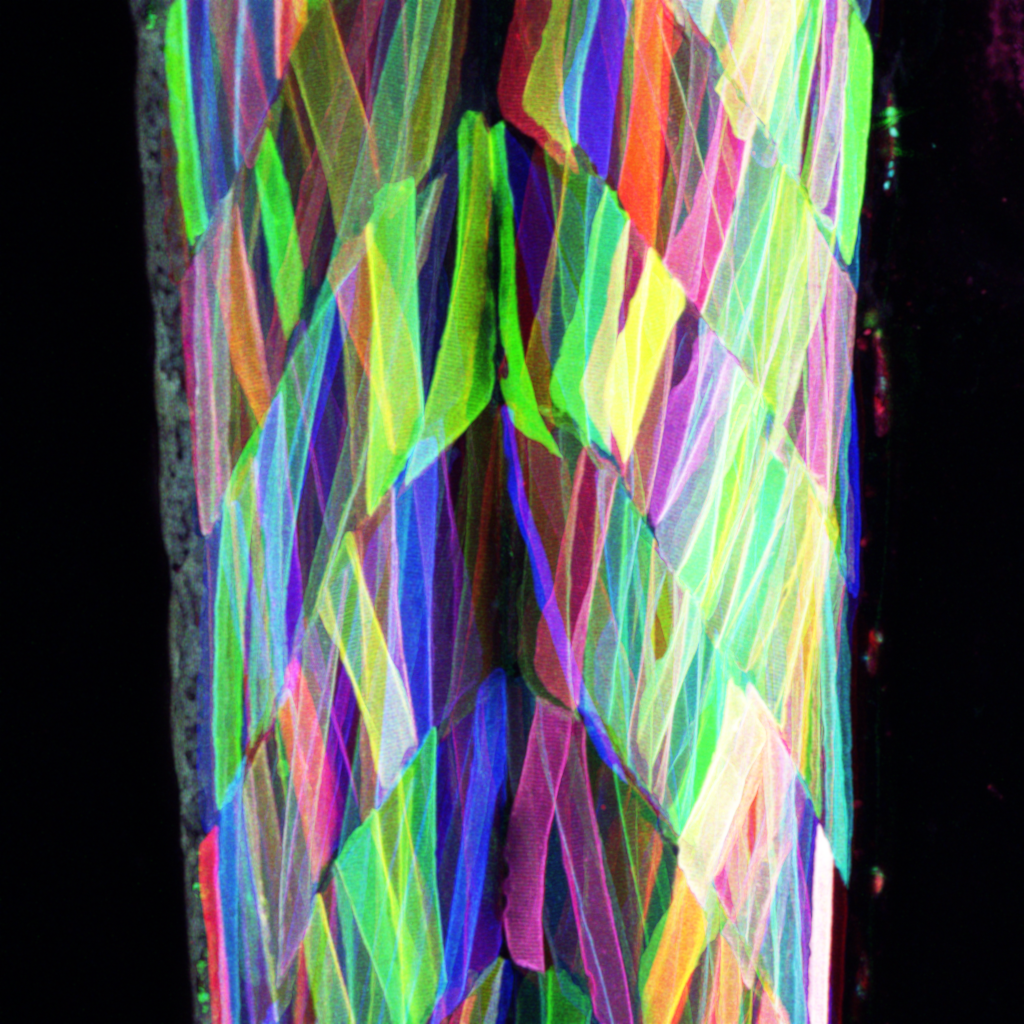

Supplement: Supplementary file 13 — Source data Fig. 4 [file 44318_2024_136_MOESM13_ESM.zip › Figure 4C/palmuscle-Multi-10 dpf-11.tif]

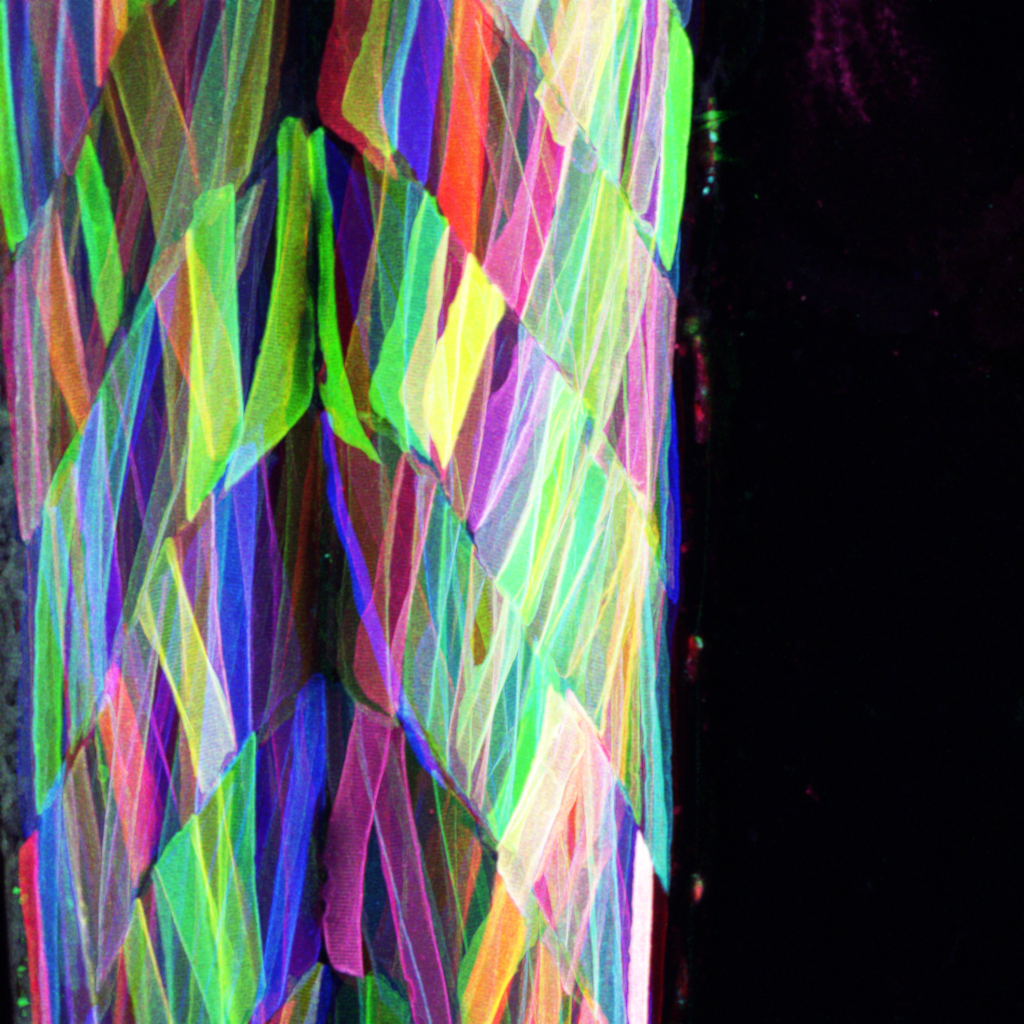

Supplement: Supplementary file 13 — Source data Fig. 4 [file 44318_2024_136_MOESM13_ESM.zip › Figure 4C/palmuscle-Multi-10 dpf-12.tif]

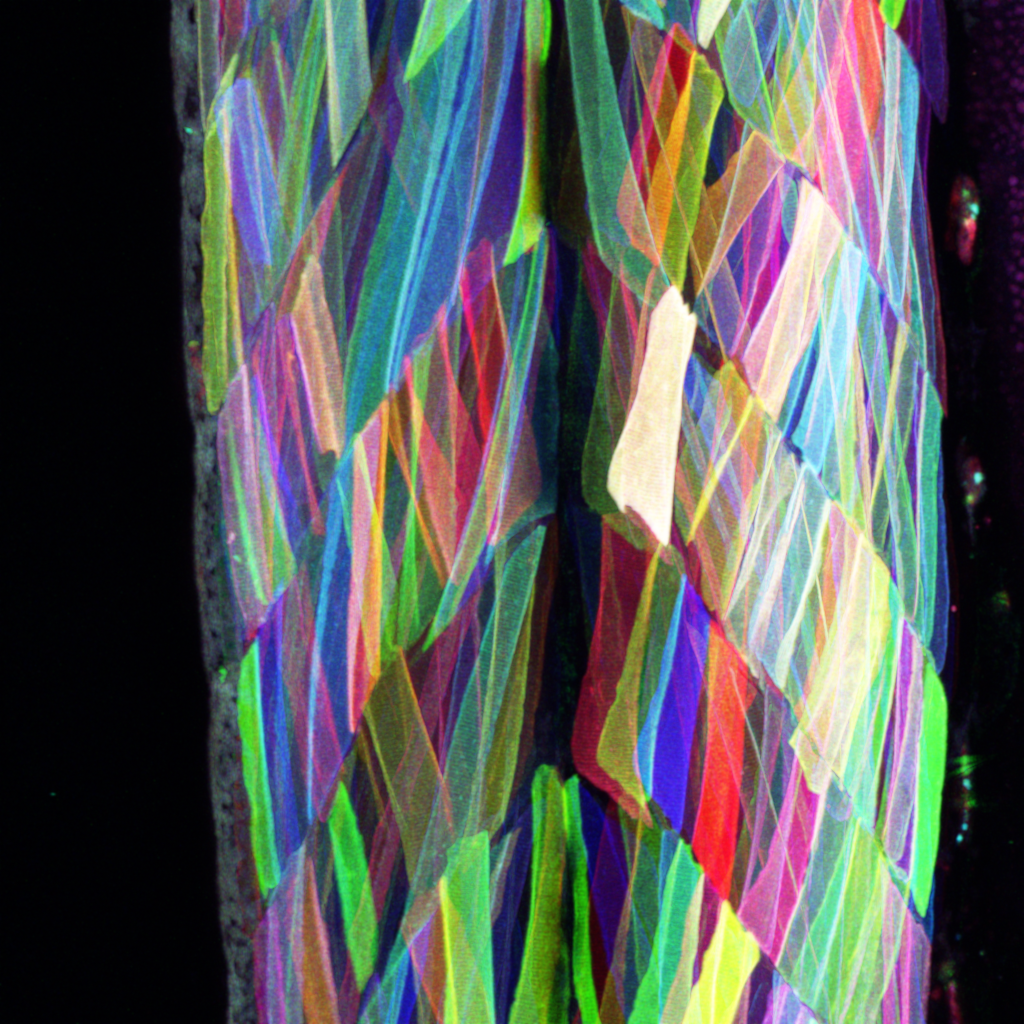

Supplement: Supplementary file 13 — Source data Fig. 4 [file 44318_2024_136_MOESM13_ESM.zip › Figure 4C/palmuscle-Multi-10 dpf-13.tif]

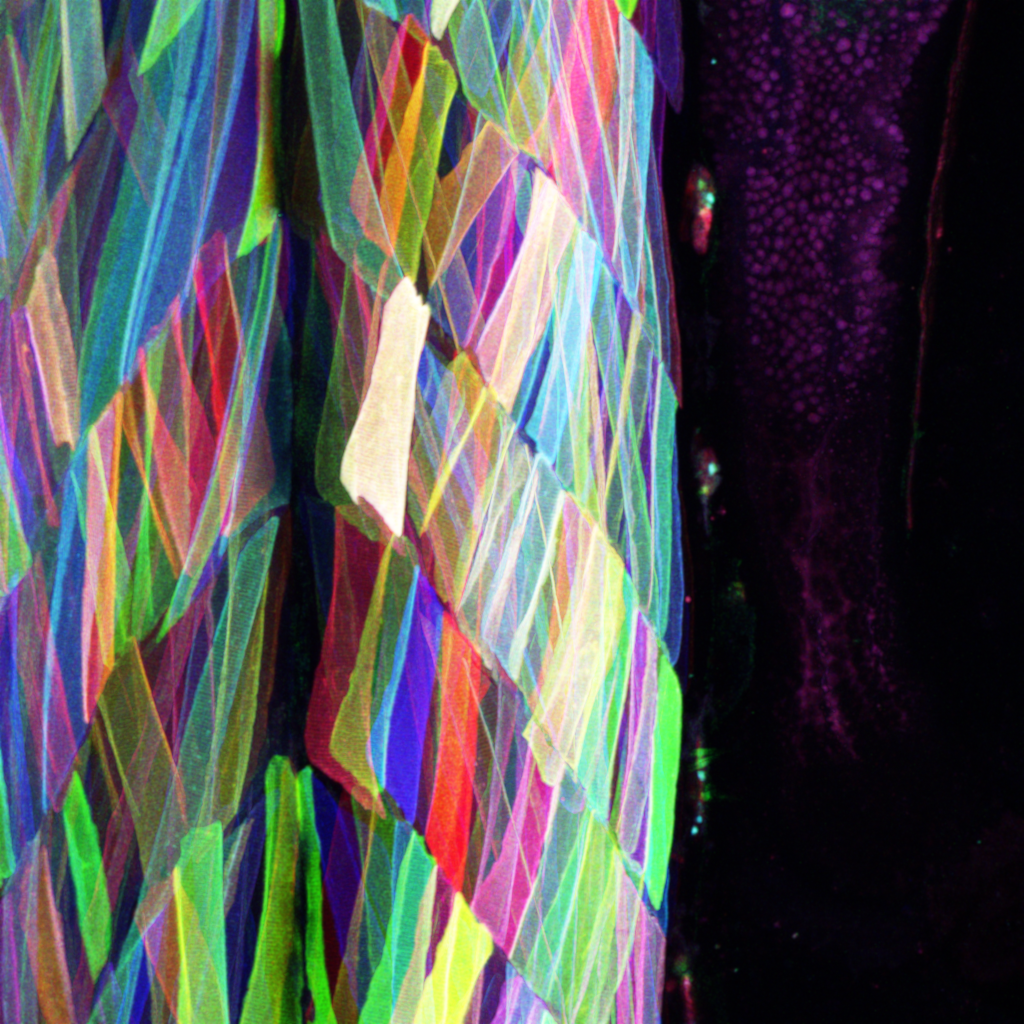

Supplement: Supplementary file 13 — Source data Fig. 4 [file 44318_2024_136_MOESM13_ESM.zip › Figure 4C/palmuscle-Multi-10 dpf-14.tif]

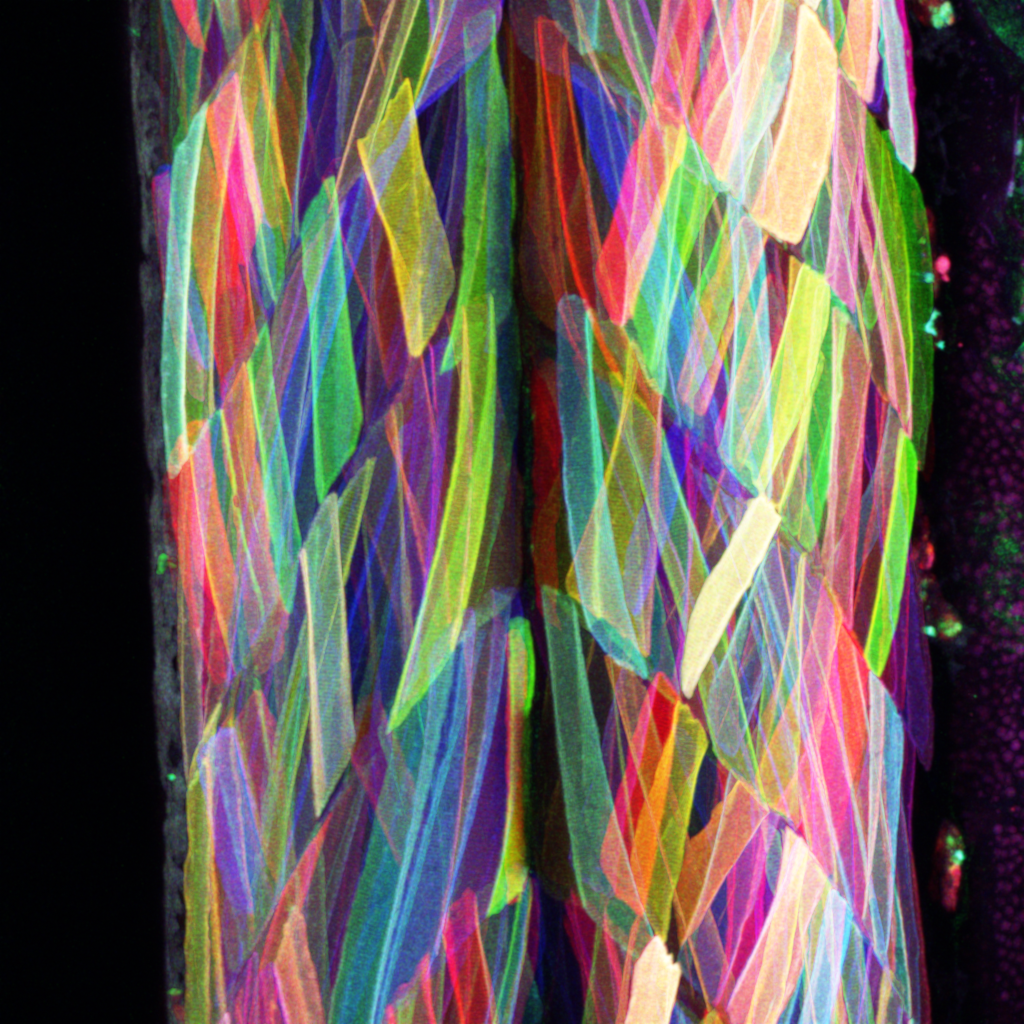

Supplement: Supplementary file 13 — Source data Fig. 4 [file 44318_2024_136_MOESM13_ESM.zip › Figure 4C/palmuscle-Multi-10 dpf-15.tif]

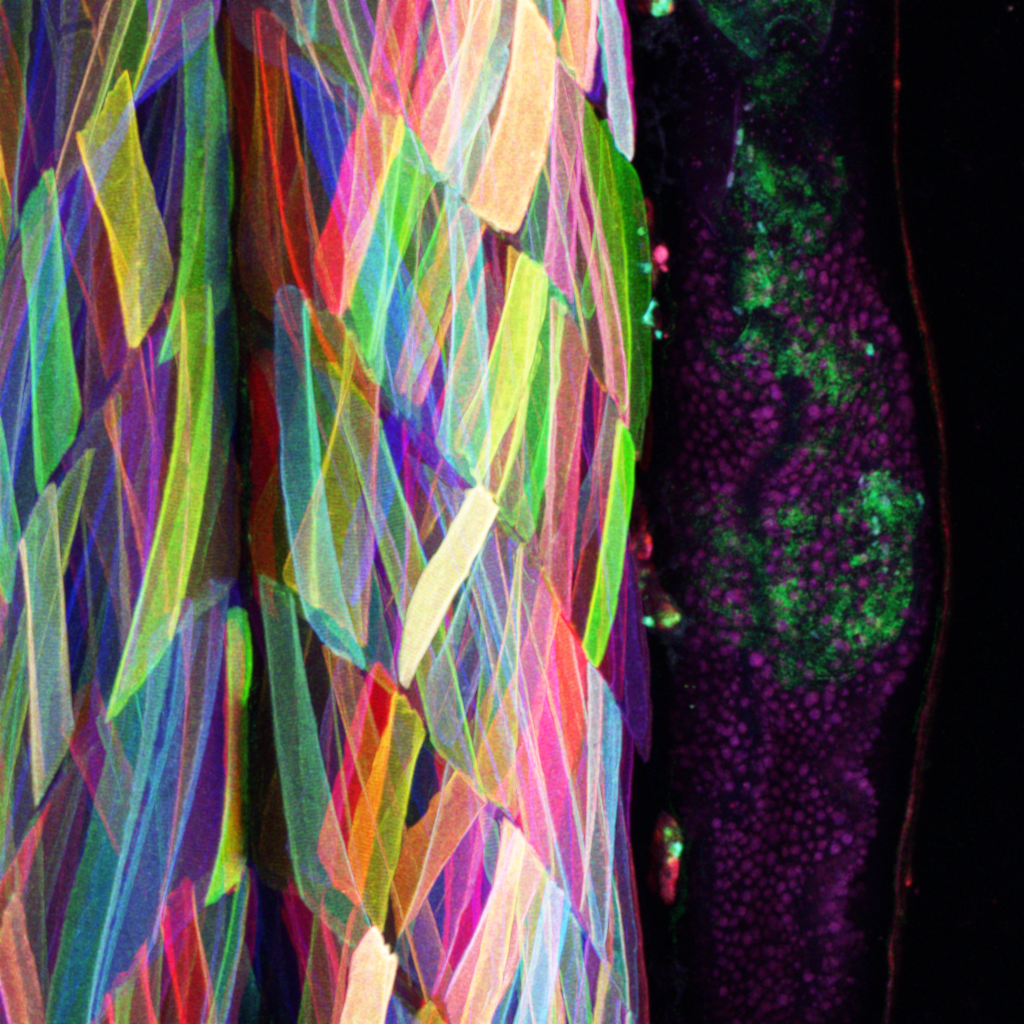

Supplement: Supplementary file 13 — Source data Fig. 4 [file 44318_2024_136_MOESM13_ESM.zip › Figure 4C/palmuscle-Multi-10 dpf-16.tif]

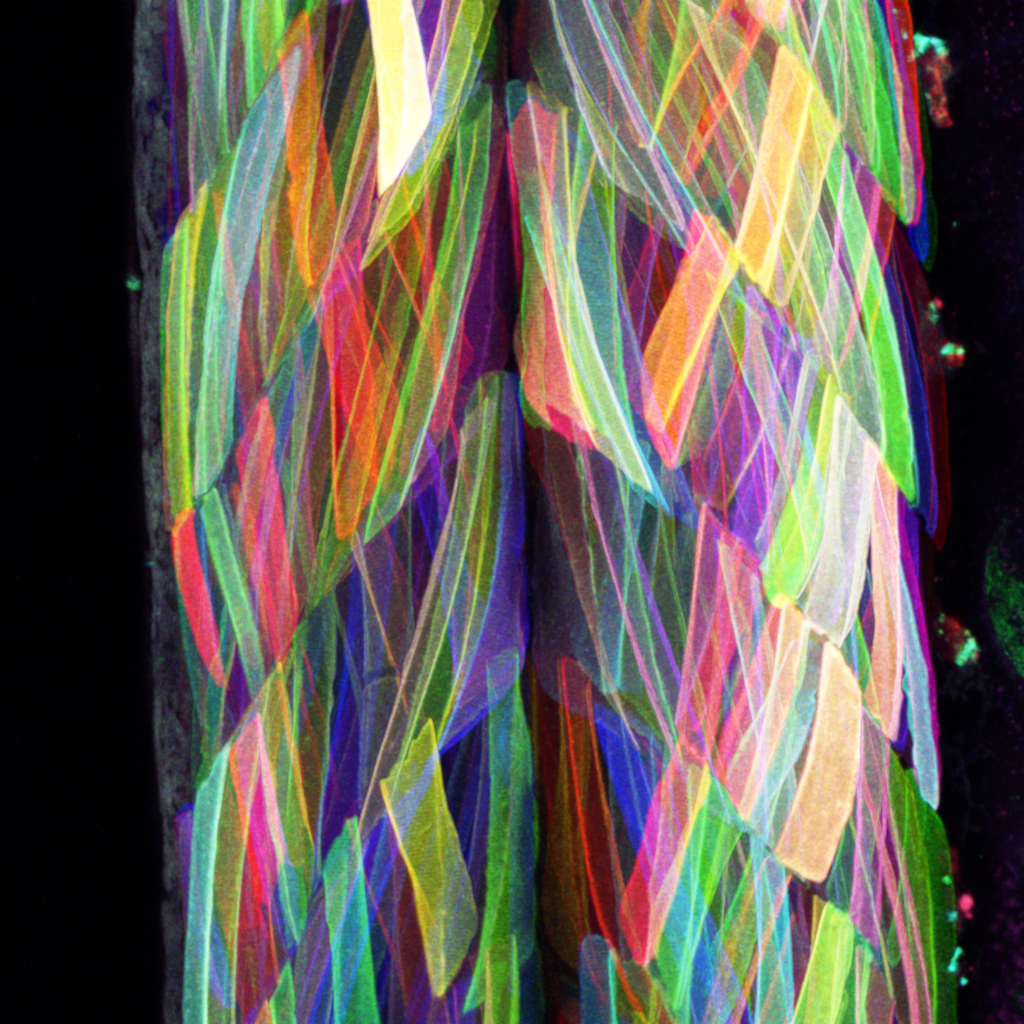

Supplement: Supplementary file 13 — Source data Fig. 4 [file 44318_2024_136_MOESM13_ESM.zip › Figure 4C/palmuscle-Multi-10 dpf-17.tif]

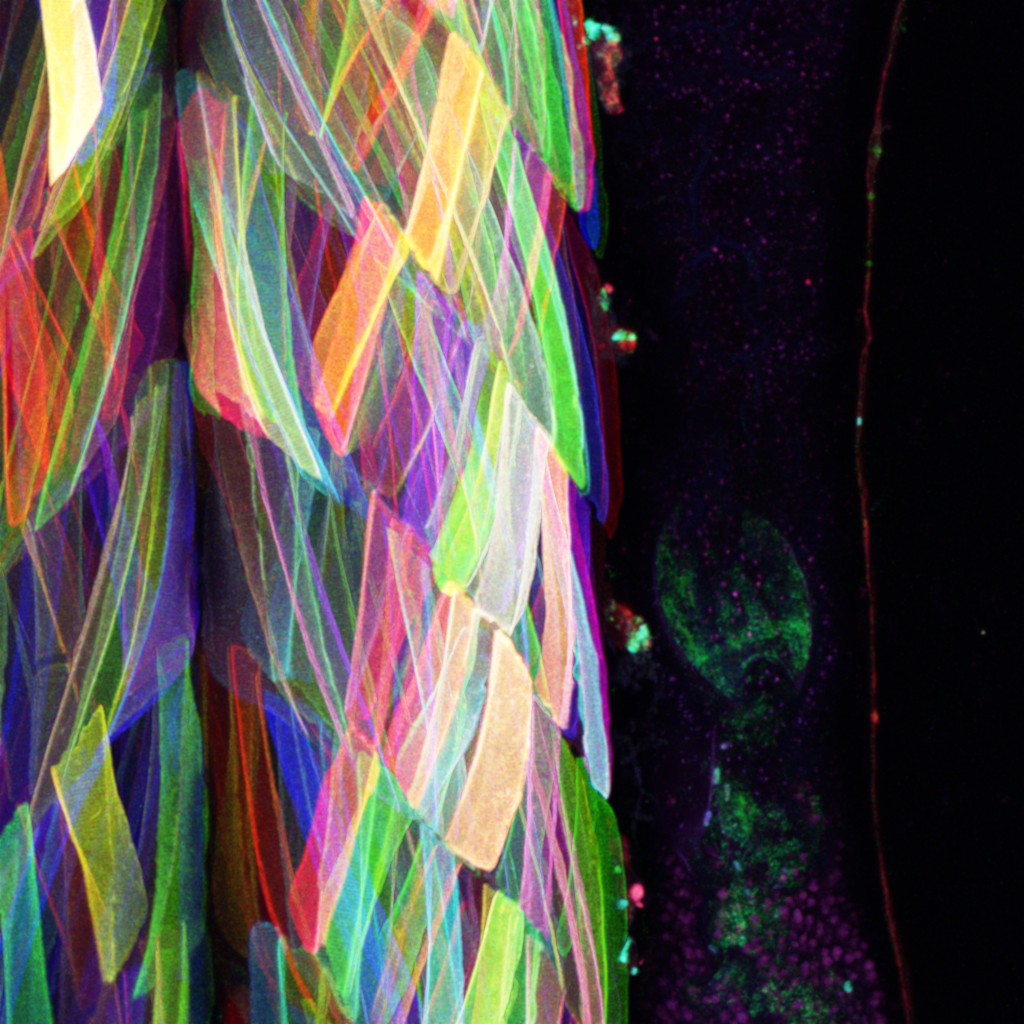

Supplement: Supplementary file 13 — Source data Fig. 4 [file 44318_2024_136_MOESM13_ESM.zip › Figure 4C/palmuscle-Multi-10 dpf-18.tif]

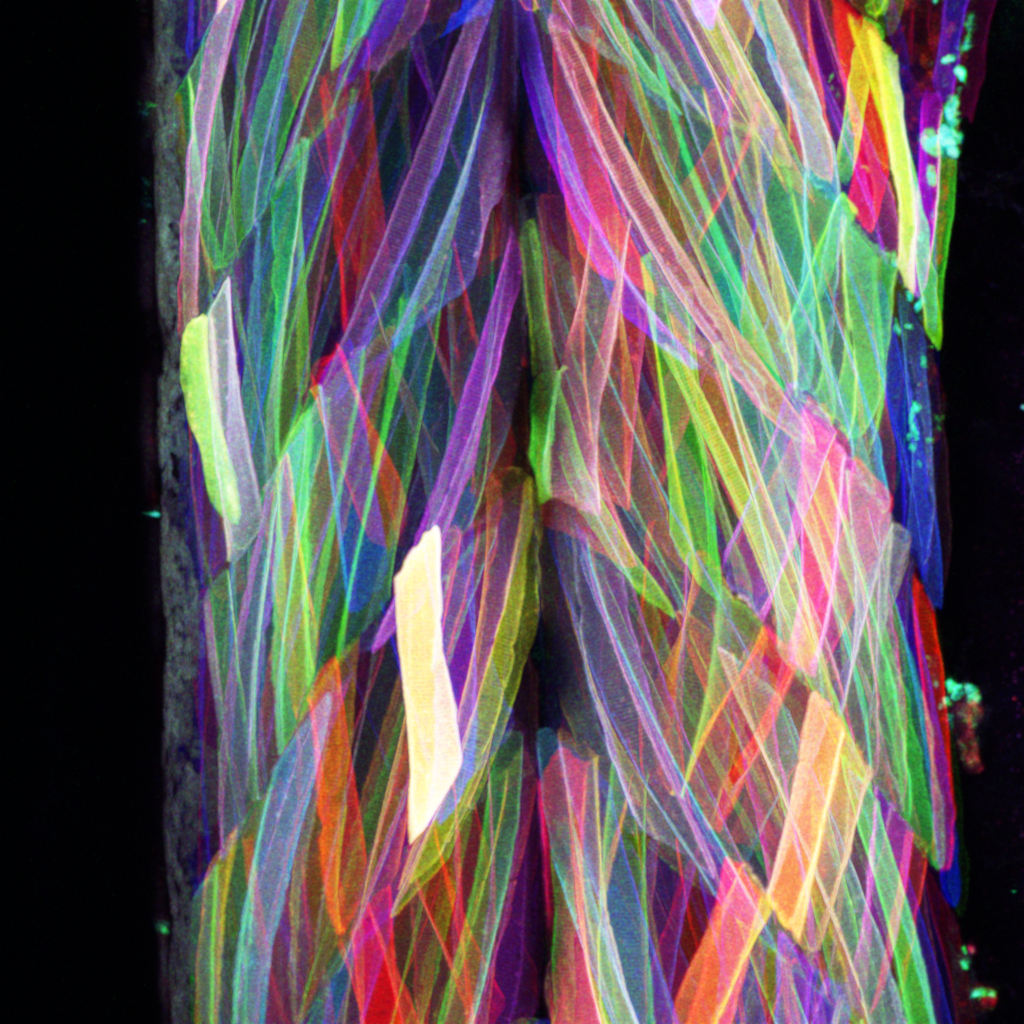

Supplement: Supplementary file 13 — Source data Fig. 4 [file 44318_2024_136_MOESM13_ESM.zip › Figure 4C/palmuscle-Multi-10 dpf-19.tif]

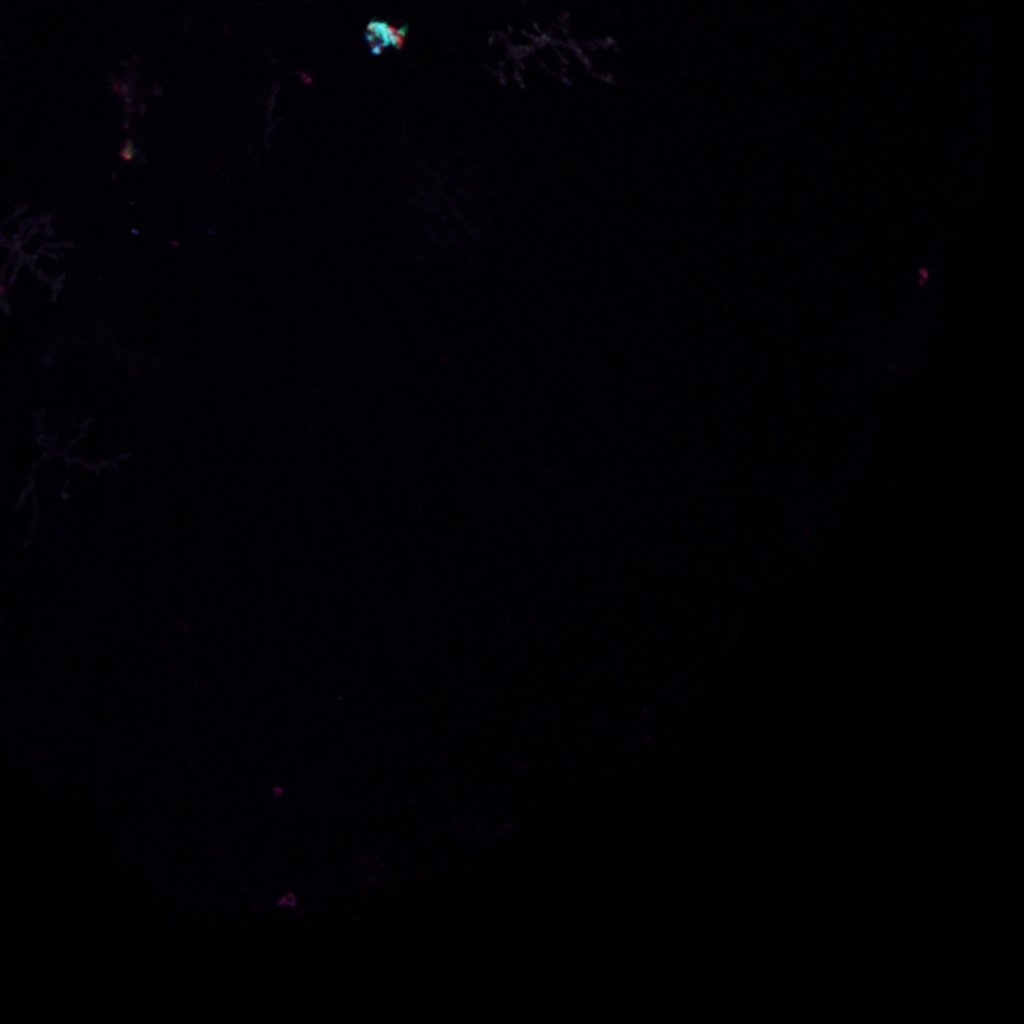

Supplement: Supplementary file 13 — Source data Fig. 4 [file 44318_2024_136_MOESM13_ESM.zip › Figure 4C/palmuscle-Multi-10 dpf-2.tif]

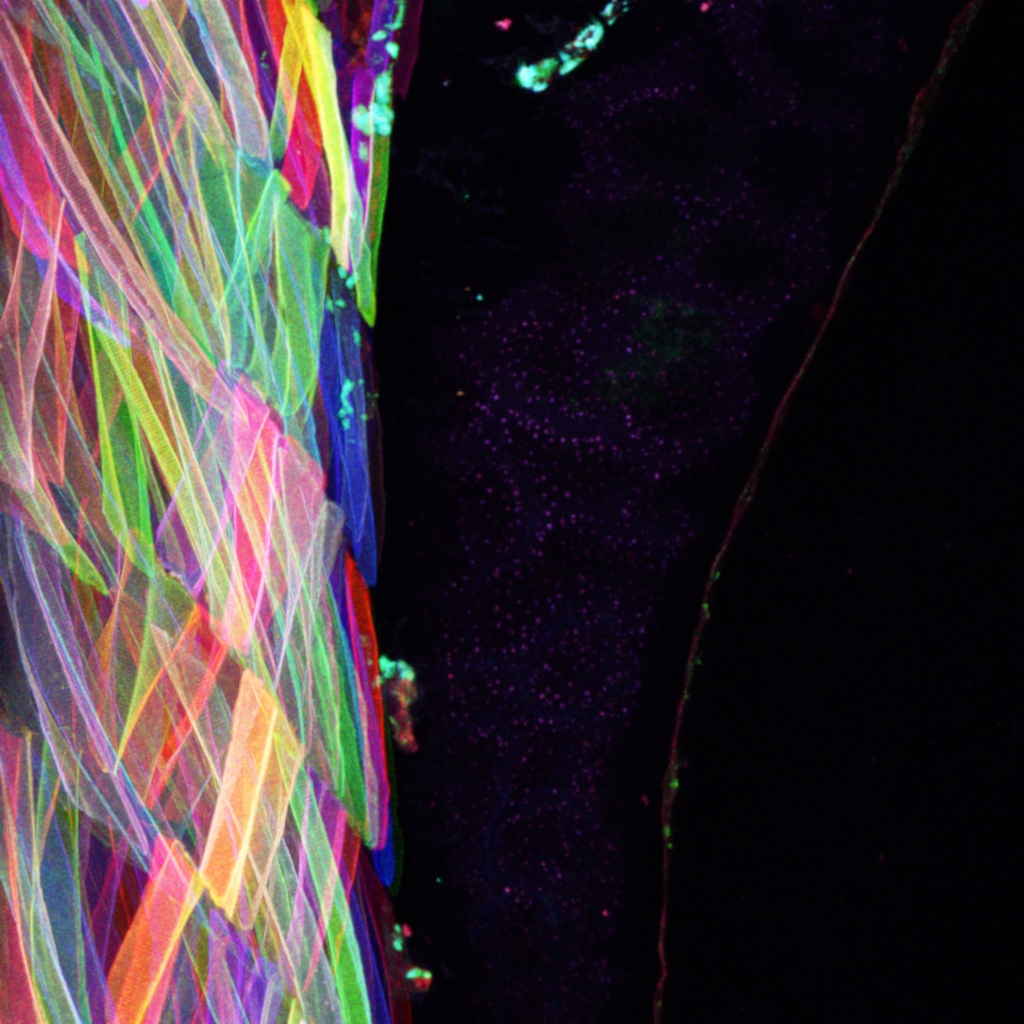

Supplement: Supplementary file 13 — Source data Fig. 4 [file 44318_2024_136_MOESM13_ESM.zip › Figure 4C/palmuscle-Multi-10 dpf-20.tif]

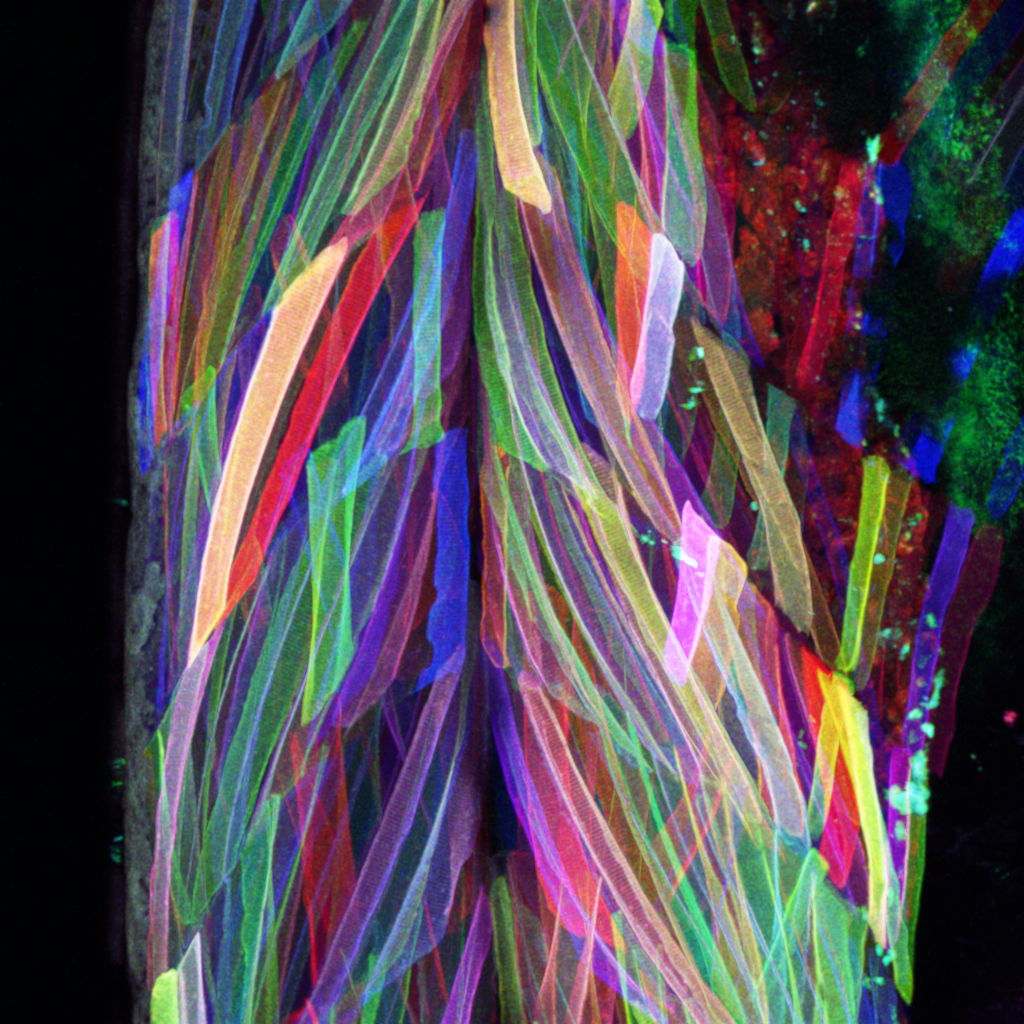

Supplement: Supplementary file 13 — Source data Fig. 4 [file 44318_2024_136_MOESM13_ESM.zip › Figure 4C/palmuscle-Multi-10 dpf-21.tif]

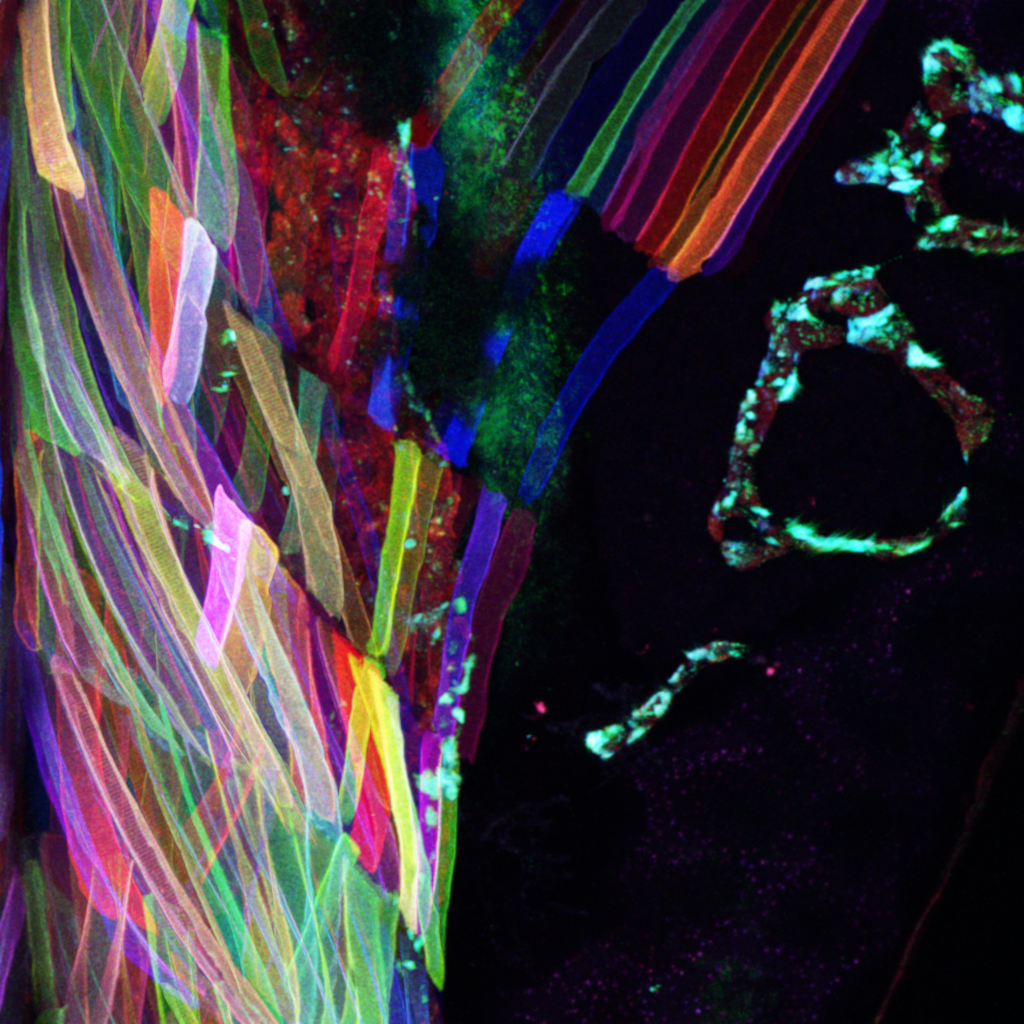

Supplement: Supplementary file 13 — Source data Fig. 4 [file 44318_2024_136_MOESM13_ESM.zip › Figure 4C/palmuscle-Multi-10 dpf-22.tif]

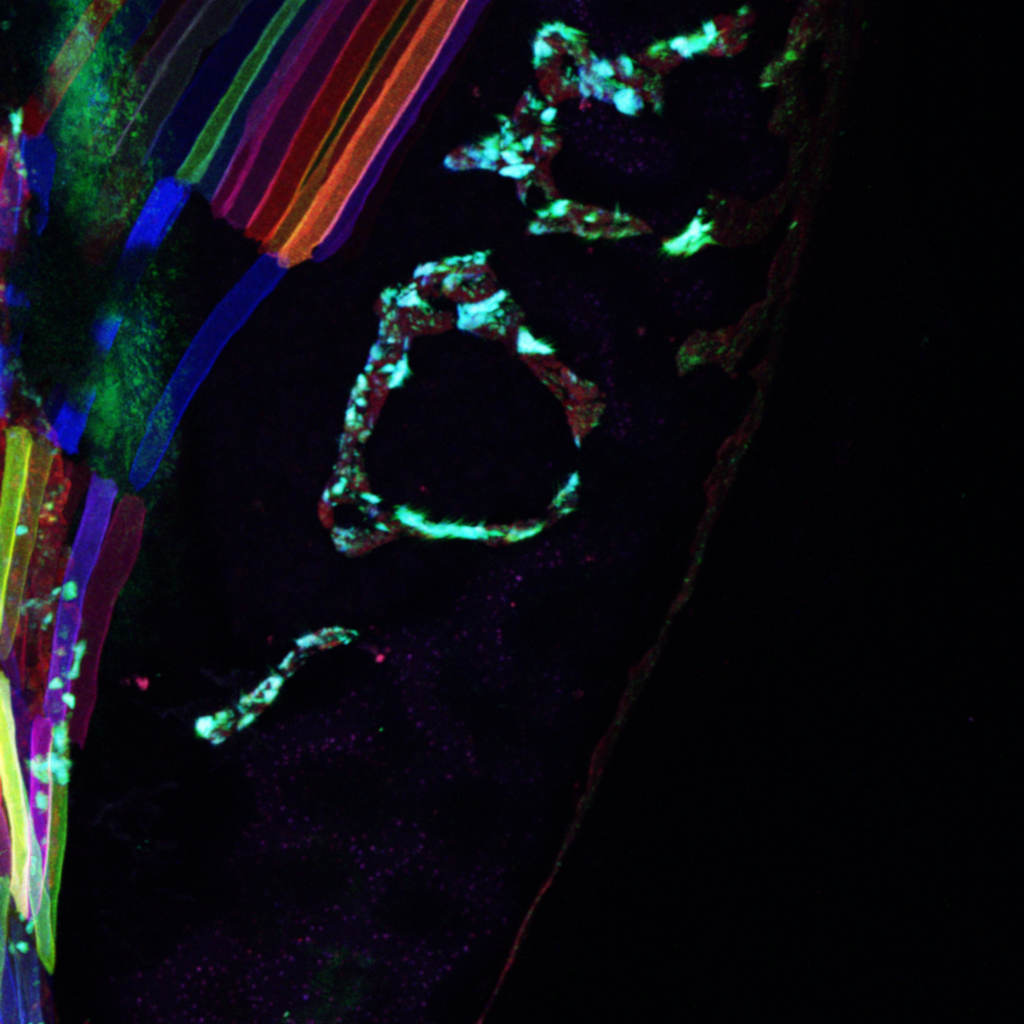

Supplement: Supplementary file 13 — Source data Fig. 4 [file 44318_2024_136_MOESM13_ESM.zip › Figure 4C/palmuscle-Multi-10 dpf-23.tif]

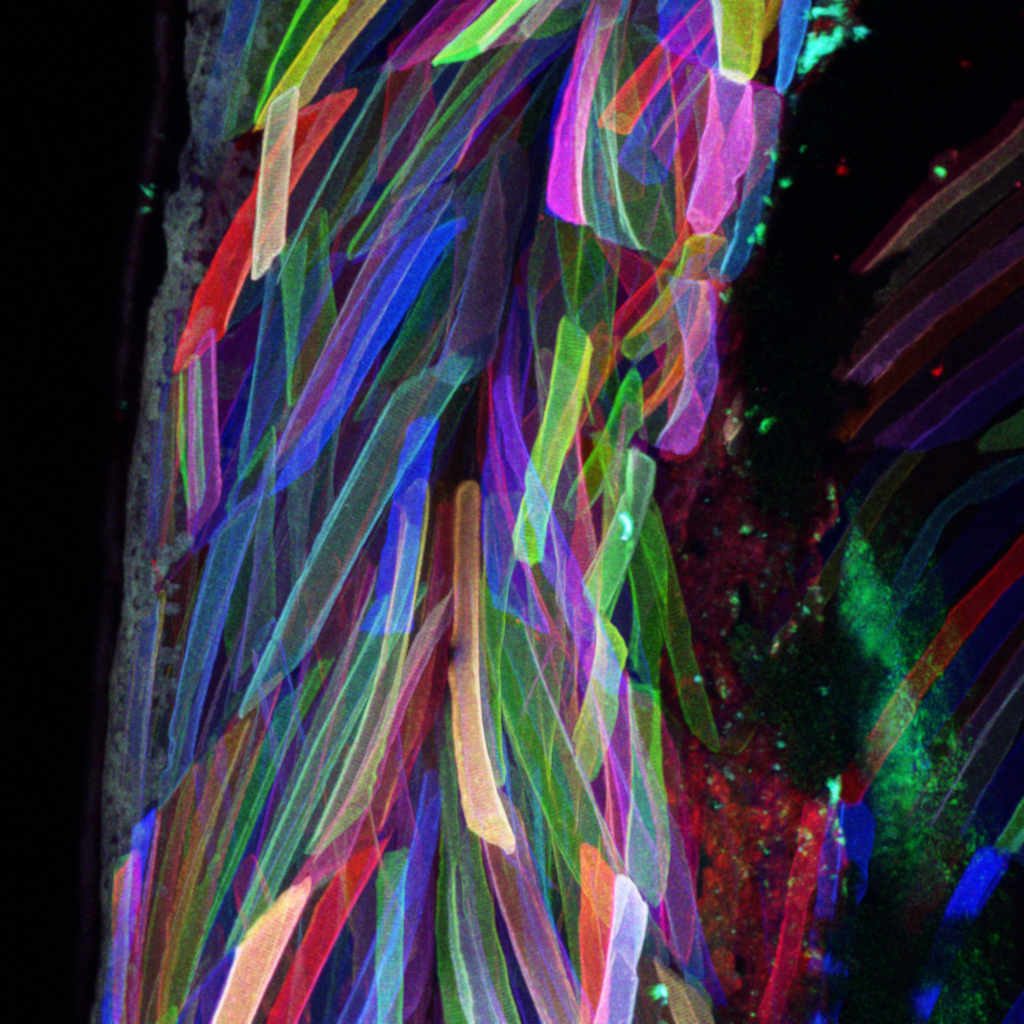

Supplement: Supplementary file 13 — Source data Fig. 4 [file 44318_2024_136_MOESM13_ESM.zip › Figure 4C/palmuscle-Multi-10 dpf-24.tif]

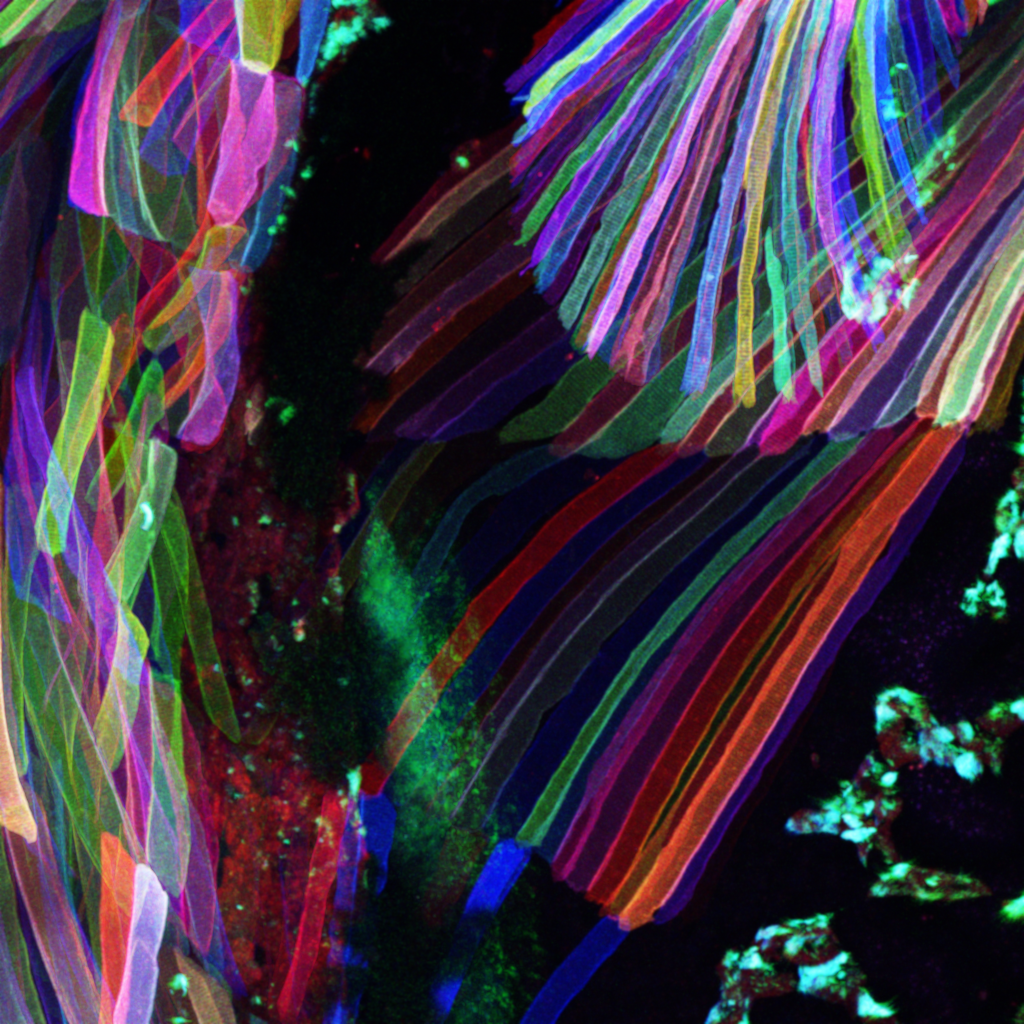

Supplement: Supplementary file 13 — Source data Fig. 4 [file 44318_2024_136_MOESM13_ESM.zip › Figure 4C/palmuscle-Multi-10 dpf-25.tif]

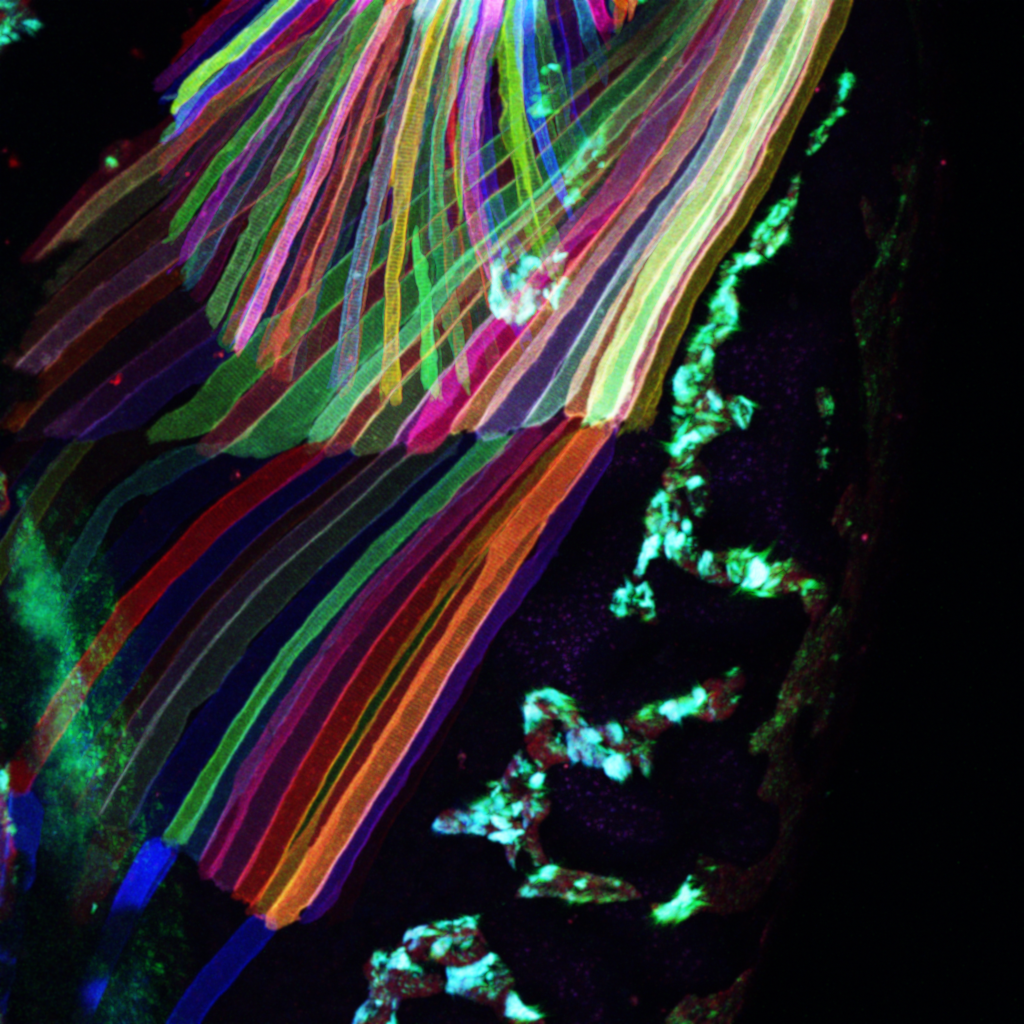

Supplement: Supplementary file 13 — Source data Fig. 4 [file 44318_2024_136_MOESM13_ESM.zip › Figure 4C/palmuscle-Multi-10 dpf-26.tif]

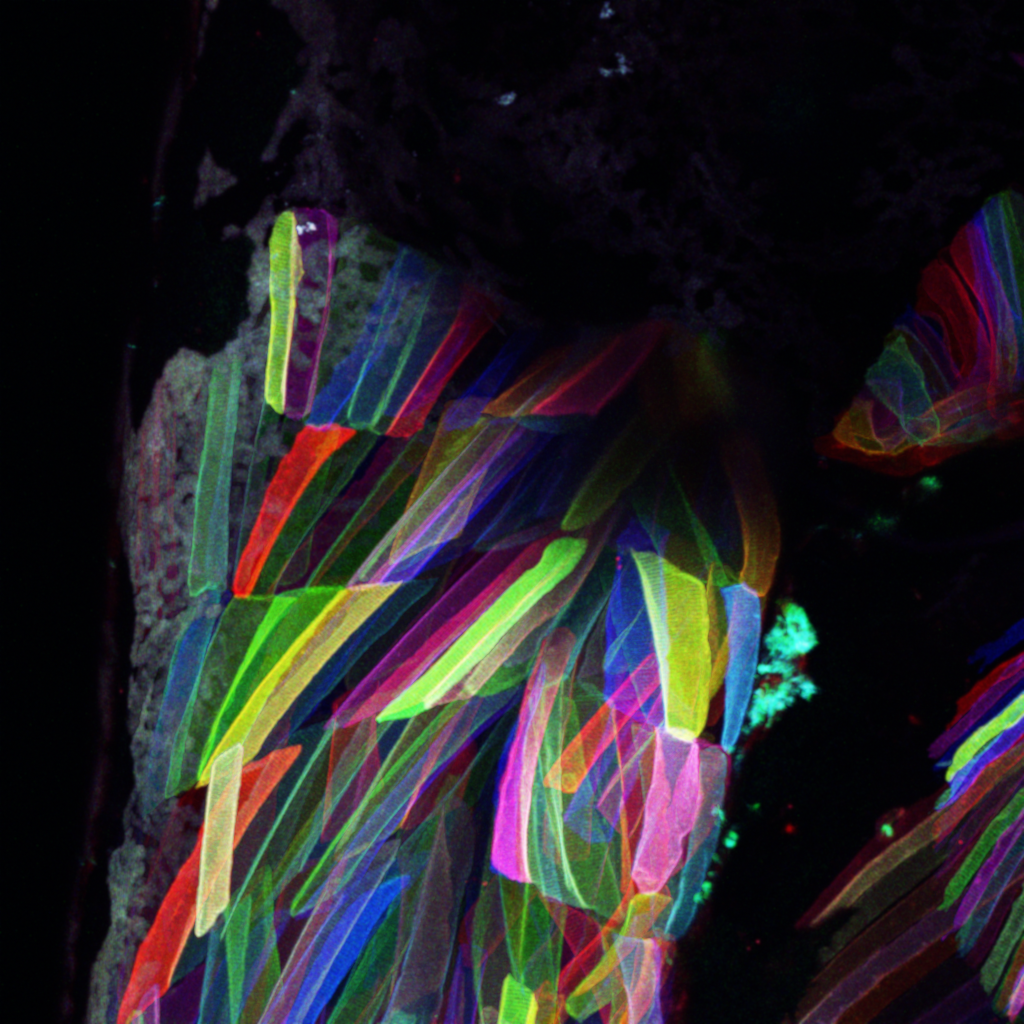

Supplement: Supplementary file 13 — Source data Fig. 4 [file 44318_2024_136_MOESM13_ESM.zip › Figure 4C/palmuscle-Multi-10 dpf-27.tif]

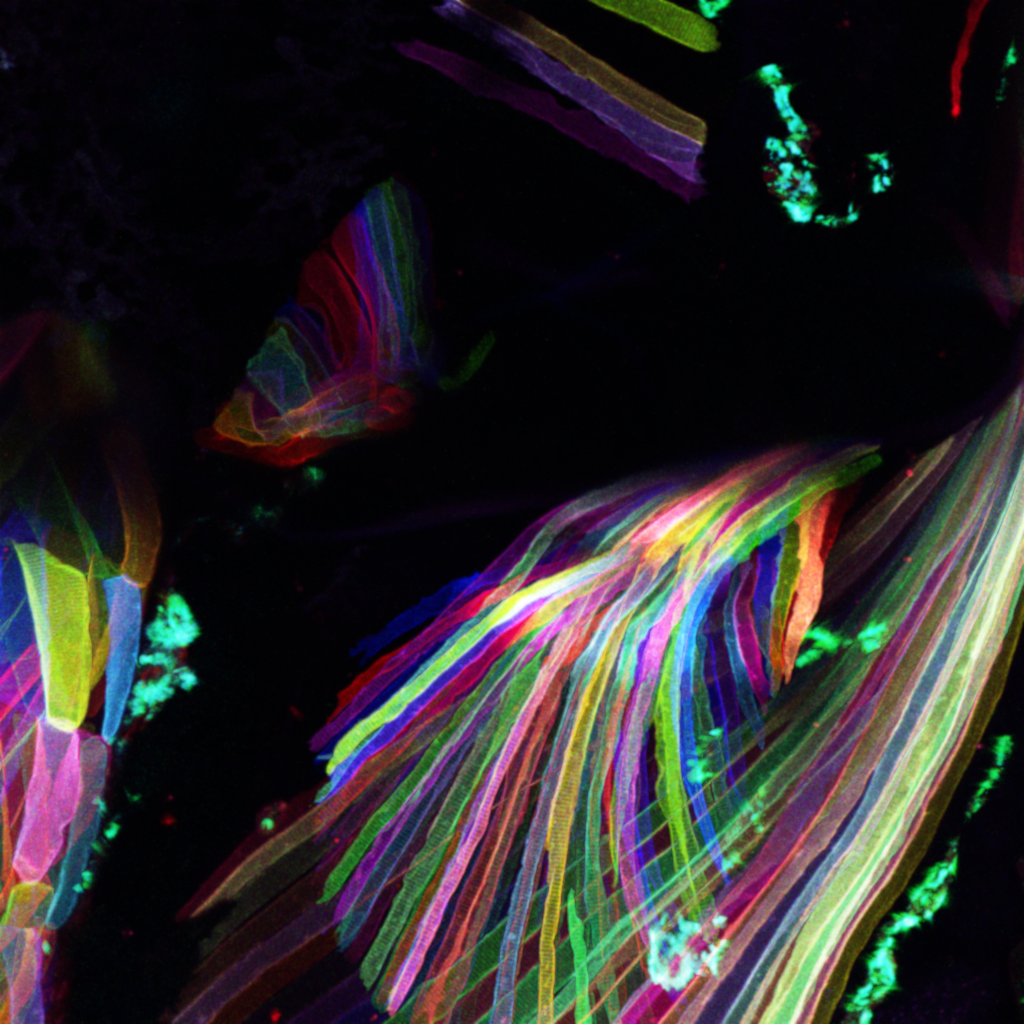

Supplement: Supplementary file 13 — Source data Fig. 4 [file 44318_2024_136_MOESM13_ESM.zip › Figure 4C/palmuscle-Multi-10 dpf-28.tif]

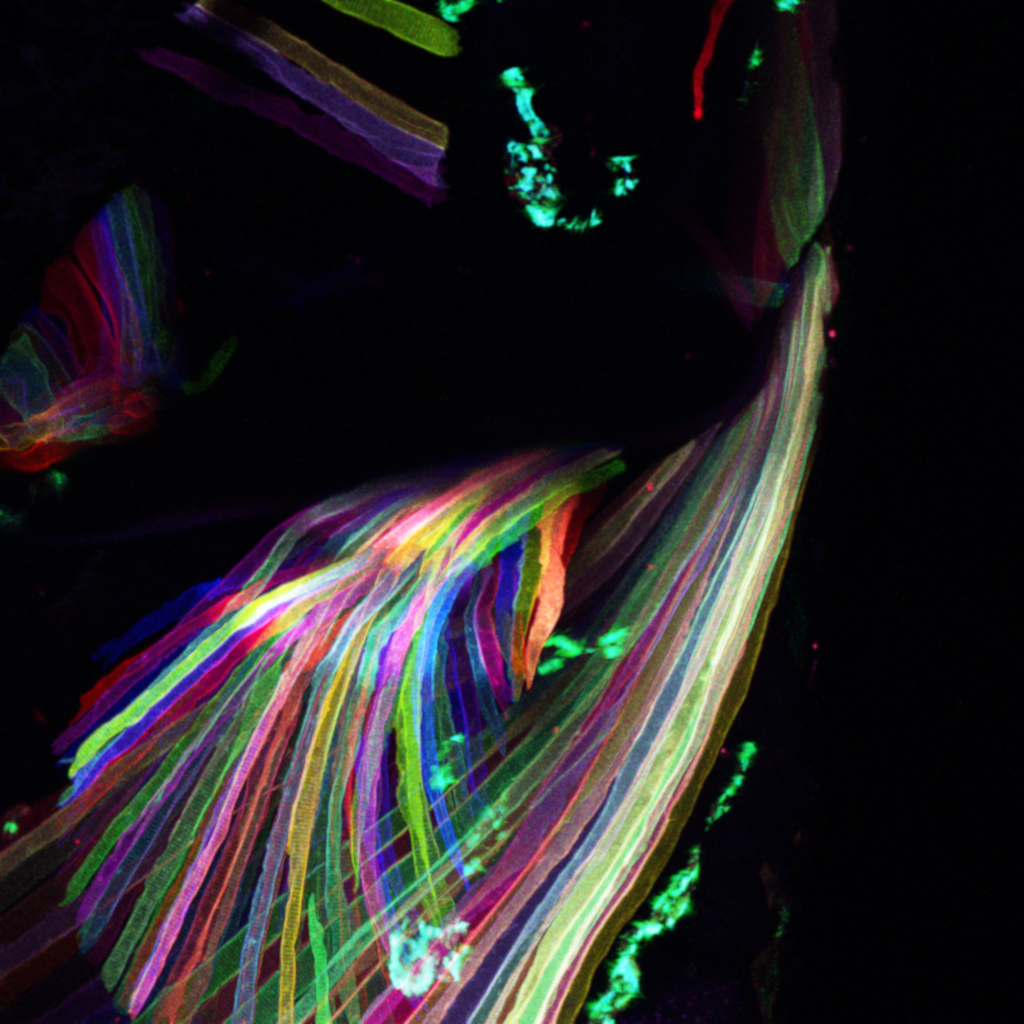

Supplement: Supplementary file 13 — Source data Fig. 4 [file 44318_2024_136_MOESM13_ESM.zip › Figure 4C/palmuscle-Multi-10 dpf-29.tif]

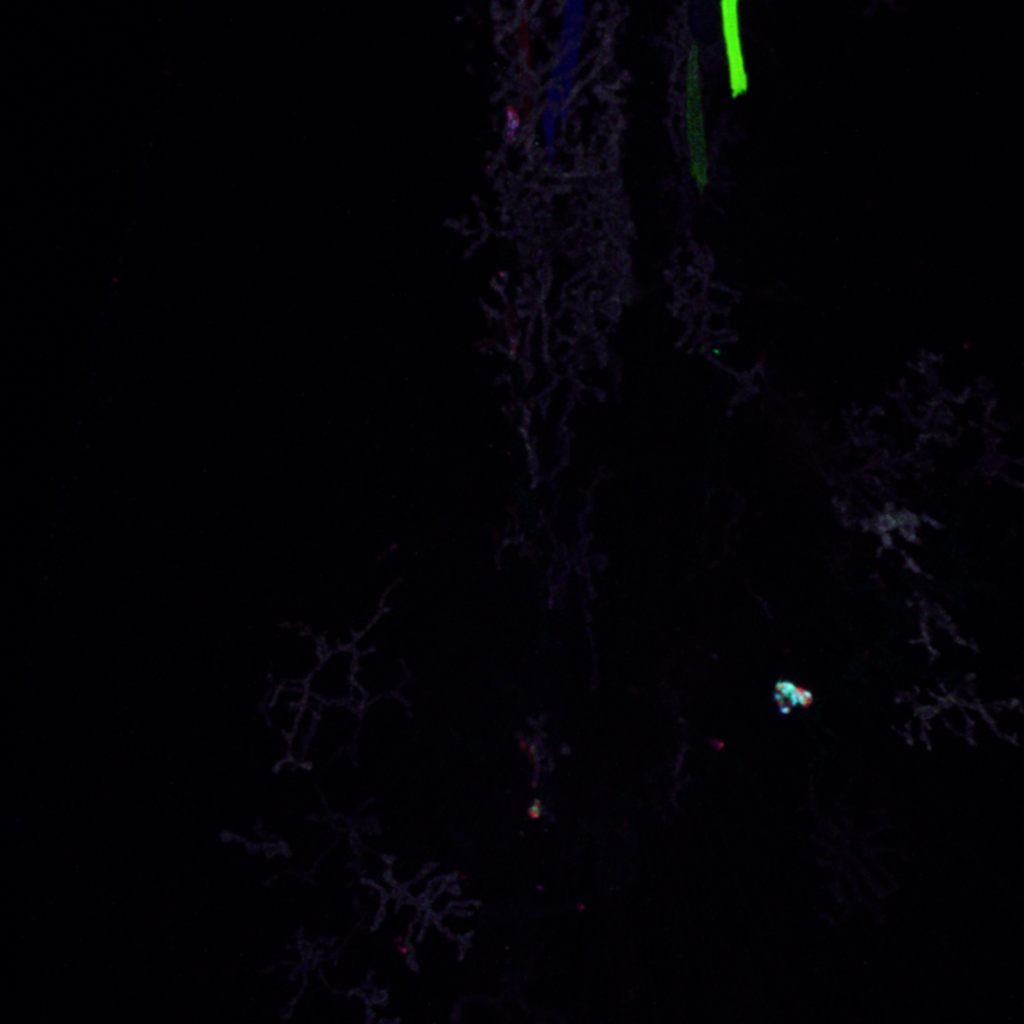

Supplement: Supplementary file 13 — Source data Fig. 4 [file 44318_2024_136_MOESM13_ESM.zip › Figure 4C/palmuscle-Multi-10 dpf-3.tif]

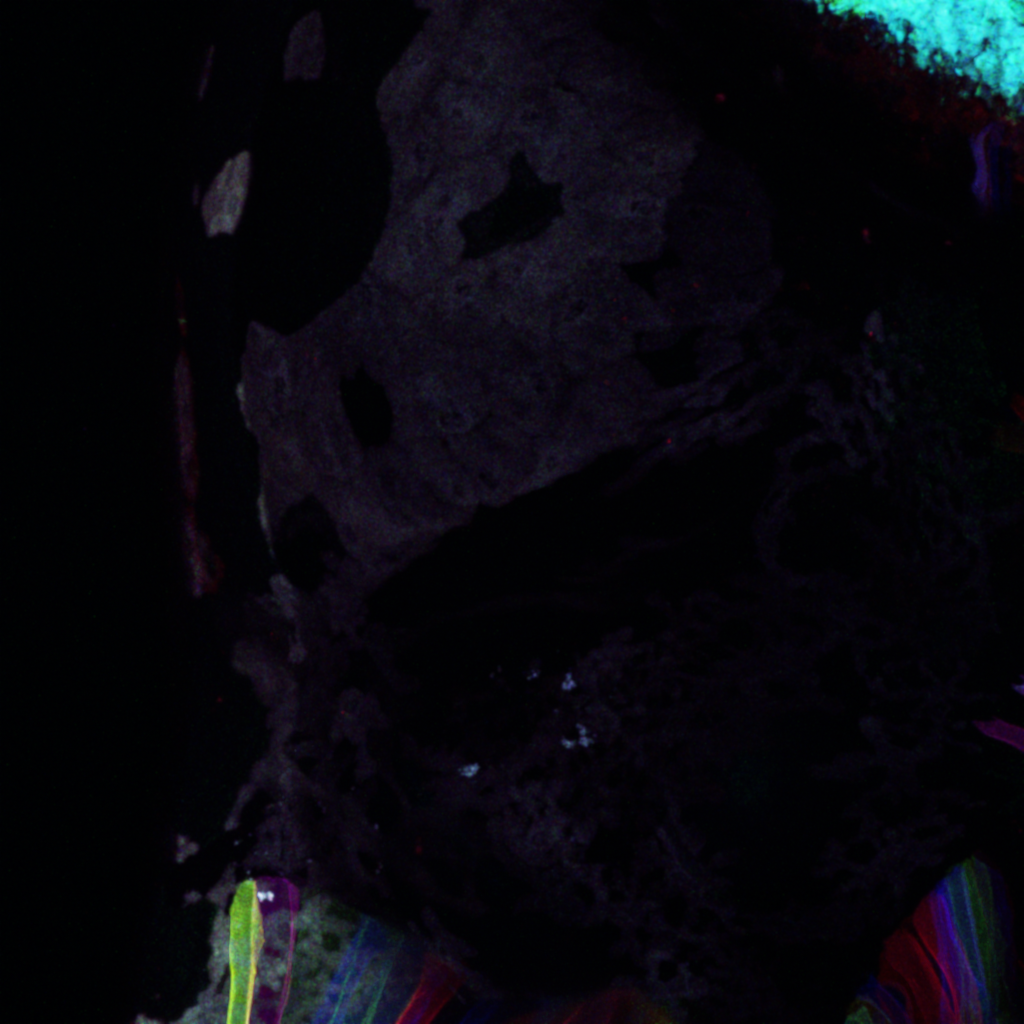

Supplement: Supplementary file 13 — Source data Fig. 4 [file 44318_2024_136_MOESM13_ESM.zip › Figure 4C/palmuscle-Multi-10 dpf-30.tif]

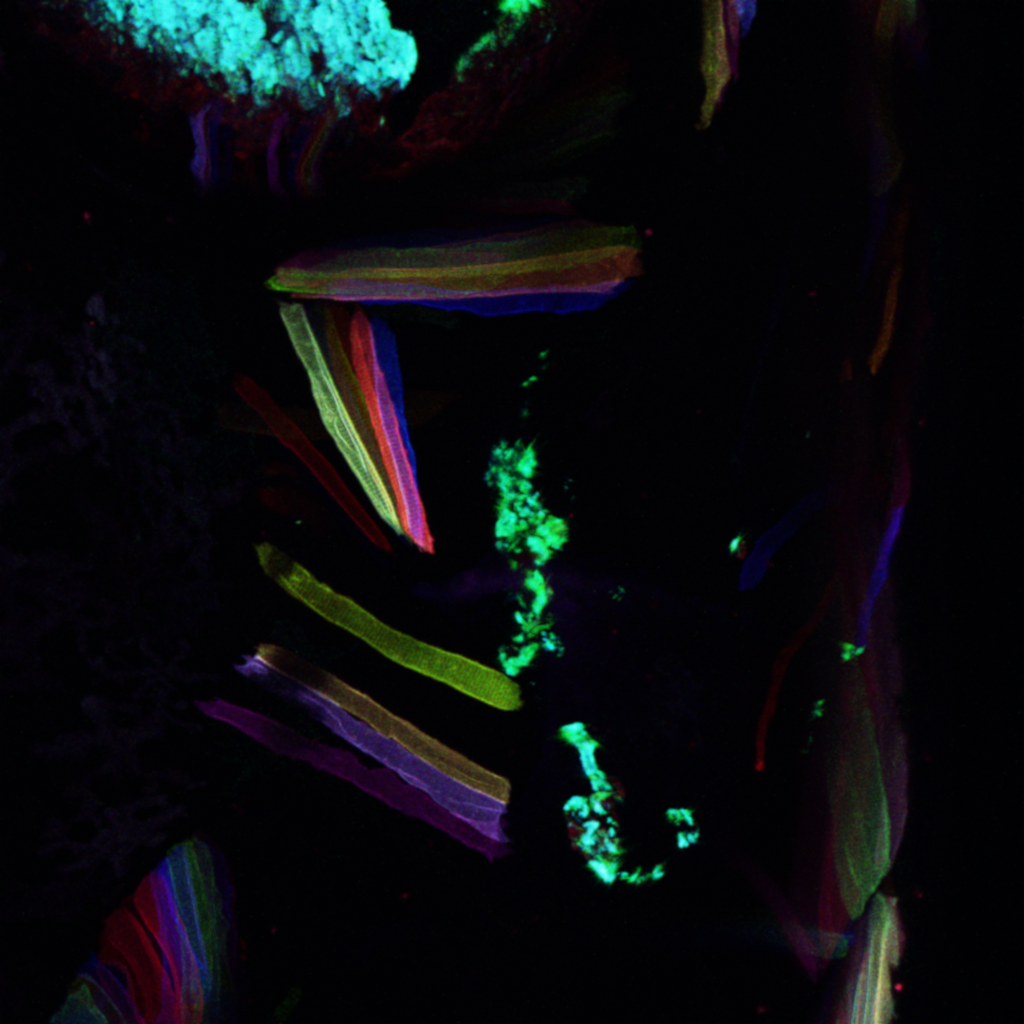

Supplement: Supplementary file 13 — Source data Fig. 4 [file 44318_2024_136_MOESM13_ESM.zip › Figure 4C/palmuscle-Multi-10 dpf-31.tif]

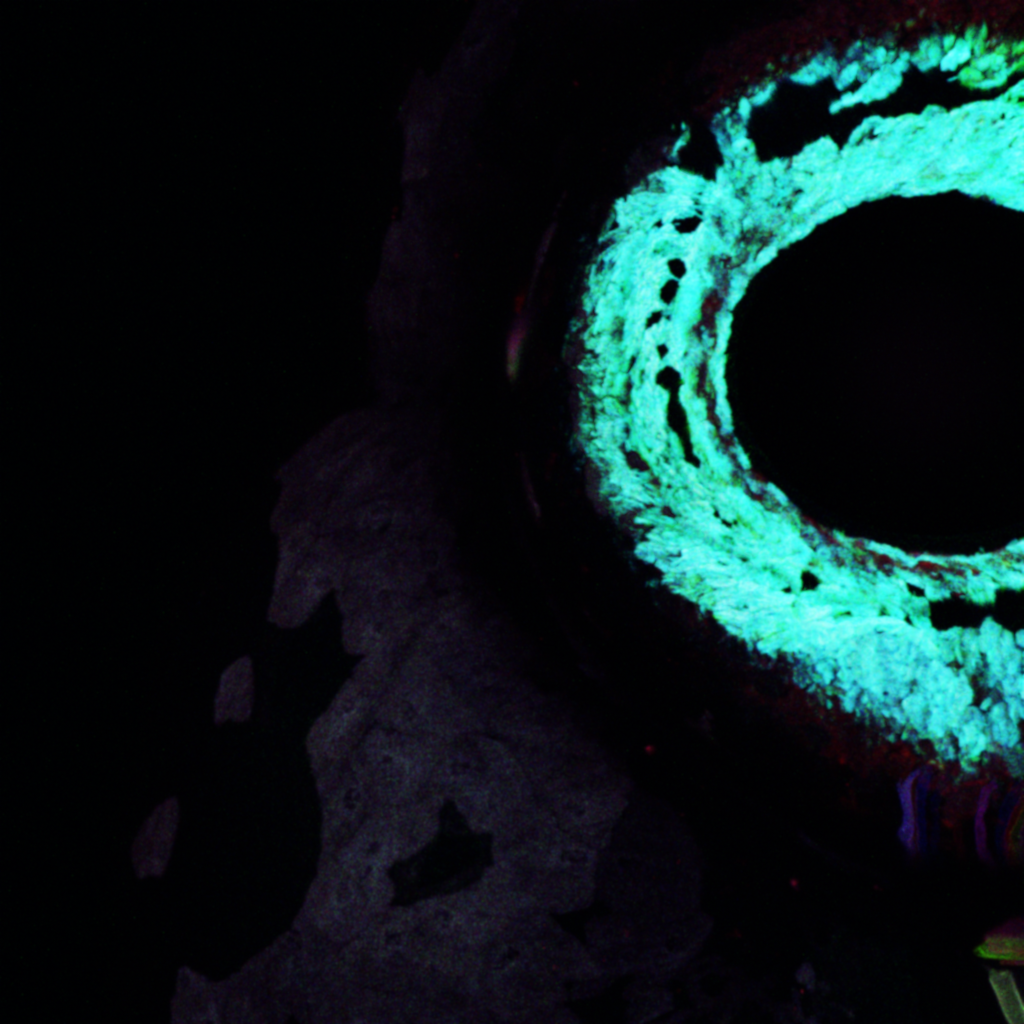

Supplement: Supplementary file 13 — Source data Fig. 4 [file 44318_2024_136_MOESM13_ESM.zip › Figure 4C/palmuscle-Multi-10 dpf-32.tif]

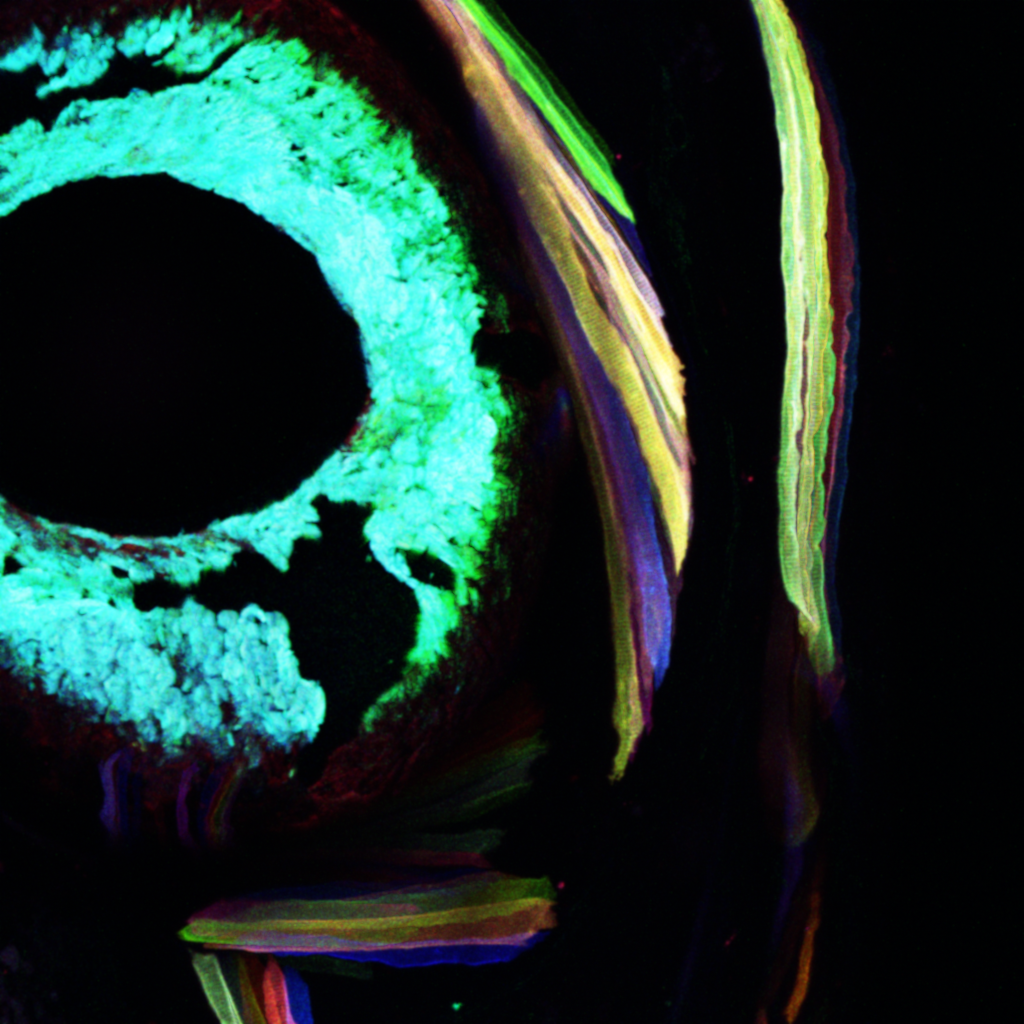

Supplement: Supplementary file 13 — Source data Fig. 4 [file 44318_2024_136_MOESM13_ESM.zip › Figure 4C/palmuscle-Multi-10 dpf-33.tif]

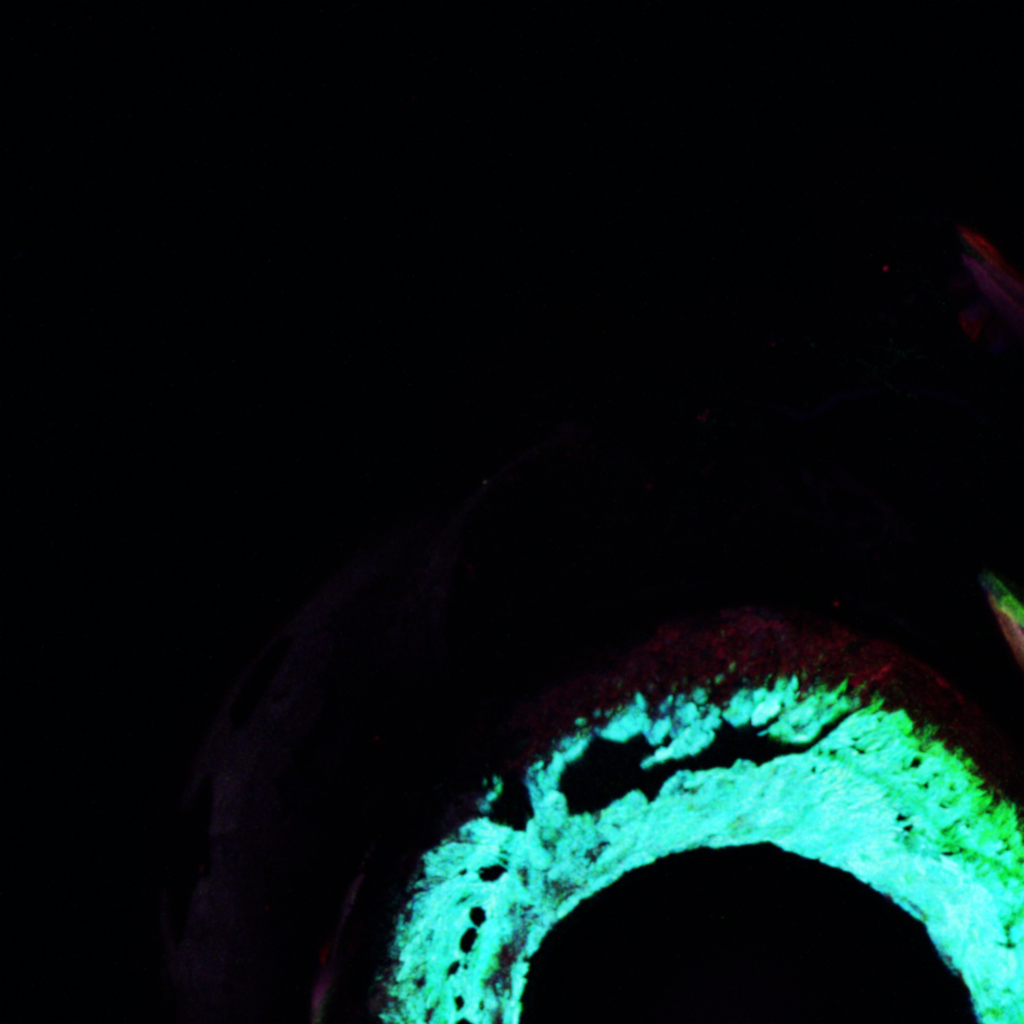

Supplement: Supplementary file 13 — Source data Fig. 4 [file 44318_2024_136_MOESM13_ESM.zip › Figure 4C/palmuscle-Multi-10 dpf-34.tif]

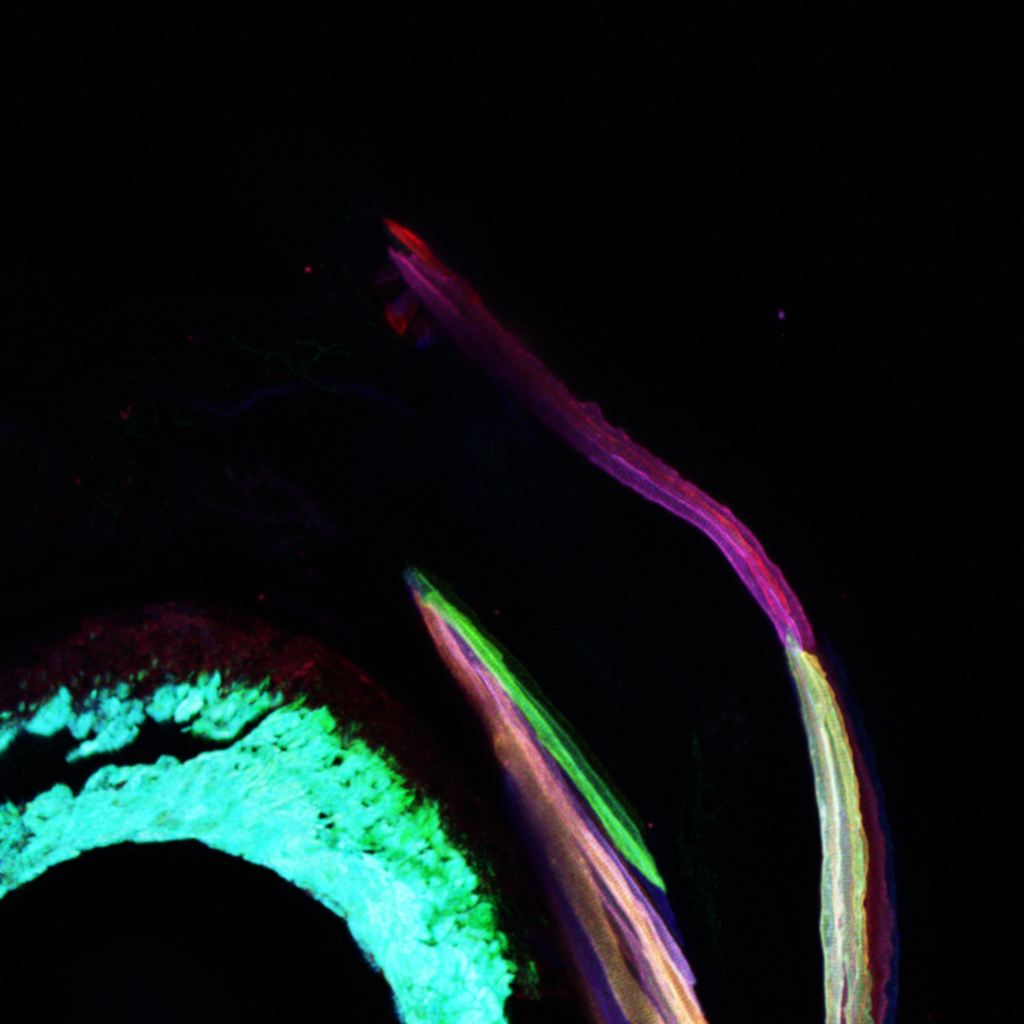

Supplement: Supplementary file 13 — Source data Fig. 4 [file 44318_2024_136_MOESM13_ESM.zip › Figure 4C/palmuscle-Multi-10 dpf-35.tif]

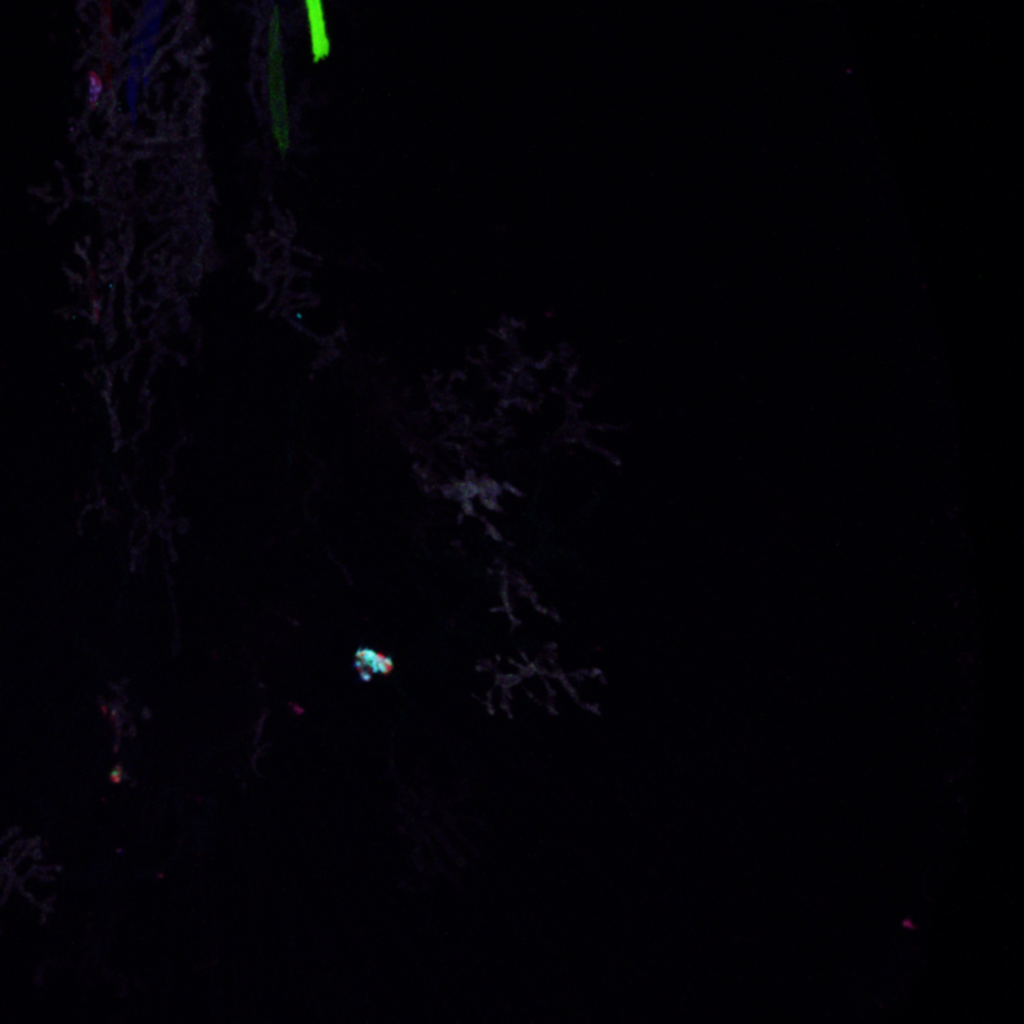

Supplement: Supplementary file 13 — Source data Fig. 4 [file 44318_2024_136_MOESM13_ESM.zip › Figure 4C/palmuscle-Multi-10 dpf-4.tif]

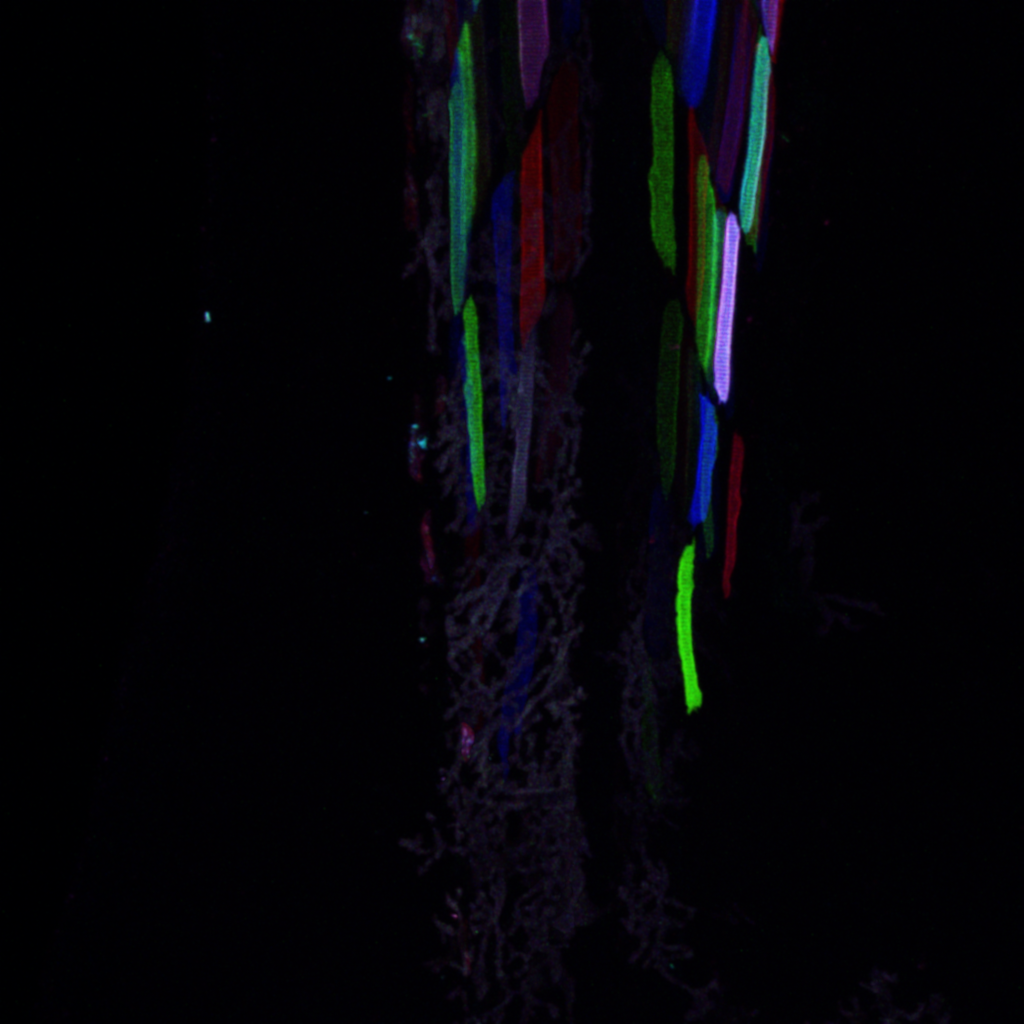

Supplement: Supplementary file 13 — Source data Fig. 4 [file 44318_2024_136_MOESM13_ESM.zip › Figure 4C/palmuscle-Multi-10 dpf-5.tif]

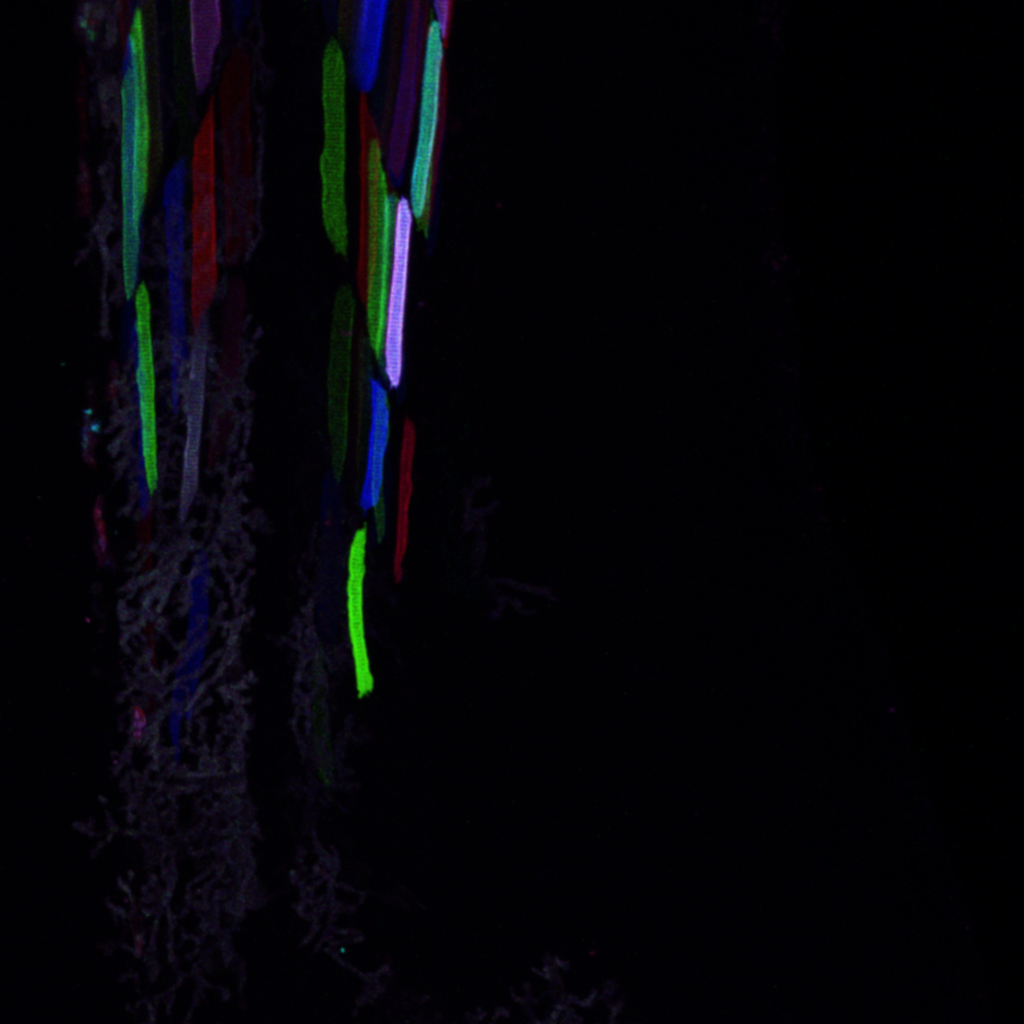

Supplement: Supplementary file 13 — Source data Fig. 4 [file 44318_2024_136_MOESM13_ESM.zip › Figure 4C/palmuscle-Multi-10 dpf-6.tif]

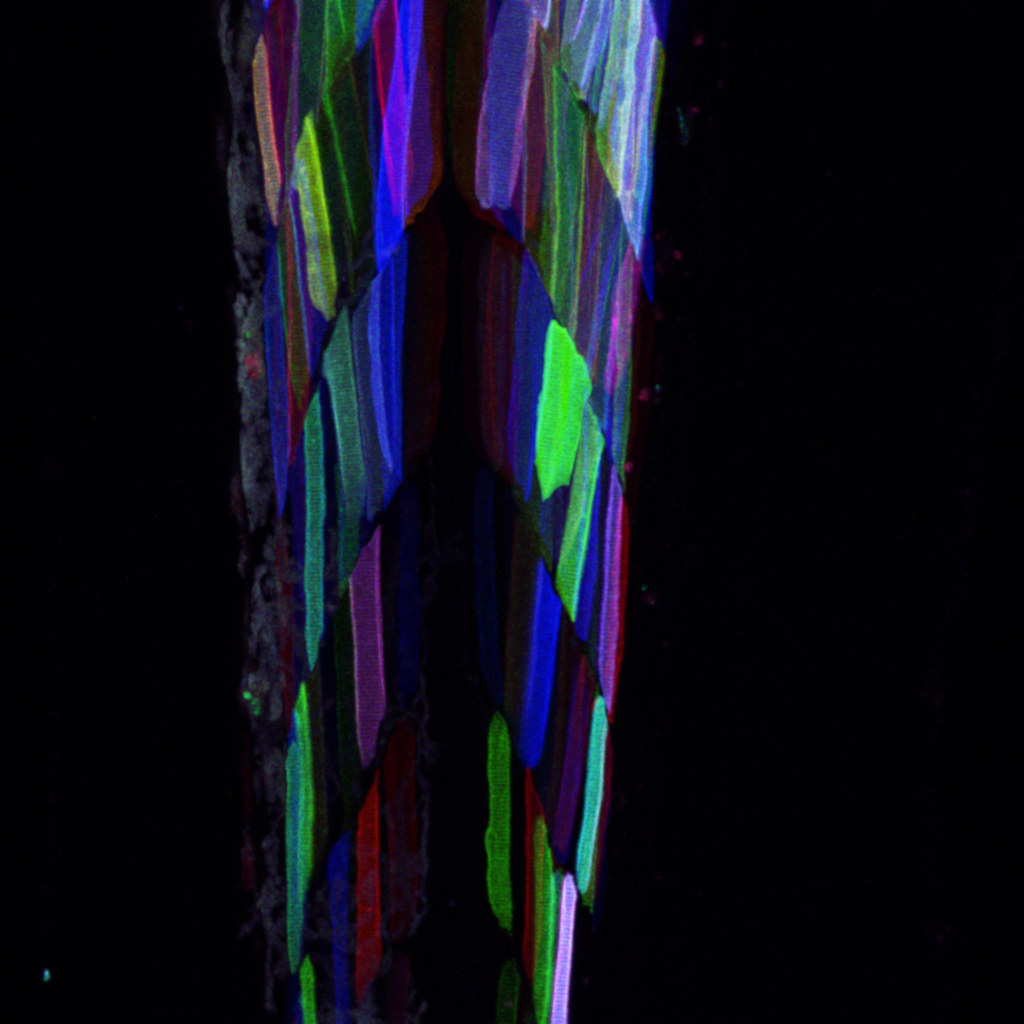

Supplement: Supplementary file 13 — Source data Fig. 4 [file 44318_2024_136_MOESM13_ESM.zip › Figure 4C/palmuscle-Multi-10 dpf-7.tif]

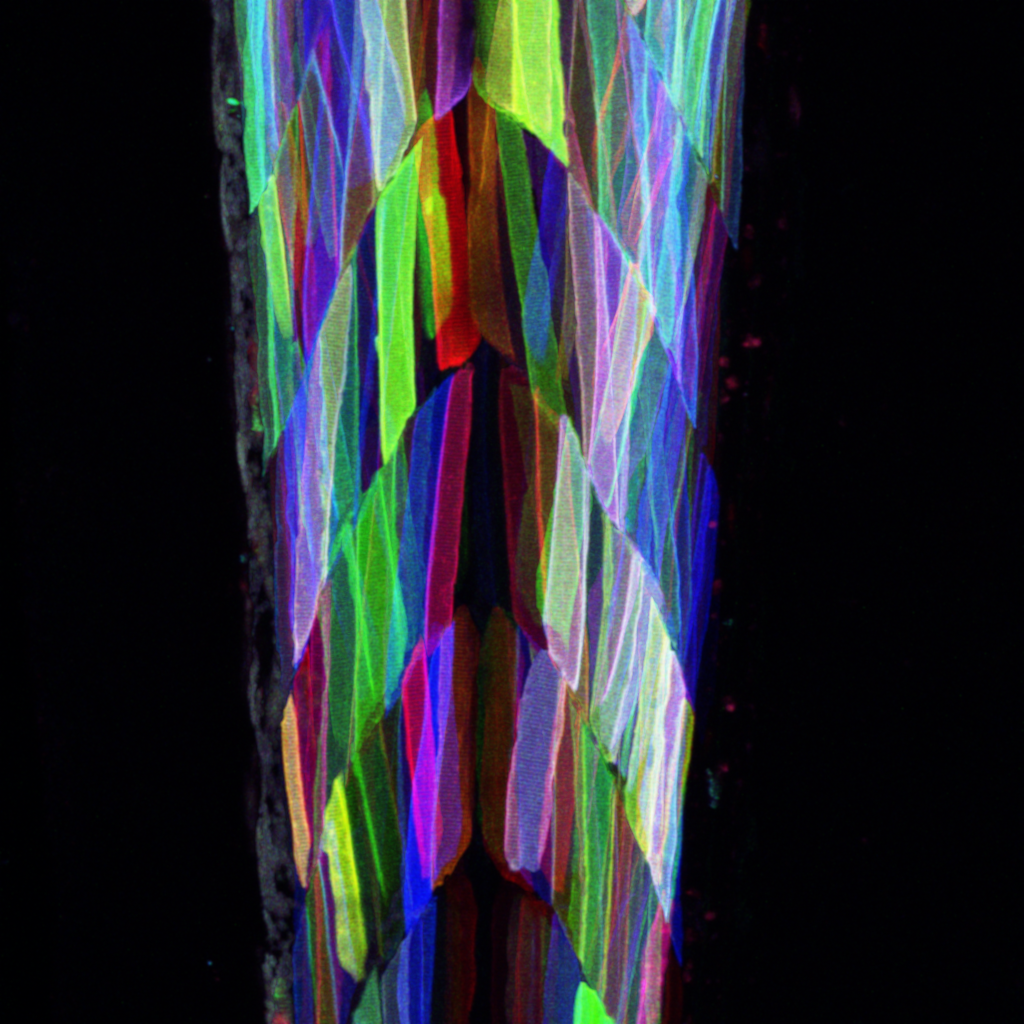

Supplement: Supplementary file 13 — Source data Fig. 4 [file 44318_2024_136_MOESM13_ESM.zip › Figure 4C/palmuscle-Multi-10 dpf-8.tif]

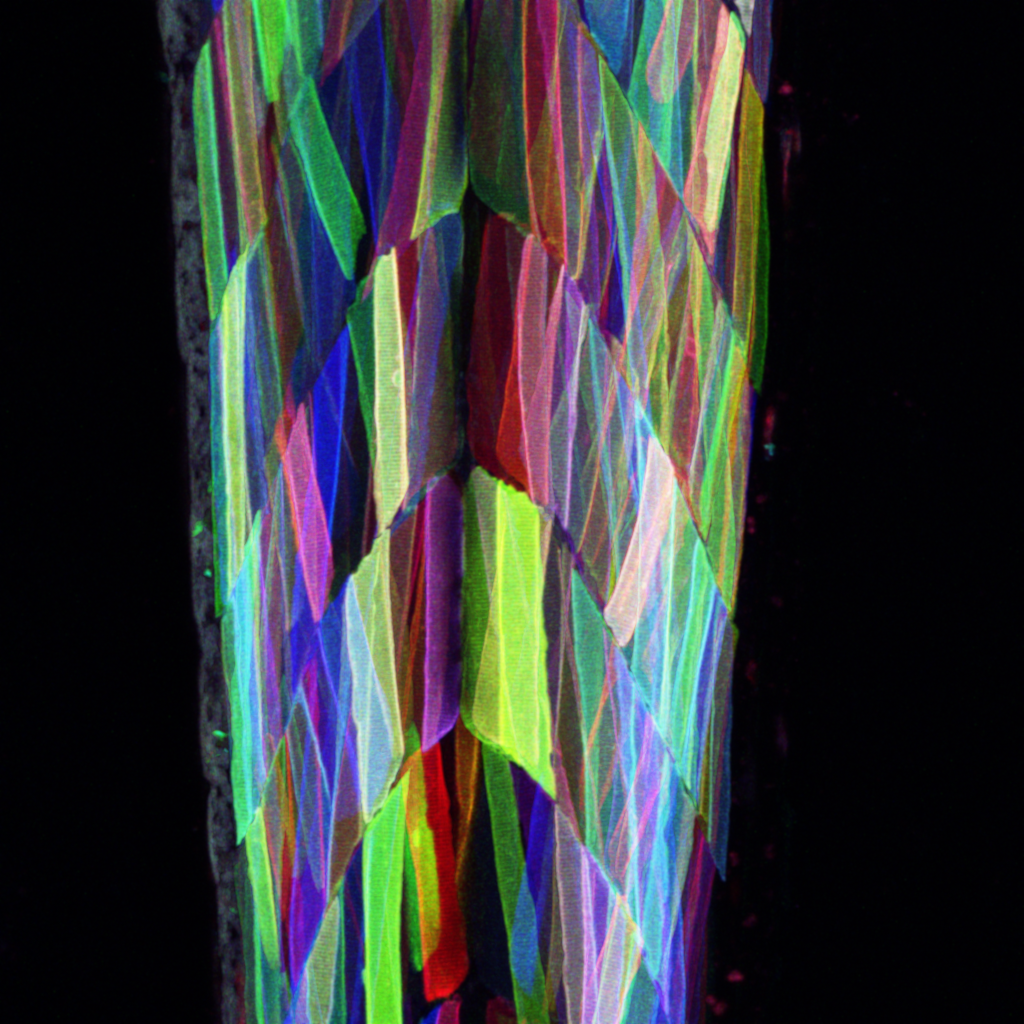

Supplement: Supplementary file 13 — Source data Fig. 4 [file 44318_2024_136_MOESM13_ESM.zip › Figure 4C/palmuscle-Multi-10 dpf-9.tif]

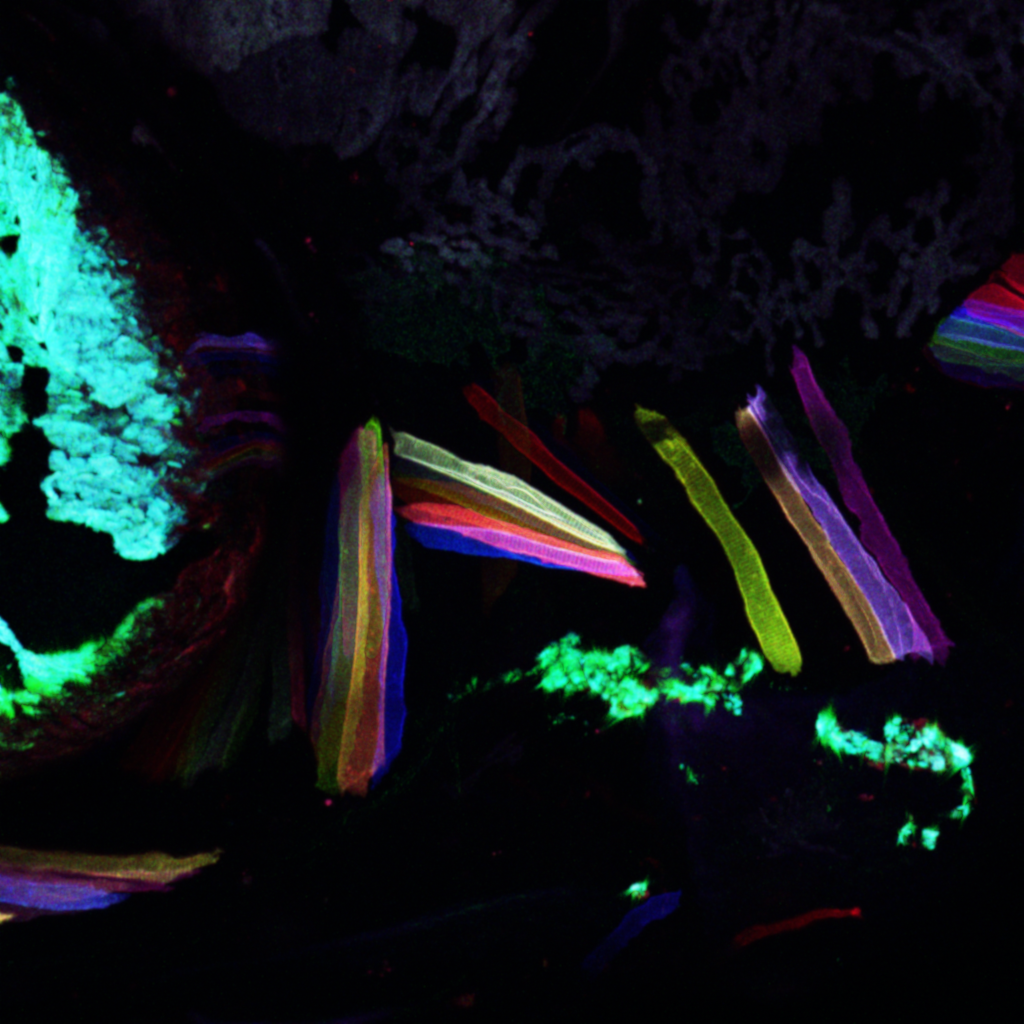

Supplement: Supplementary file 13 — Source data Fig. 4 [file 44318_2024_136_MOESM13_ESM.zip › Figure 4D/palmuscle-Multi-craniofacial.tif]

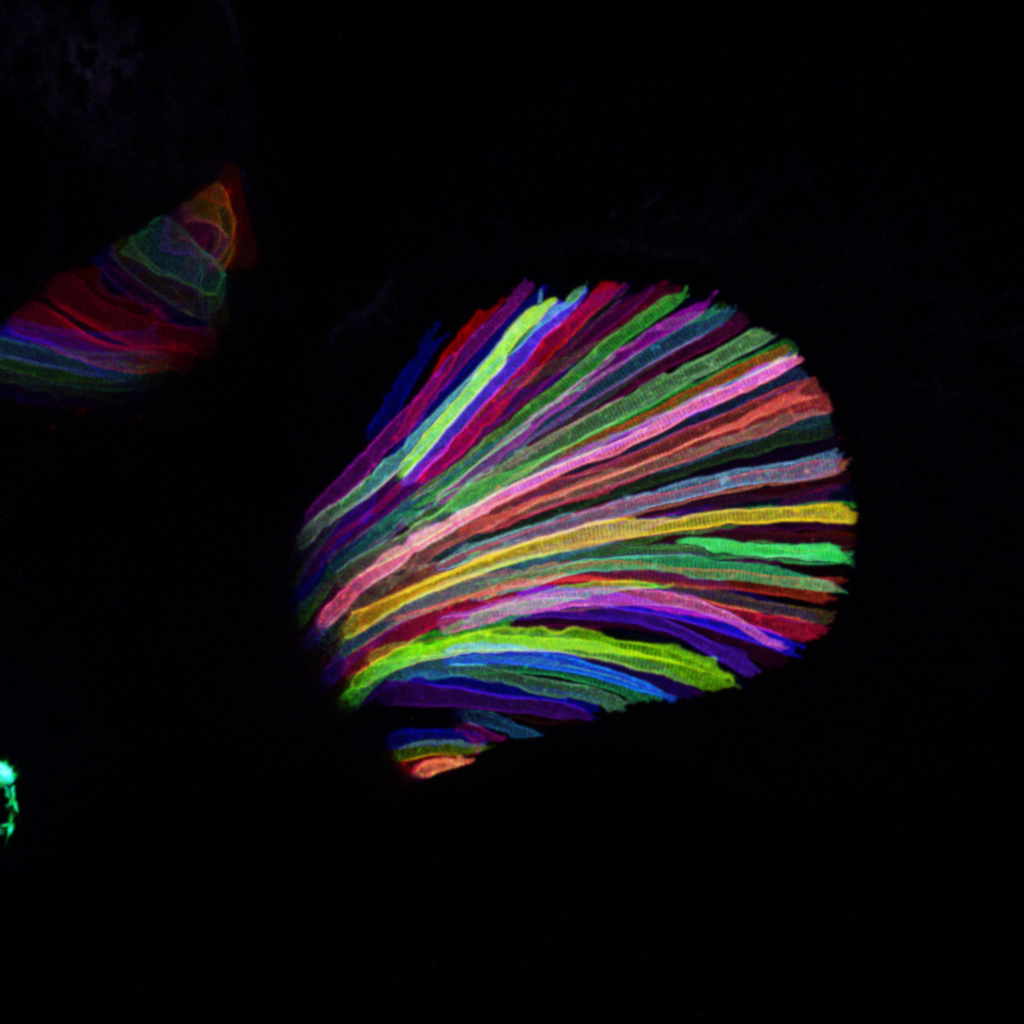

Supplement: Supplementary file 13 — Source data Fig. 4 [file 44318_2024_136_MOESM13_ESM.zip › Figure 4D/palmuscle-Multi-pectoral fin.tif]

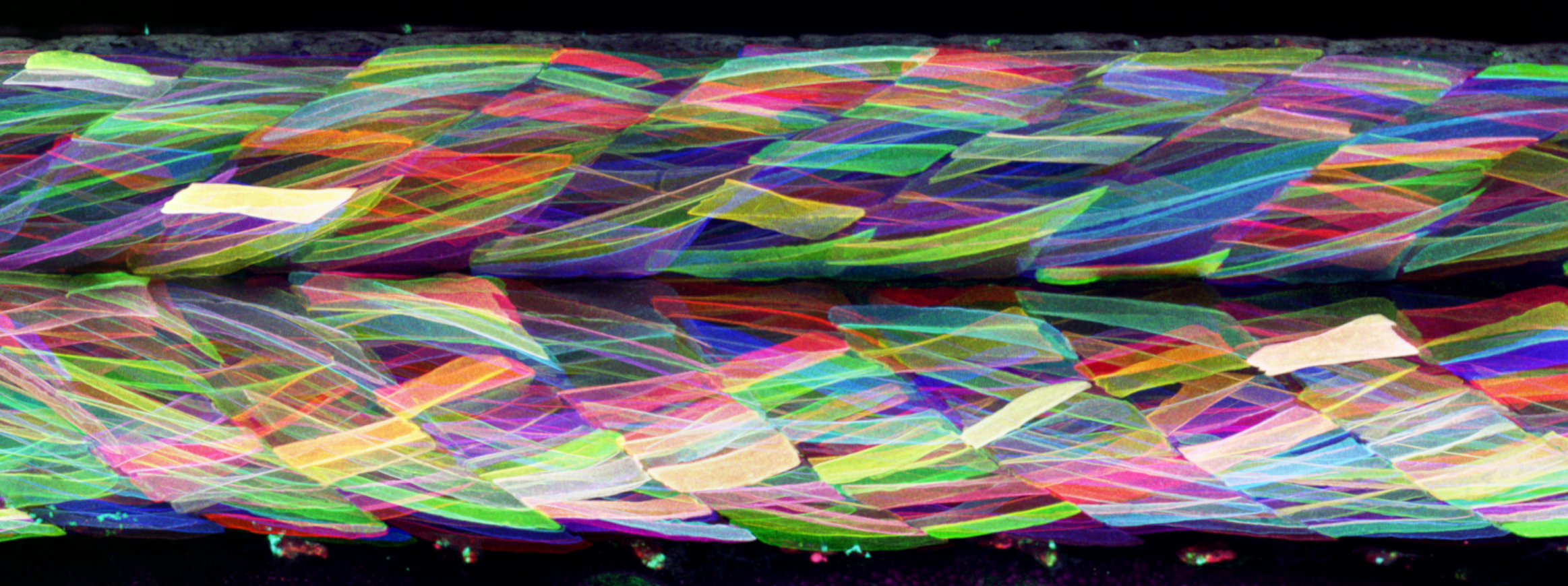

Supplement: Supplementary file 13 — Source data Fig. 4 [file 44318_2024_136_MOESM13_ESM.zip › Figure 4D/palmuscle-Multi-Trunk.tif]

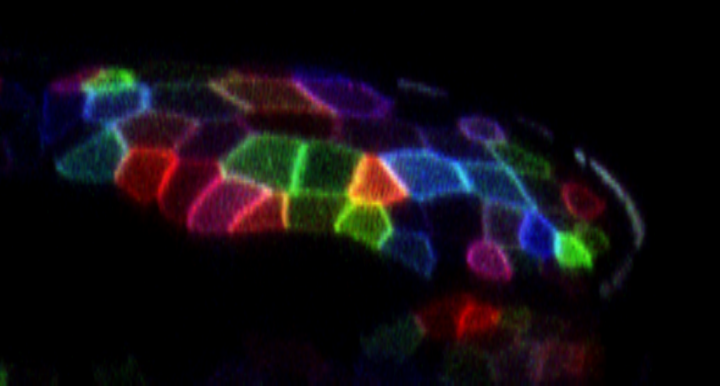

Supplement: Supplementary file 13 — Source data Fig. 4 [file 44318_2024_136_MOESM13_ESM.zip › Figure 4E/palmuscle-Multi-cross-section-dorsal.tif]

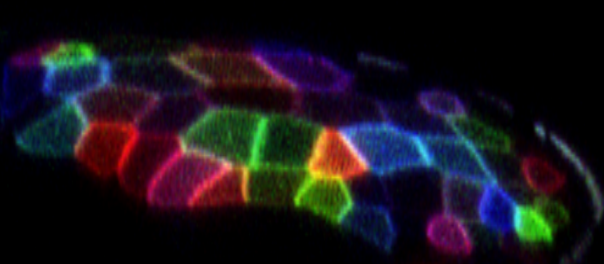

Supplement: Supplementary file 13 — Source data Fig. 4 [file 44318_2024_136_MOESM13_ESM.zip › Figure 4E/palmuscle-Multi-cross-section-dorsal-crop.tif]

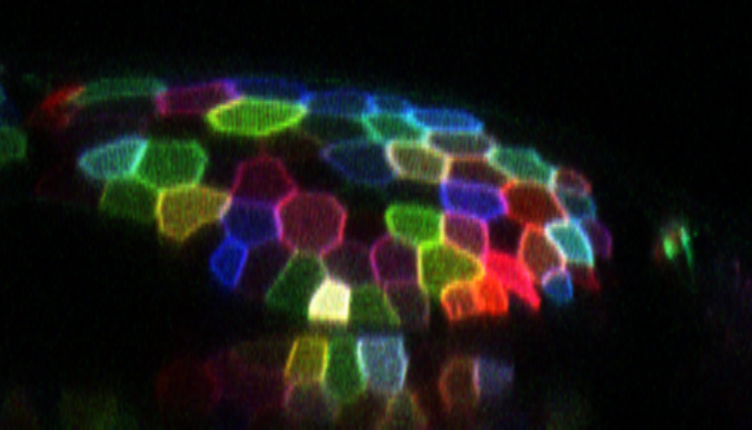

Supplement: Supplementary file 13 — Source data Fig. 4 [file 44318_2024_136_MOESM13_ESM.zip › Figure 4E/palmuscle-Multi-cross-section-ventral.tif]

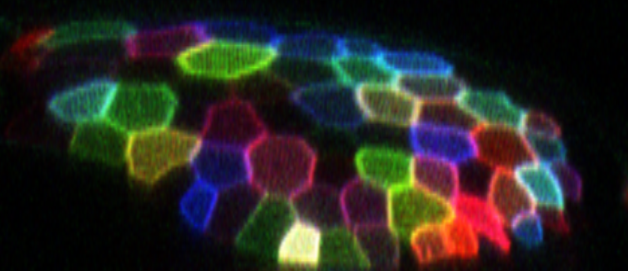

Supplement: Supplementary file 13 — Source data Fig. 4 [file 44318_2024_136_MOESM13_ESM.zip › Figure 4E/palmuscle-Multi-cross-section-ventral-crop.tif]

## Slide 1
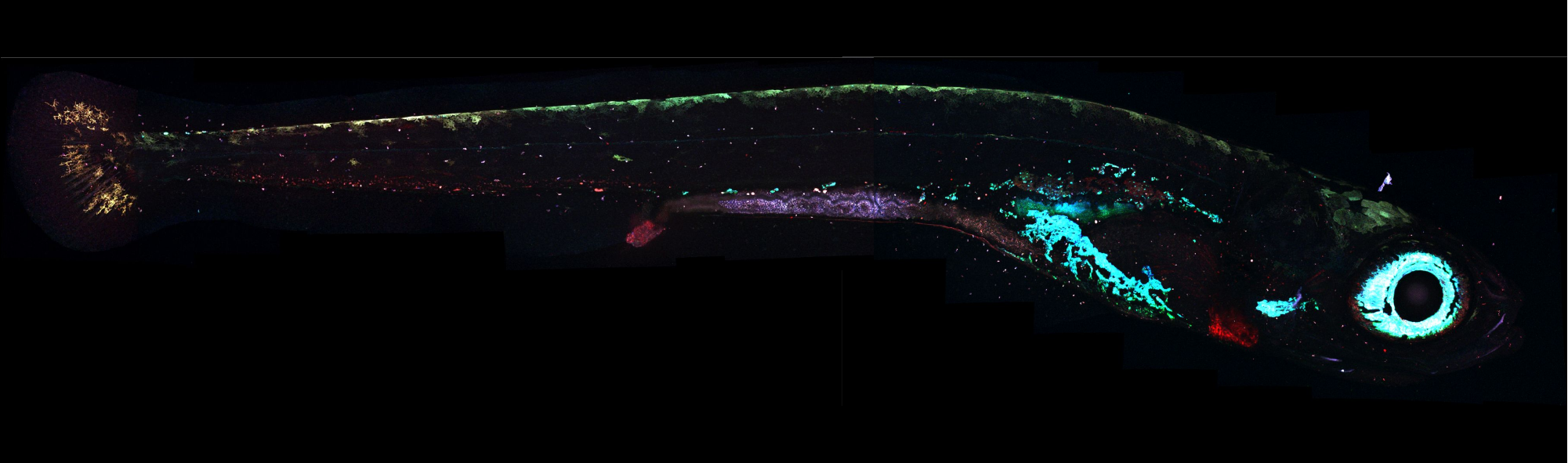

## Slide 2
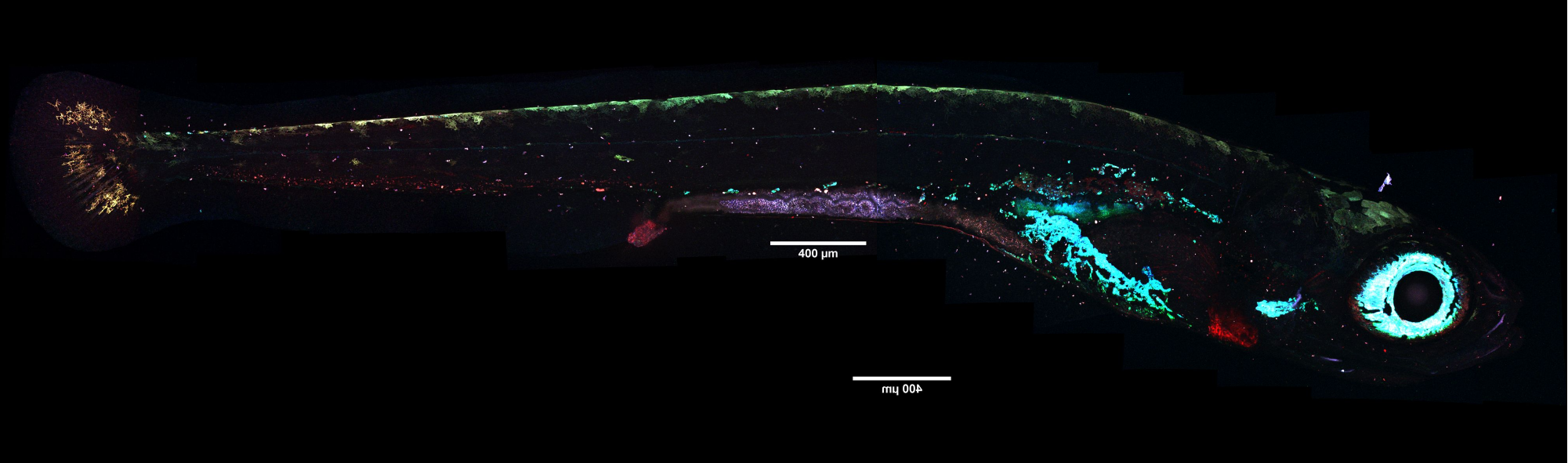

Supplement: Supplementary file 13 — Source data Fig. 4 [file 44318_2024_136_MOESM13_ESM.zip › Figure 4J/Manual stitching.pptx]

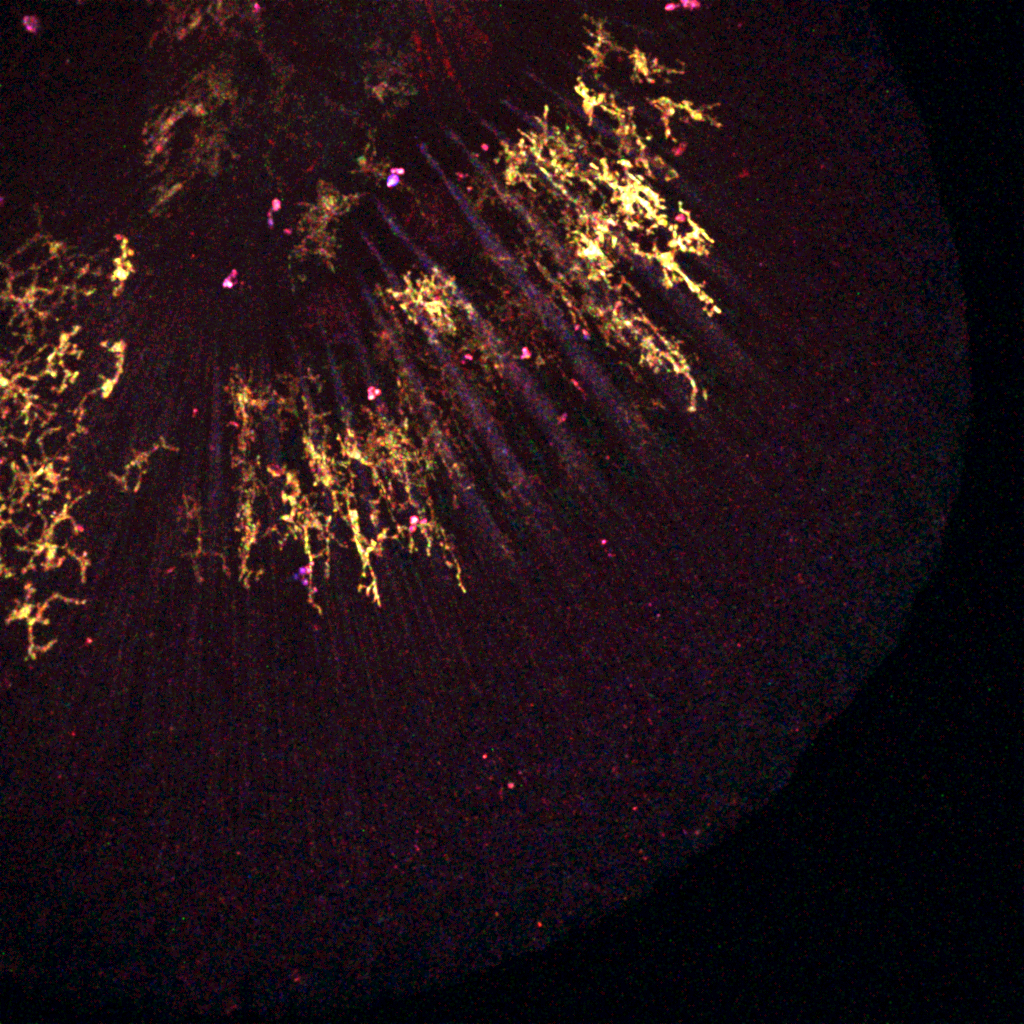

Supplement: Supplementary file 13 — Source data Fig. 4 [file 44318_2024_136_MOESM13_ESM.zip › Figure 4J/palmuscle-Multi-No treatement-14 dpf-1.tif]

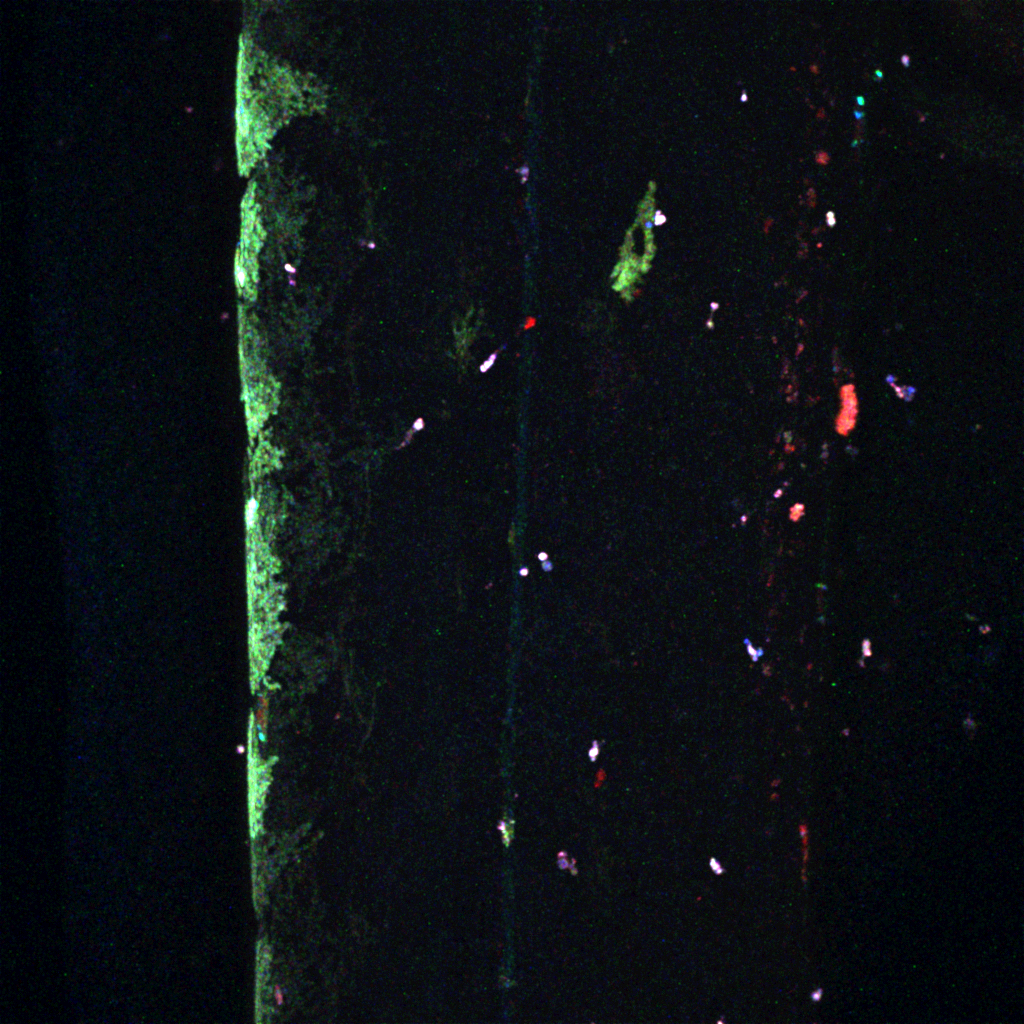

Supplement: Supplementary file 13 — Source data Fig. 4 [file 44318_2024_136_MOESM13_ESM.zip › Figure 4J/palmuscle-Multi-No treatement-14 dpf-10.tif]
