# Supplementary figures and images for: Whole-body replacement of larval myofibers generates permanent adult myofibers in zebrafish (part 3 of 4)
Source: EMBO J. 2024 Jun 5;43(15):2. doi: 10.1038/s44318-024-00136-y (PMC11294464; doi:10.1038/s44318-024-00136-y)

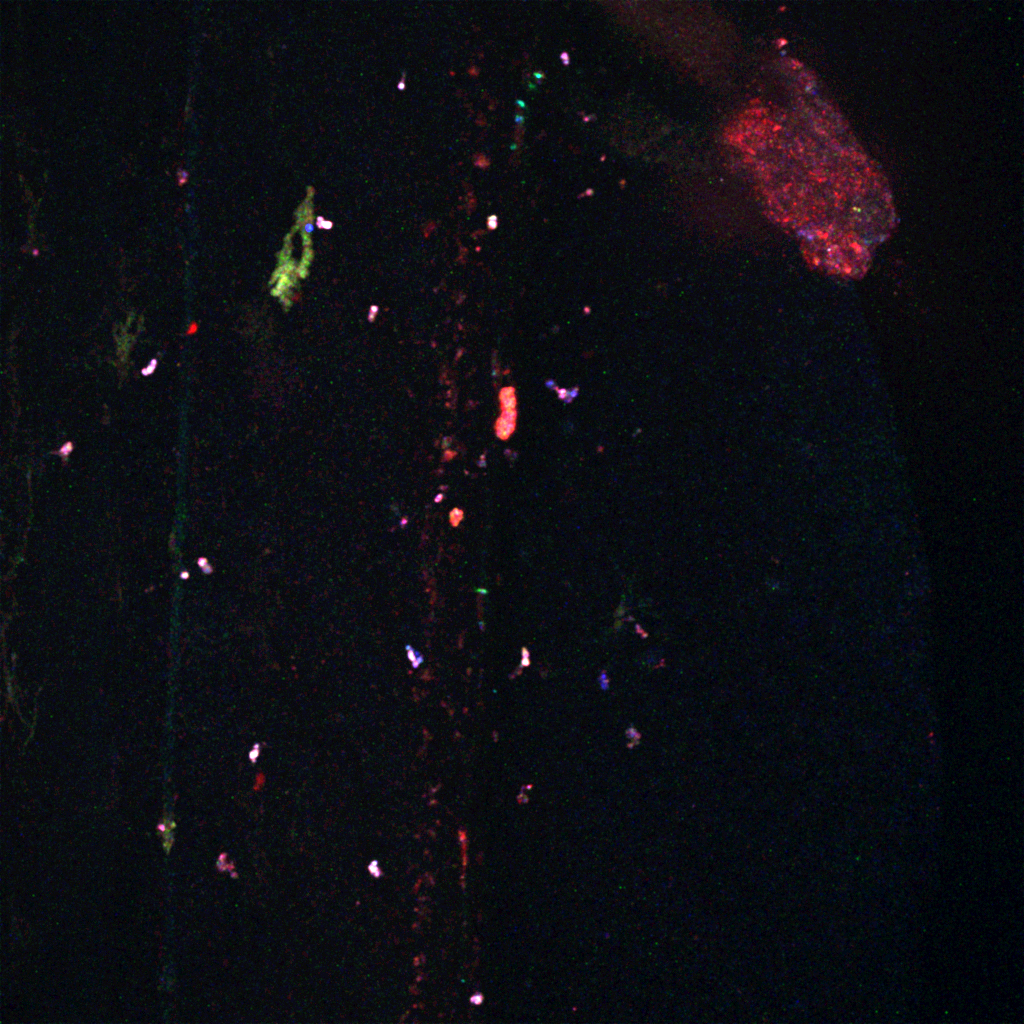

Supplement: Supplementary file 13 — Source data Fig. 4 [file 44318_2024_136_MOESM13_ESM.zip › Figure 4J/palmuscle-Multi-No treatement-14 dpf-11.tif]

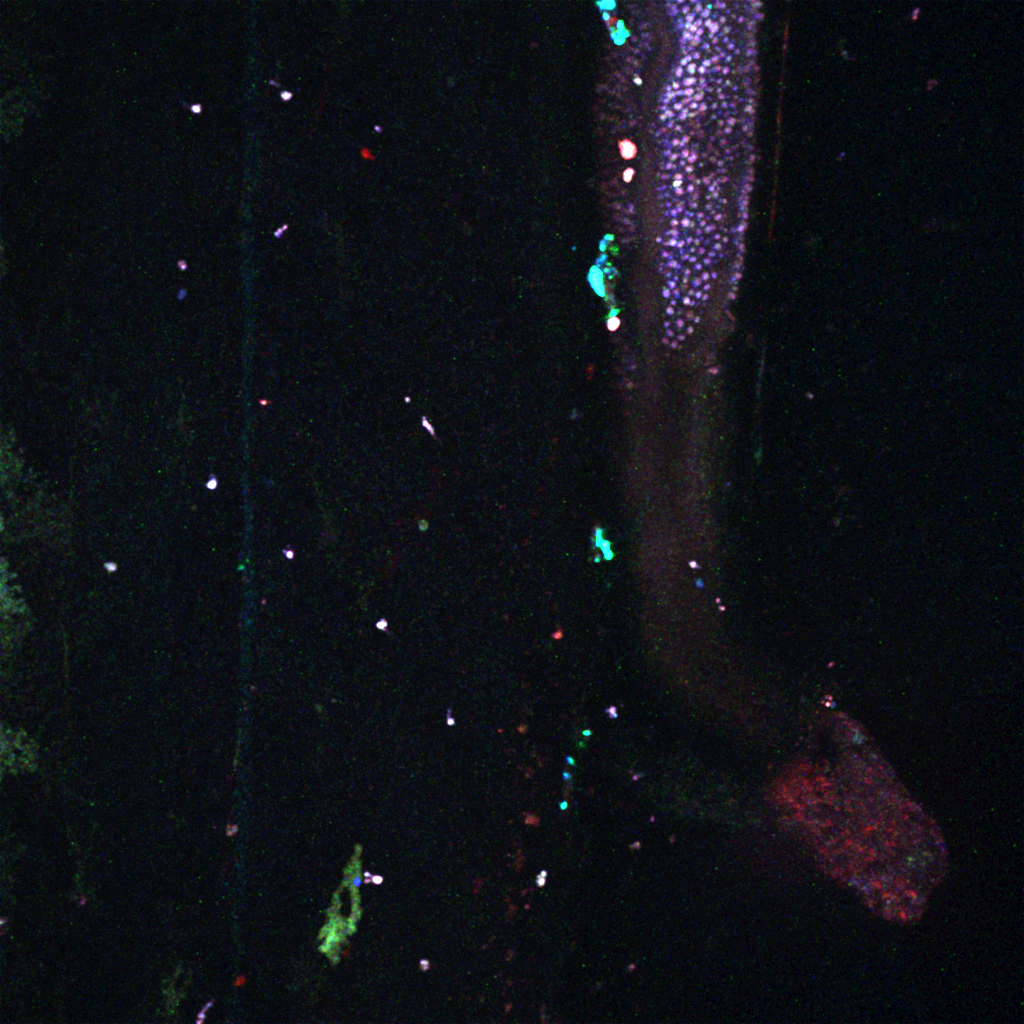

Supplement: Supplementary file 13 — Source data Fig. 4 [file 44318_2024_136_MOESM13_ESM.zip › Figure 4J/palmuscle-Multi-No treatement-14 dpf-12.tif]

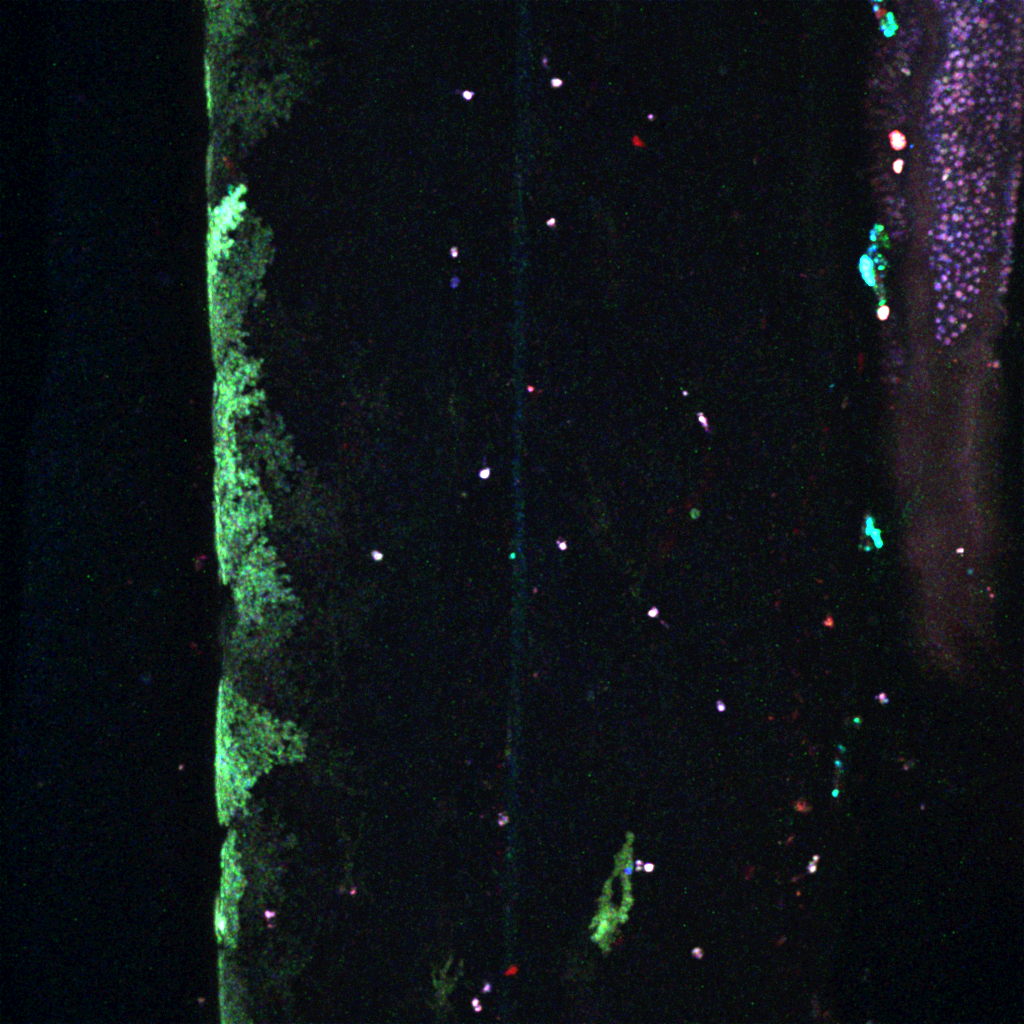

Supplement: Supplementary file 13 — Source data Fig. 4 [file 44318_2024_136_MOESM13_ESM.zip › Figure 4J/palmuscle-Multi-No treatement-14 dpf-13.tif]

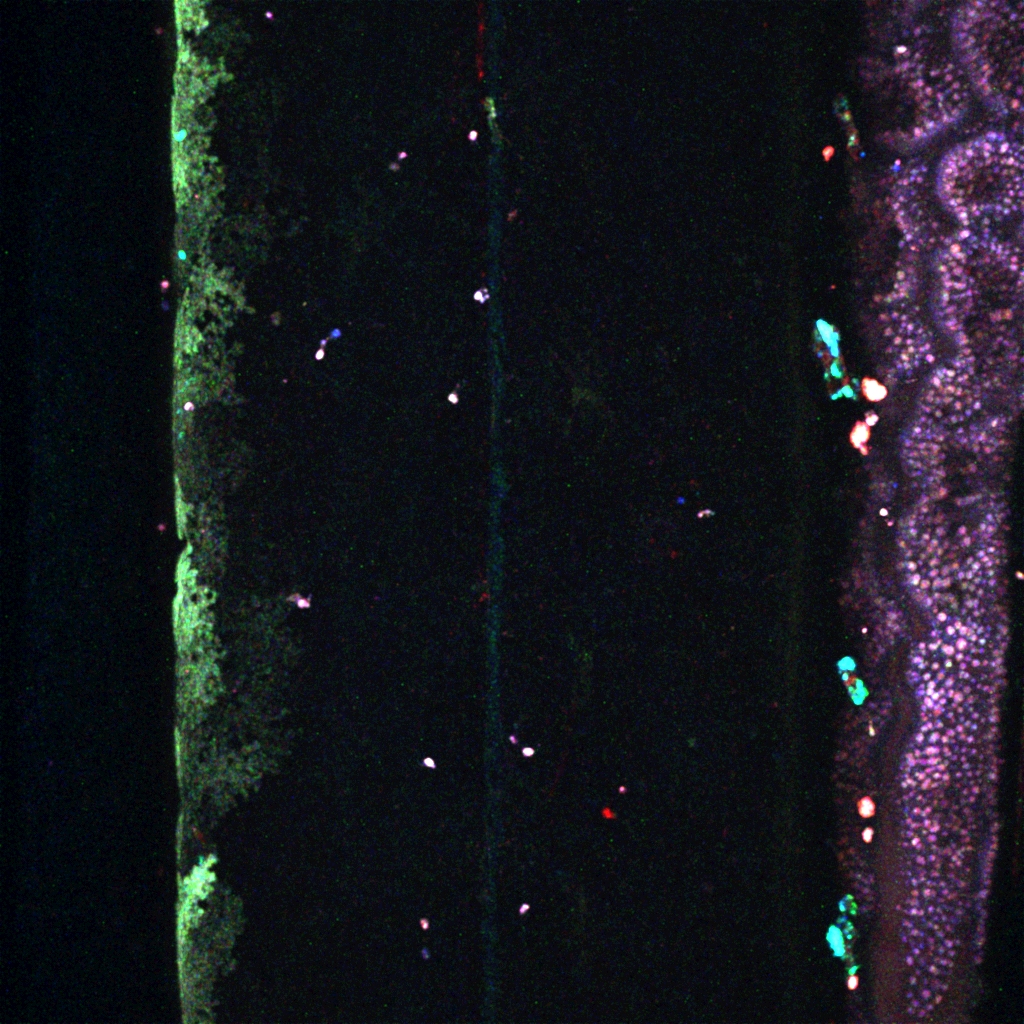

Supplement: Supplementary file 13 — Source data Fig. 4 [file 44318_2024_136_MOESM13_ESM.zip › Figure 4J/palmuscle-Multi-No treatement-14 dpf-14.tif]

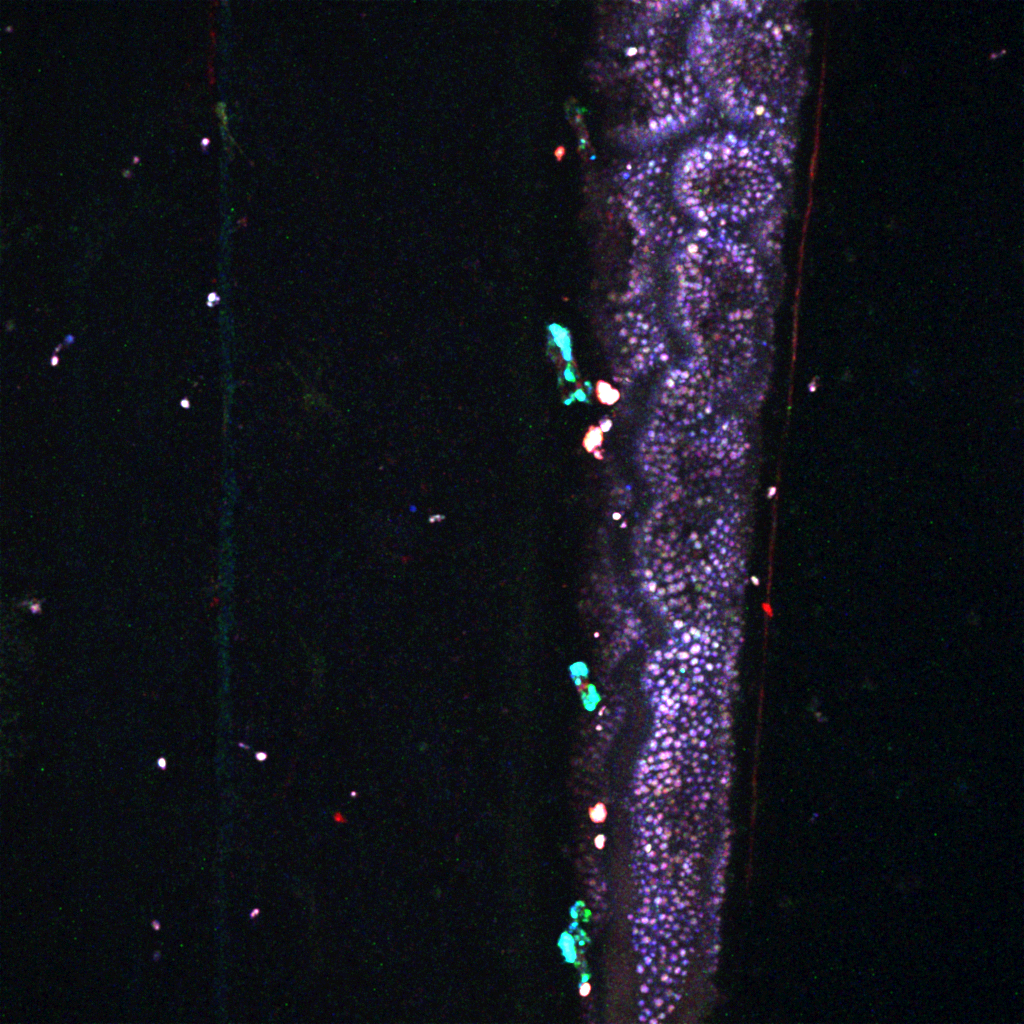

Supplement: Supplementary file 13 — Source data Fig. 4 [file 44318_2024_136_MOESM13_ESM.zip › Figure 4J/palmuscle-Multi-No treatement-14 dpf-15.tif]

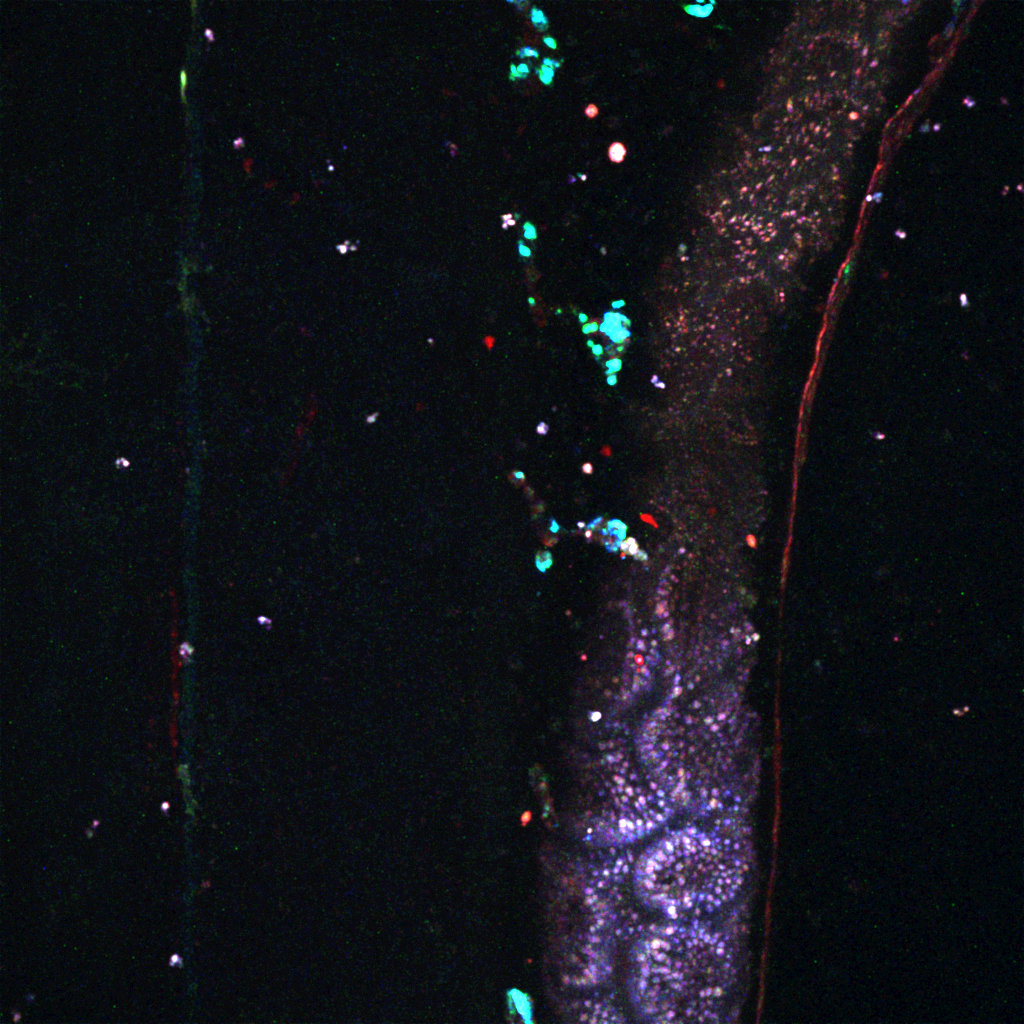

Supplement: Supplementary file 13 — Source data Fig. 4 [file 44318_2024_136_MOESM13_ESM.zip › Figure 4J/palmuscle-Multi-No treatement-14 dpf-16.tif]

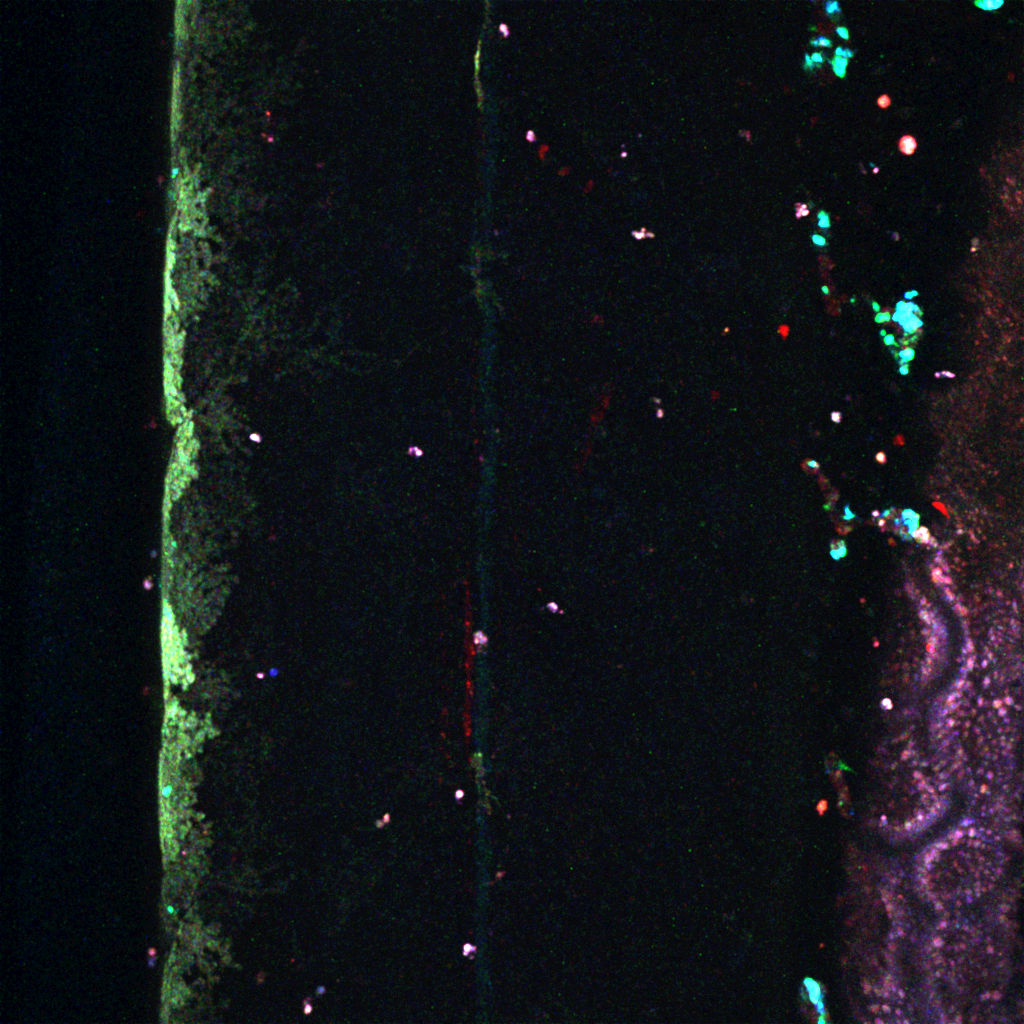

Supplement: Supplementary file 13 — Source data Fig. 4 [file 44318_2024_136_MOESM13_ESM.zip › Figure 4J/palmuscle-Multi-No treatement-14 dpf-17.tif]

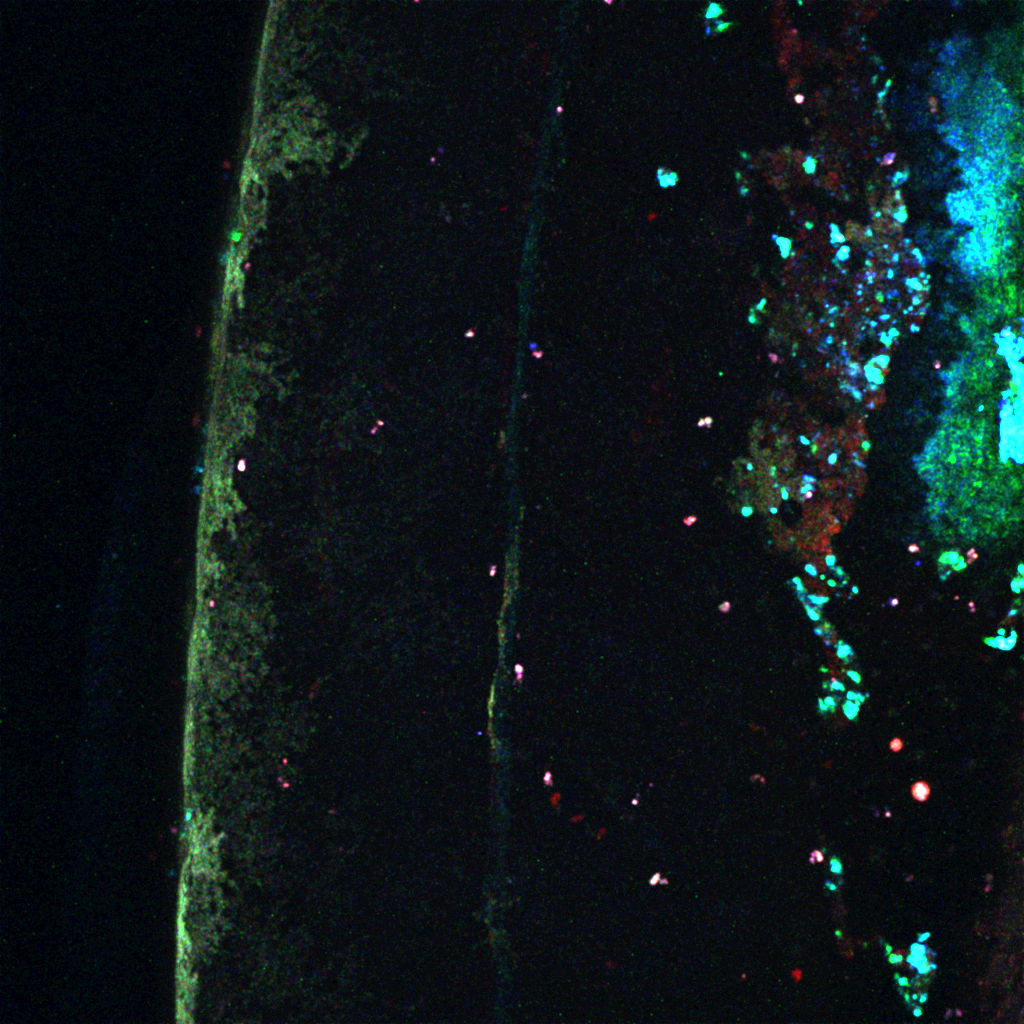

Supplement: Supplementary file 13 — Source data Fig. 4 [file 44318_2024_136_MOESM13_ESM.zip › Figure 4J/palmuscle-Multi-No treatement-14 dpf-18.tif]

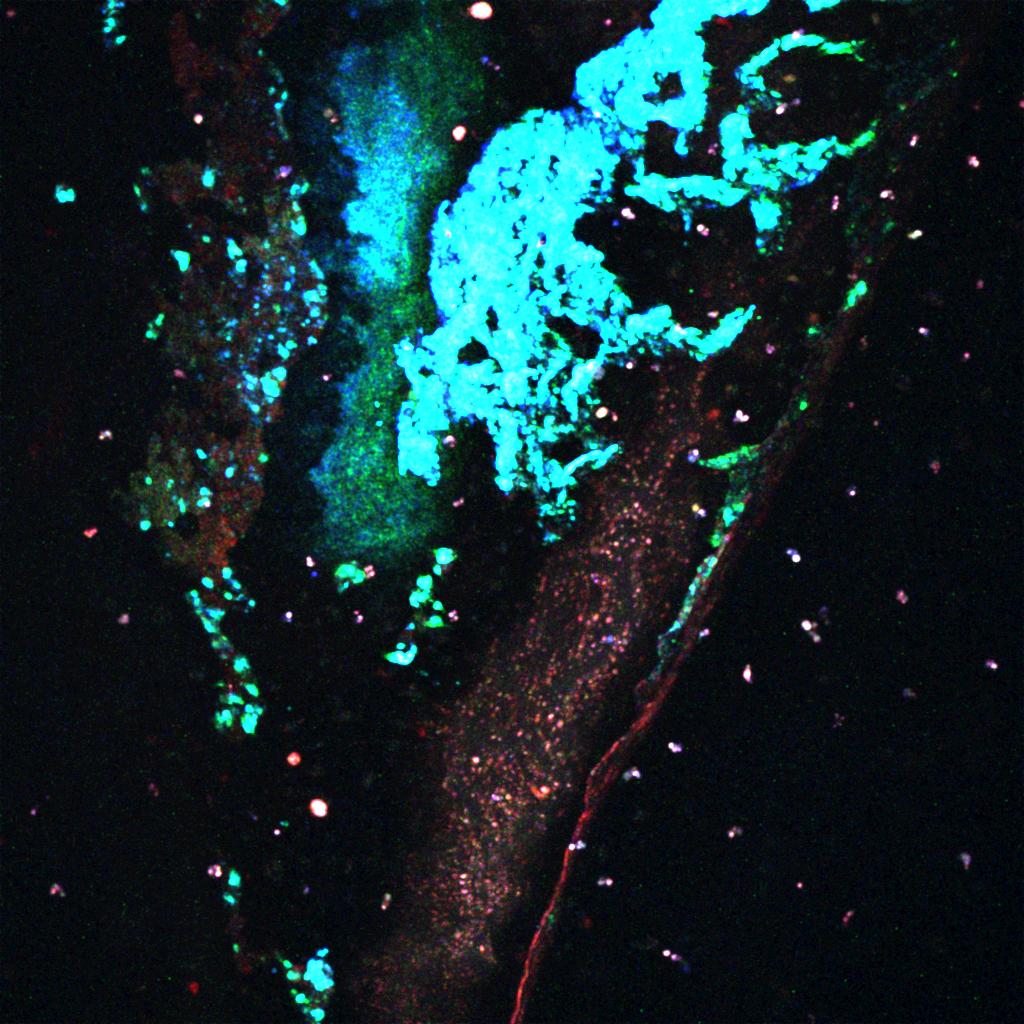

Supplement: Supplementary file 13 — Source data Fig. 4 [file 44318_2024_136_MOESM13_ESM.zip › Figure 4J/palmuscle-Multi-No treatement-14 dpf-19.tif]

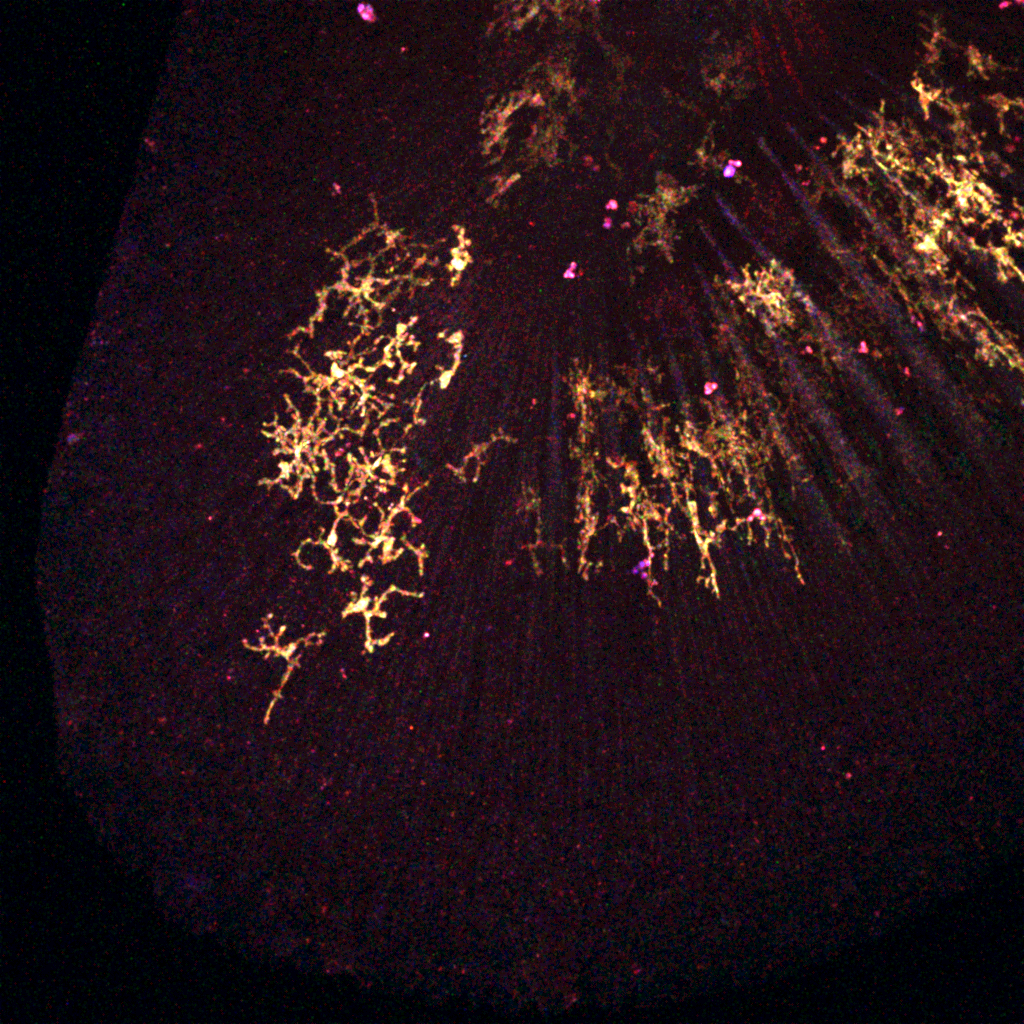

Supplement: Supplementary file 13 — Source data Fig. 4 [file 44318_2024_136_MOESM13_ESM.zip › Figure 4J/palmuscle-Multi-No treatement-14 dpf-2.tif]

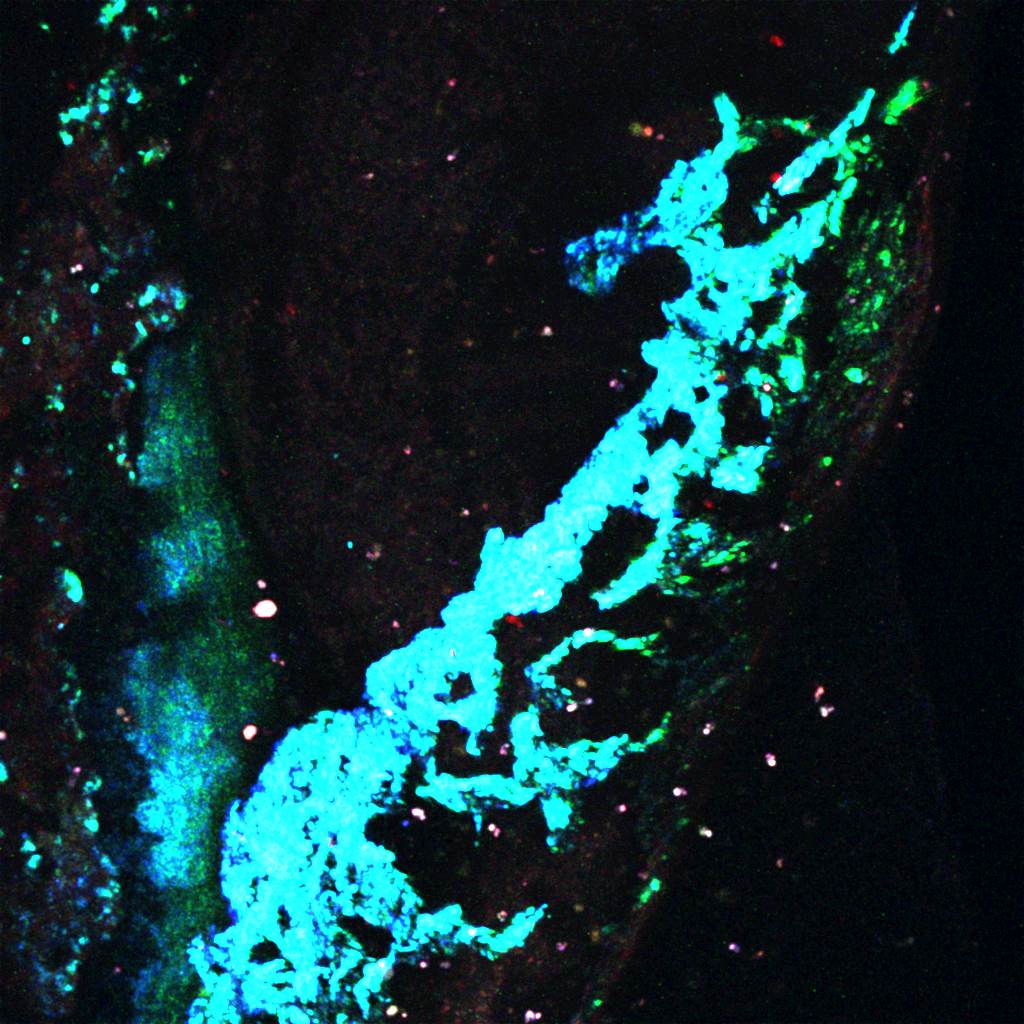

Supplement: Supplementary file 13 — Source data Fig. 4 [file 44318_2024_136_MOESM13_ESM.zip › Figure 4J/palmuscle-Multi-No treatement-14 dpf-20.tif]

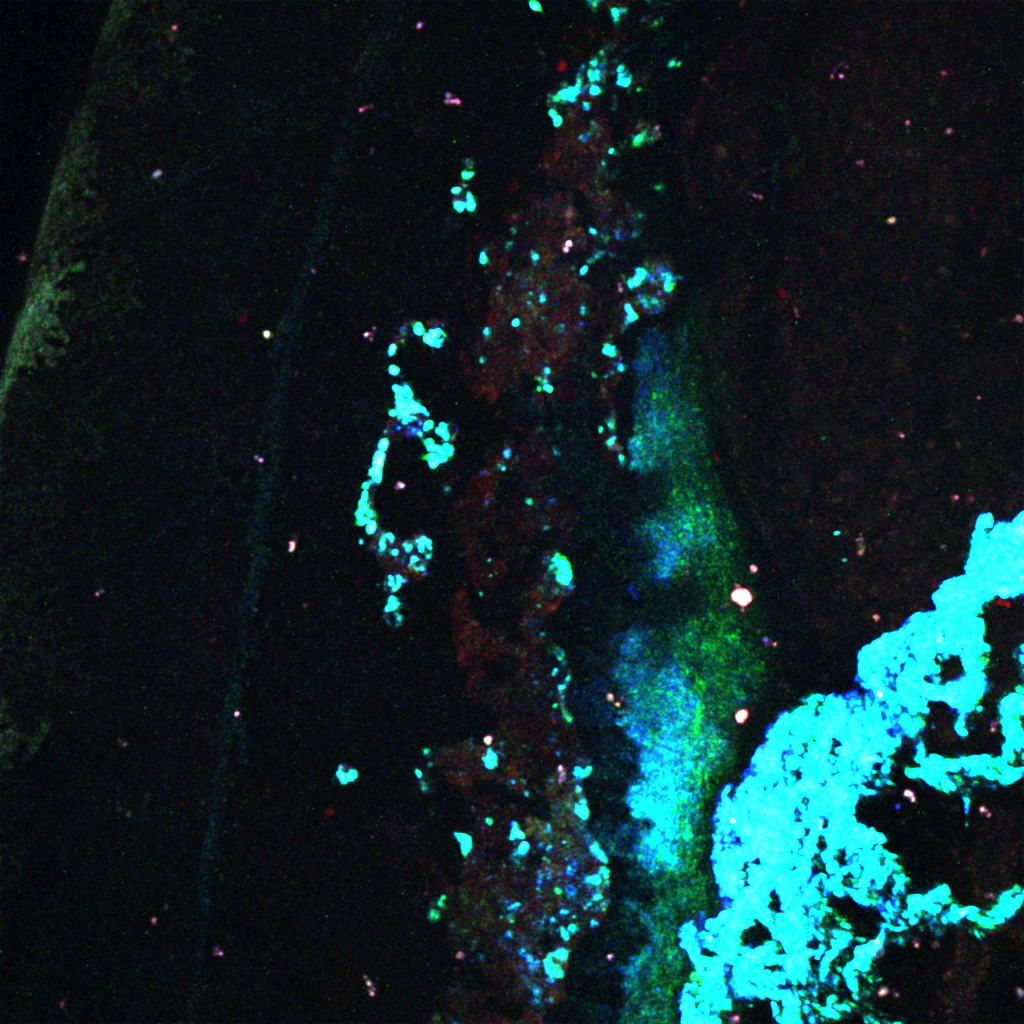

Supplement: Supplementary file 13 — Source data Fig. 4 [file 44318_2024_136_MOESM13_ESM.zip › Figure 4J/palmuscle-Multi-No treatement-14 dpf-21.tif]

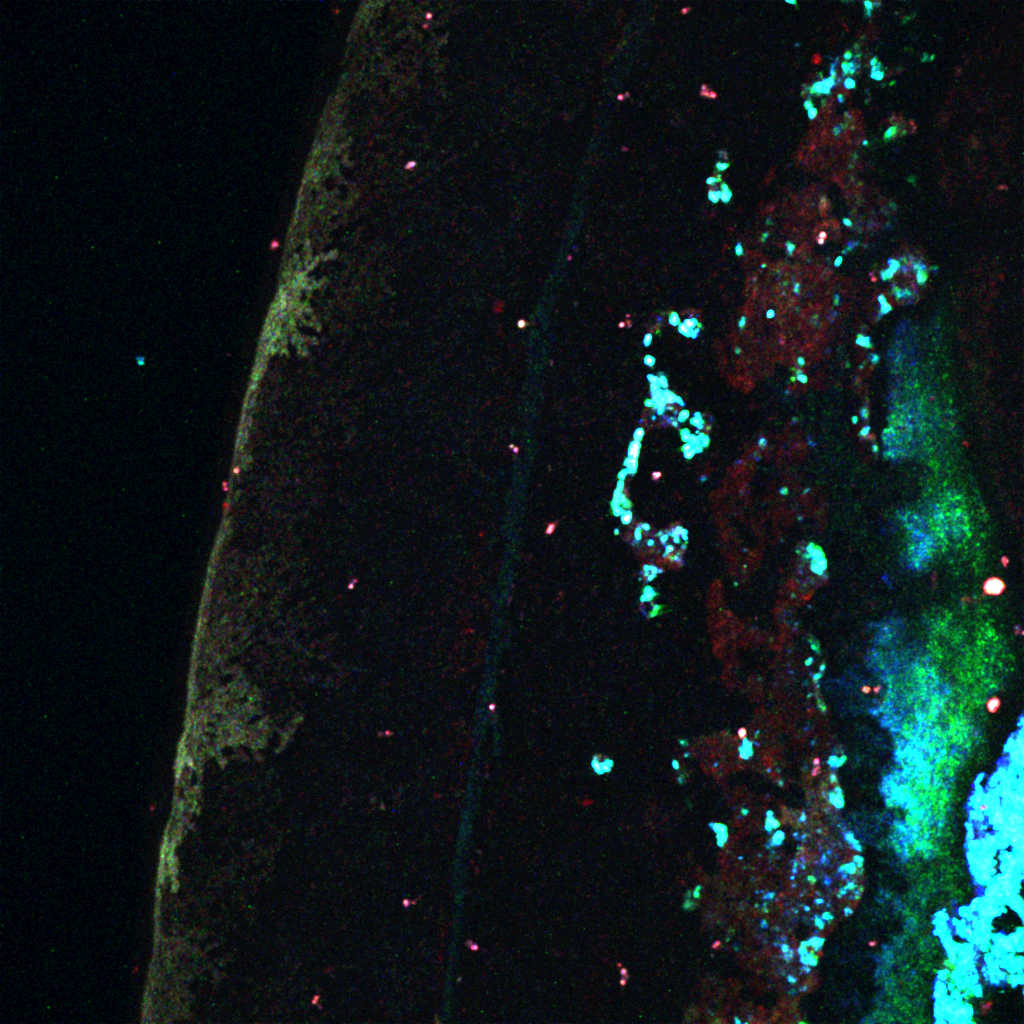

Supplement: Supplementary file 13 — Source data Fig. 4 [file 44318_2024_136_MOESM13_ESM.zip › Figure 4J/palmuscle-Multi-No treatement-14 dpf-22.tif]

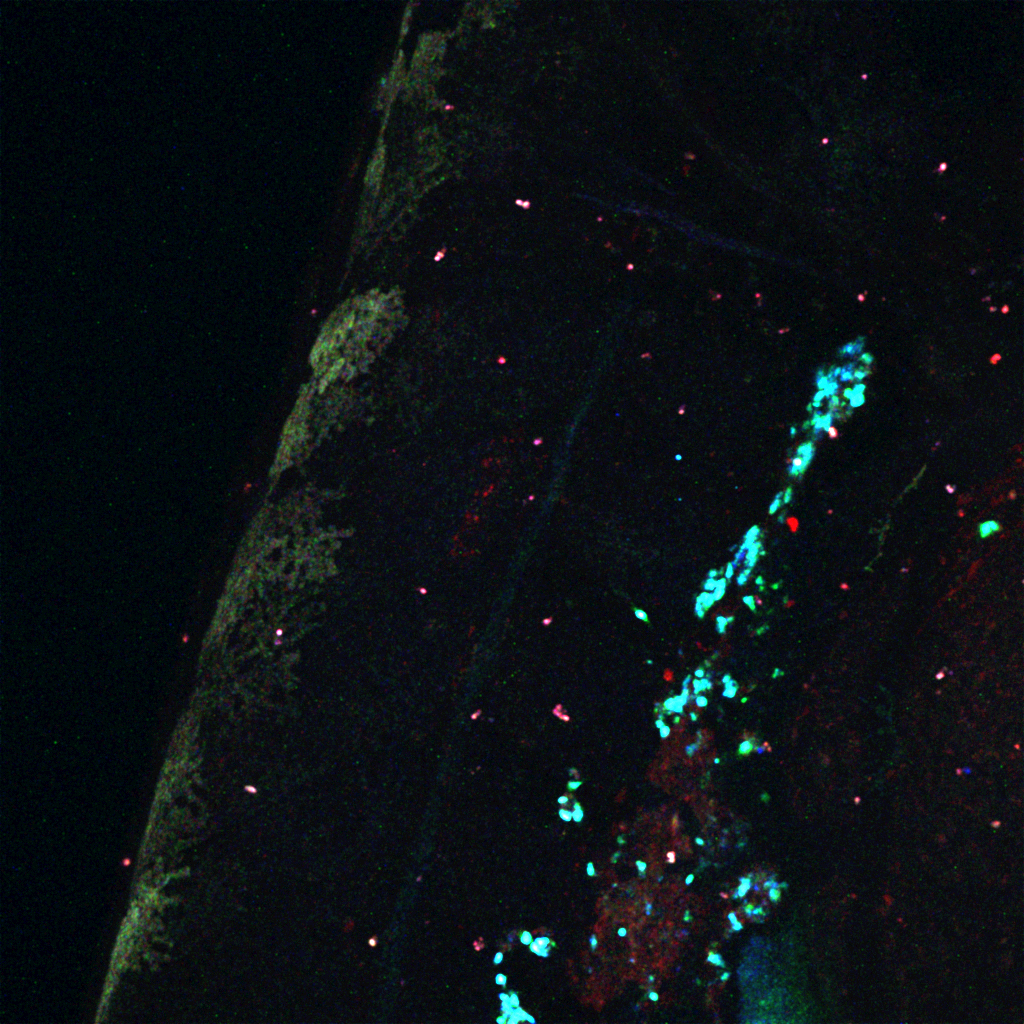

Supplement: Supplementary file 13 — Source data Fig. 4 [file 44318_2024_136_MOESM13_ESM.zip › Figure 4J/palmuscle-Multi-No treatement-14 dpf-23.tif]

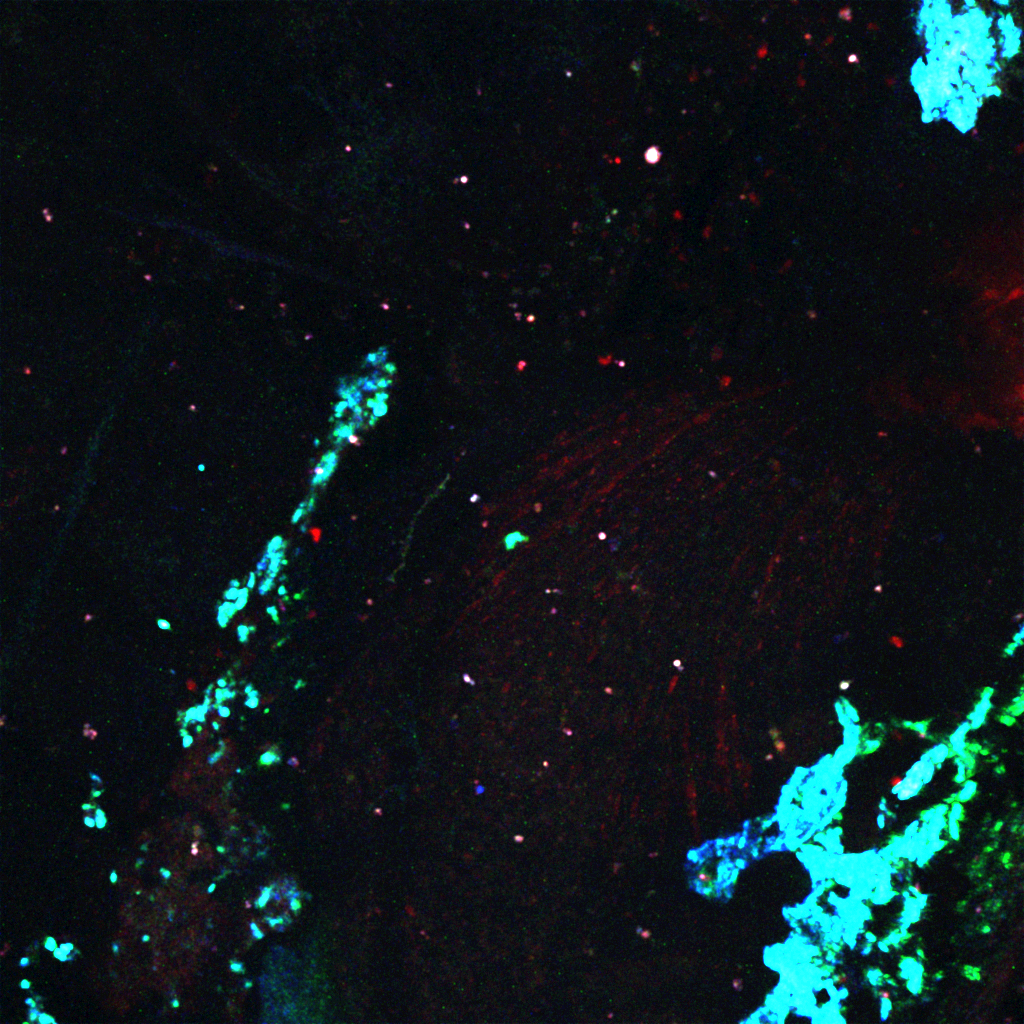

Supplement: Supplementary file 13 — Source data Fig. 4 [file 44318_2024_136_MOESM13_ESM.zip › Figure 4J/palmuscle-Multi-No treatement-14 dpf-24.tif]

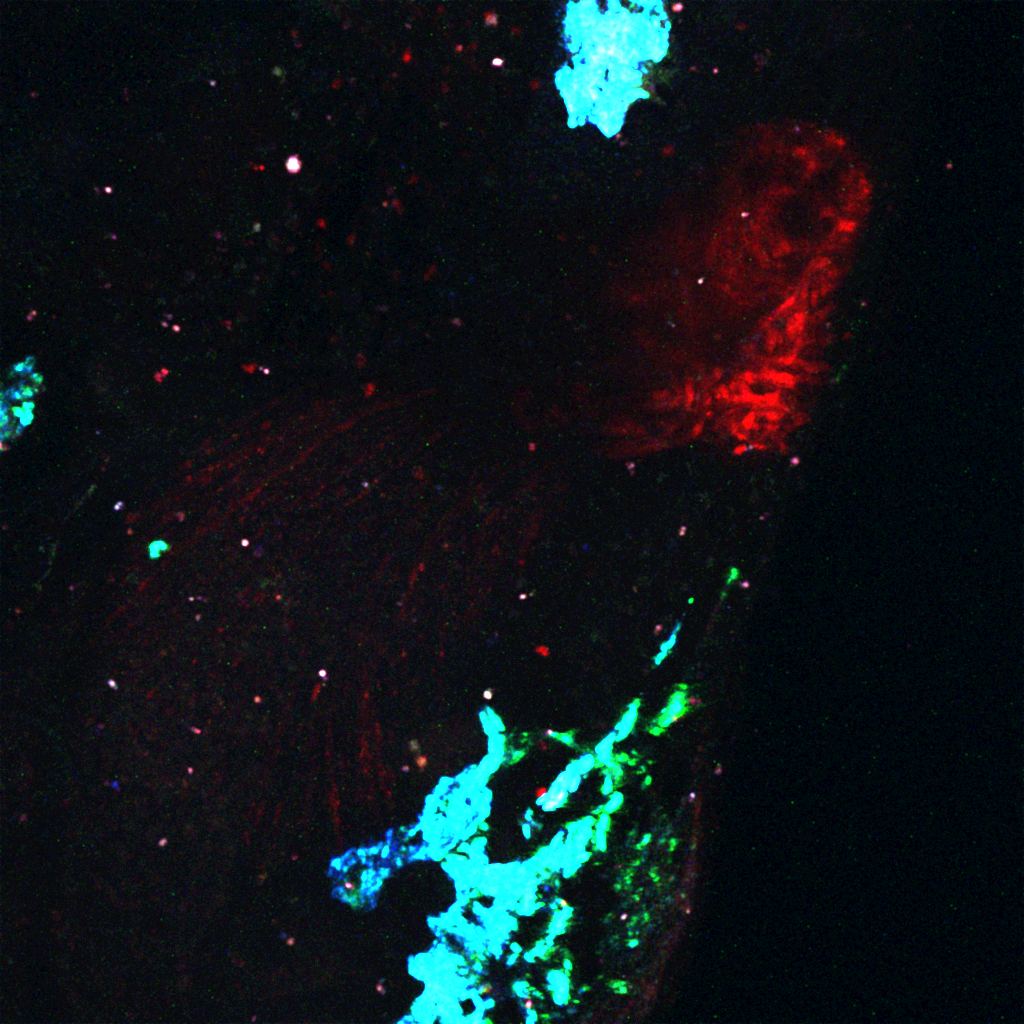

Supplement: Supplementary file 13 — Source data Fig. 4 [file 44318_2024_136_MOESM13_ESM.zip › Figure 4J/palmuscle-Multi-No treatement-14 dpf-25.tif]

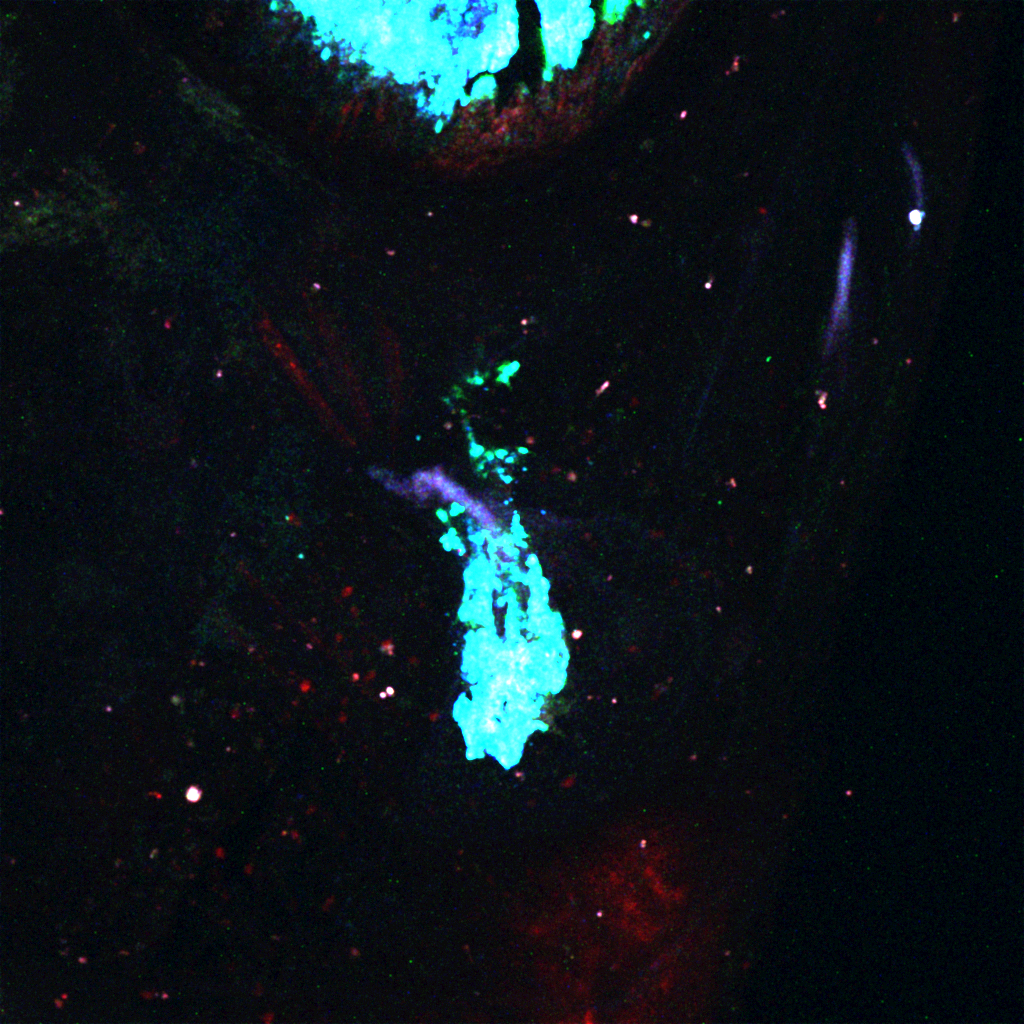

Supplement: Supplementary file 13 — Source data Fig. 4 [file 44318_2024_136_MOESM13_ESM.zip › Figure 4J/palmuscle-Multi-No treatement-14 dpf-26.tif]

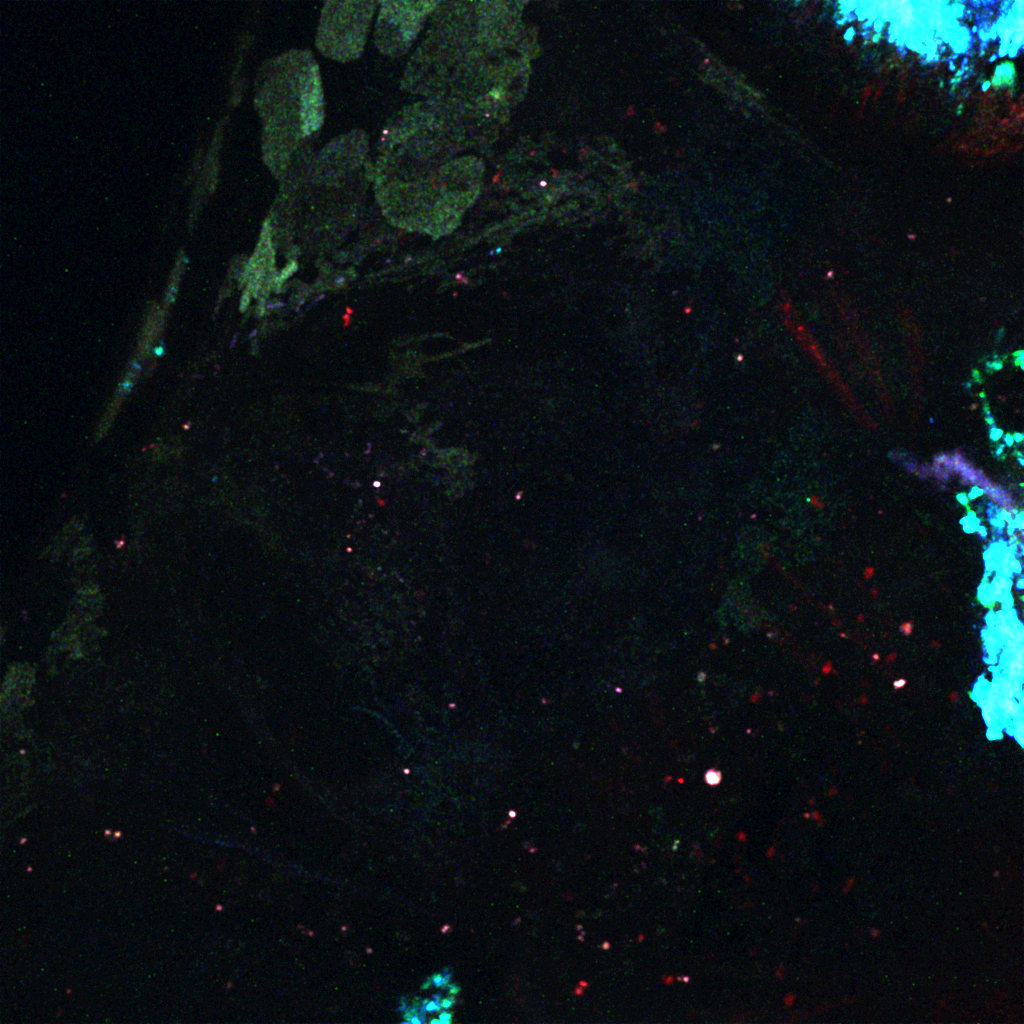

Supplement: Supplementary file 13 — Source data Fig. 4 [file 44318_2024_136_MOESM13_ESM.zip › Figure 4J/palmuscle-Multi-No treatement-14 dpf-27.tif]

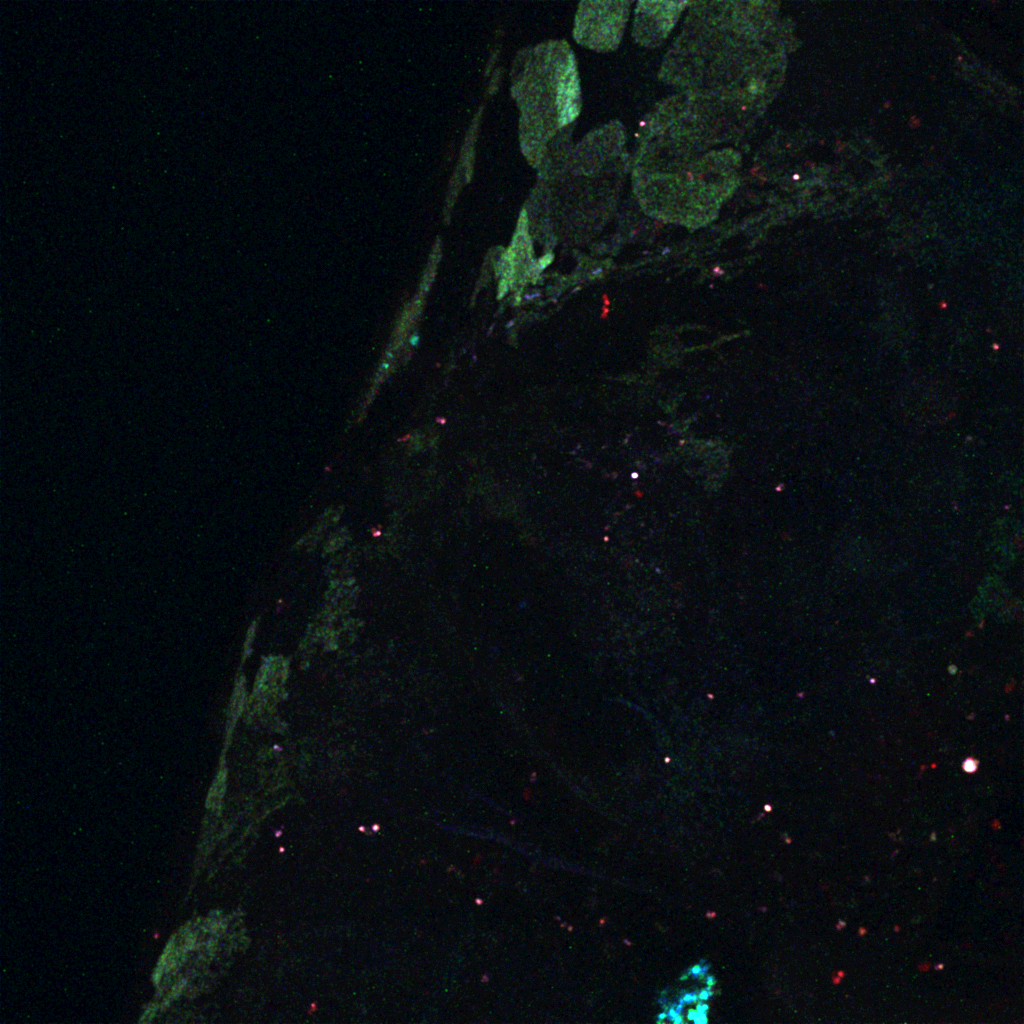

Supplement: Supplementary file 13 — Source data Fig. 4 [file 44318_2024_136_MOESM13_ESM.zip › Figure 4J/palmuscle-Multi-No treatement-14 dpf-28.tif]

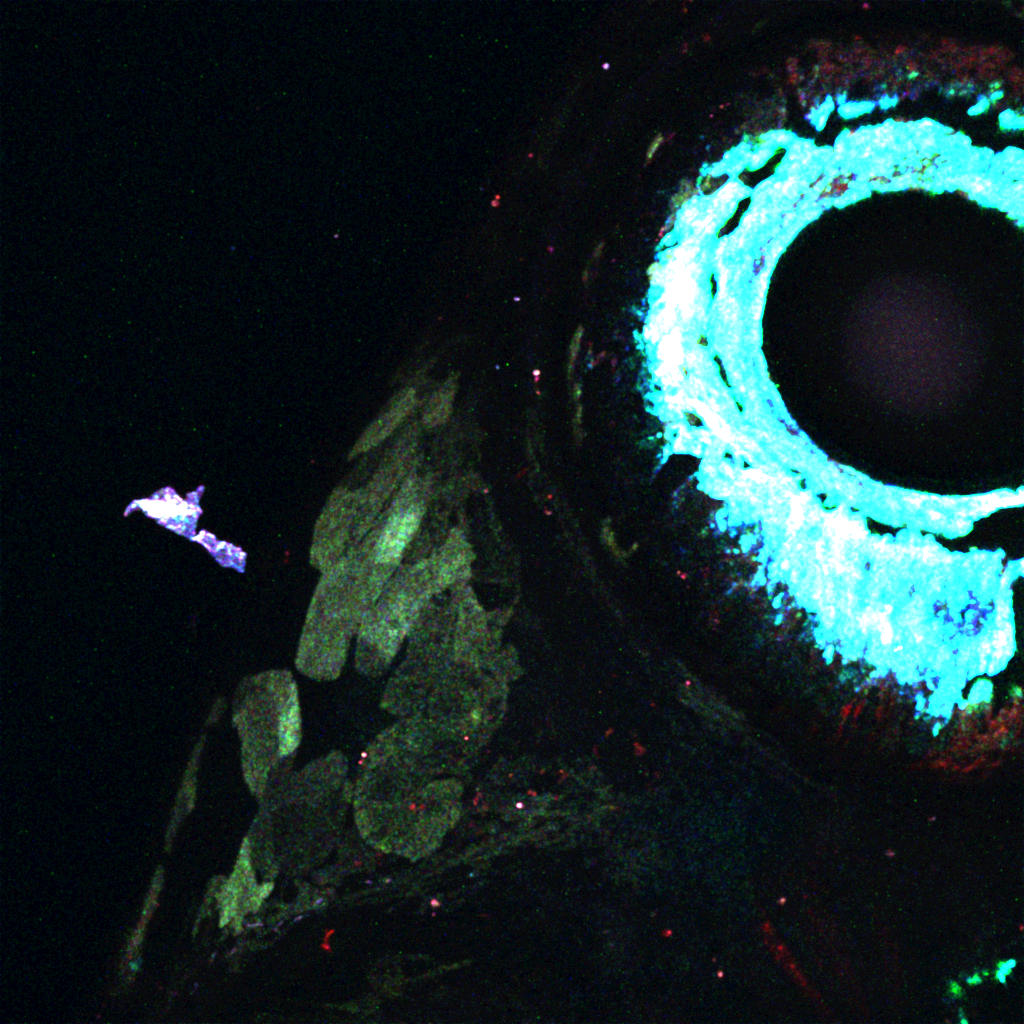

Supplement: Supplementary file 13 — Source data Fig. 4 [file 44318_2024_136_MOESM13_ESM.zip › Figure 4J/palmuscle-Multi-No treatement-14 dpf-29.tif]

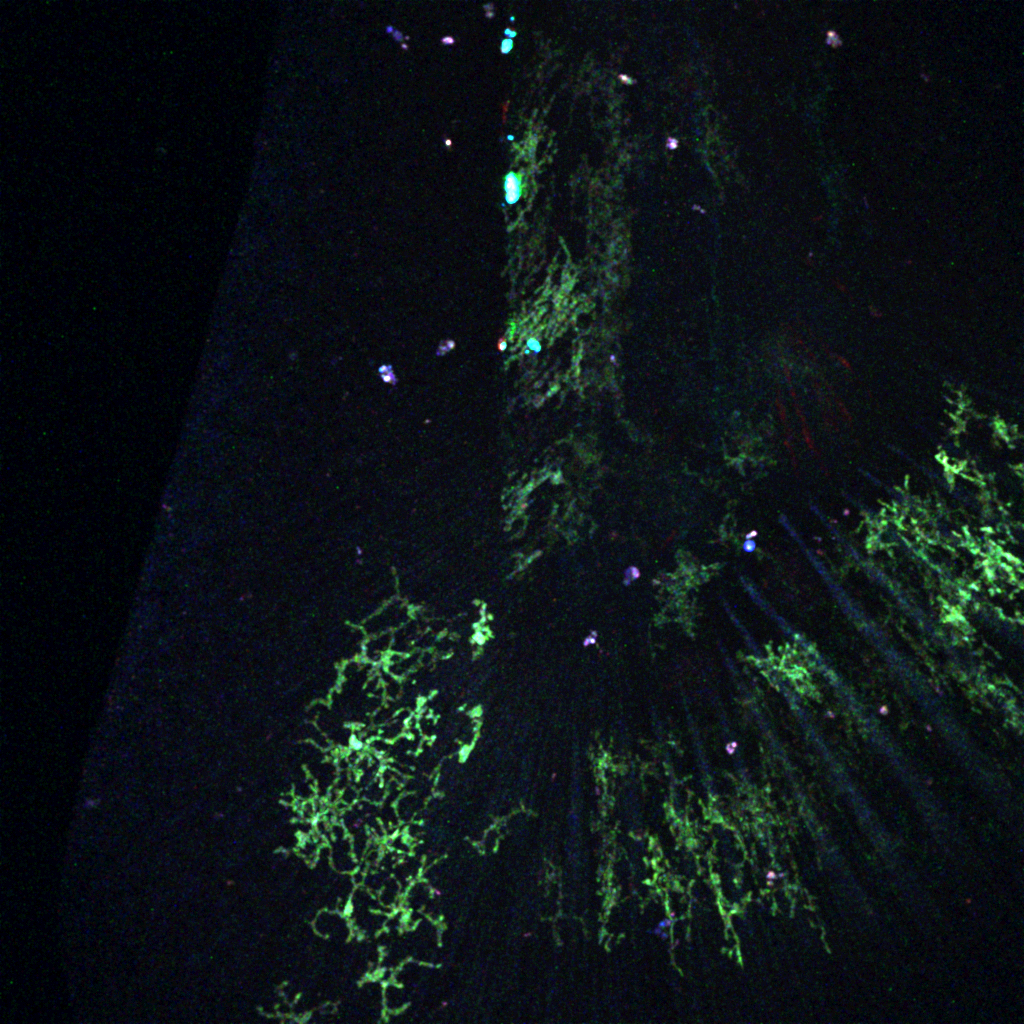

Supplement: Supplementary file 13 — Source data Fig. 4 [file 44318_2024_136_MOESM13_ESM.zip › Figure 4J/palmuscle-Multi-No treatement-14 dpf-3.tif]

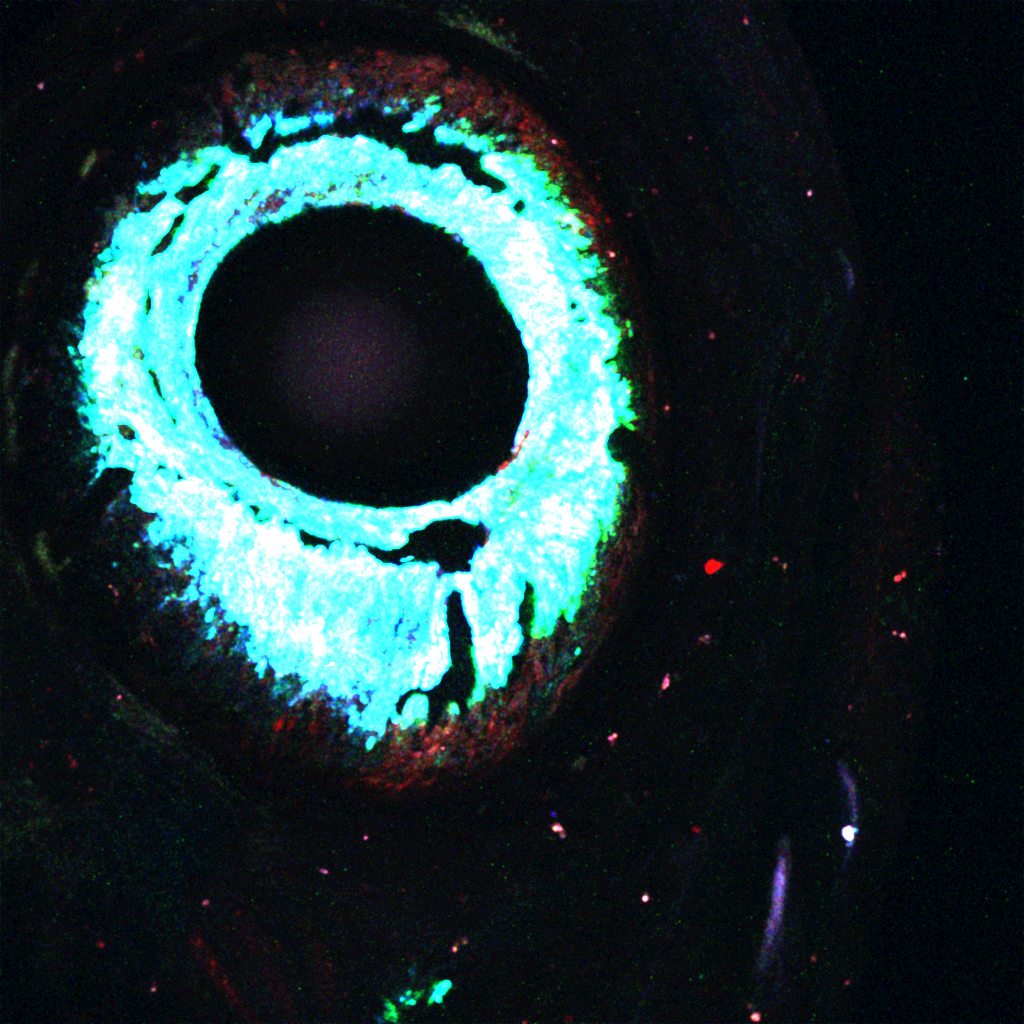

Supplement: Supplementary file 13 — Source data Fig. 4 [file 44318_2024_136_MOESM13_ESM.zip › Figure 4J/palmuscle-Multi-No treatement-14 dpf-30.tif]

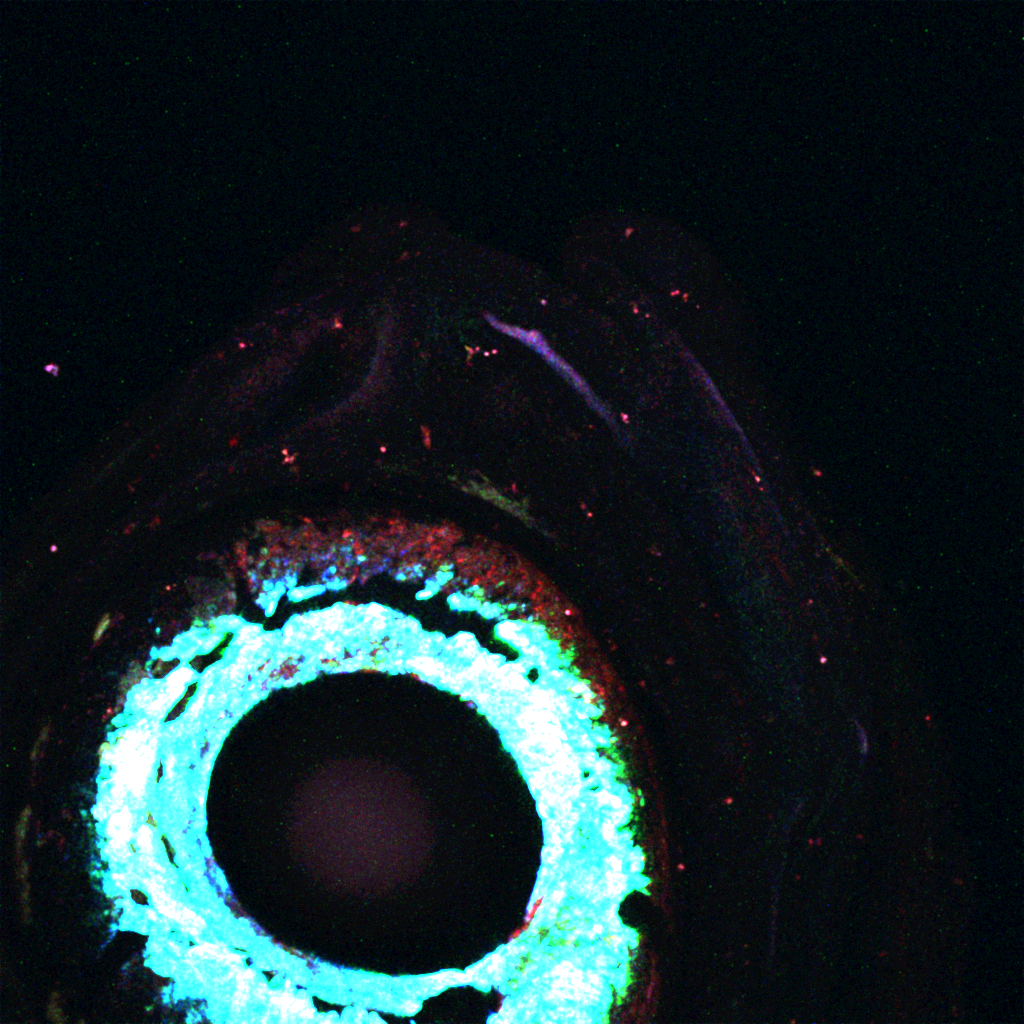

Supplement: Supplementary file 13 — Source data Fig. 4 [file 44318_2024_136_MOESM13_ESM.zip › Figure 4J/palmuscle-Multi-No treatement-14 dpf-31.tif]

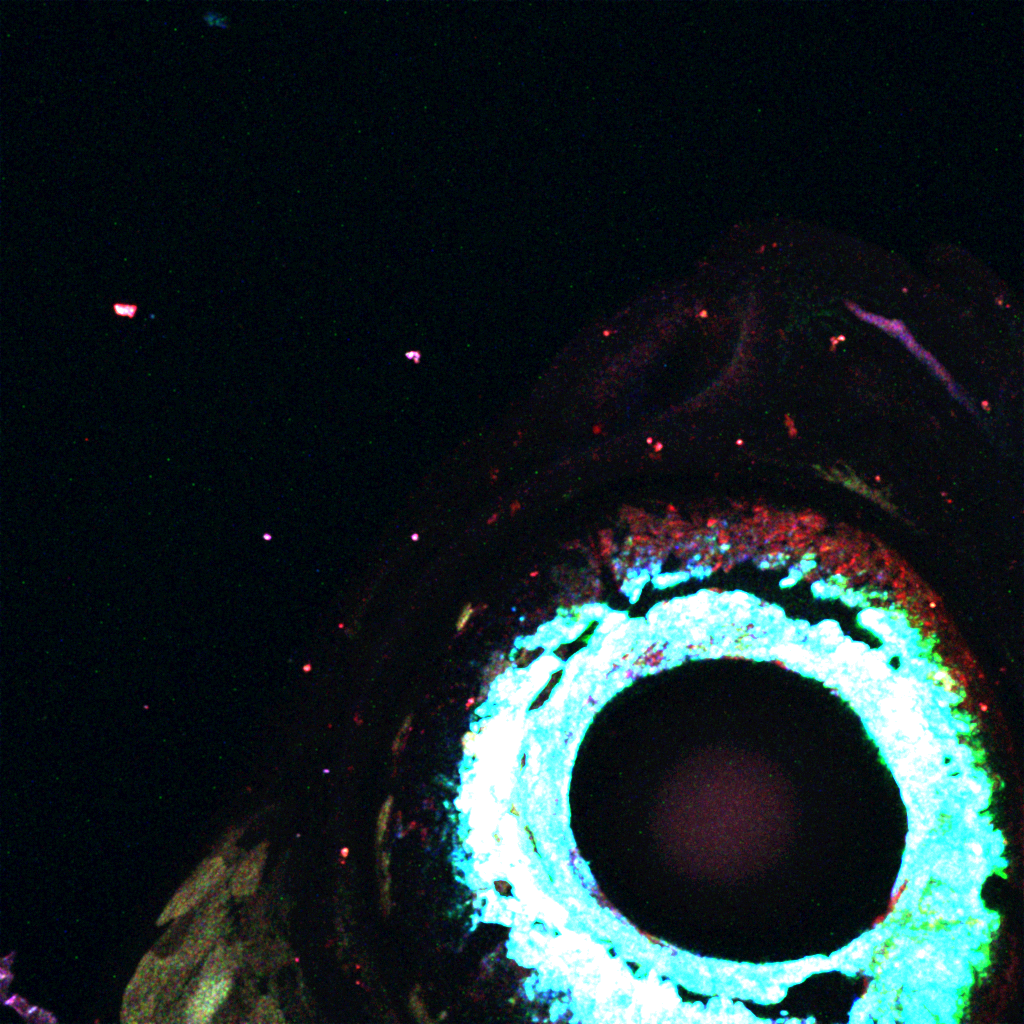

Supplement: Supplementary file 13 — Source data Fig. 4 [file 44318_2024_136_MOESM13_ESM.zip › Figure 4J/palmuscle-Multi-No treatement-14 dpf-32.tif]

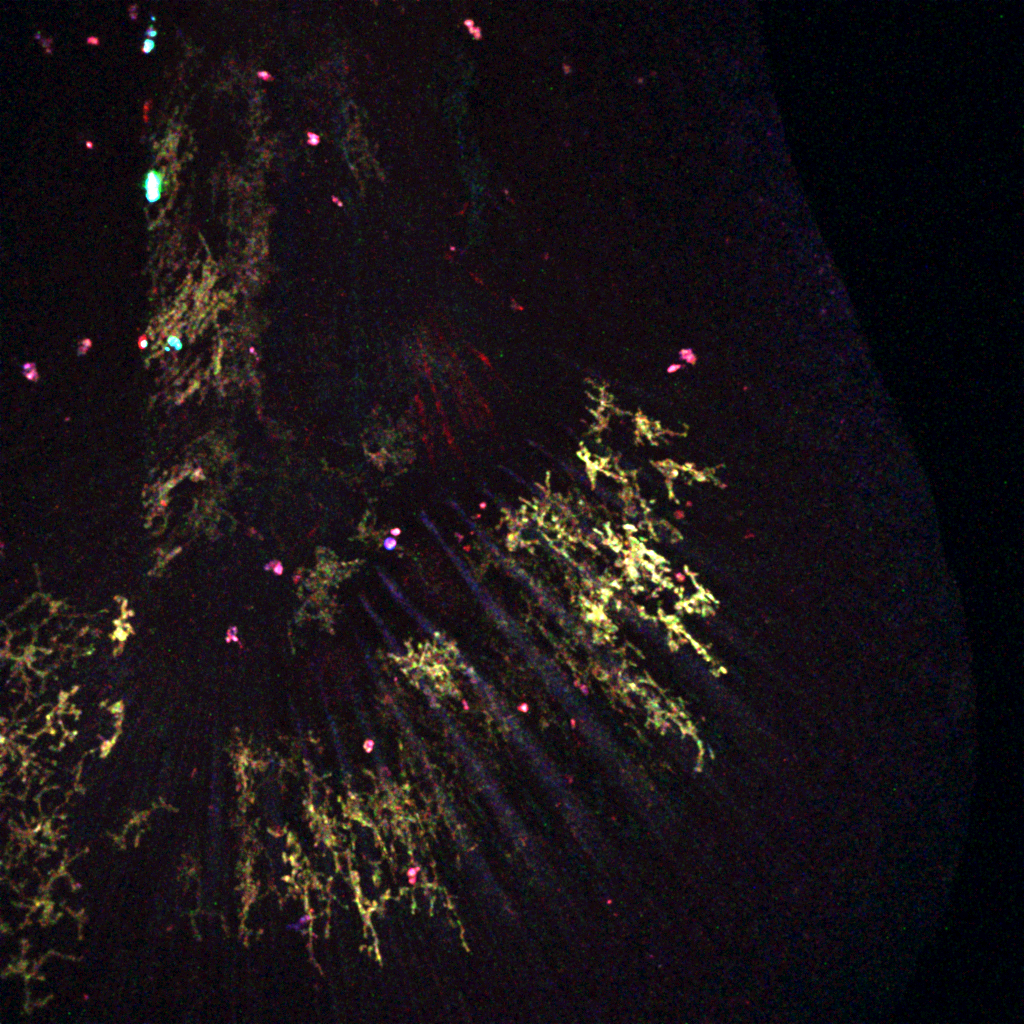

Supplement: Supplementary file 13 — Source data Fig. 4 [file 44318_2024_136_MOESM13_ESM.zip › Figure 4J/palmuscle-Multi-No treatement-14 dpf-4.tif]

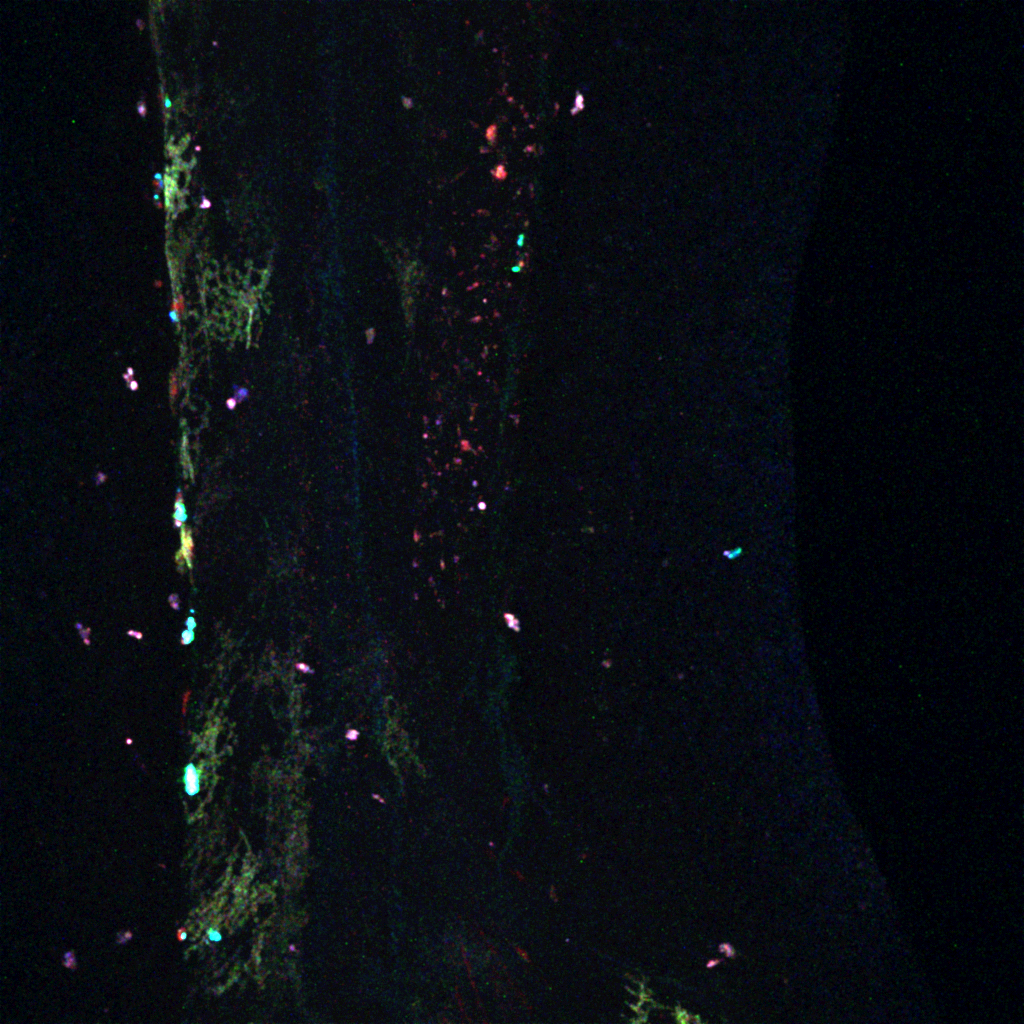

Supplement: Supplementary file 13 — Source data Fig. 4 [file 44318_2024_136_MOESM13_ESM.zip › Figure 4J/palmuscle-Multi-No treatement-14 dpf-5.tif]

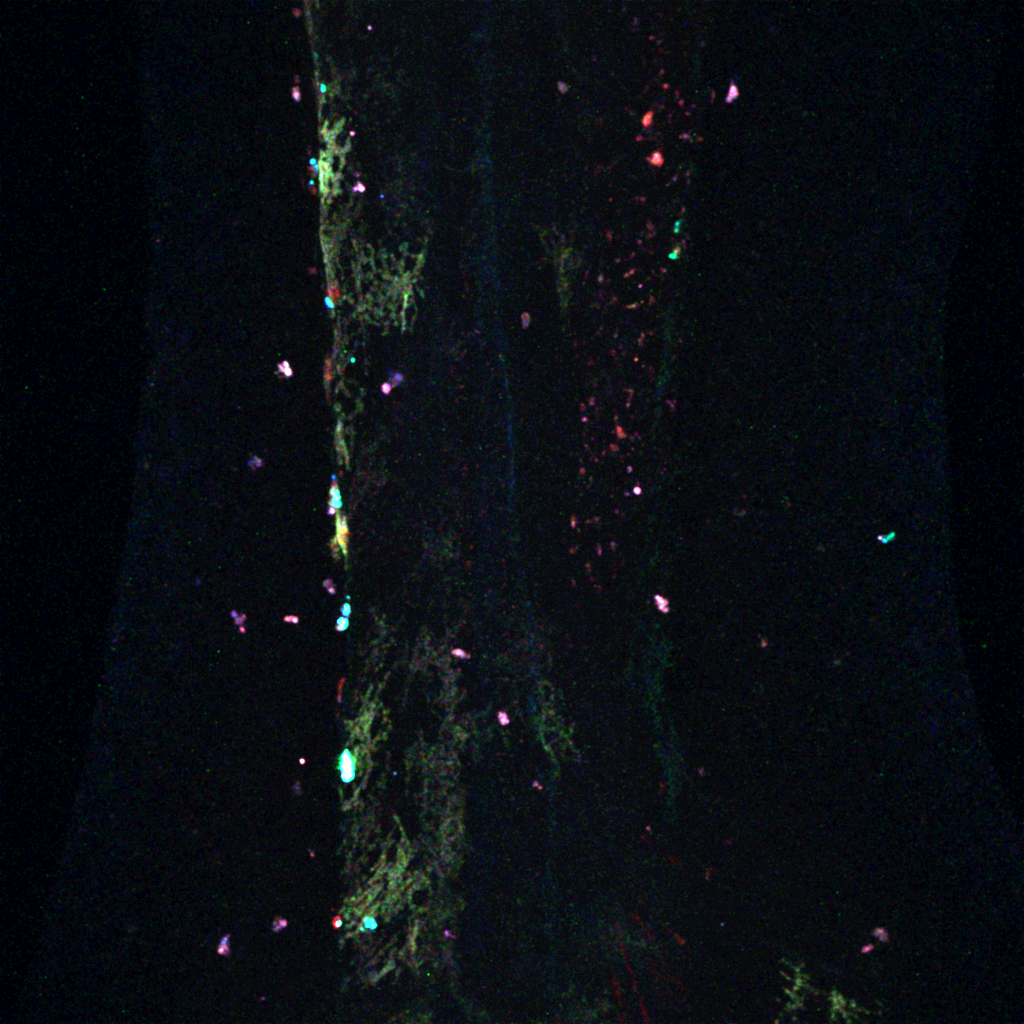

Supplement: Supplementary file 13 — Source data Fig. 4 [file 44318_2024_136_MOESM13_ESM.zip › Figure 4J/palmuscle-Multi-No treatement-14 dpf-6.tif]

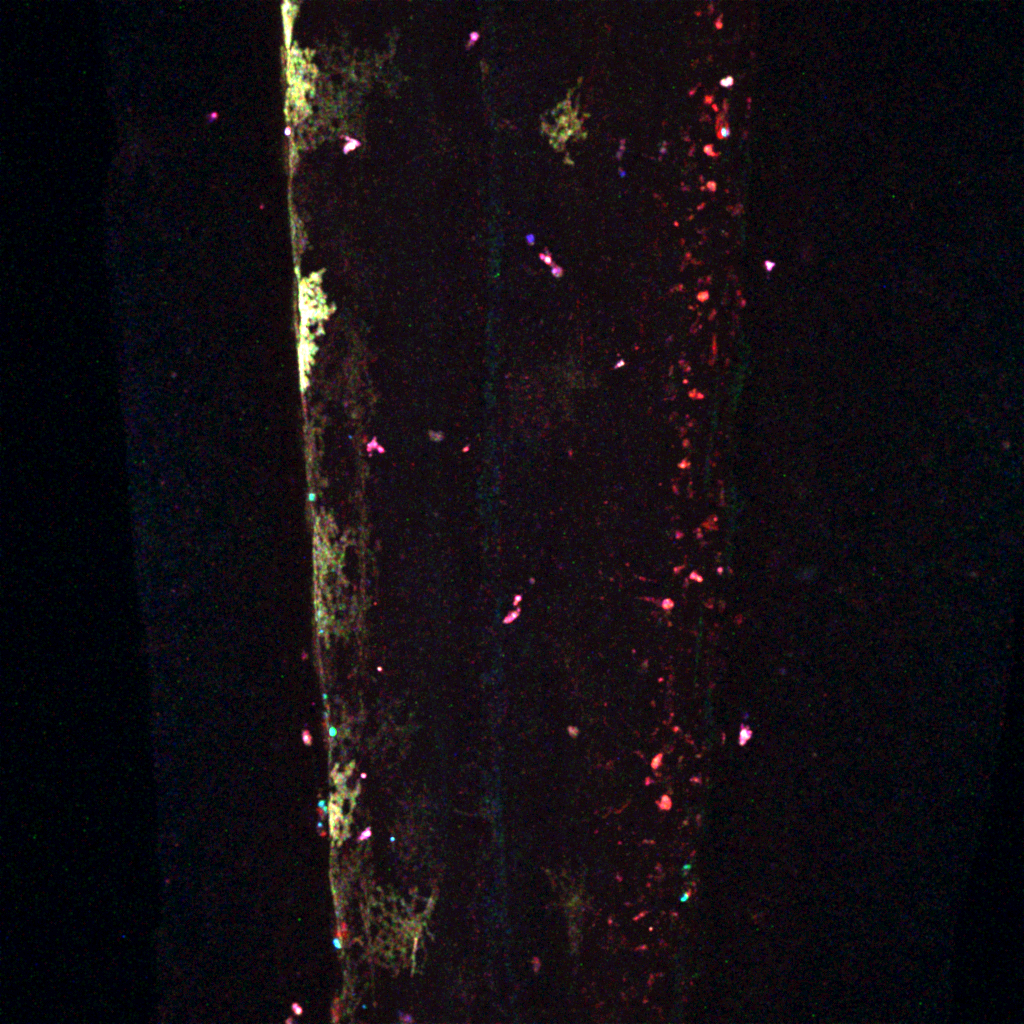

Supplement: Supplementary file 13 — Source data Fig. 4 [file 44318_2024_136_MOESM13_ESM.zip › Figure 4J/palmuscle-Multi-No treatement-14 dpf-7.tif]

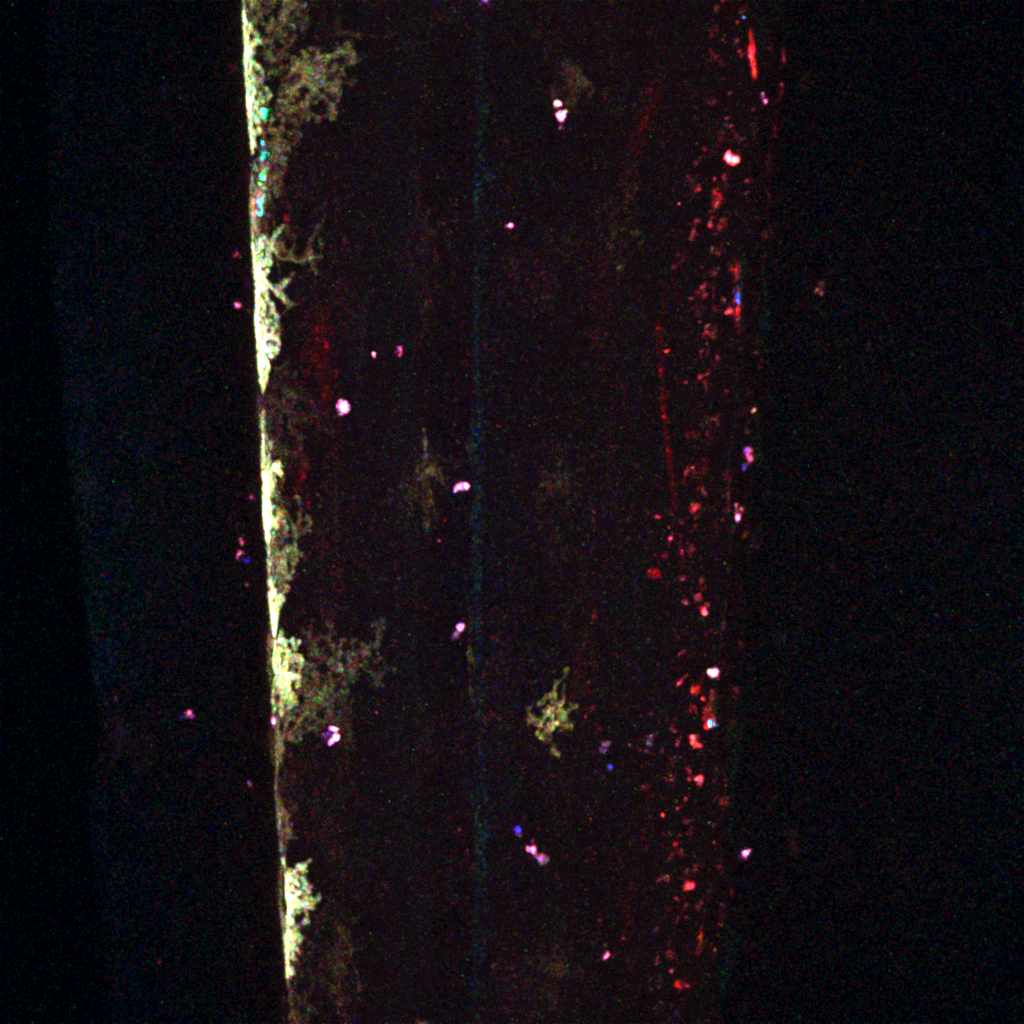

Supplement: Supplementary file 13 — Source data Fig. 4 [file 44318_2024_136_MOESM13_ESM.zip › Figure 4J/palmuscle-Multi-No treatement-14 dpf-8.tif]

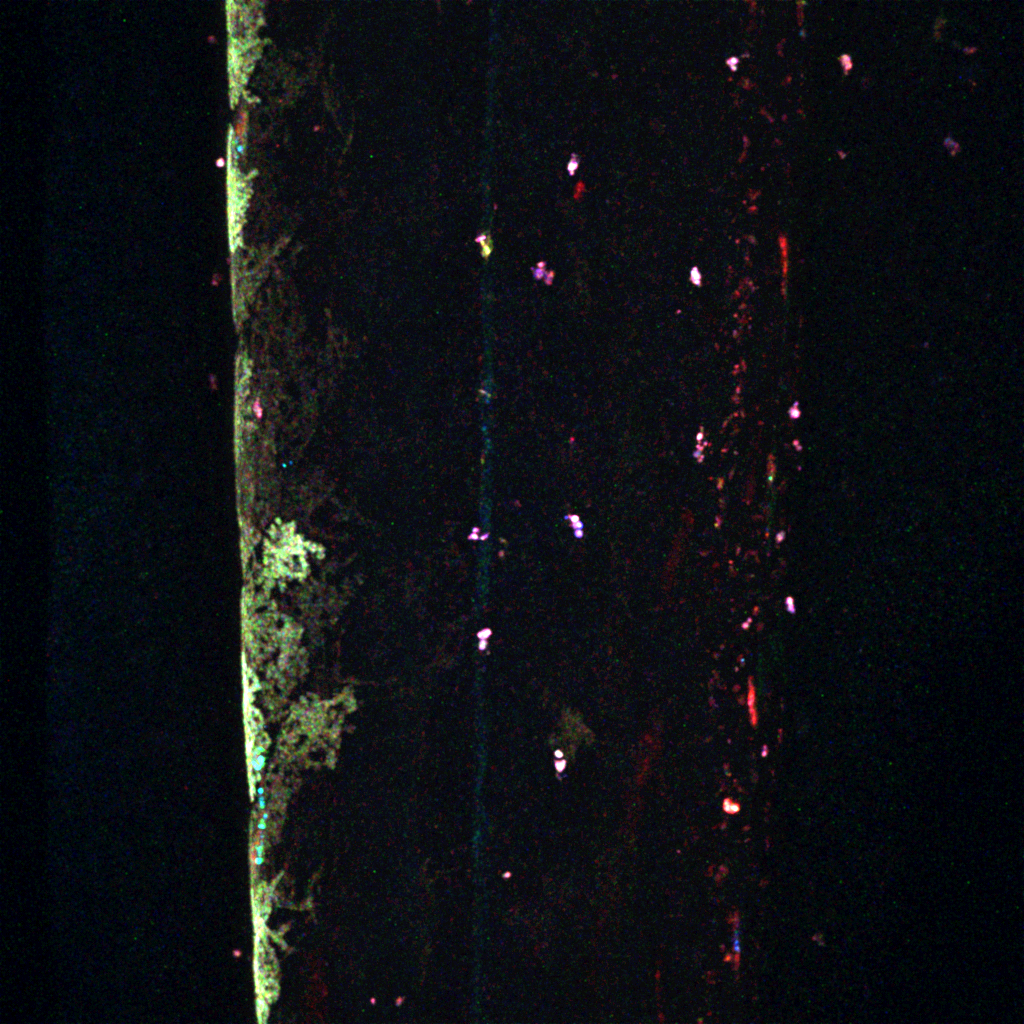

Supplement: Supplementary file 13 — Source data Fig. 4 [file 44318_2024_136_MOESM13_ESM.zip › Figure 4J/palmuscle-Multi-No treatement-14 dpf-9.tif]

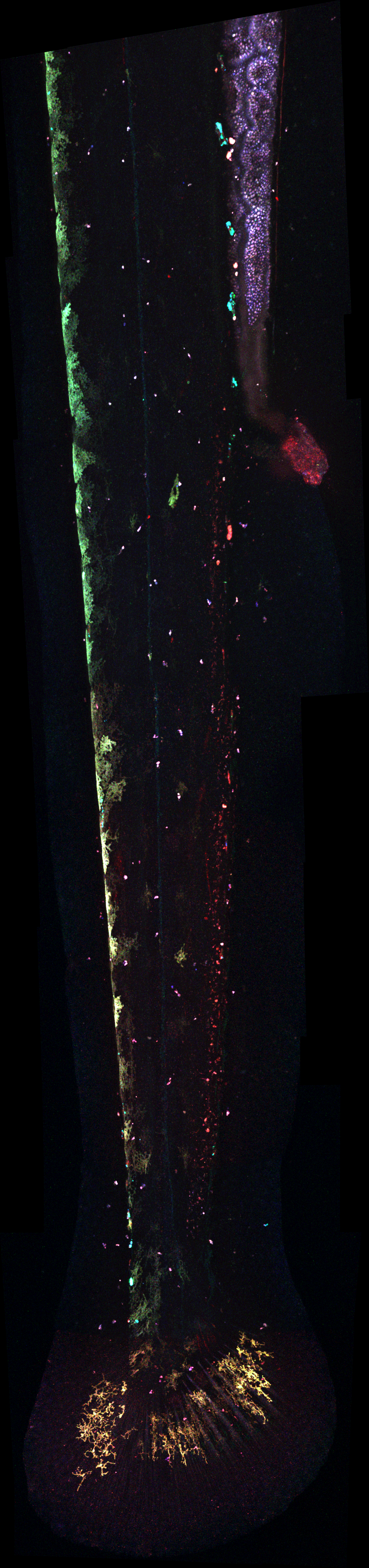

Supplement: Supplementary file 13 — Source data Fig. 4 [file 44318_2024_136_MOESM13_ESM.zip › Figure 4J/palmuscle-Multi-No treatement-14 dpf-stitched 1.tiff]

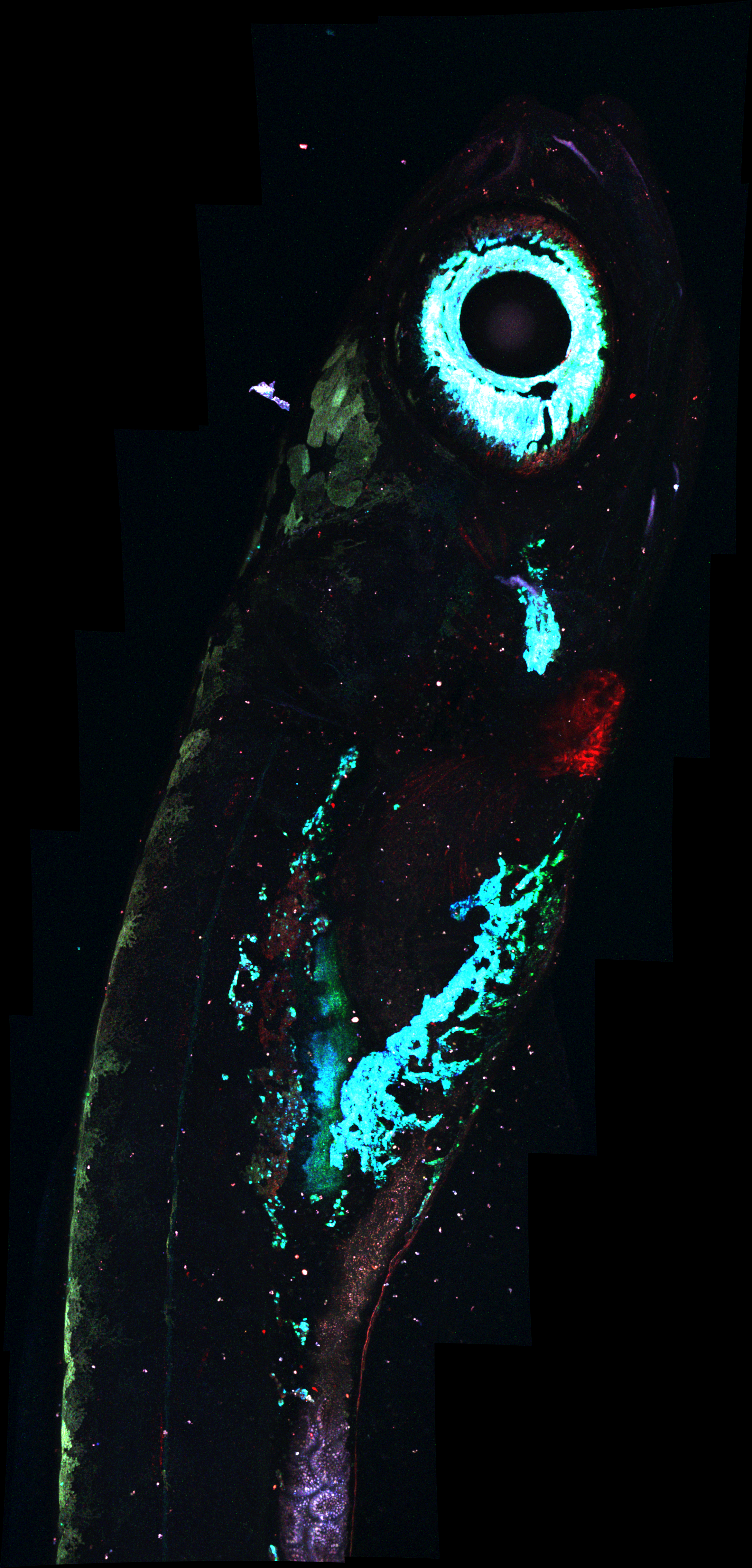

Supplement: Supplementary file 13 — Source data Fig. 4 [file 44318_2024_136_MOESM13_ESM.zip › Figure 4J/palmuscle-Multi-No treatement-14 dpf-stitched 2.tiff]

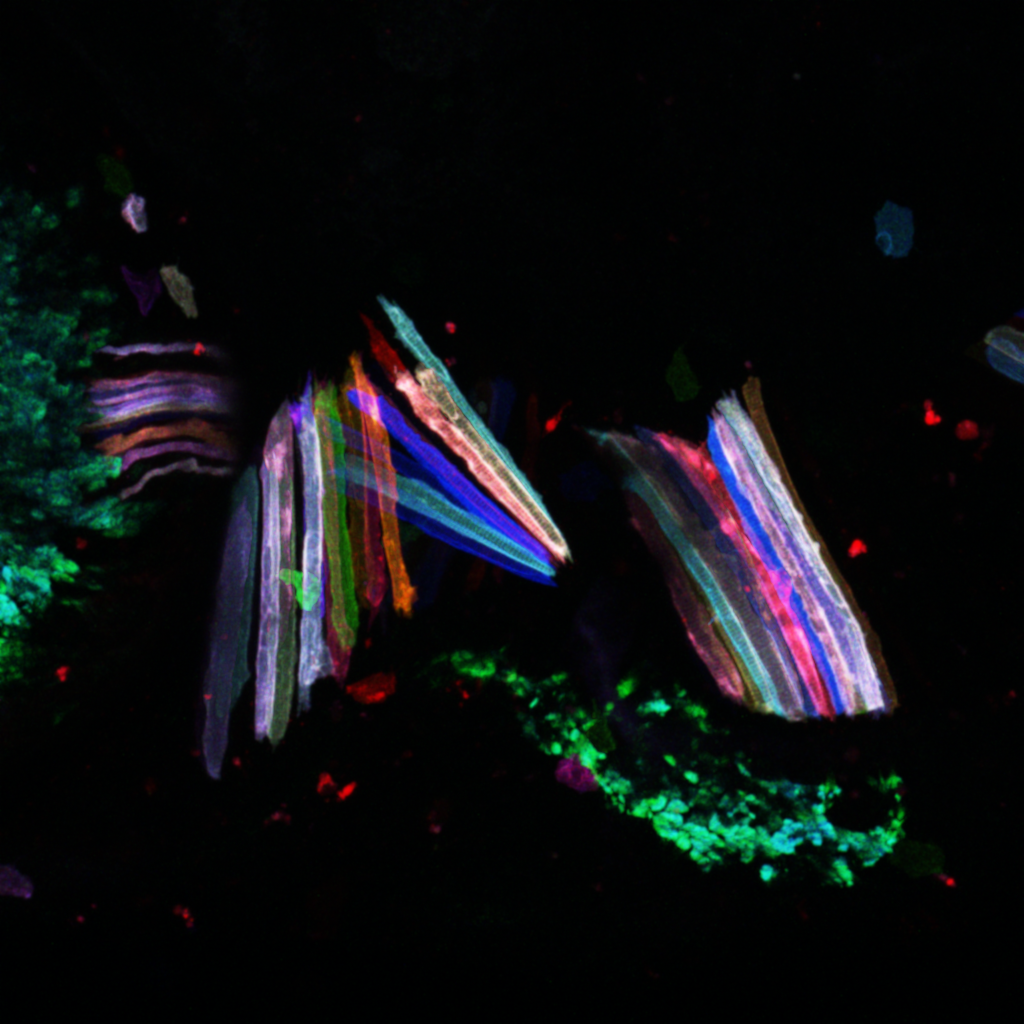

Supplement: Supplementary file 13 — Source data Fig. 4 [file 44318_2024_136_MOESM13_ESM.zip › Figure 4K/palmulscle-Multi-craniofacial.tif]

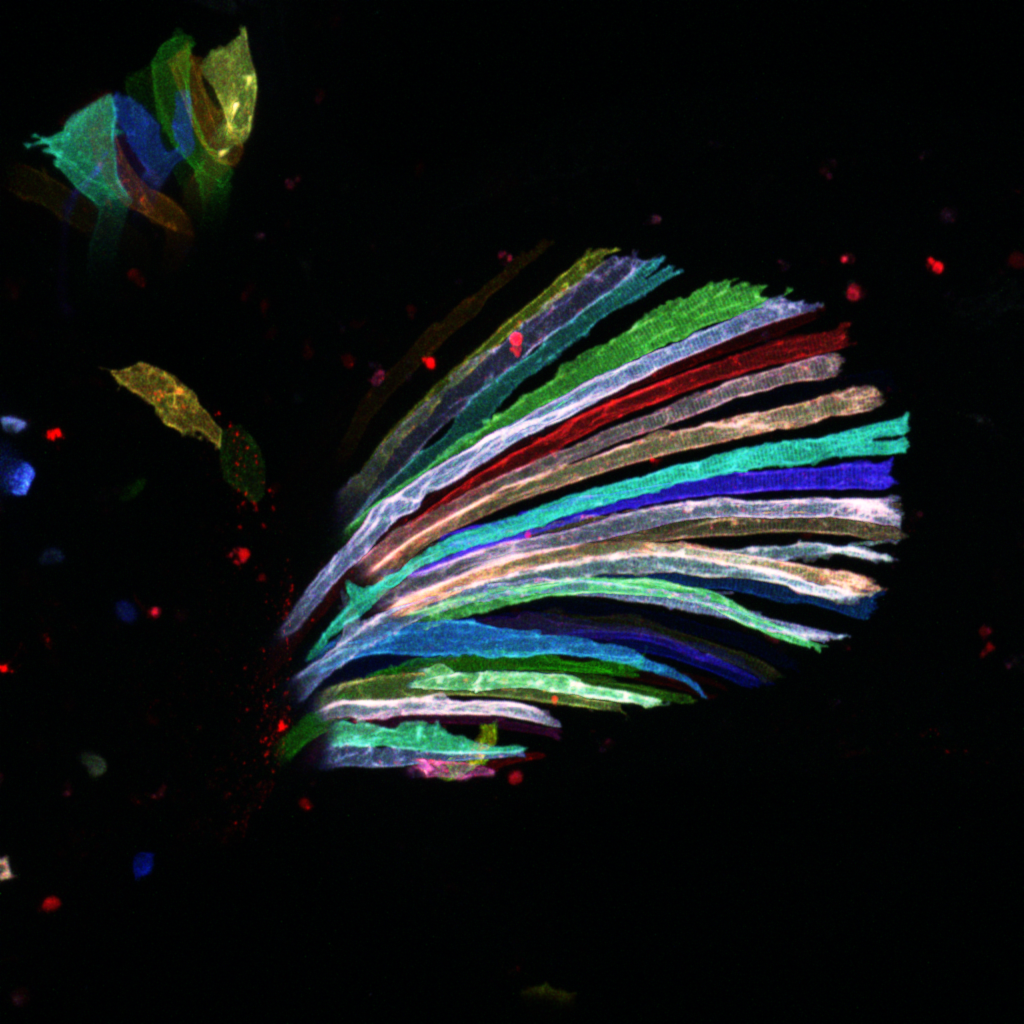

Supplement: Supplementary file 13 — Source data Fig. 4 [file 44318_2024_136_MOESM13_ESM.zip › Figure 4K/palmulscle-Multi-pectoral fin.tif]

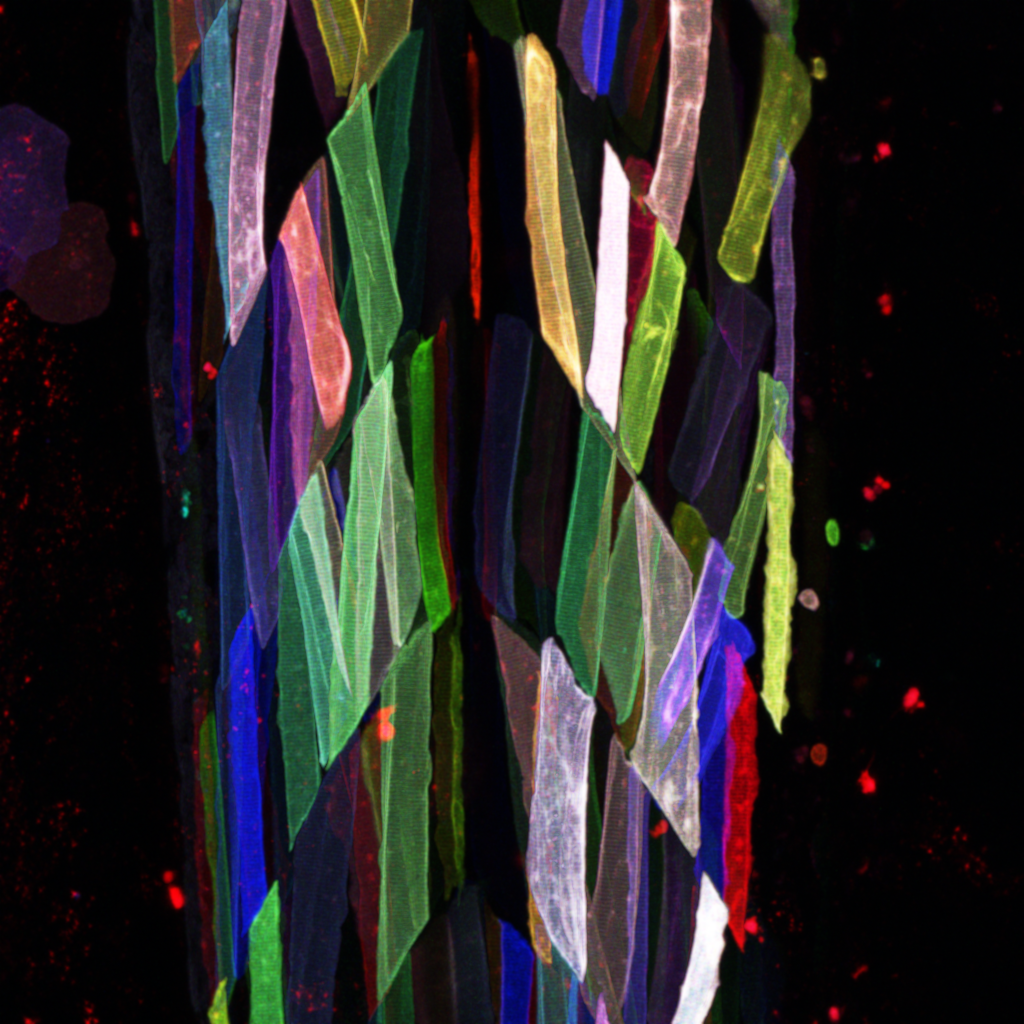

Supplement: Supplementary file 13 — Source data Fig. 4 [file 44318_2024_136_MOESM13_ESM.zip › Figure 4K/palmulscle-Multi-trunk.tif]

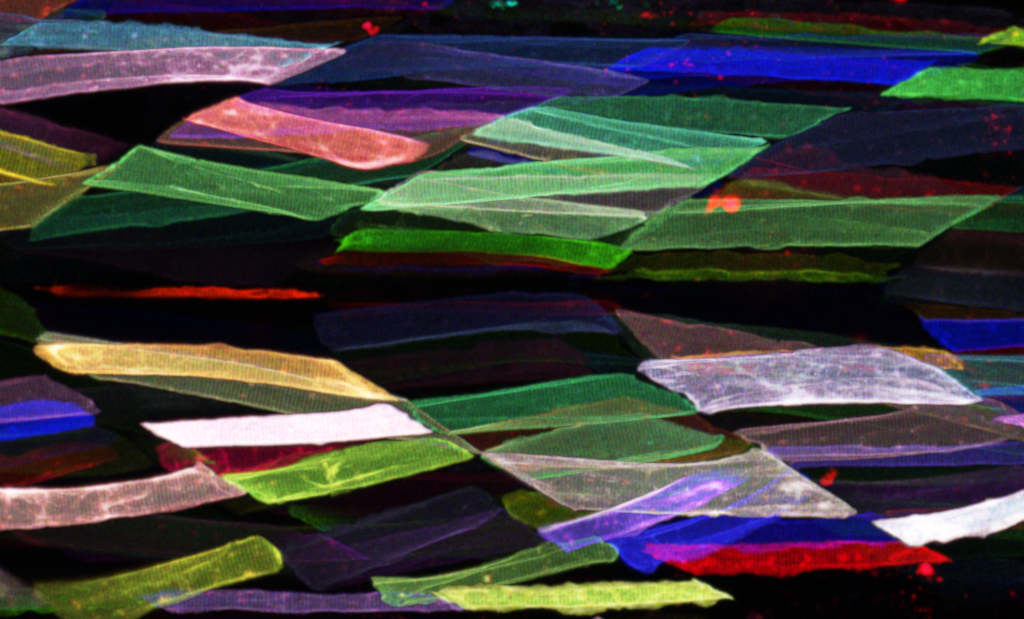

Supplement: Supplementary file 13 — Source data Fig. 4 [file 44318_2024_136_MOESM13_ESM.zip › Figure 4K/palmulscle-Multi-trunk-crop.tif]

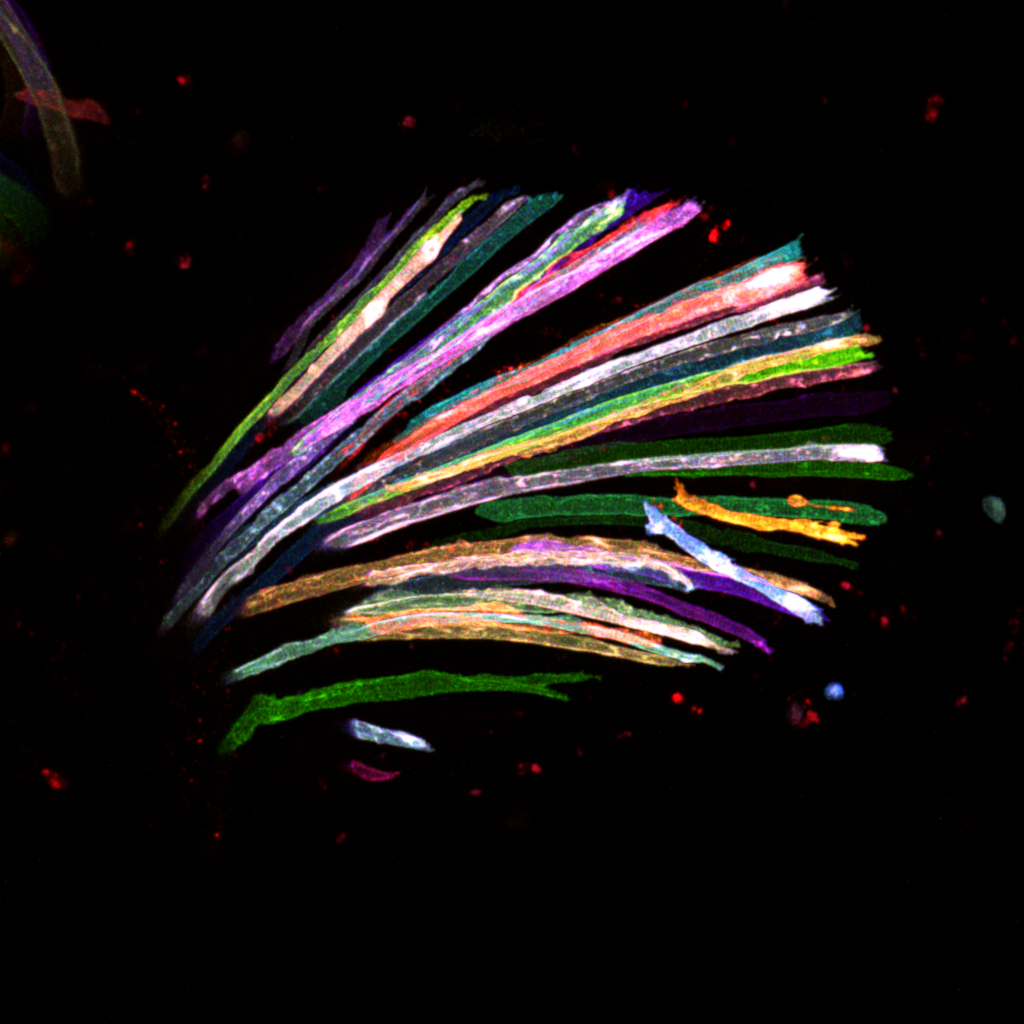

Supplement: Supplementary file 14 — Source data Fig. 5 [file 44318_2024_136_MOESM14_ESM.zip › Figure 5B/Pectoral fin-14 dpf.tif]

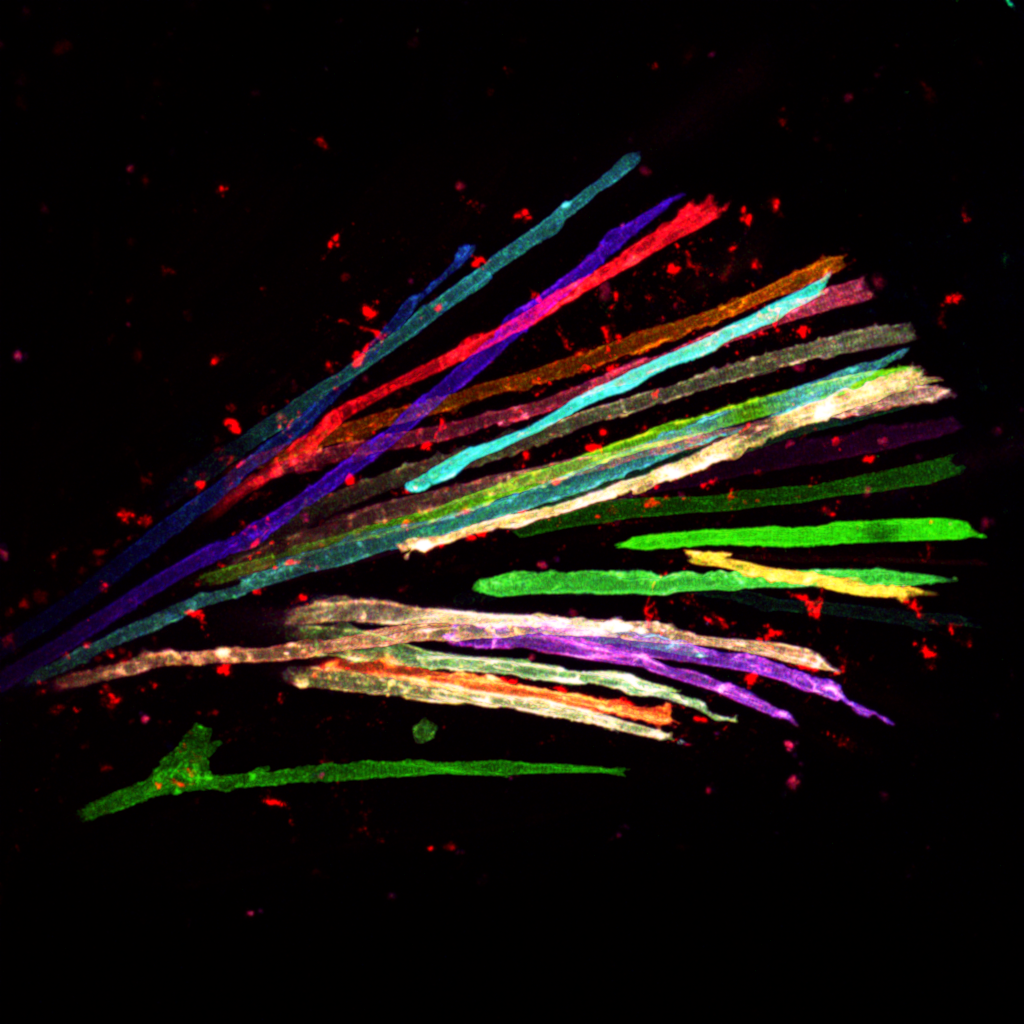

Supplement: Supplementary file 14 — Source data Fig. 5 [file 44318_2024_136_MOESM14_ESM.zip › Figure 5B/Pectoral fin-21 dpf.tif]

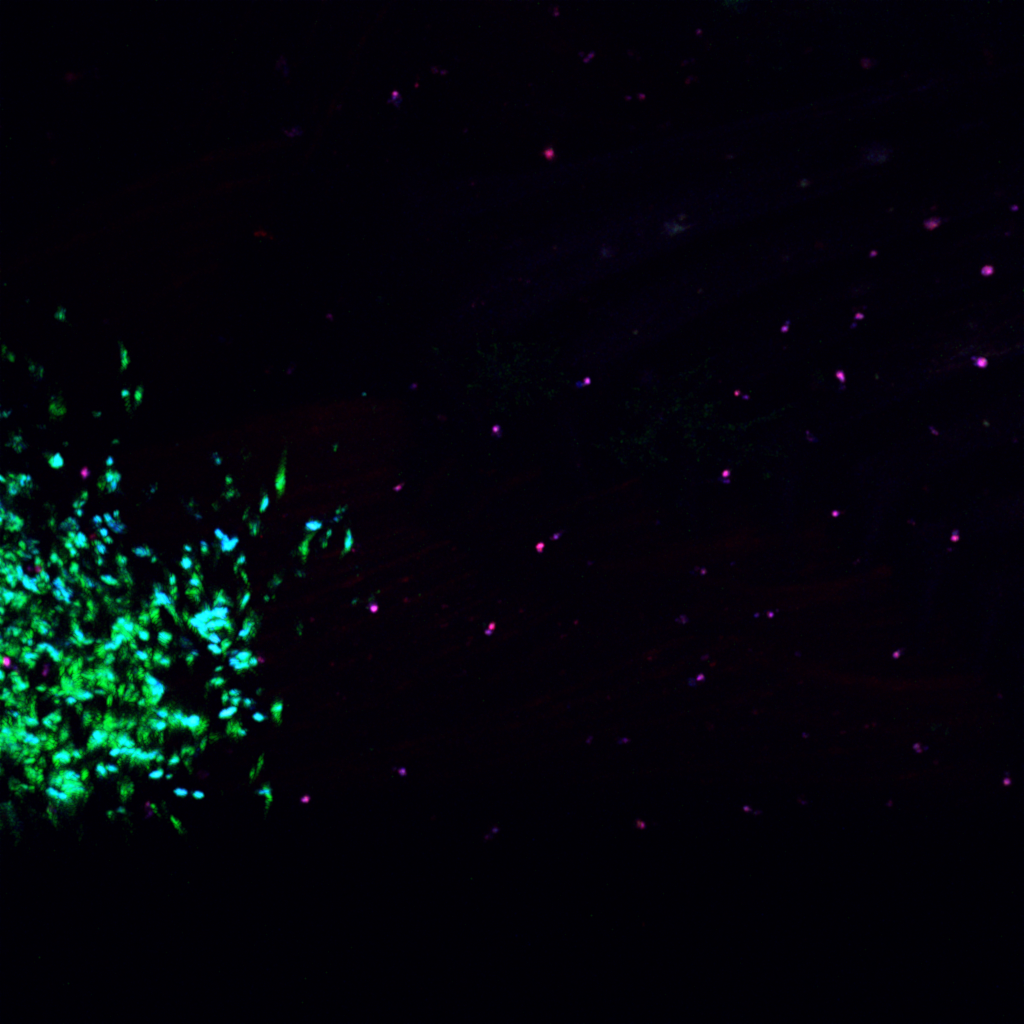

Supplement: Supplementary file 14 — Source data Fig. 5 [file 44318_2024_136_MOESM14_ESM.zip › Figure 5B/Pectoral fin-28 dpf.tif]

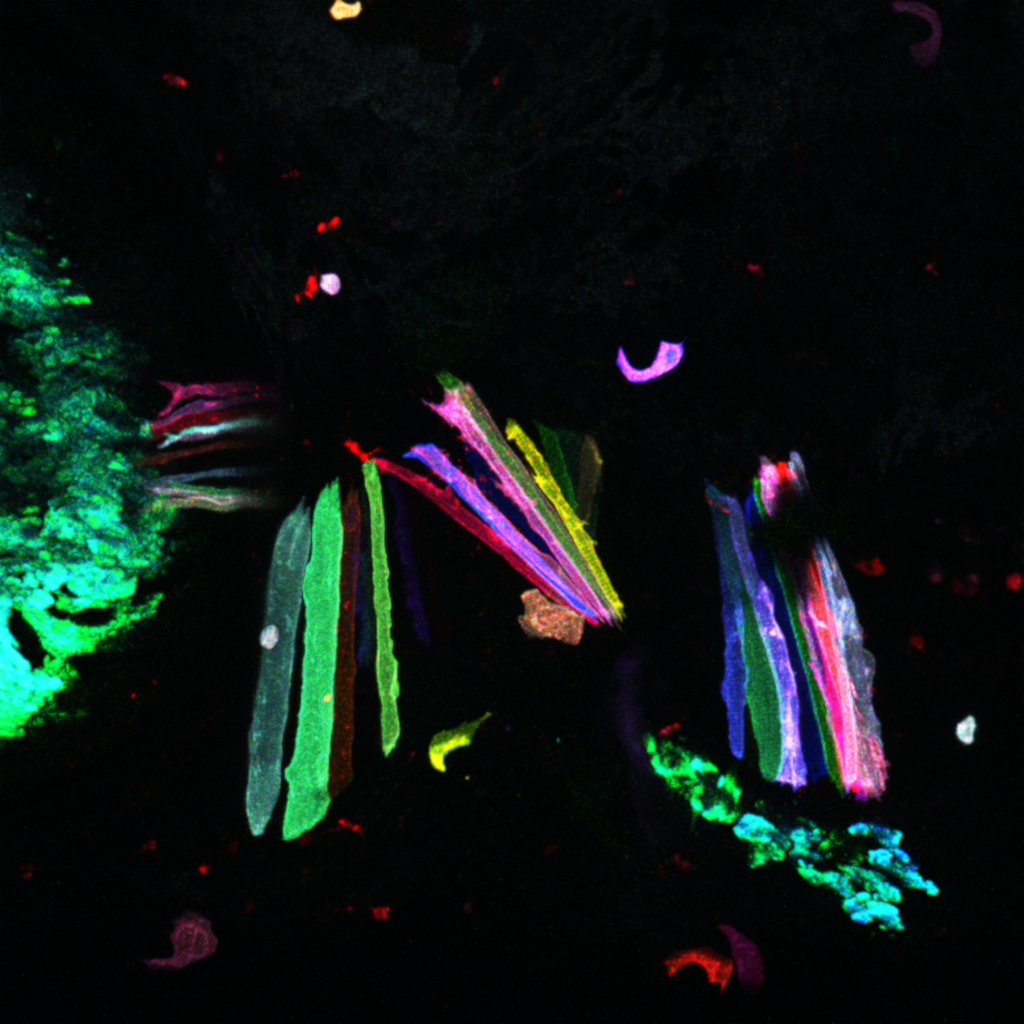

Supplement: Supplementary file 14 — Source data Fig. 5 [file 44318_2024_136_MOESM14_ESM.zip › Figure 5C/Craniofacial-14 dpf.tif]

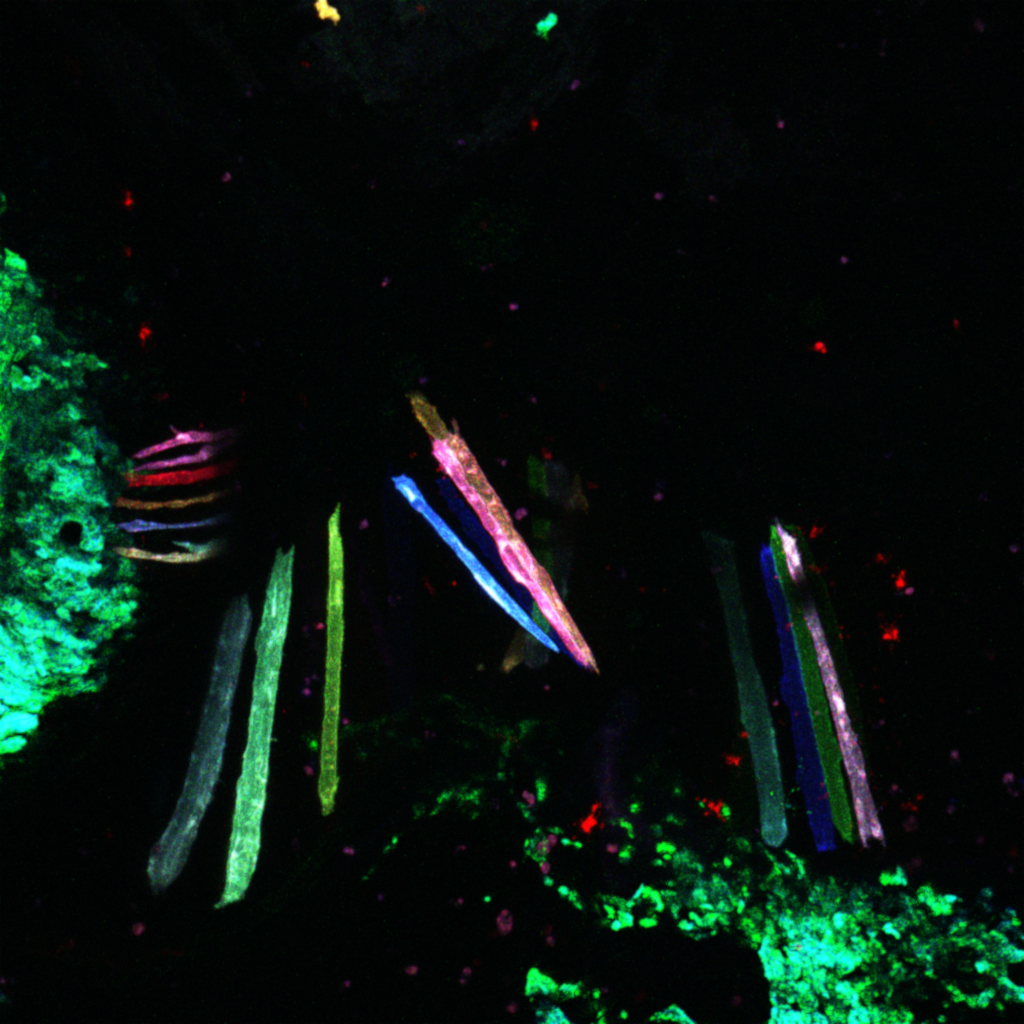

Supplement: Supplementary file 14 — Source data Fig. 5 [file 44318_2024_136_MOESM14_ESM.zip › Figure 5C/Craniofacial-21 dpf.tif]

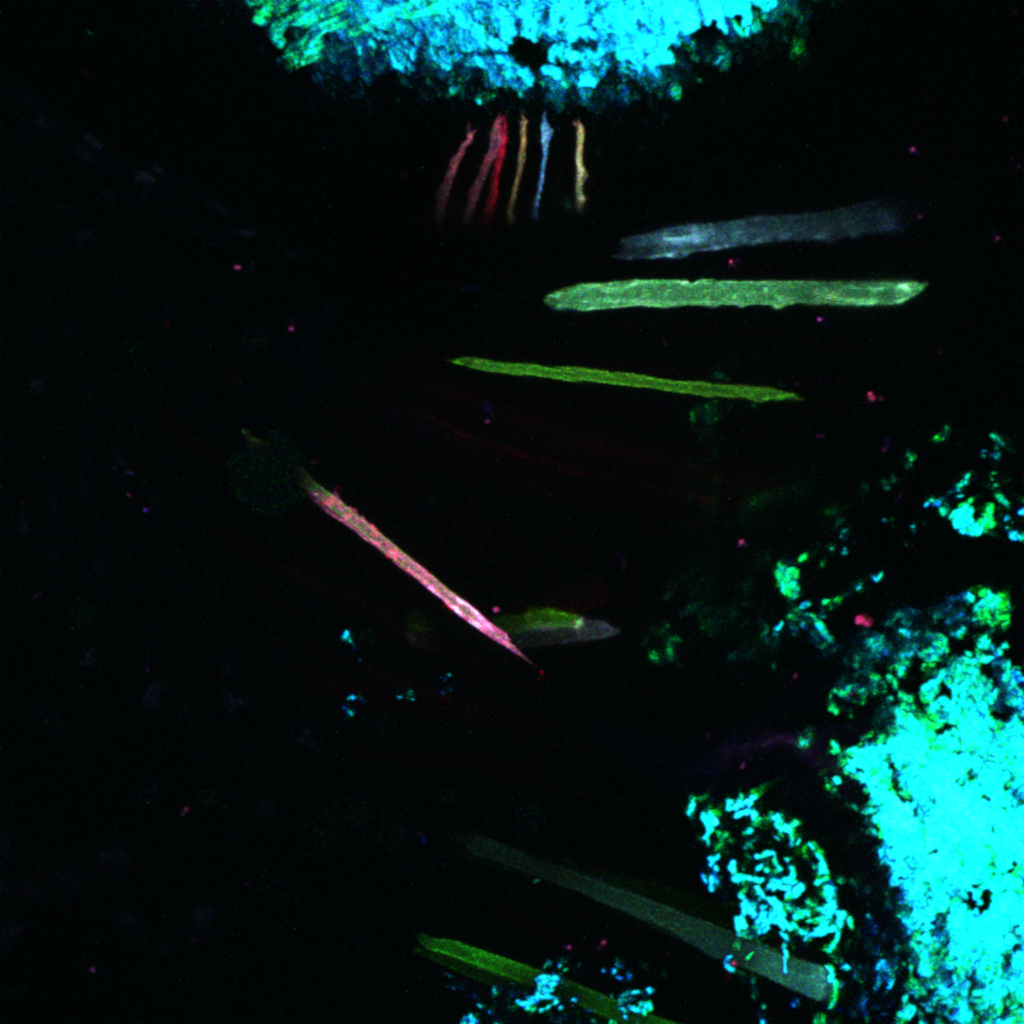

Supplement: Supplementary file 14 — Source data Fig. 5 [file 44318_2024_136_MOESM14_ESM.zip › Figure 5C/Craniofacial-28 dpf-1.tif]

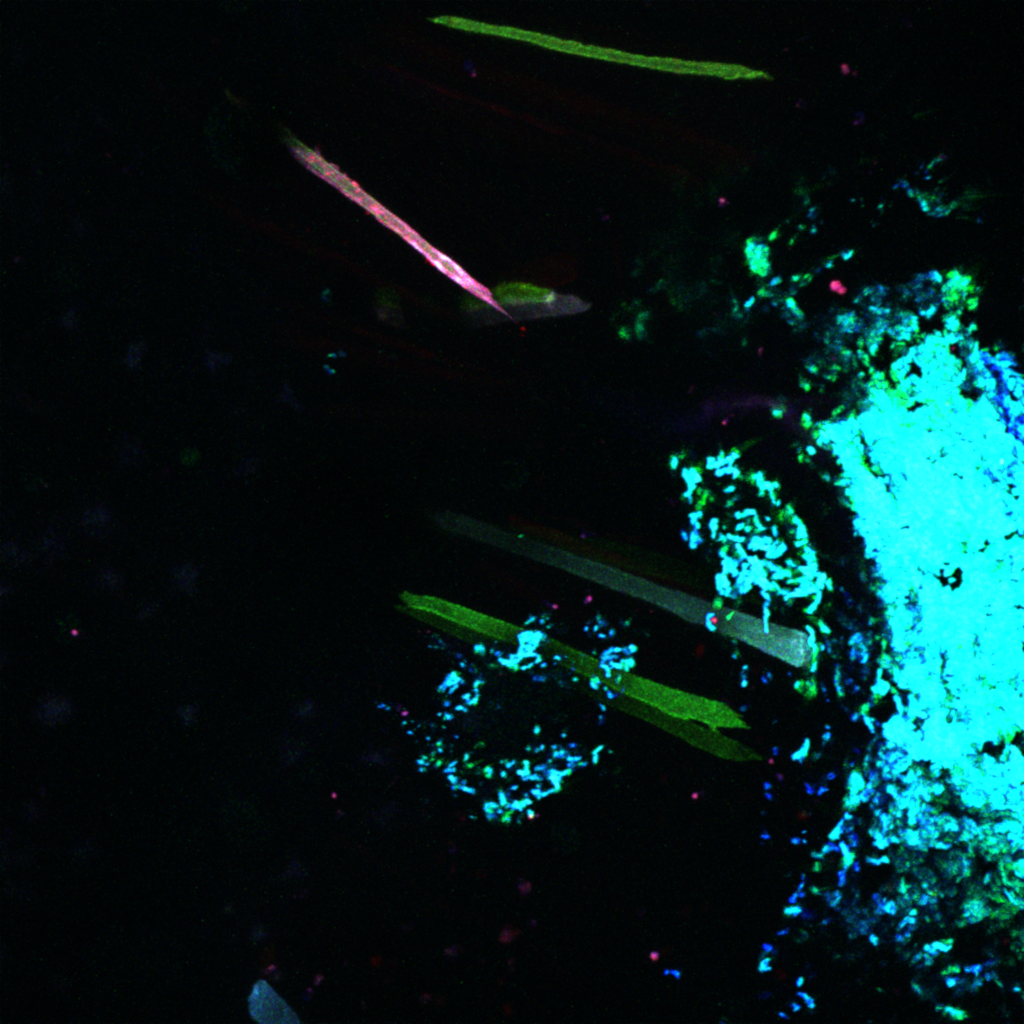

Supplement: Supplementary file 14 — Source data Fig. 5 [file 44318_2024_136_MOESM14_ESM.zip › Figure 5C/Craniofacial-28 dpf-2.tif]

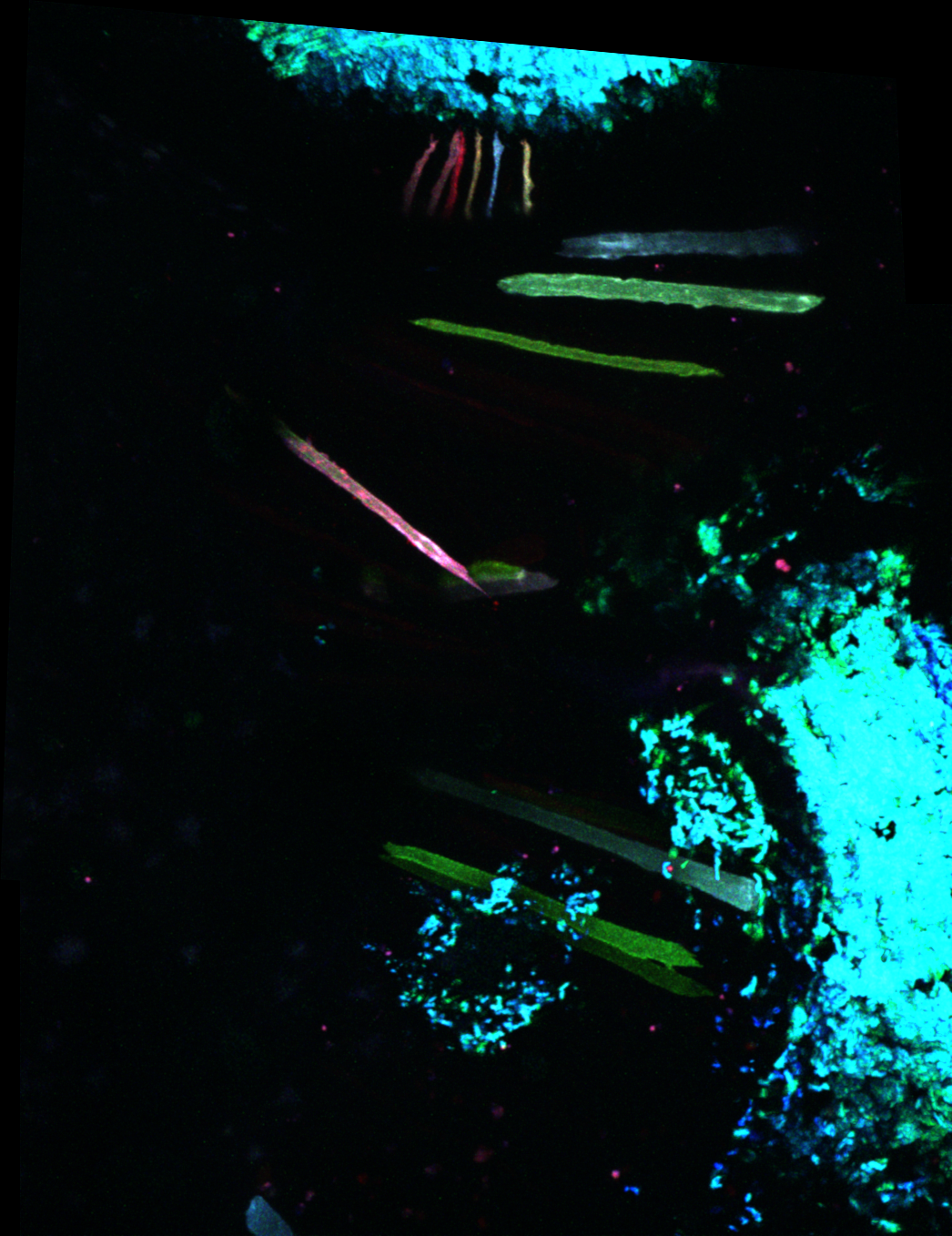

Supplement: Supplementary file 14 — Source data Fig. 5 [file 44318_2024_136_MOESM14_ESM.zip › Figure 5C/Craniofacial-28 dpf-stitched.tiff]

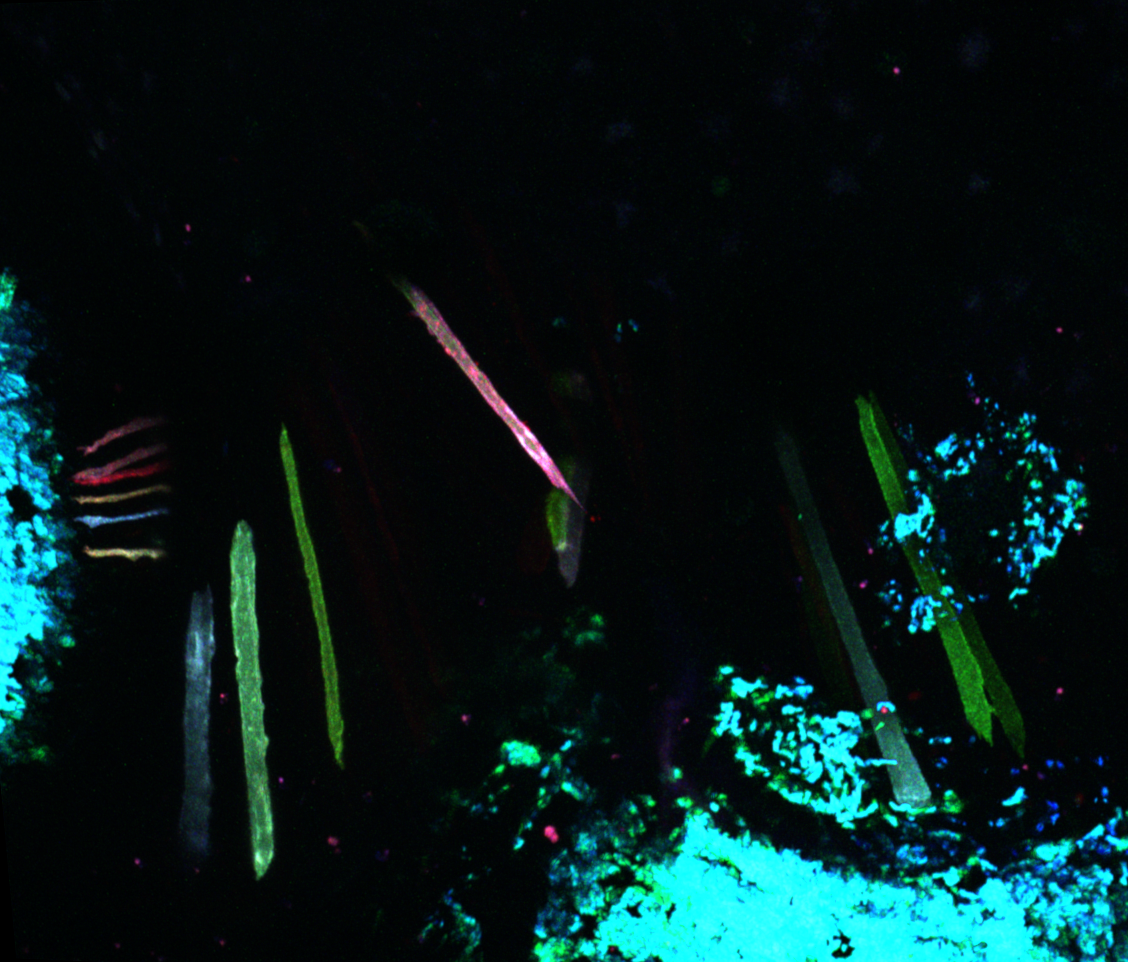

Supplement: Supplementary file 14 — Source data Fig. 5 [file 44318_2024_136_MOESM14_ESM.zip › Figure 5C/Craniofacial-28 dpf-stitched-crop.tif]

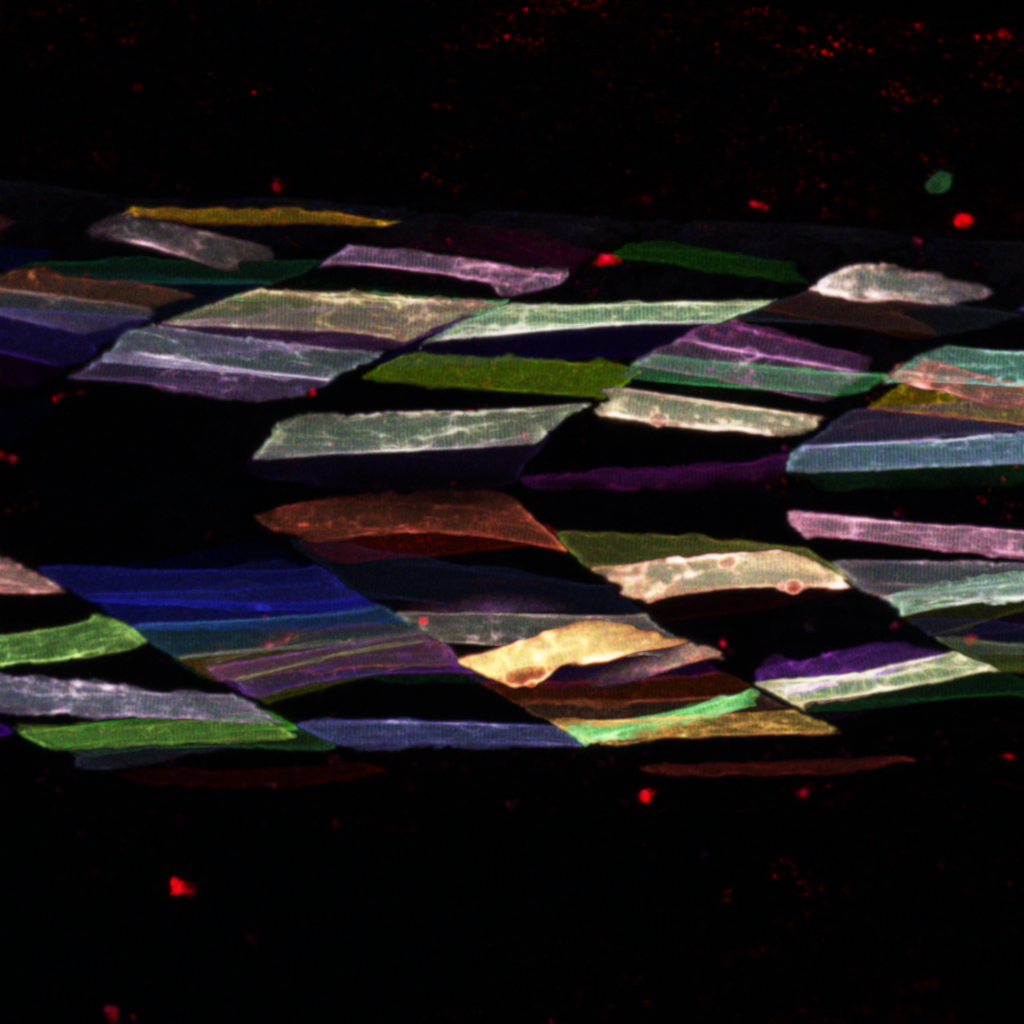

Supplement: Supplementary file 14 — Source data Fig. 5 [file 44318_2024_136_MOESM14_ESM.zip › Figure 5D/Trunk-14 dpf.tif]

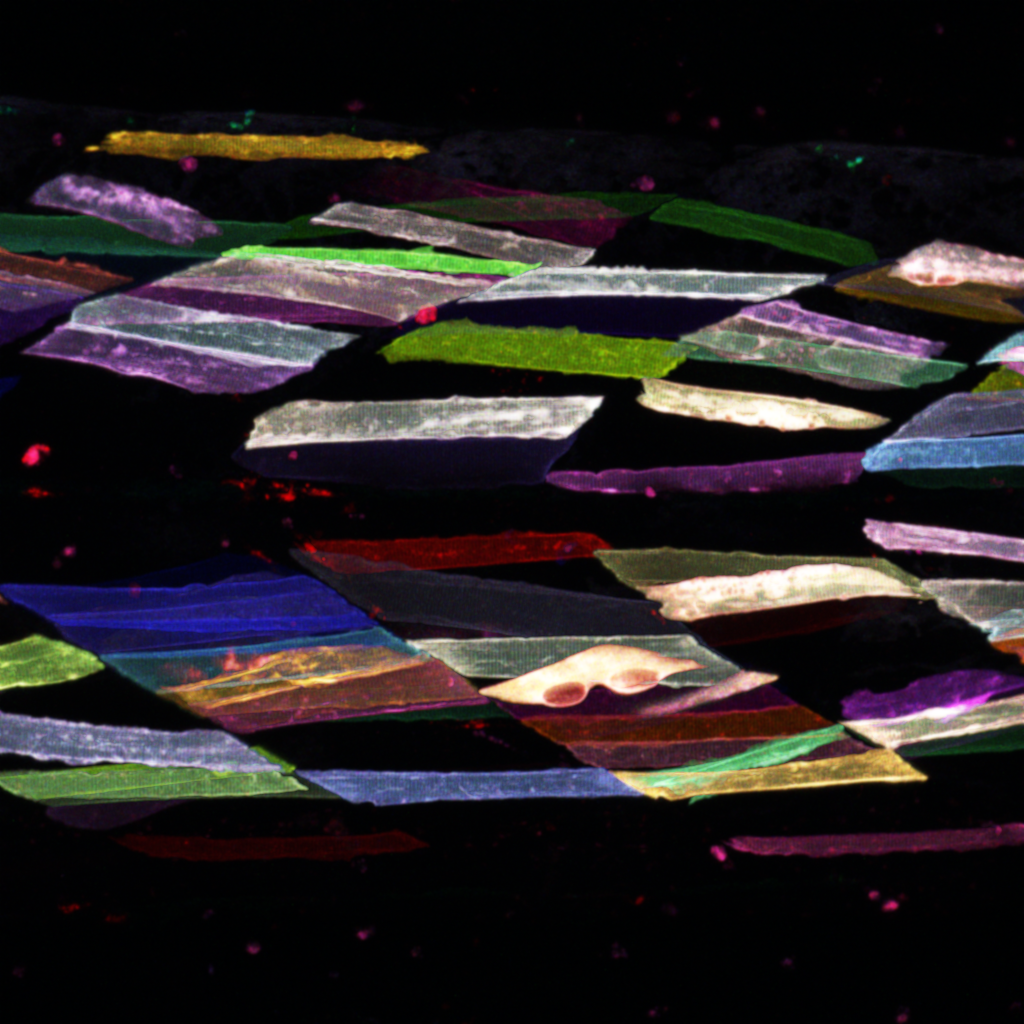

Supplement: Supplementary file 14 — Source data Fig. 5 [file 44318_2024_136_MOESM14_ESM.zip › Figure 5D/Trunk-18 dpf.tif]

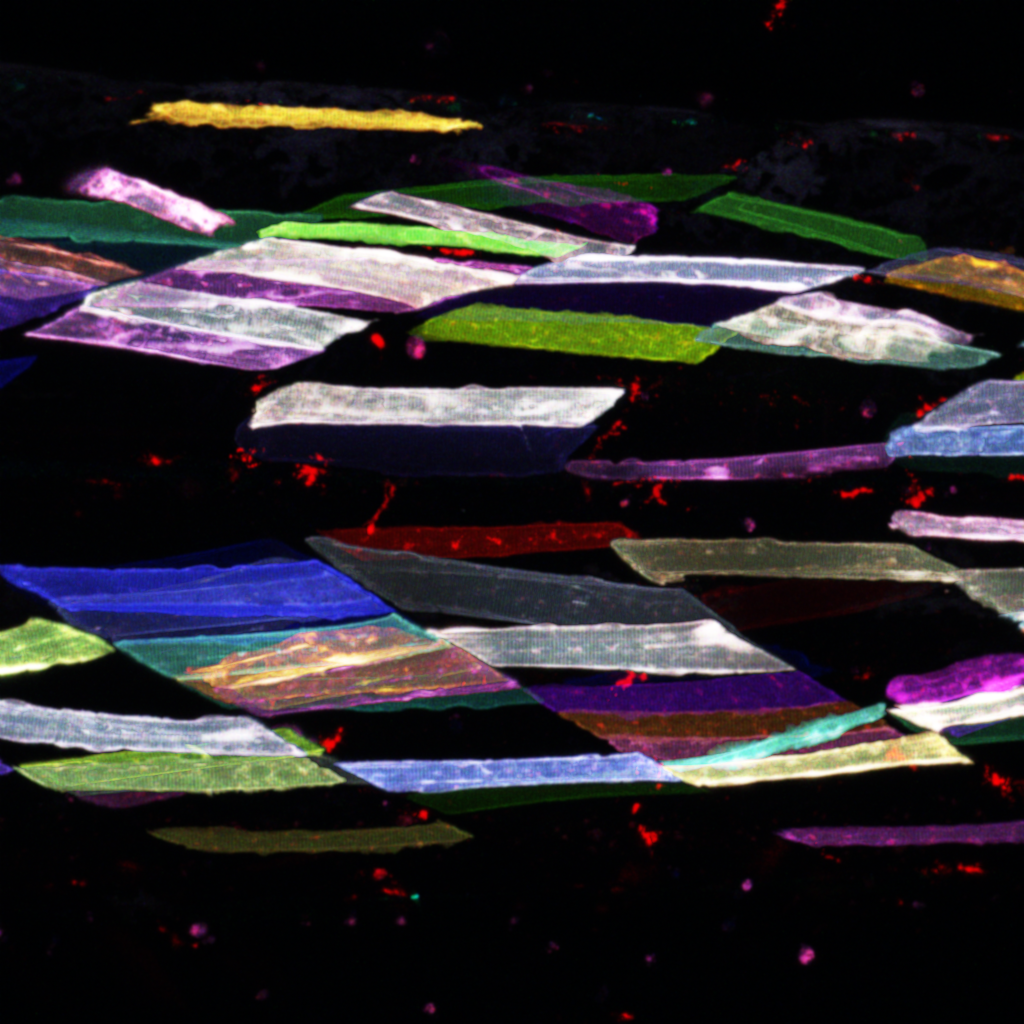

Supplement: Supplementary file 14 — Source data Fig. 5 [file 44318_2024_136_MOESM14_ESM.zip › Figure 5D/Trunk-21 dpf.tif]

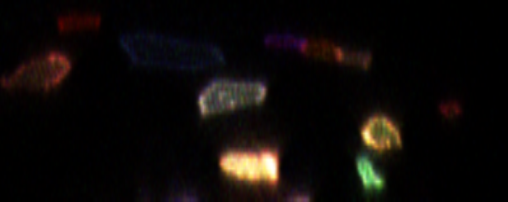

Supplement: Supplementary file 14 — Source data Fig. 5 [file 44318_2024_136_MOESM14_ESM.zip › Figure 5D/Trunk-cross-section-14 dpf.tif]

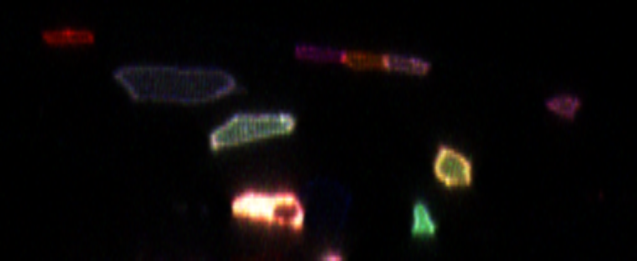

Supplement: Supplementary file 14 — Source data Fig. 5 [file 44318_2024_136_MOESM14_ESM.zip › Figure 5D/Trunk-cross-section-18 dpf.tif]

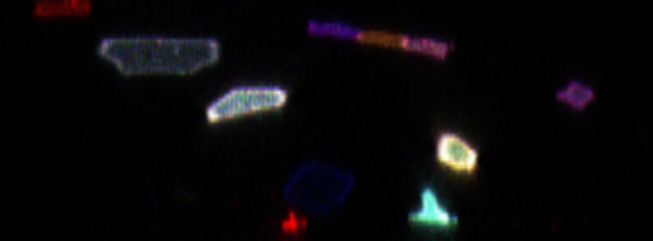

Supplement: Supplementary file 14 — Source data Fig. 5 [file 44318_2024_136_MOESM14_ESM.zip › Figure 5D/Trunk-cross-section-21 dpf.tif]

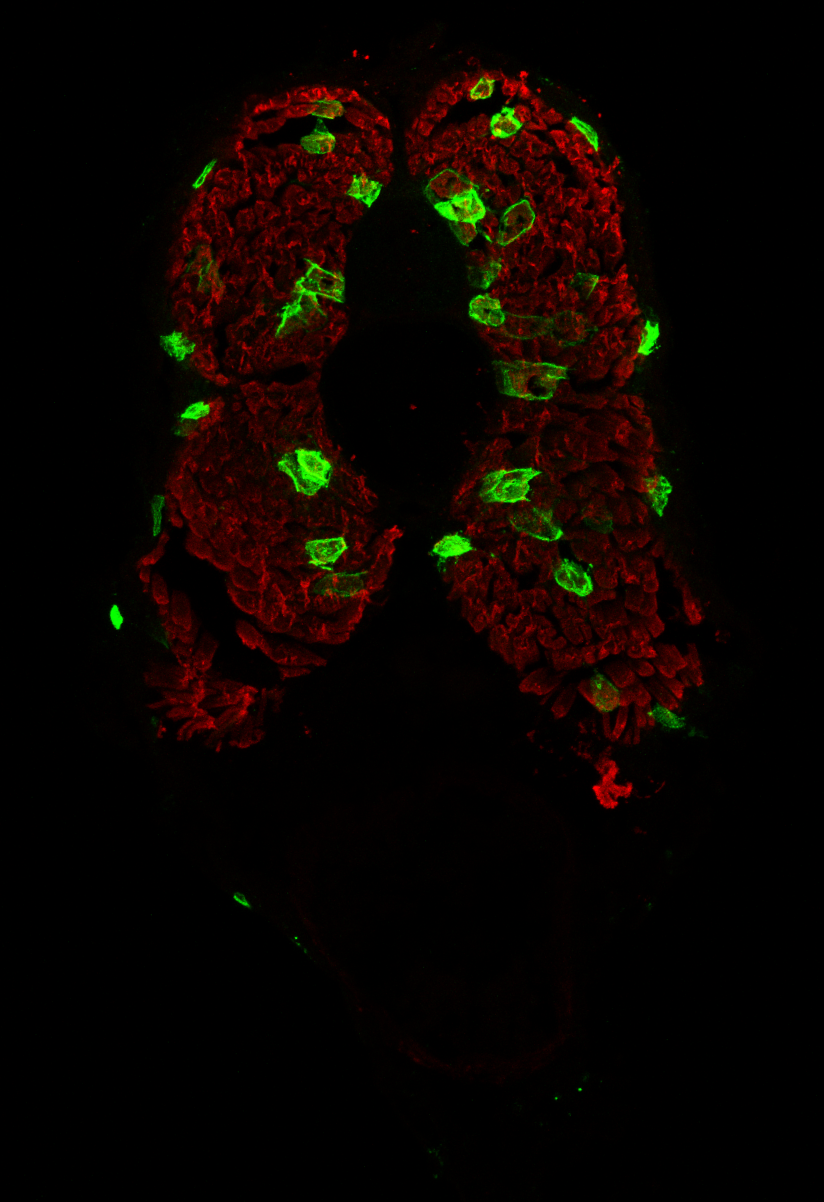

Supplement: Supplementary file 14 — Source data Fig. 5 [file 44318_2024_136_MOESM14_ESM.zip › Figure 5H/Trunk section-DT-14 dpf.tif]

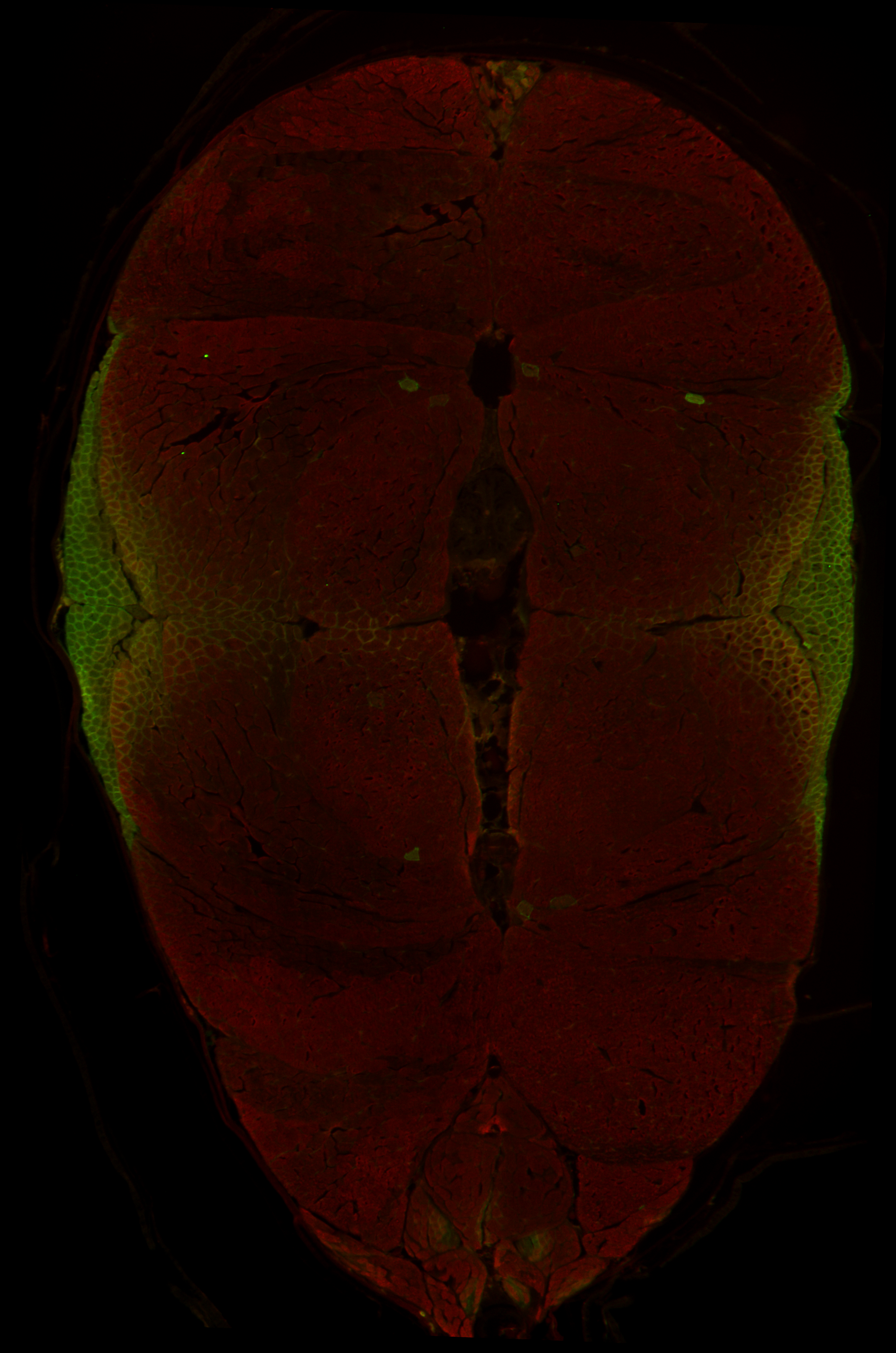

Supplement: Supplementary file 14 — Source data Fig. 5 [file 44318_2024_136_MOESM14_ESM.zip › Figure 5H/Trunk section-DT-180 dpf.tif]

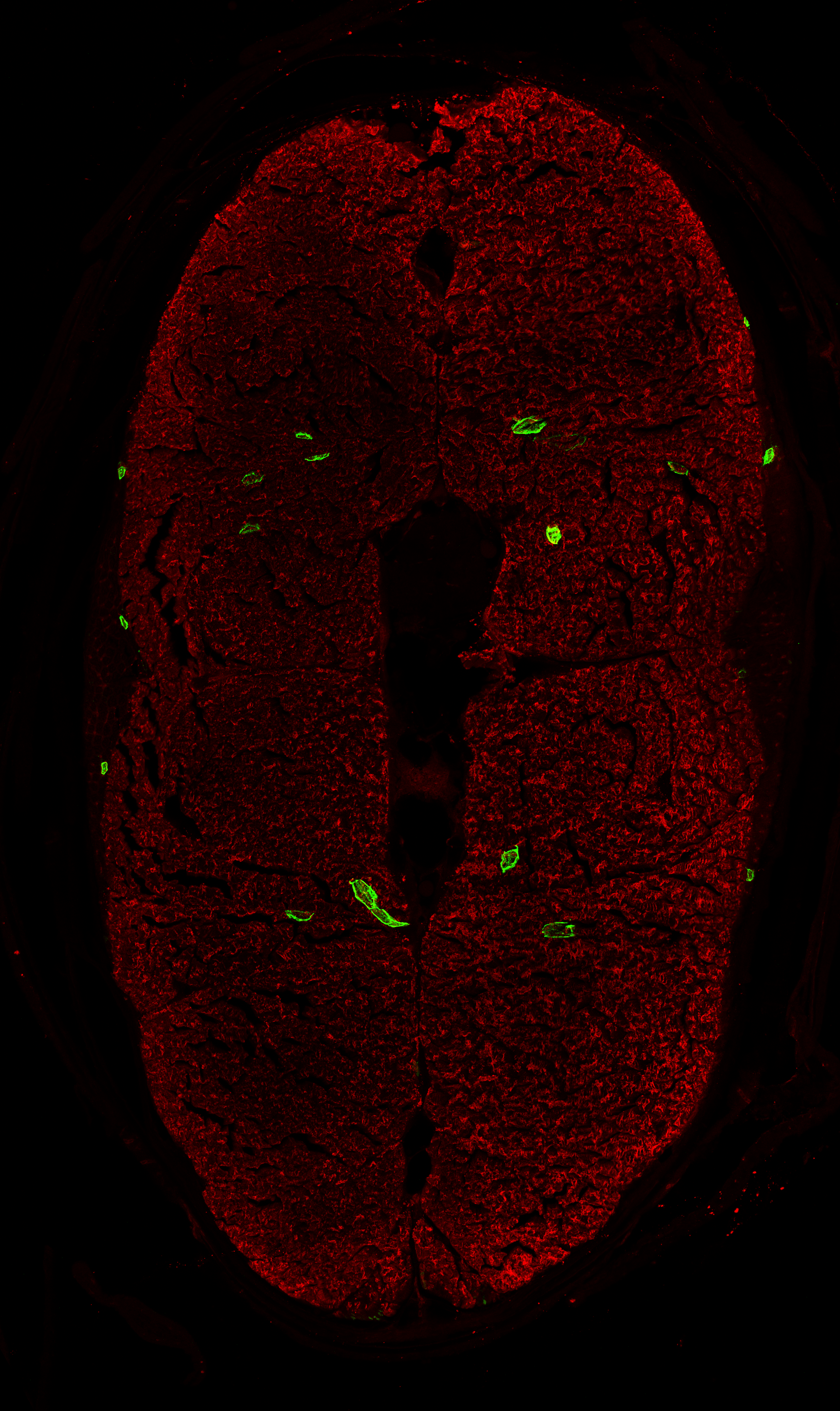

Supplement: Supplementary file 14 — Source data Fig. 5 [file 44318_2024_136_MOESM14_ESM.zip › Figure 5H/Trunk section-DT-42 dpf.tif]

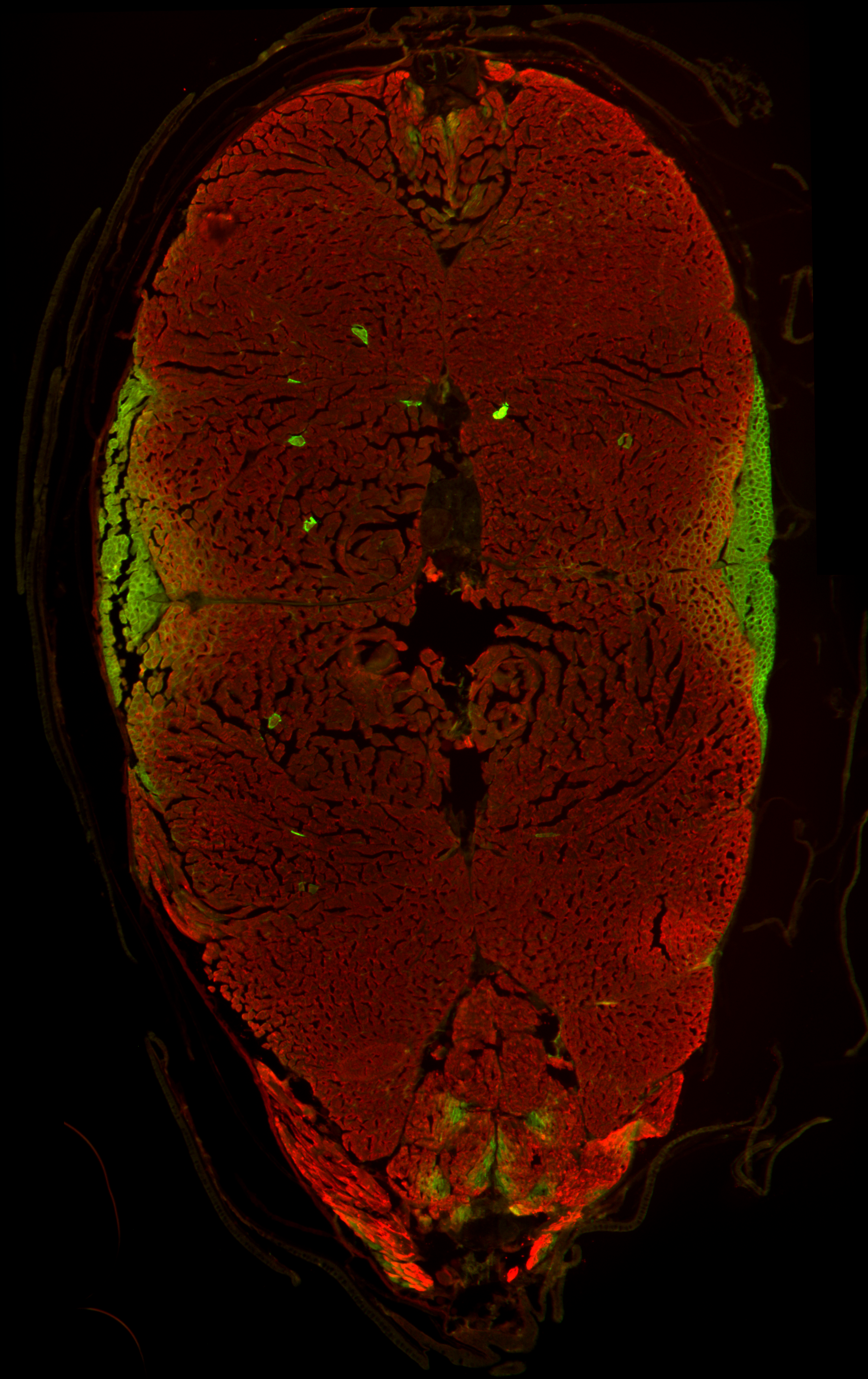

Supplement: Supplementary file 14 — Source data Fig. 5 [file 44318_2024_136_MOESM14_ESM.zip › Figure 5H/Trunk section-DT-70 dpf.tif]

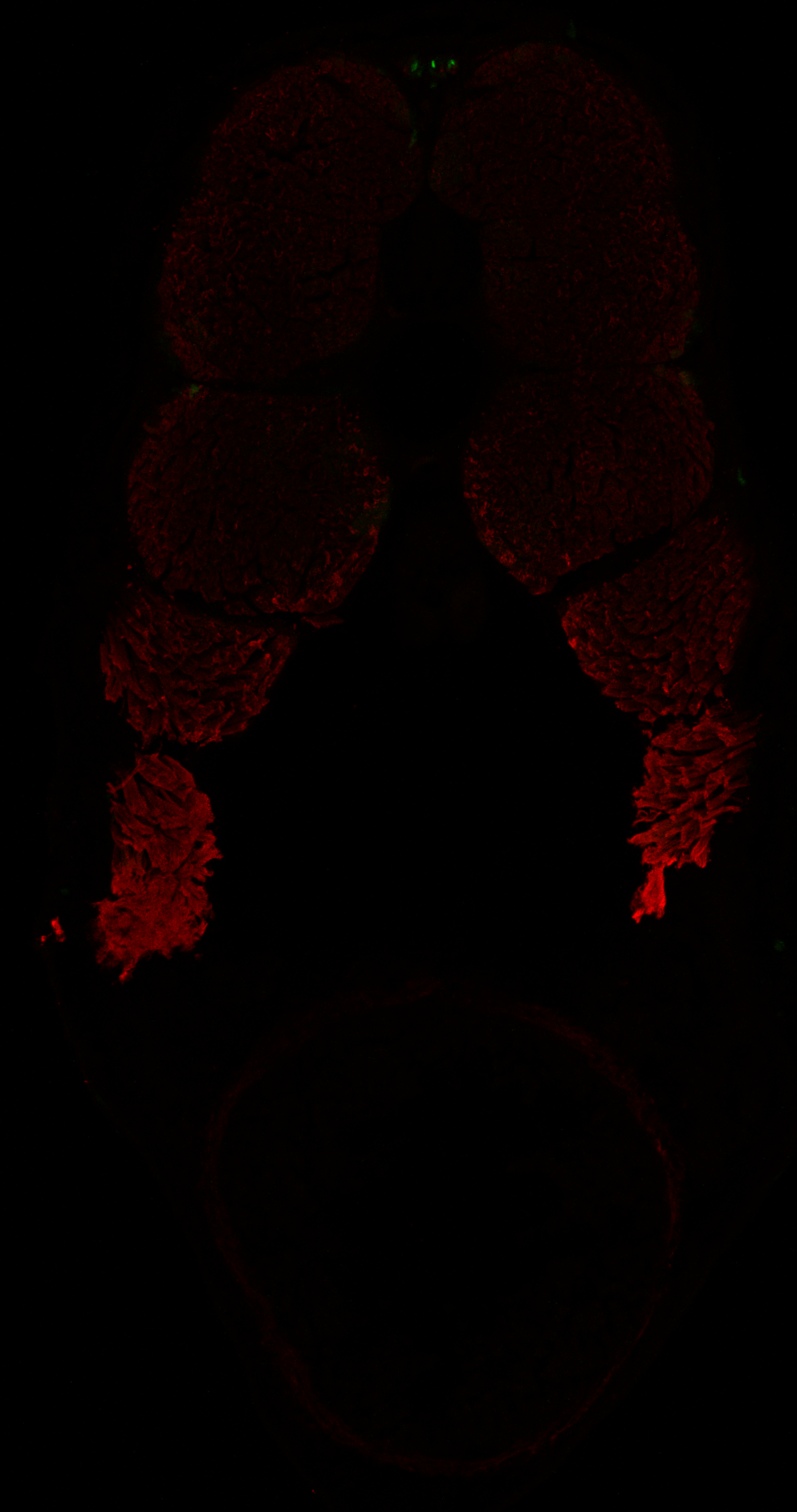

Supplement: Supplementary file 14 — Source data Fig. 5 [file 44318_2024_136_MOESM14_ESM.zip › Figure 5H/Trunk section-no treatment-14 dpf.tif]

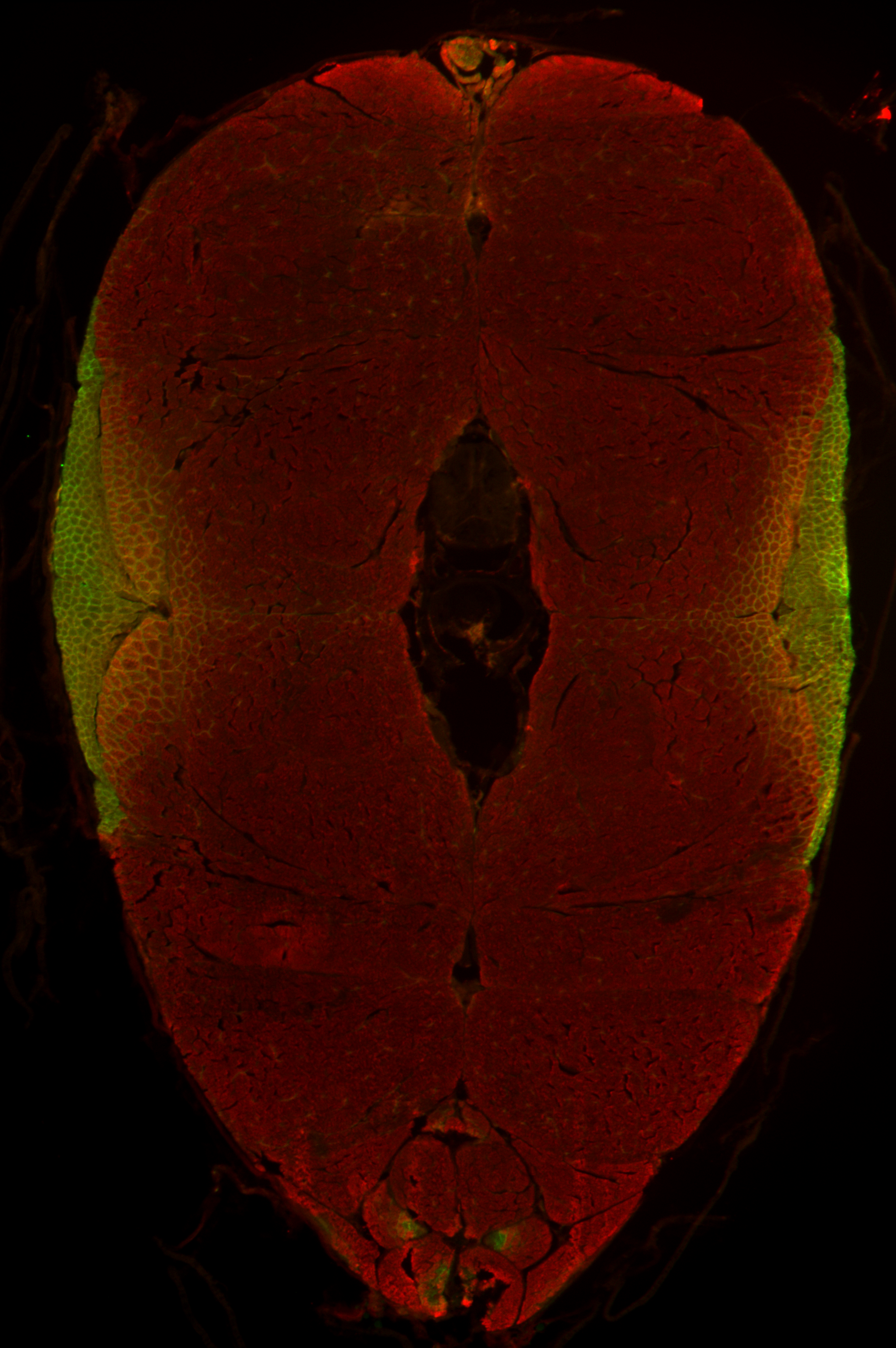

Supplement: Supplementary file 14 — Source data Fig. 5 [file 44318_2024_136_MOESM14_ESM.zip › Figure 5H/Trunk section-no treatment-180 dpf.tif]

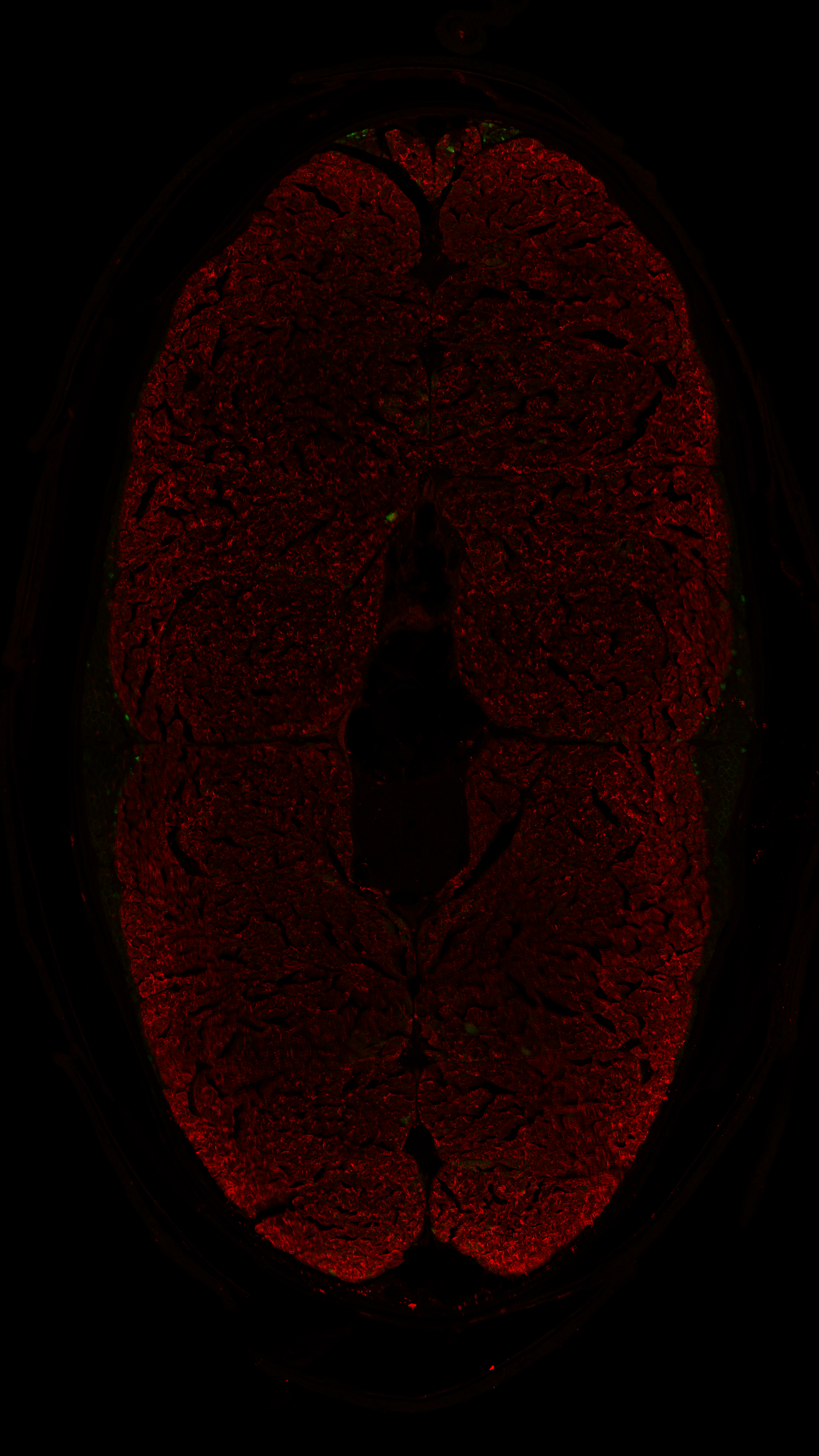

Supplement: Supplementary file 14 — Source data Fig. 5 [file 44318_2024_136_MOESM14_ESM.zip › Figure 5H/Trunk section-no treatment-42 dpf.tif]

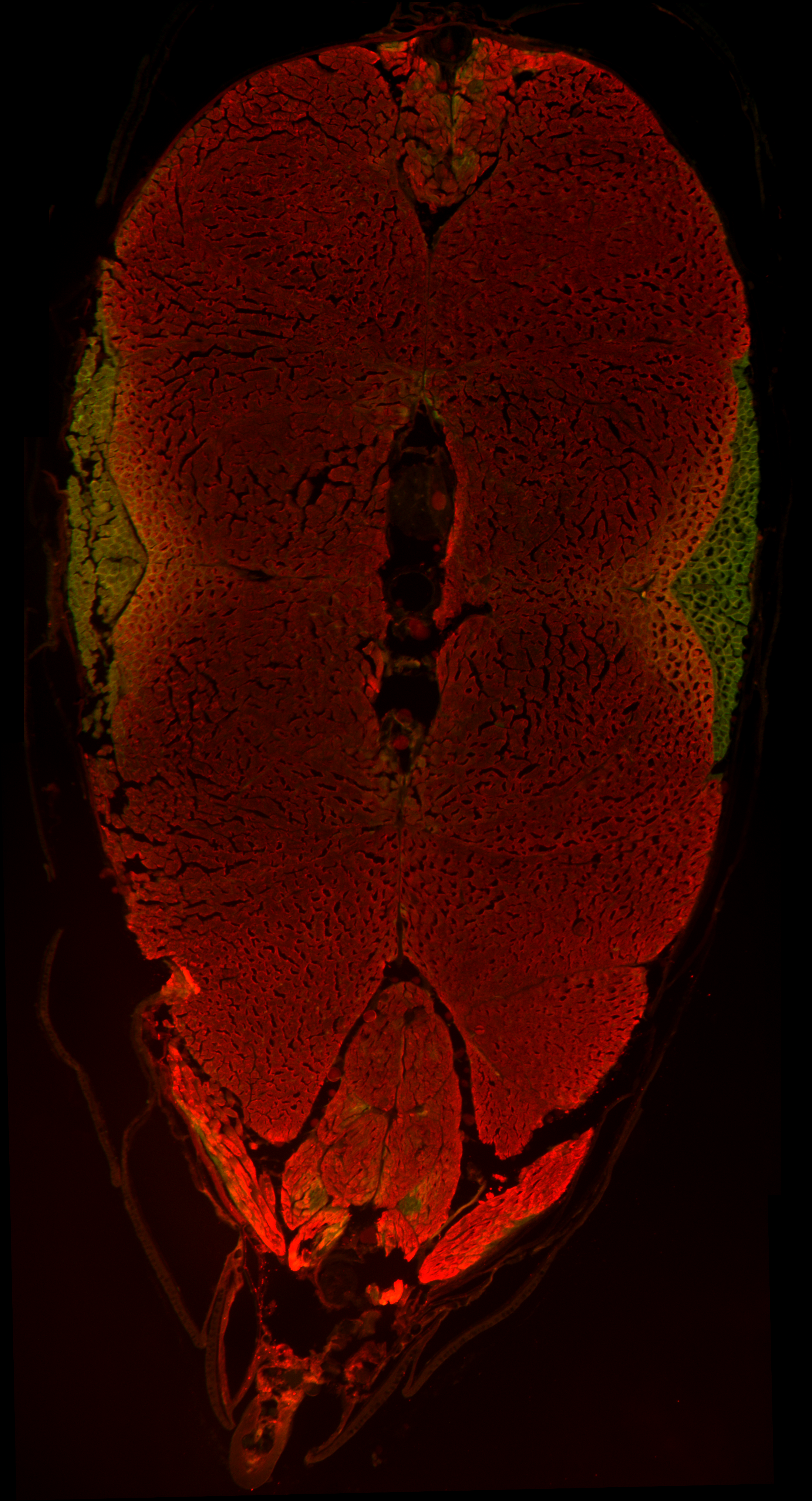

Supplement: Supplementary file 14 — Source data Fig. 5 [file 44318_2024_136_MOESM14_ESM.zip › Figure 5H/Trunk section-no treatment-70 dpf.tif]

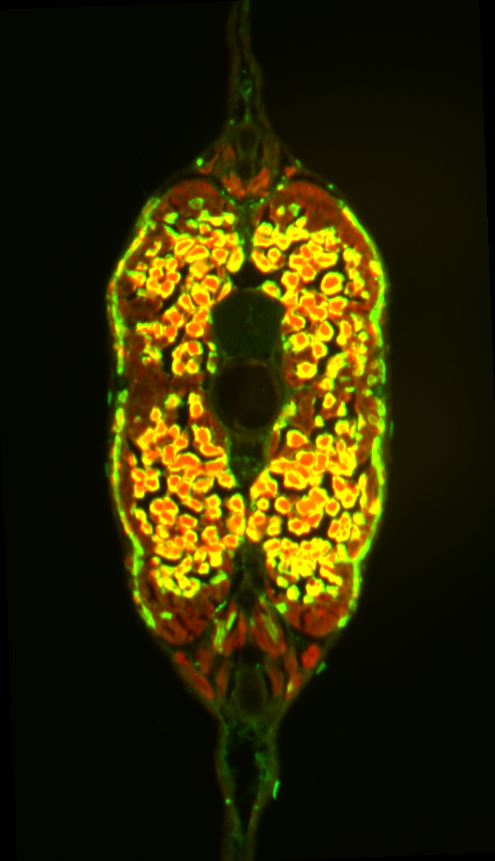

Supplement: Supplementary file 14 — Source data Fig. 5 [file 44318_2024_136_MOESM14_ESM.zip › Figure 5L/Trunk section-DT-29 dpf.tif]

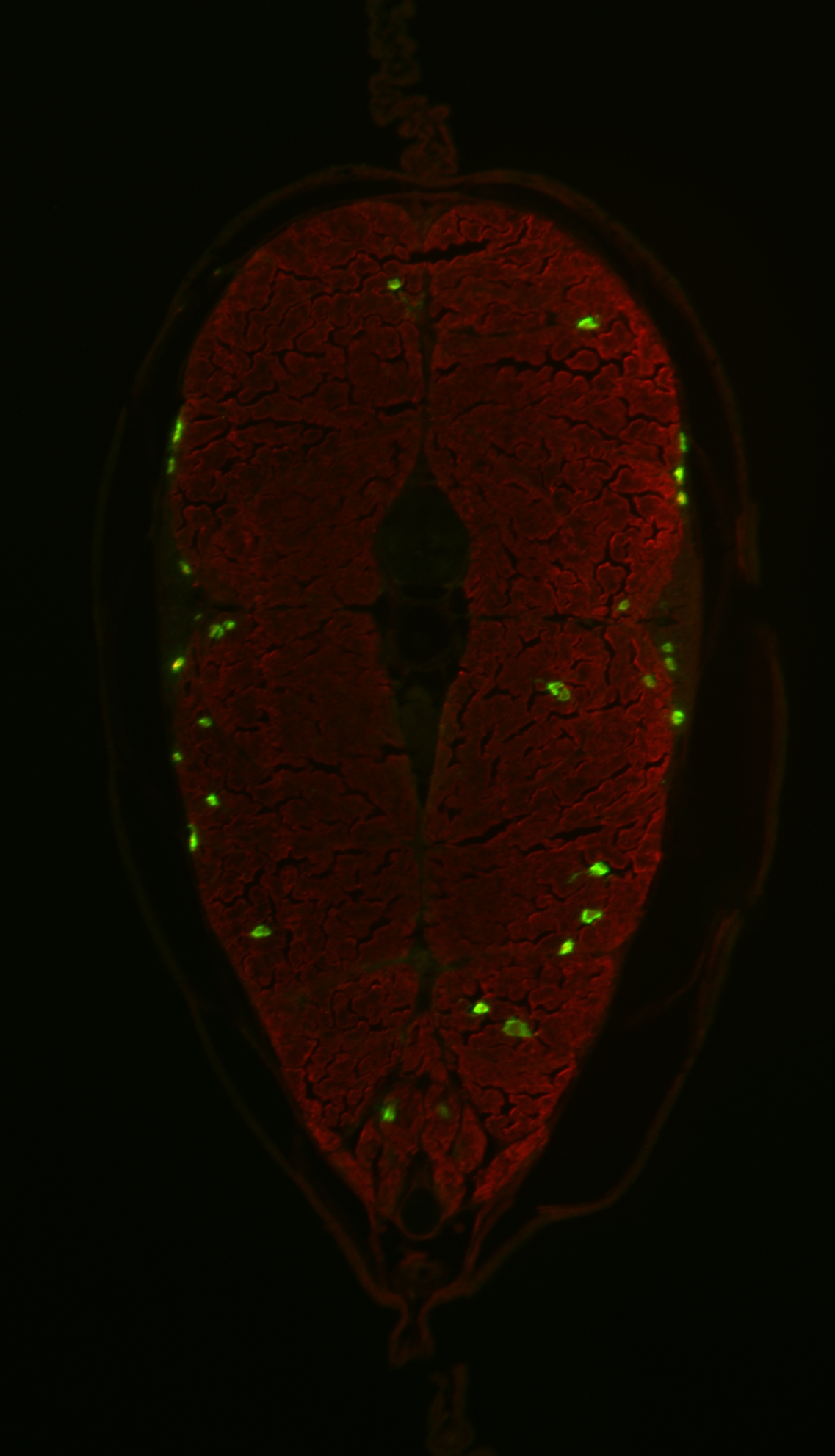

Supplement: Supplementary file 14 — Source data Fig. 5 [file 44318_2024_136_MOESM14_ESM.zip › Figure 5L/Trunk section-DT-42 dpf.tif]

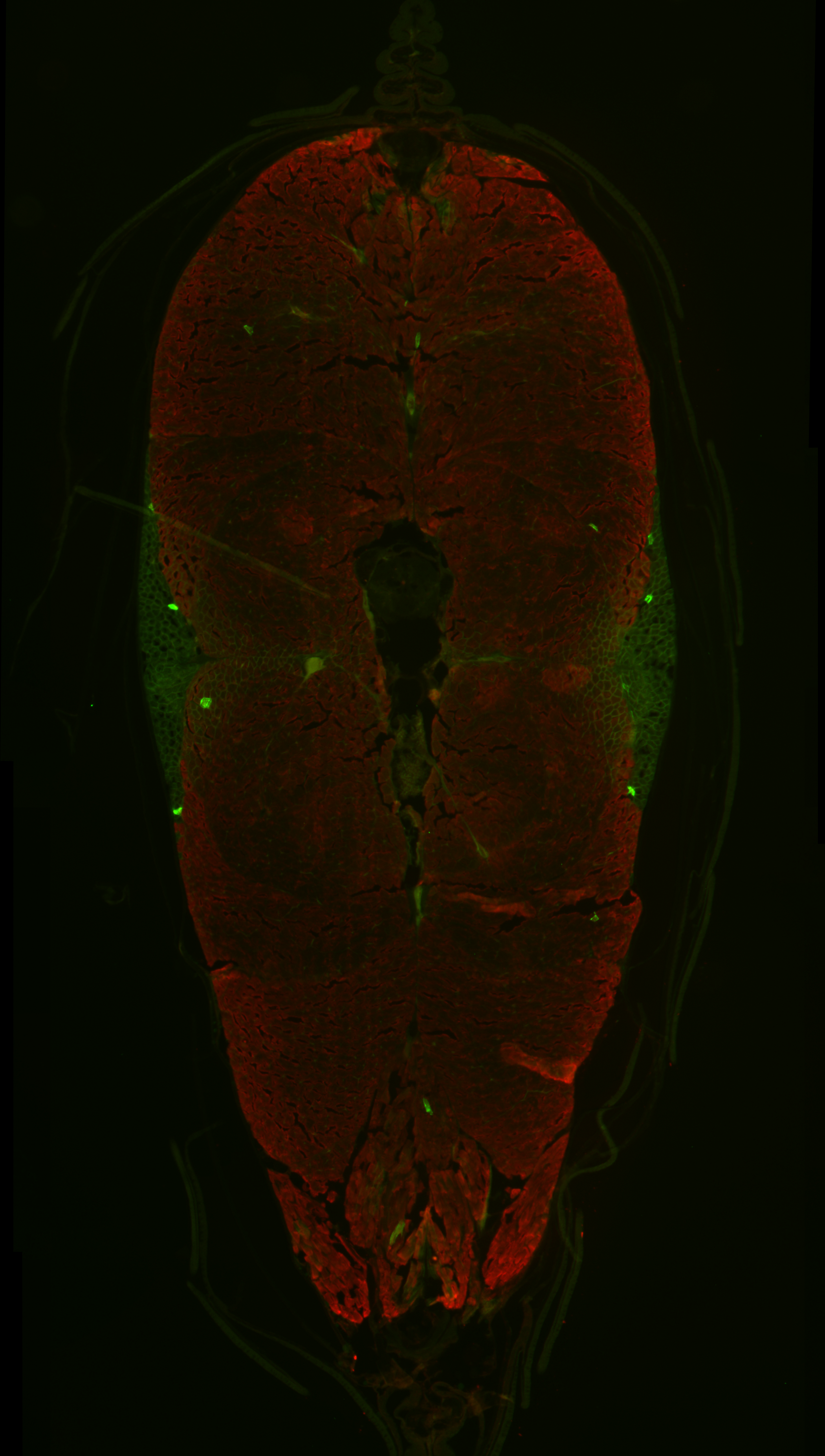

Supplement: Supplementary file 14 — Source data Fig. 5 [file 44318_2024_136_MOESM14_ESM.zip › Figure 5L/Trunk section-DT-70 dpf.tif]

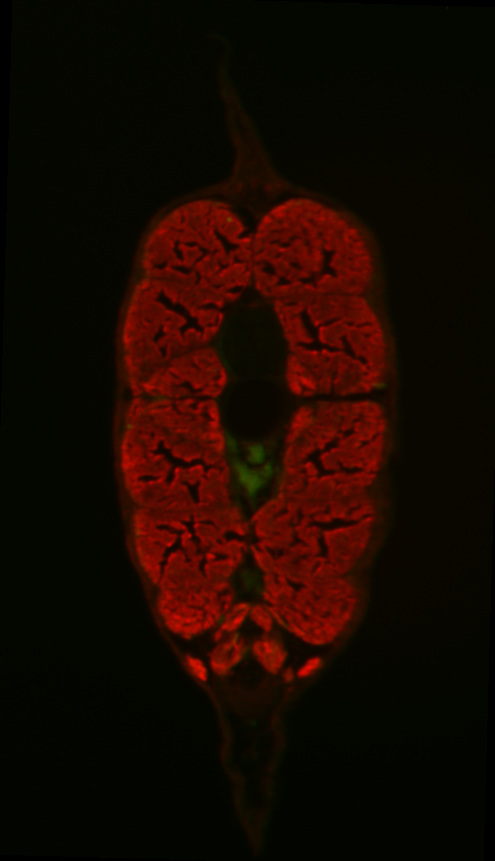

Supplement: Supplementary file 14 — Source data Fig. 5 [file 44318_2024_136_MOESM14_ESM.zip › Figure 5L/Trunk section-no treatment-29 dpf.tif]

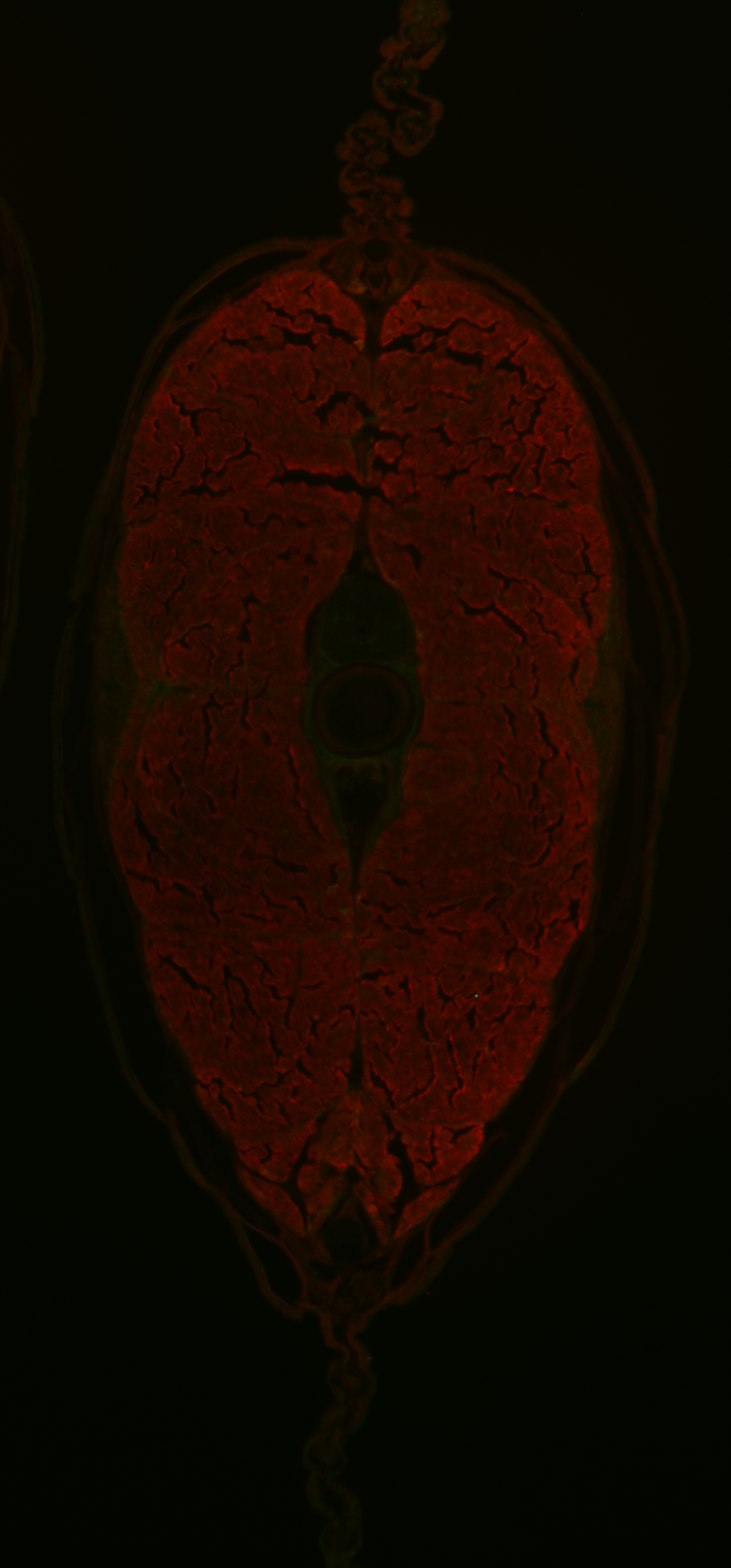

Supplement: Supplementary file 14 — Source data Fig. 5 [file 44318_2024_136_MOESM14_ESM.zip › Figure 5L/Trunk section-no treatment-42 dpf.tif]

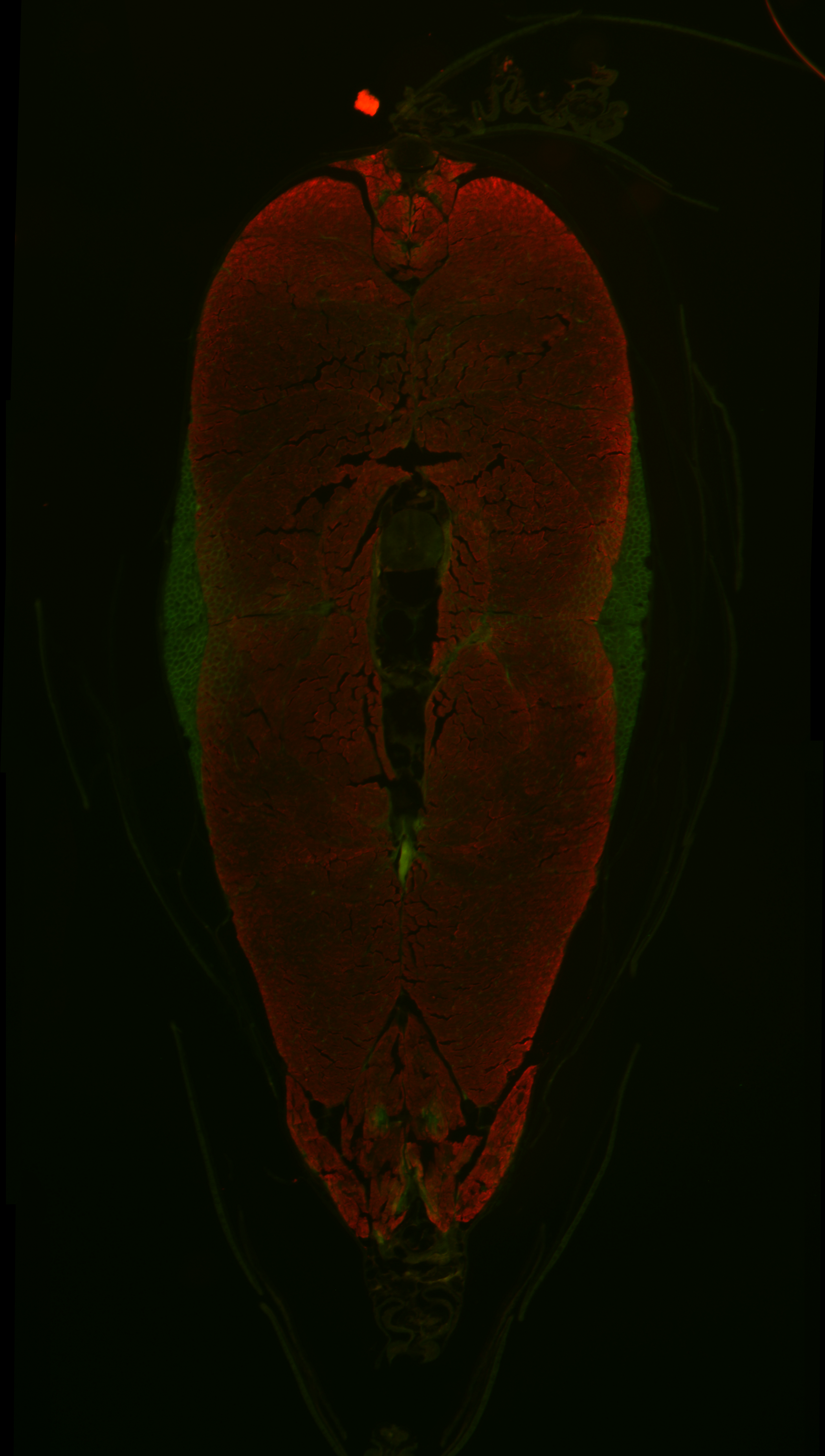

Supplement: Supplementary file 14 — Source data Fig. 5 [file 44318_2024_136_MOESM14_ESM.zip › Figure 5L/Trunk section-no treatment-70 dpf.tif]

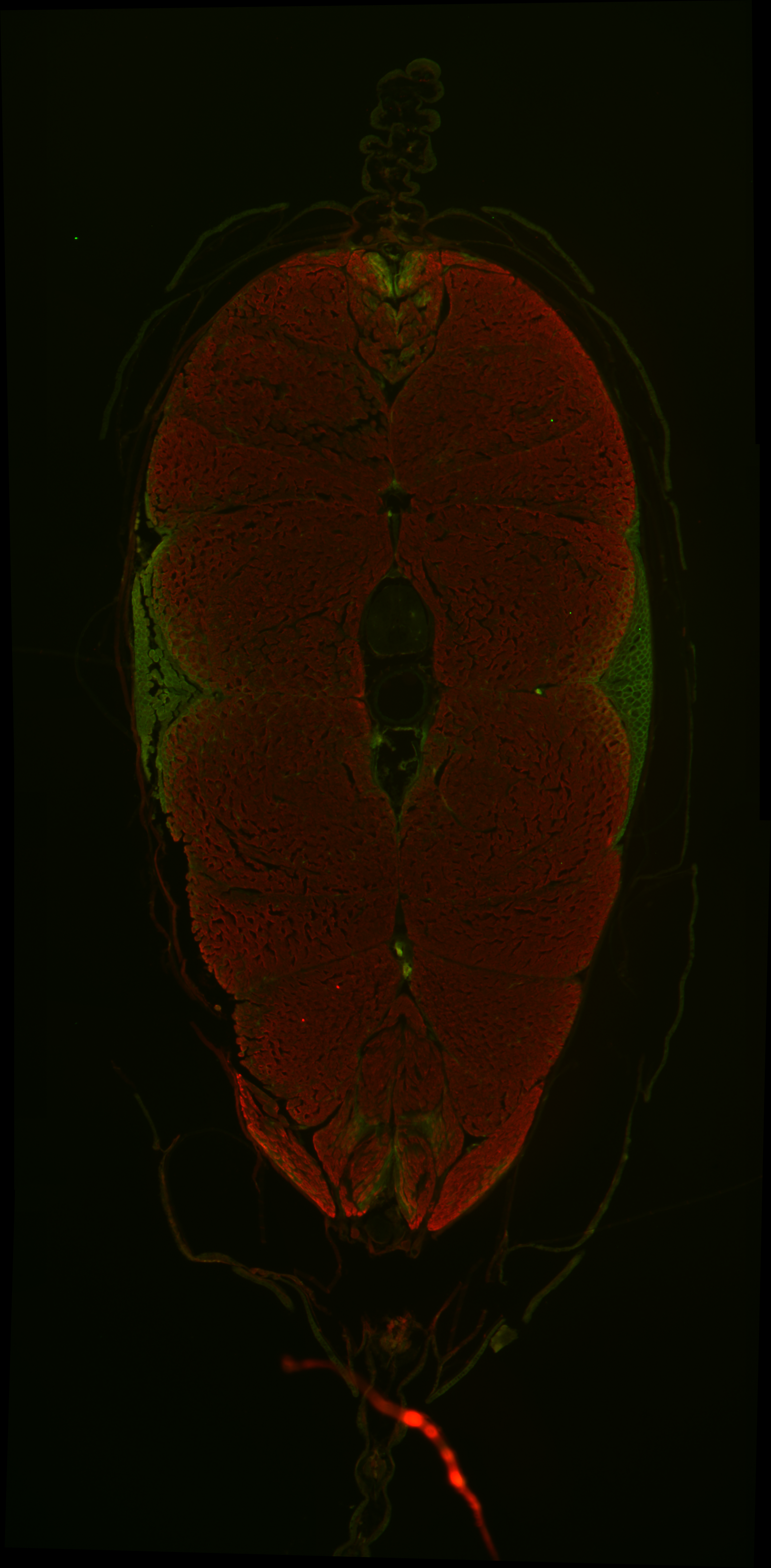

Supplement: Supplementary file 14 — Source data Fig. 5 [file 44318_2024_136_MOESM14_ESM.zip › Figure 5P/Cross-section-no treatment-10 mpf.tif]

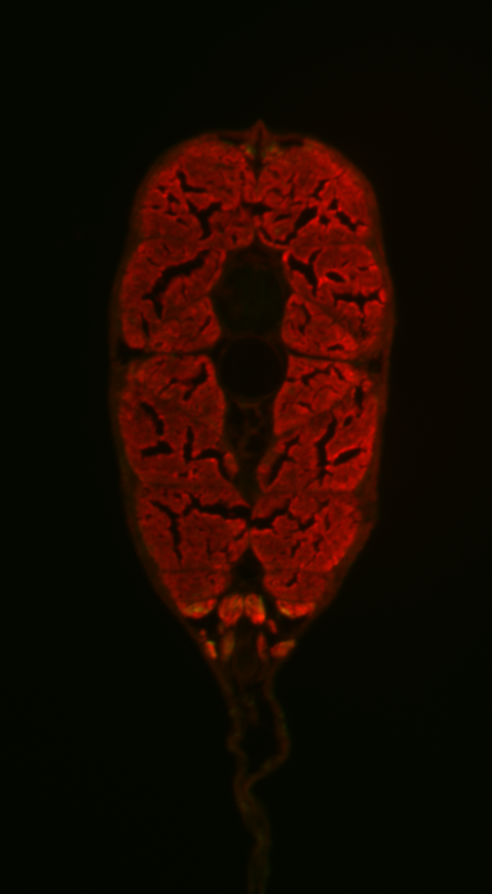

Supplement: Supplementary file 14 — Source data Fig. 5 [file 44318_2024_136_MOESM14_ESM.zip › Figure 5P/Cross-section-no treatment-29 dpf.tif]

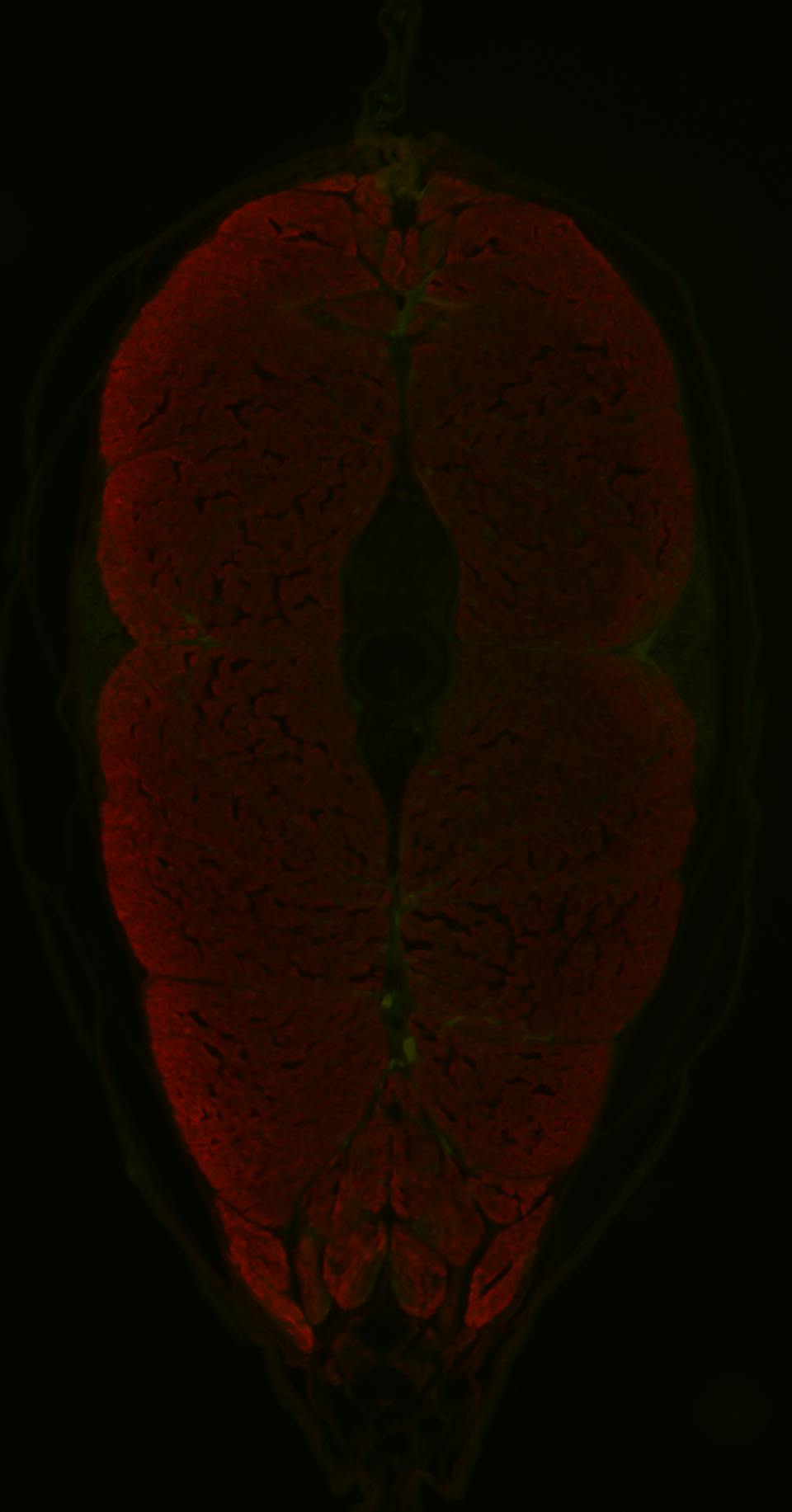

Supplement: Supplementary file 14 — Source data Fig. 5 [file 44318_2024_136_MOESM14_ESM.zip › Figure 5P/Cross-section-no treatment-43 dpf.tif]

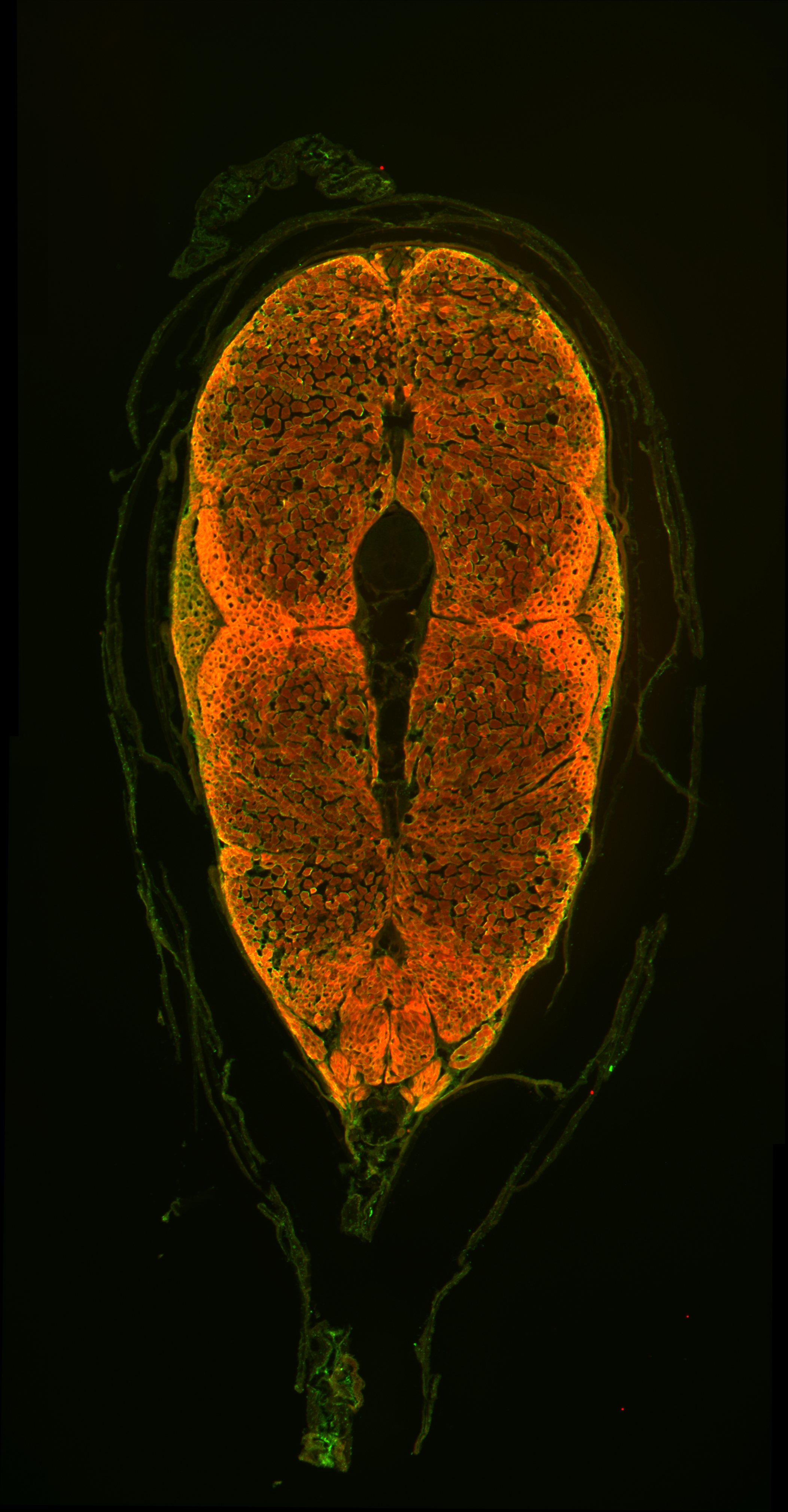

Supplement: Supplementary file 14 — Source data Fig. 5 [file 44318_2024_136_MOESM14_ESM.zip › Figure 5Q/Cross-section-DT-10 mpf.tif]

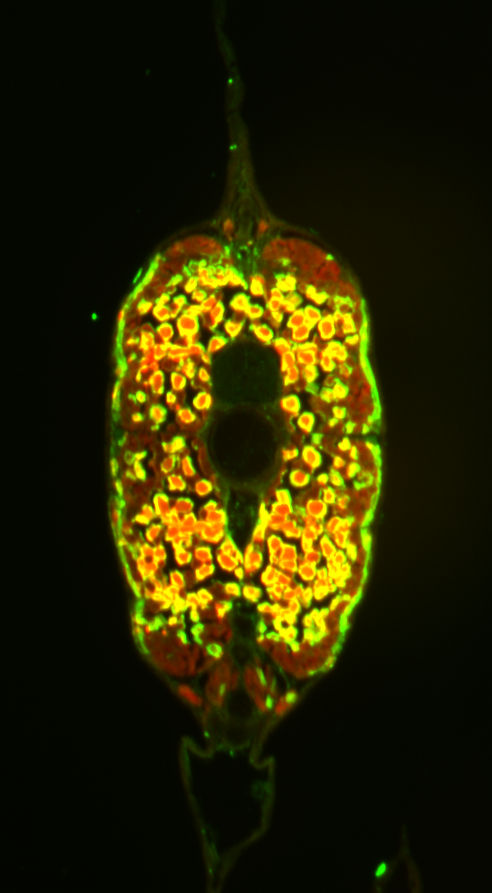

Supplement: Supplementary file 14 — Source data Fig. 5 [file 44318_2024_136_MOESM14_ESM.zip › Figure 5Q/Cross-section-DT-29 dpf.tif]

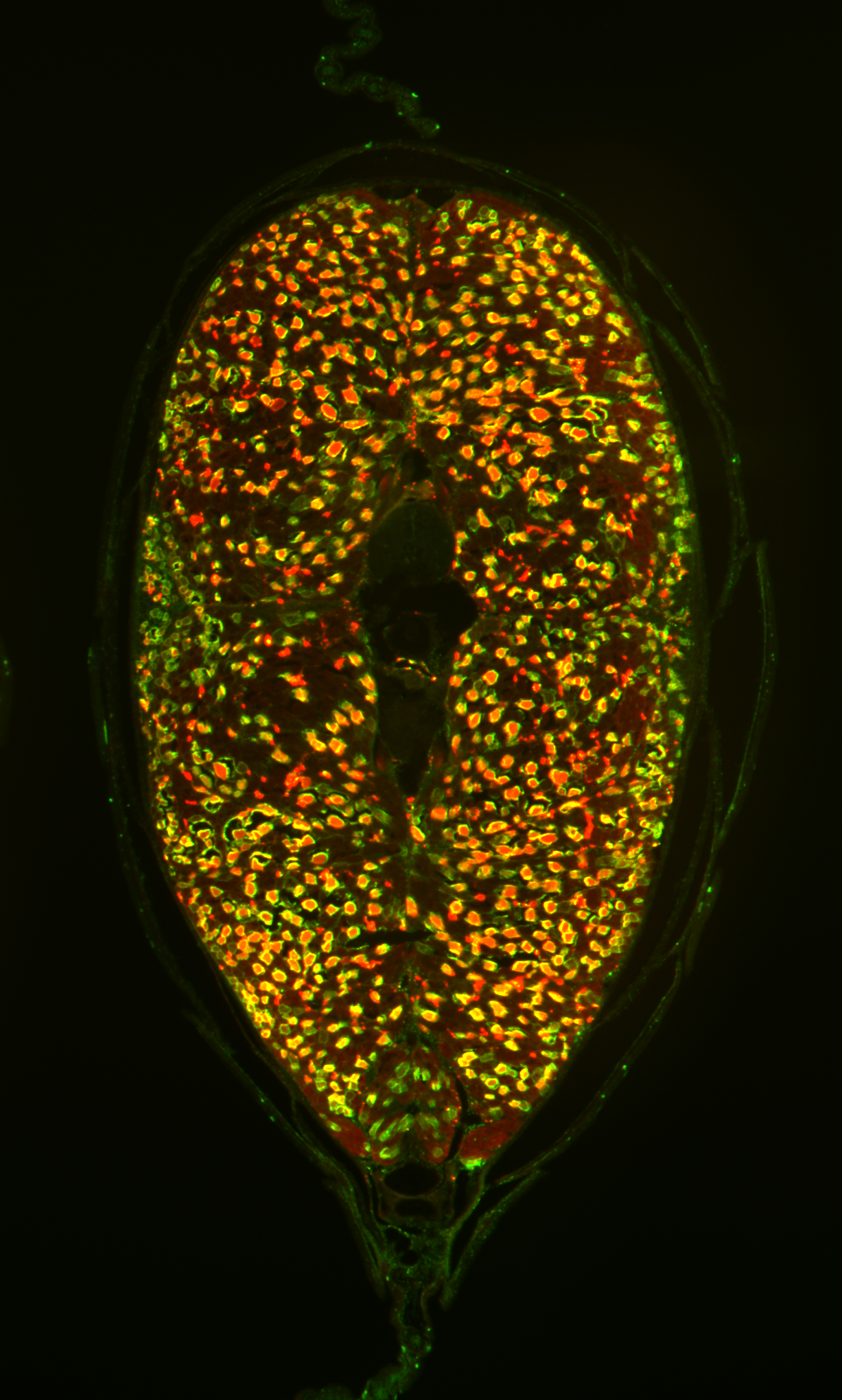

Supplement: Supplementary file 14 — Source data Fig. 5 [file 44318_2024_136_MOESM14_ESM.zip › Figure 5Q/Cross-section-DT-43 dpf.tif]

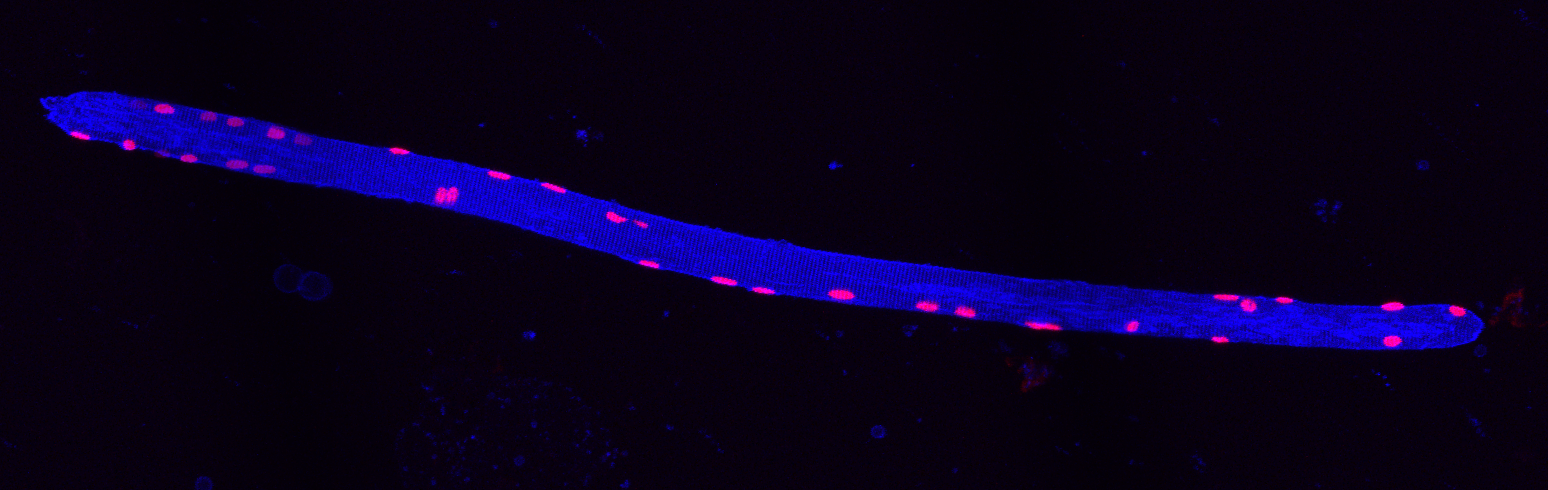

Supplement: Supplementary file 15 — Source data Fig. 6 [file 44318_2024_136_MOESM15_ESM.zip › Figure 6B/palmuscle-Dual-adult myofiber-1.7-year.tif]

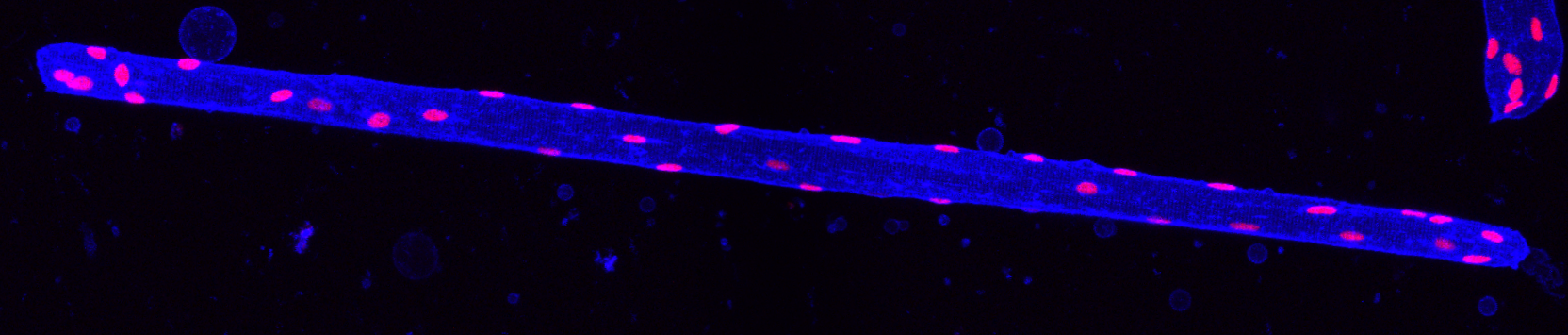

Supplement: Supplementary file 15 — Source data Fig. 6 [file 44318_2024_136_MOESM15_ESM.zip › Figure 6B/palmuscle-Dual-juvenile myofiber-70 dpf.tif]

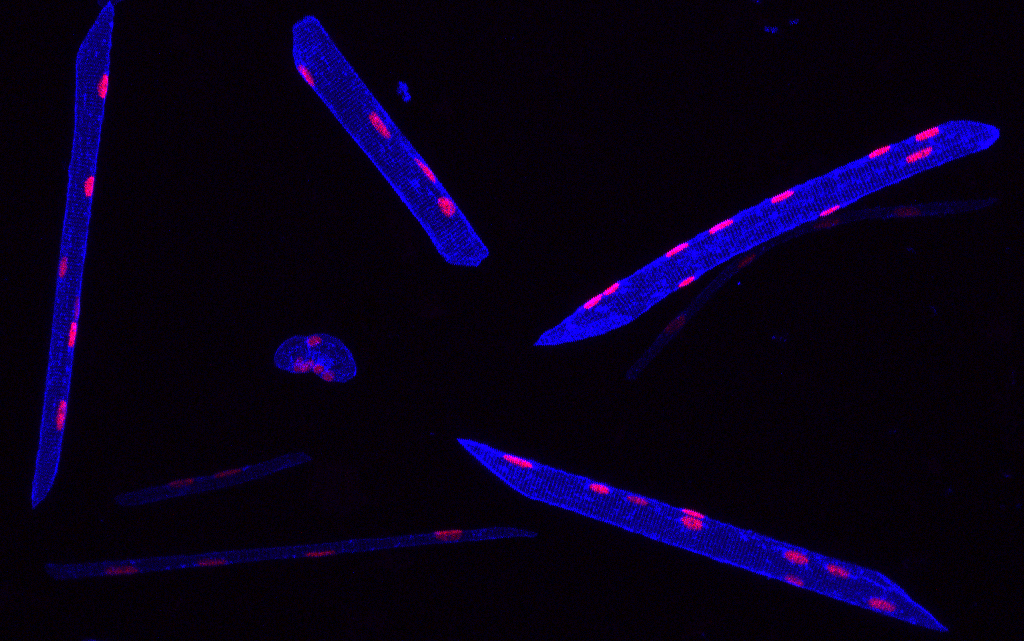

Supplement: Supplementary file 15 — Source data Fig. 6 [file 44318_2024_136_MOESM15_ESM.zip › Figure 6B/palmuscle-Dual-larvae myofiber-14 dpf.tif]

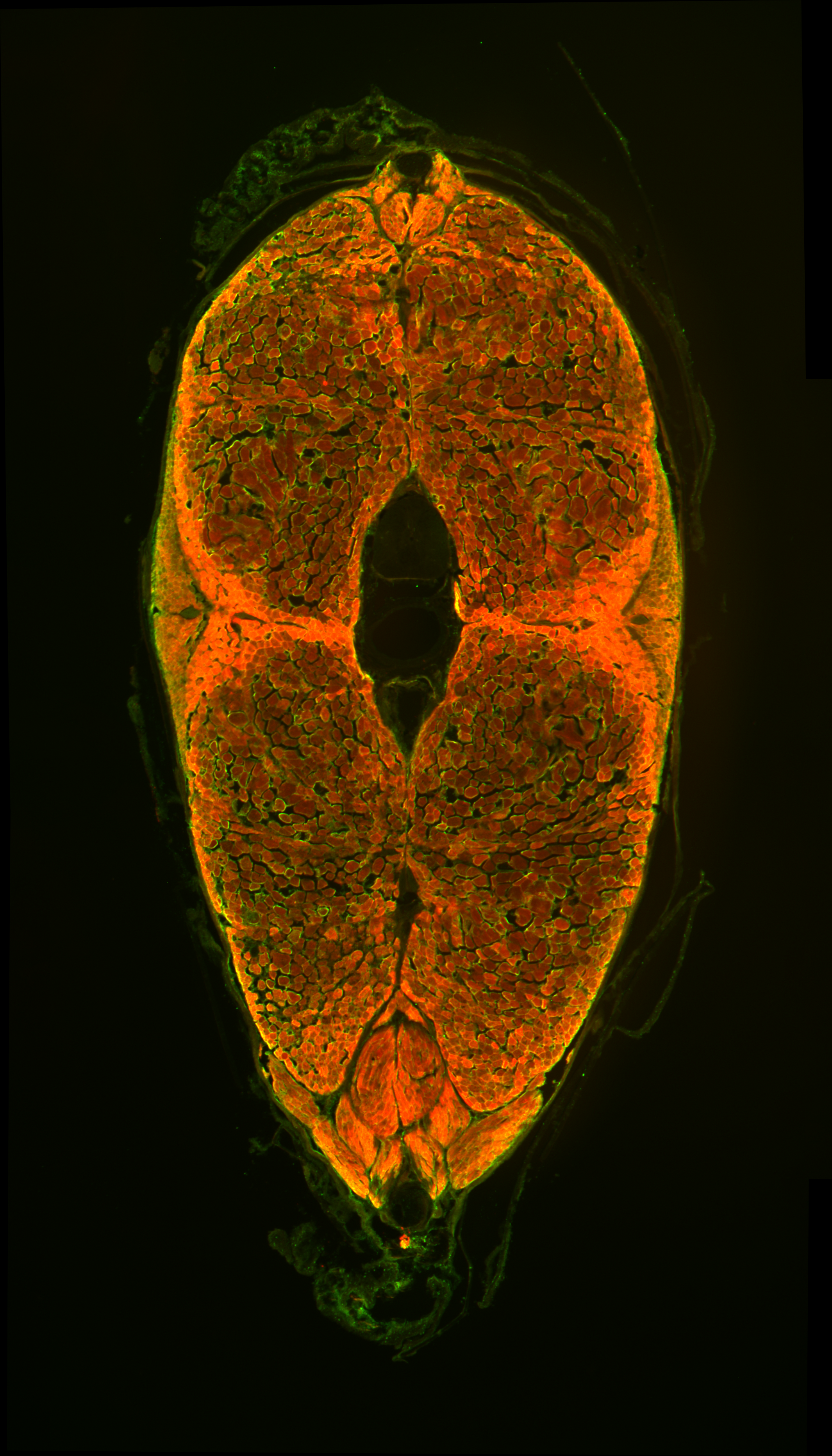

Supplement: Supplementary file 15 — Source data Fig. 6 [file 44318_2024_136_MOESM15_ESM.zip › Figure 6J/Trunk section-DT-10 mpf.tiff]

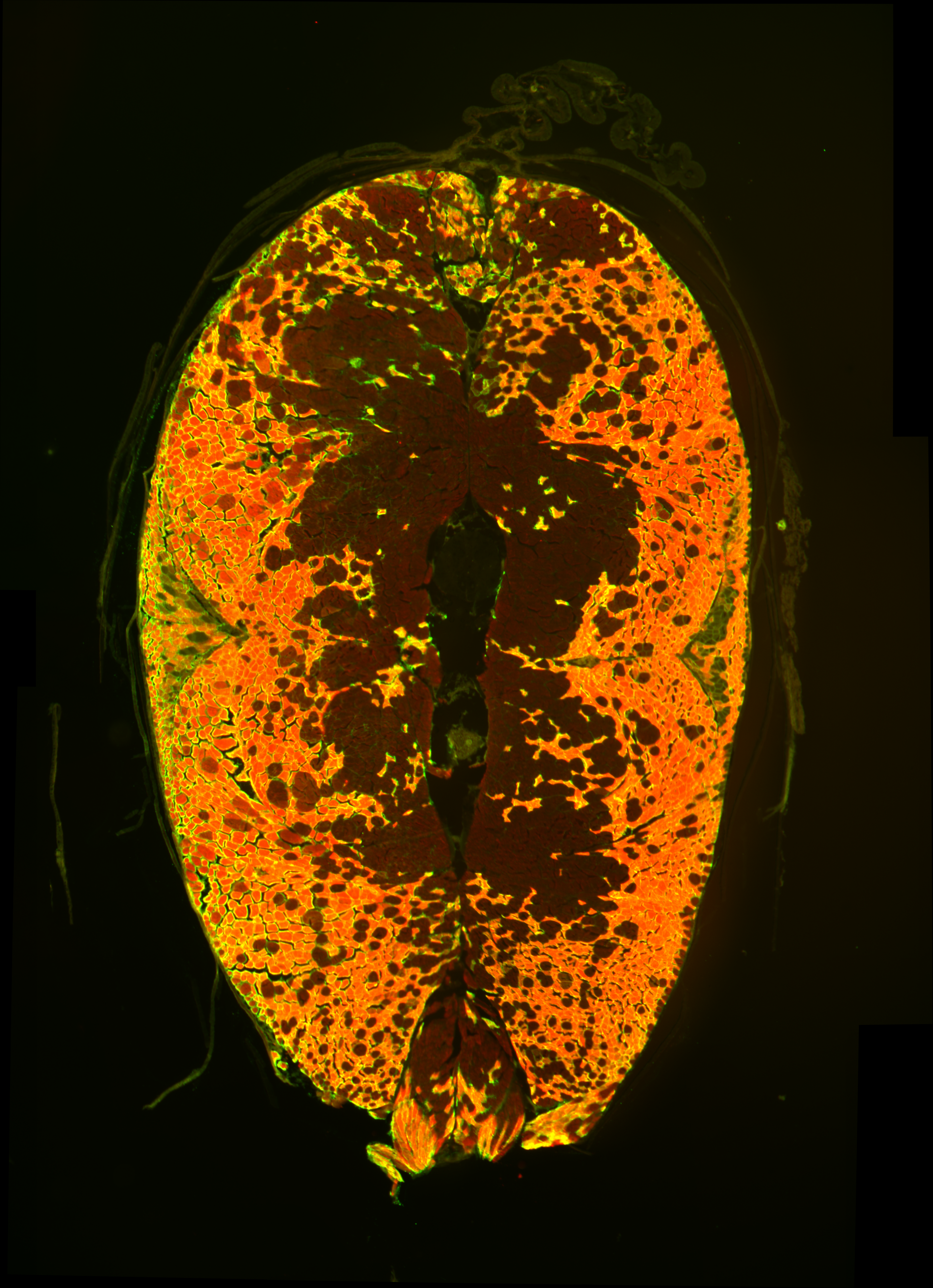

Supplement: Supplementary file 15 — Source data Fig. 6 [file 44318_2024_136_MOESM15_ESM.zip › Figure 6J/Trunk section-DT-16 mpf.tiff]

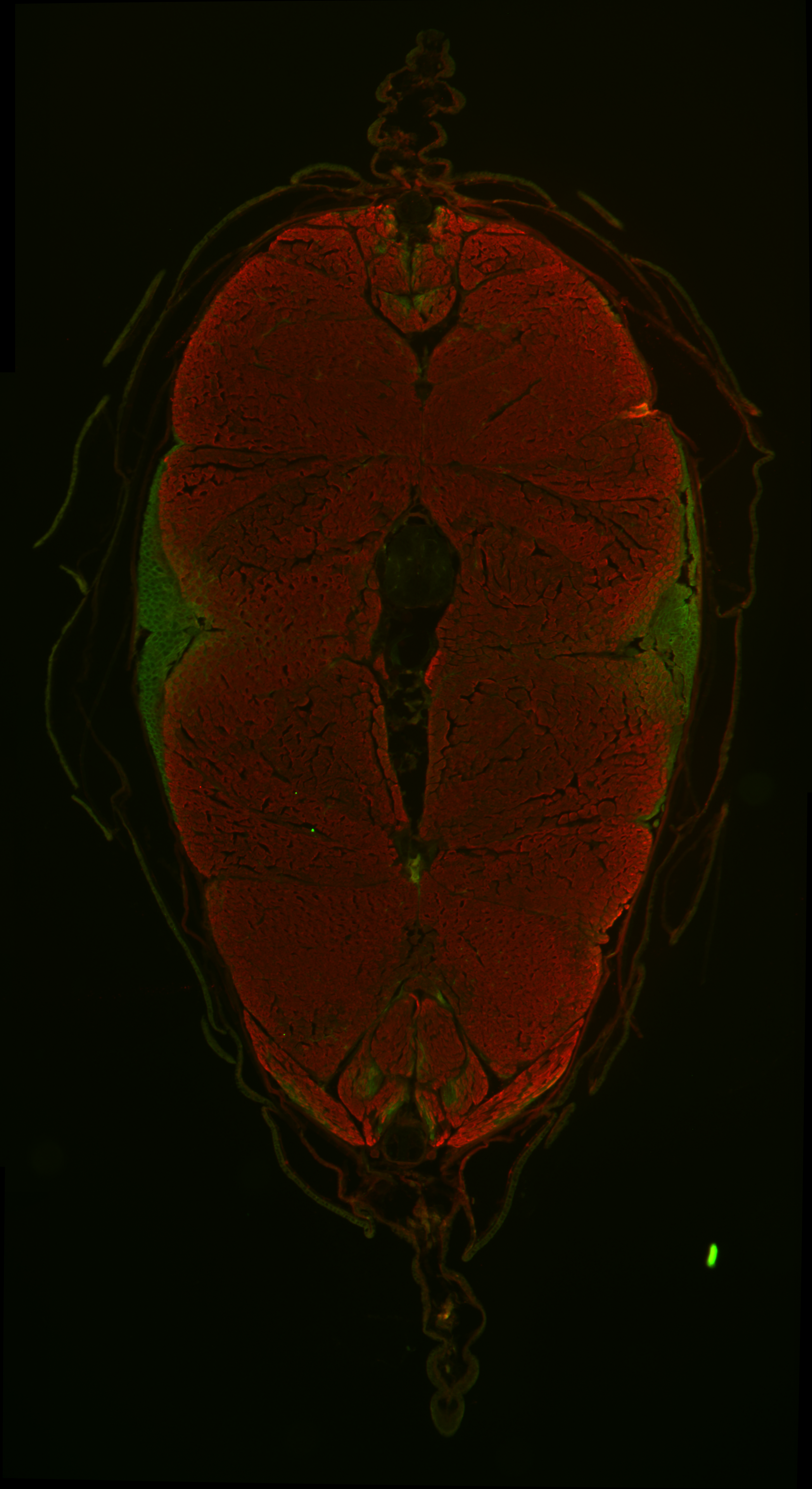

Supplement: Supplementary file 15 — Source data Fig. 6 [file 44318_2024_136_MOESM15_ESM.zip › Figure 6J/Trunk section-no treatment-10 mpf.tiff]

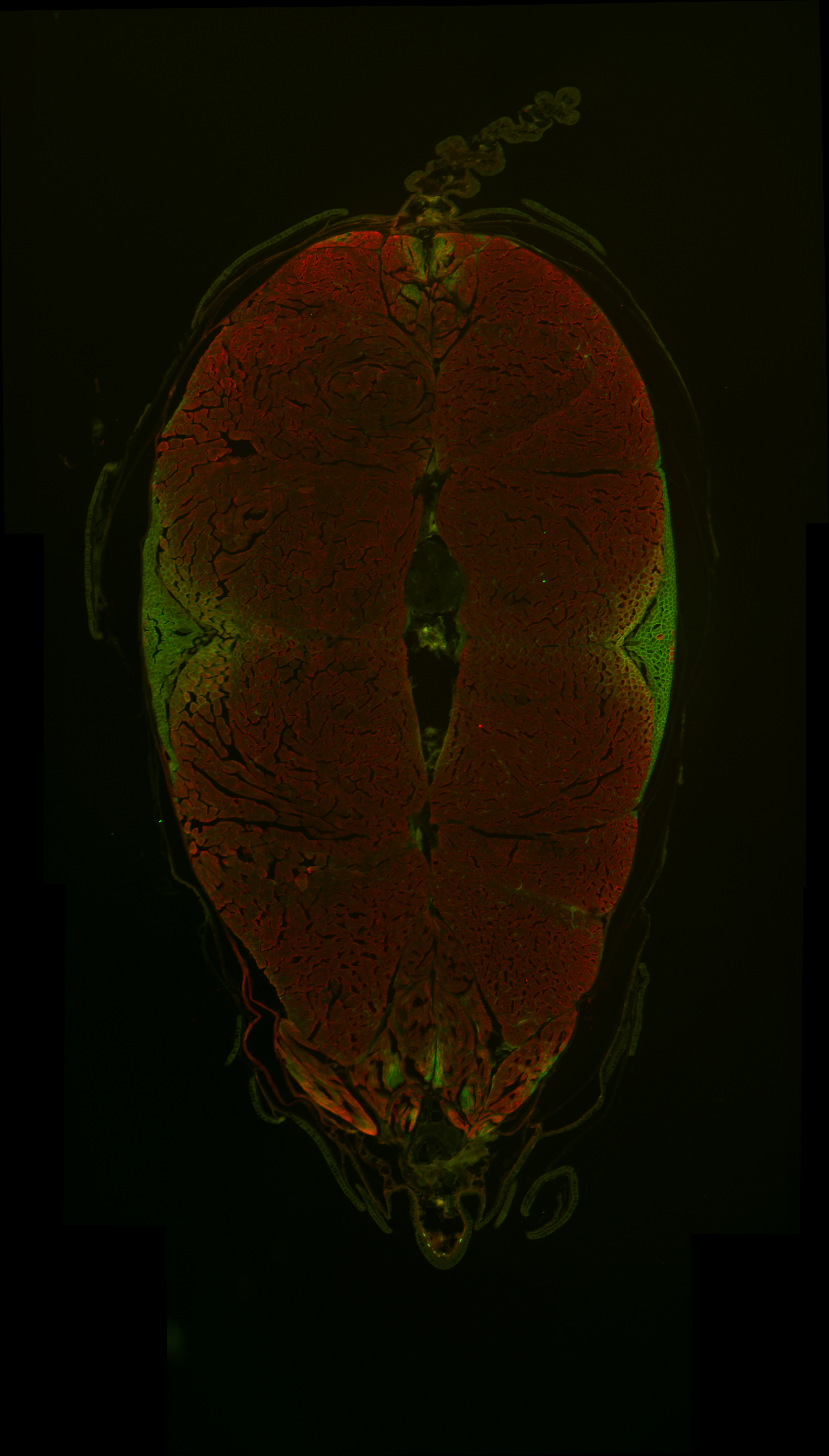

Supplement: Supplementary file 15 — Source data Fig. 6 [file 44318_2024_136_MOESM15_ESM.zip › Figure 6J/Trunk section-no treatment-16 mpf.tiff]

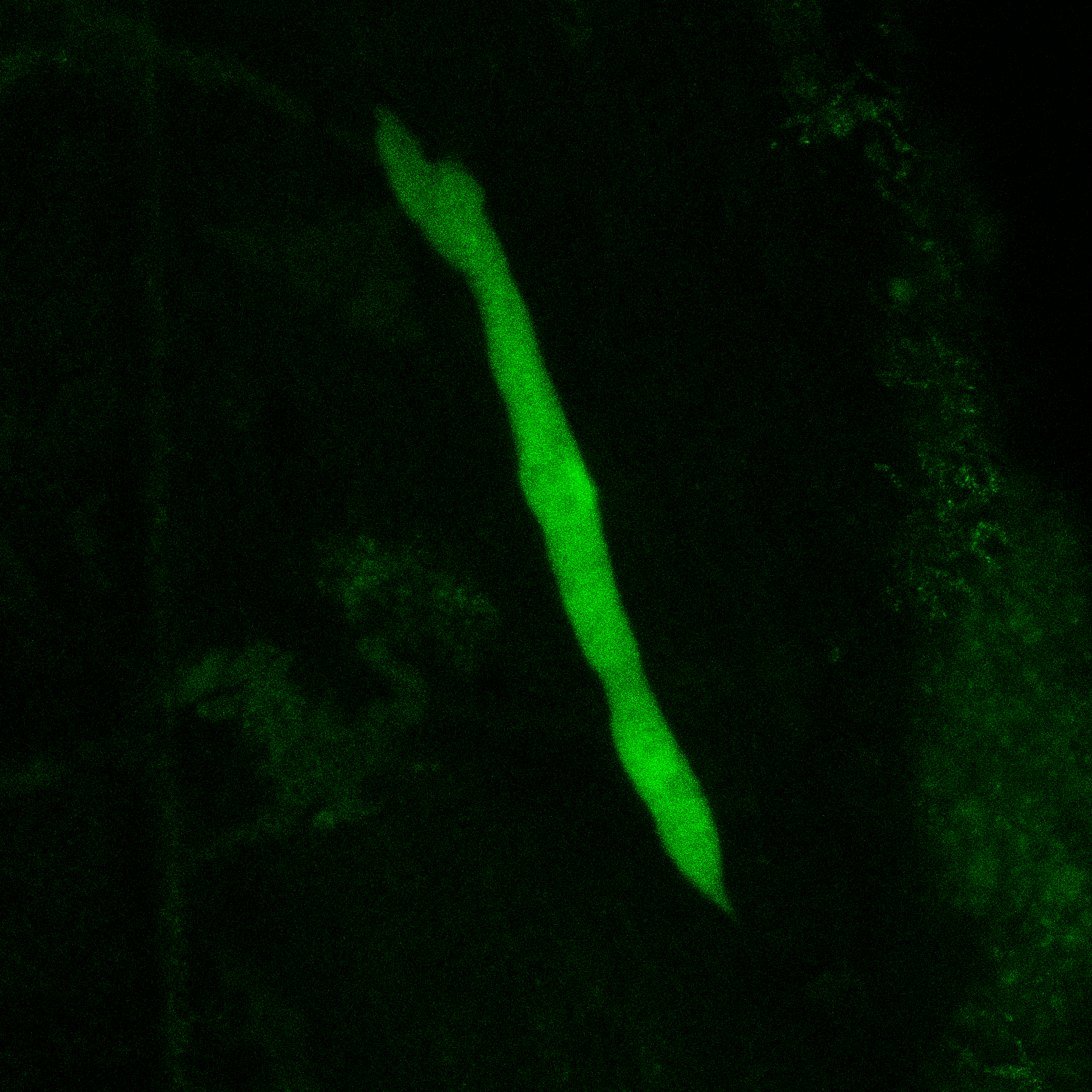

Supplement: Supplementary file 16 — Source data Fig. 7 [file 44318_2024_136_MOESM16_ESM.zip › Figure 7B/GC3AI-cycloheximide-green channel.tif]

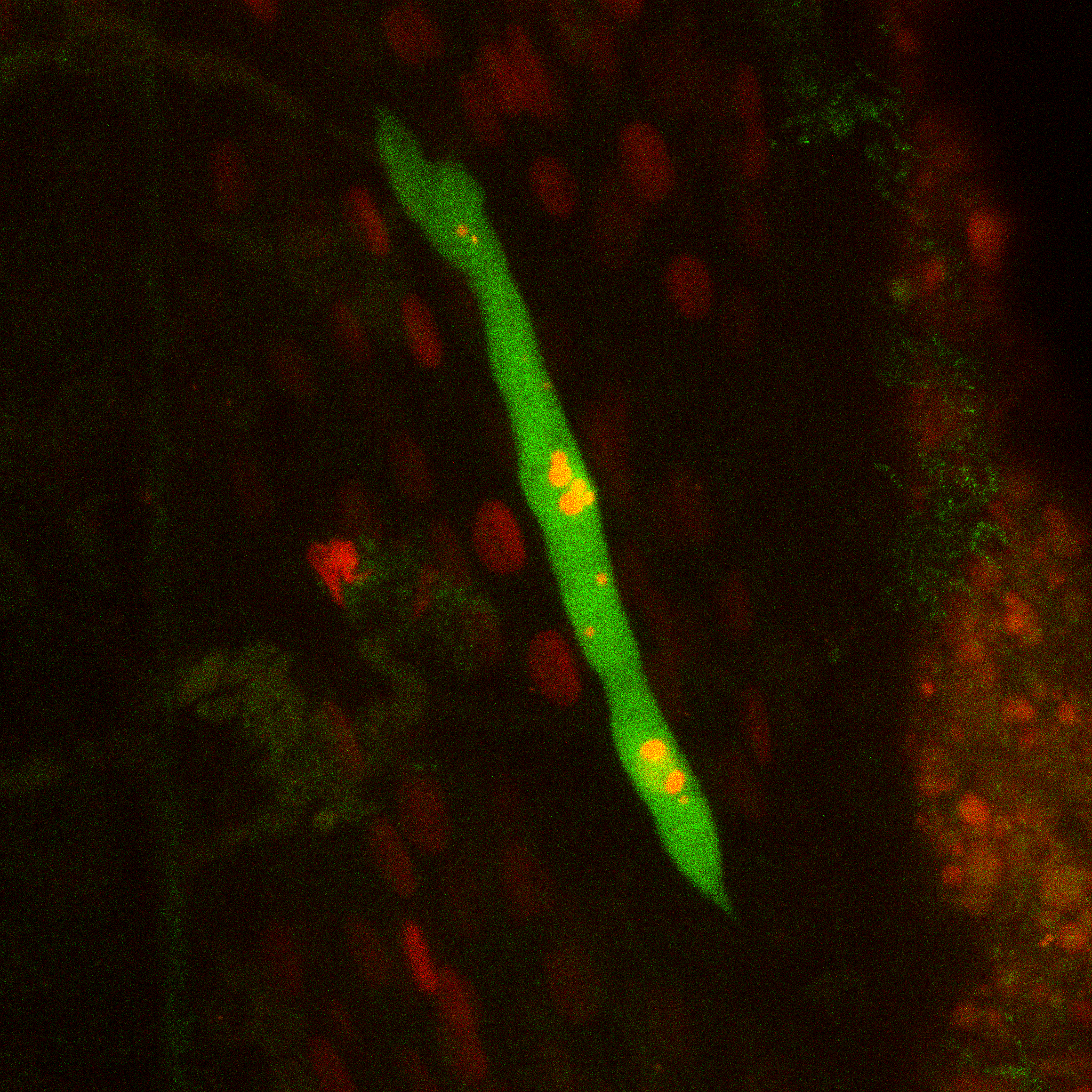

Supplement: Supplementary file 16 — Source data Fig. 7 [file 44318_2024_136_MOESM16_ESM.zip › Figure 7B/GC3AI-cycloheximide-merged.tif]

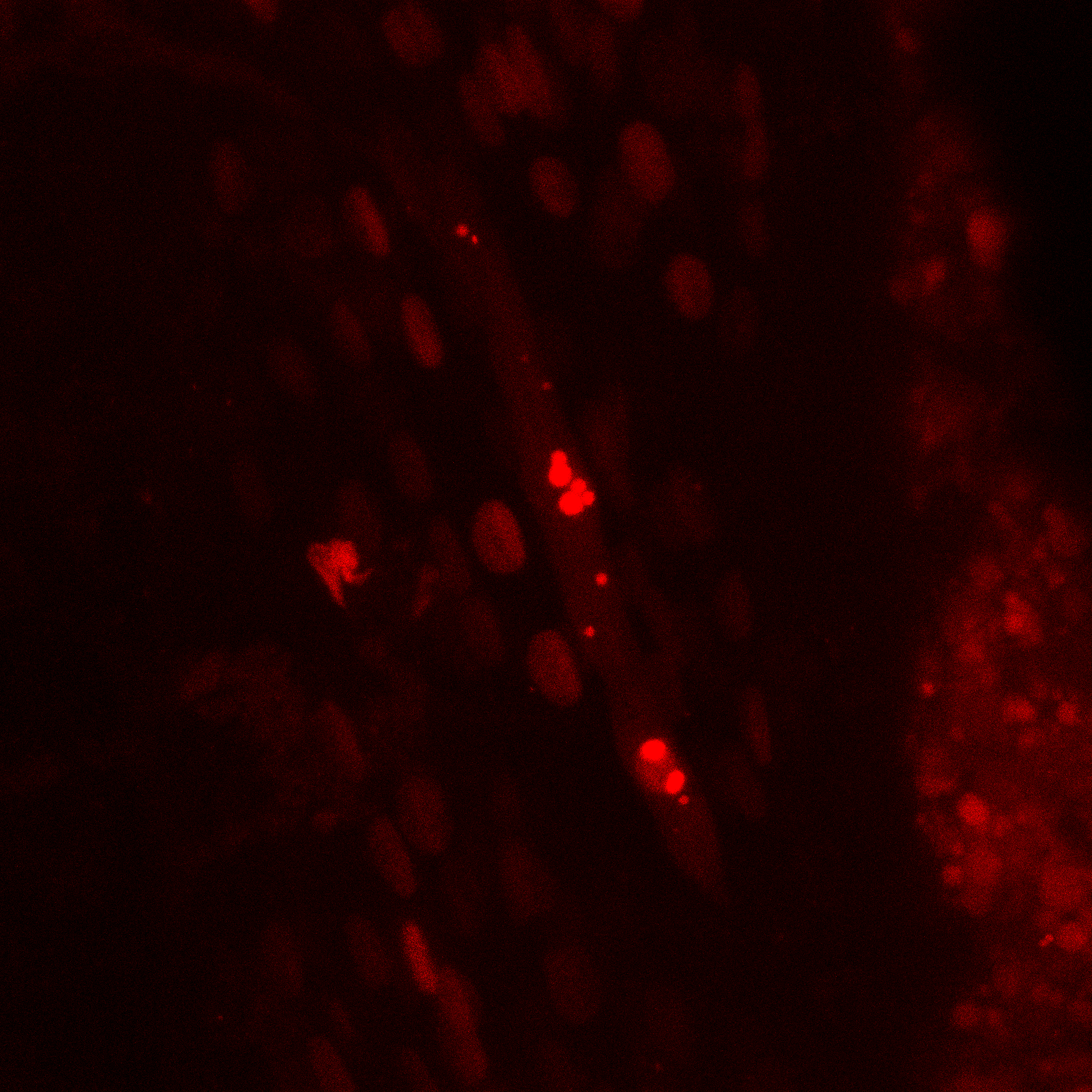

Supplement: Supplementary file 16 — Source data Fig. 7 [file 44318_2024_136_MOESM16_ESM.zip › Figure 7B/GC3AI-cycloheximide-red channel.tif]

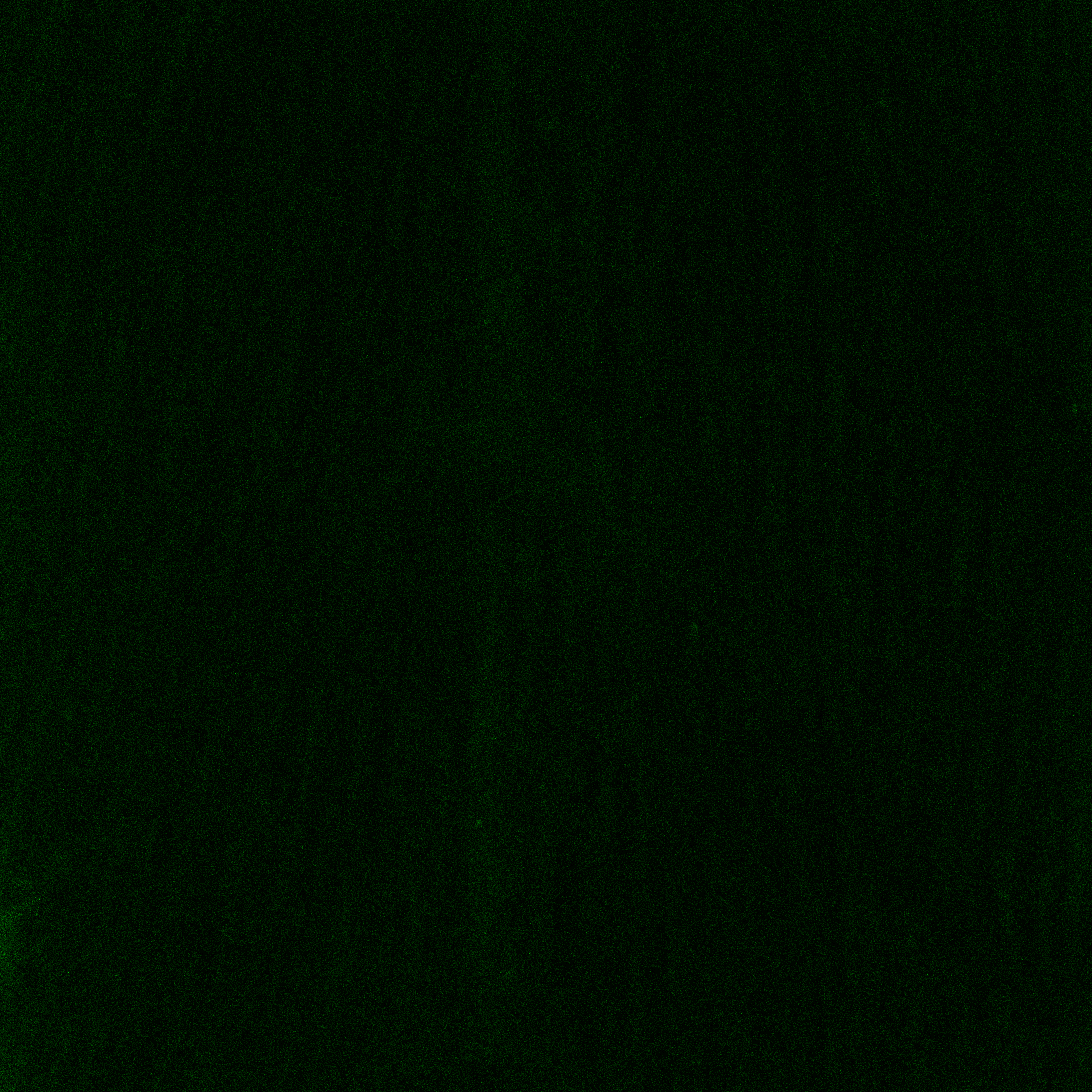

Supplement: Supplementary file 16 — Source data Fig. 7 [file 44318_2024_136_MOESM16_ESM.zip › Figure 7B/GC3AI-no treatment-green channel.tif]

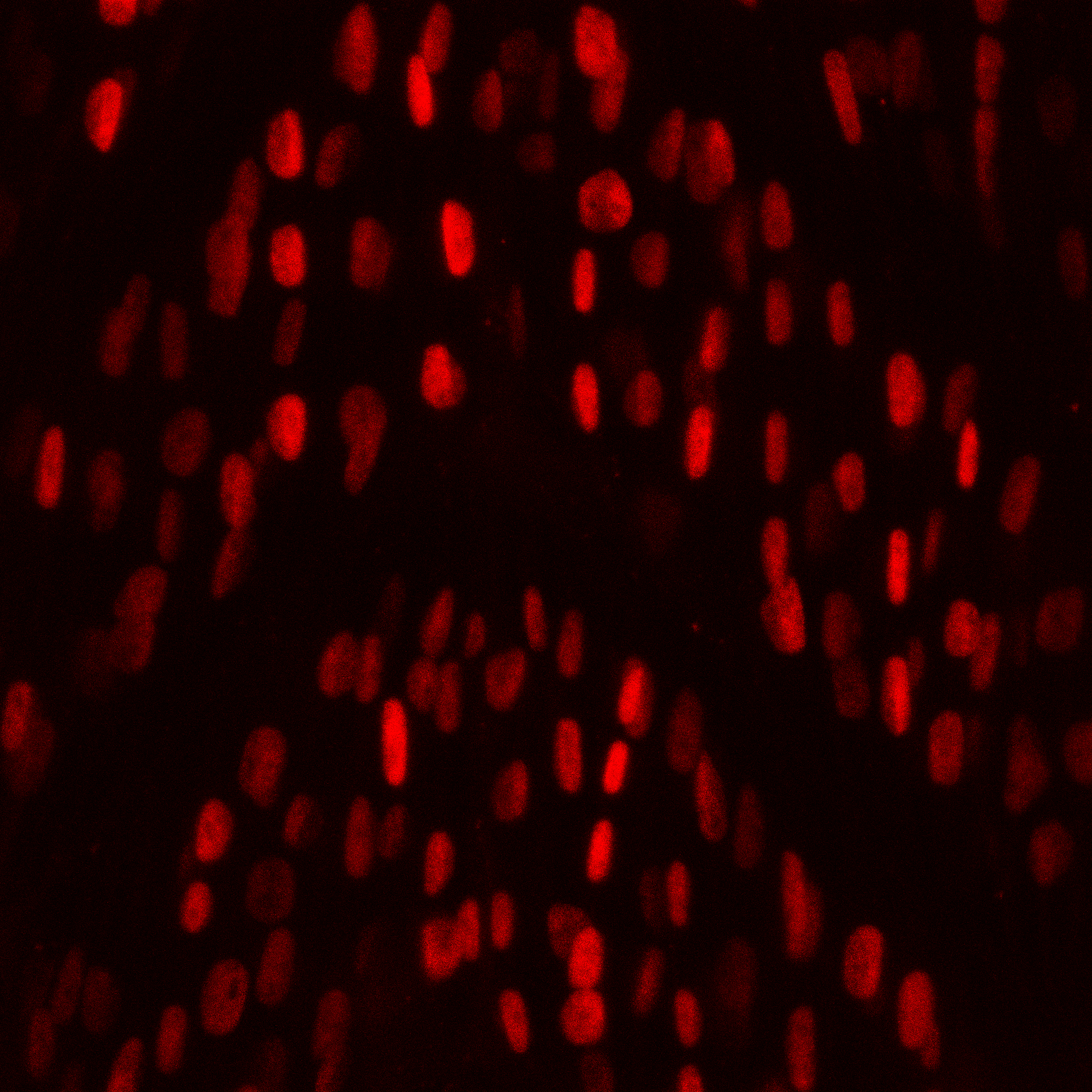

Supplement: Supplementary file 16 — Source data Fig. 7 [file 44318_2024_136_MOESM16_ESM.zip › Figure 7B/GC3AI-no treatment-merged.tif]

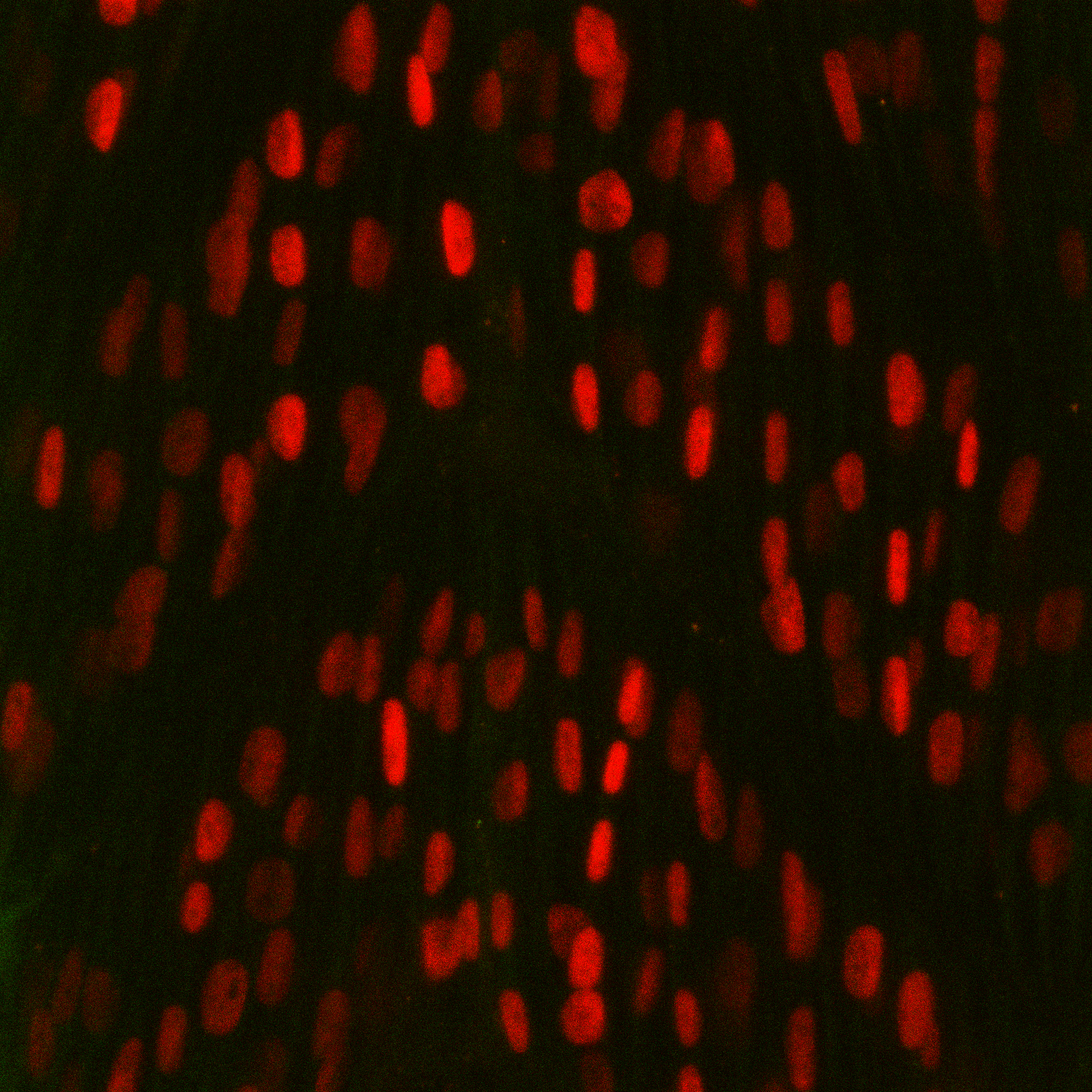

Supplement: Supplementary file 16 — Source data Fig. 7 [file 44318_2024_136_MOESM16_ESM.zip › Figure 7B/GC3AI-no treatment-red channel.tif]

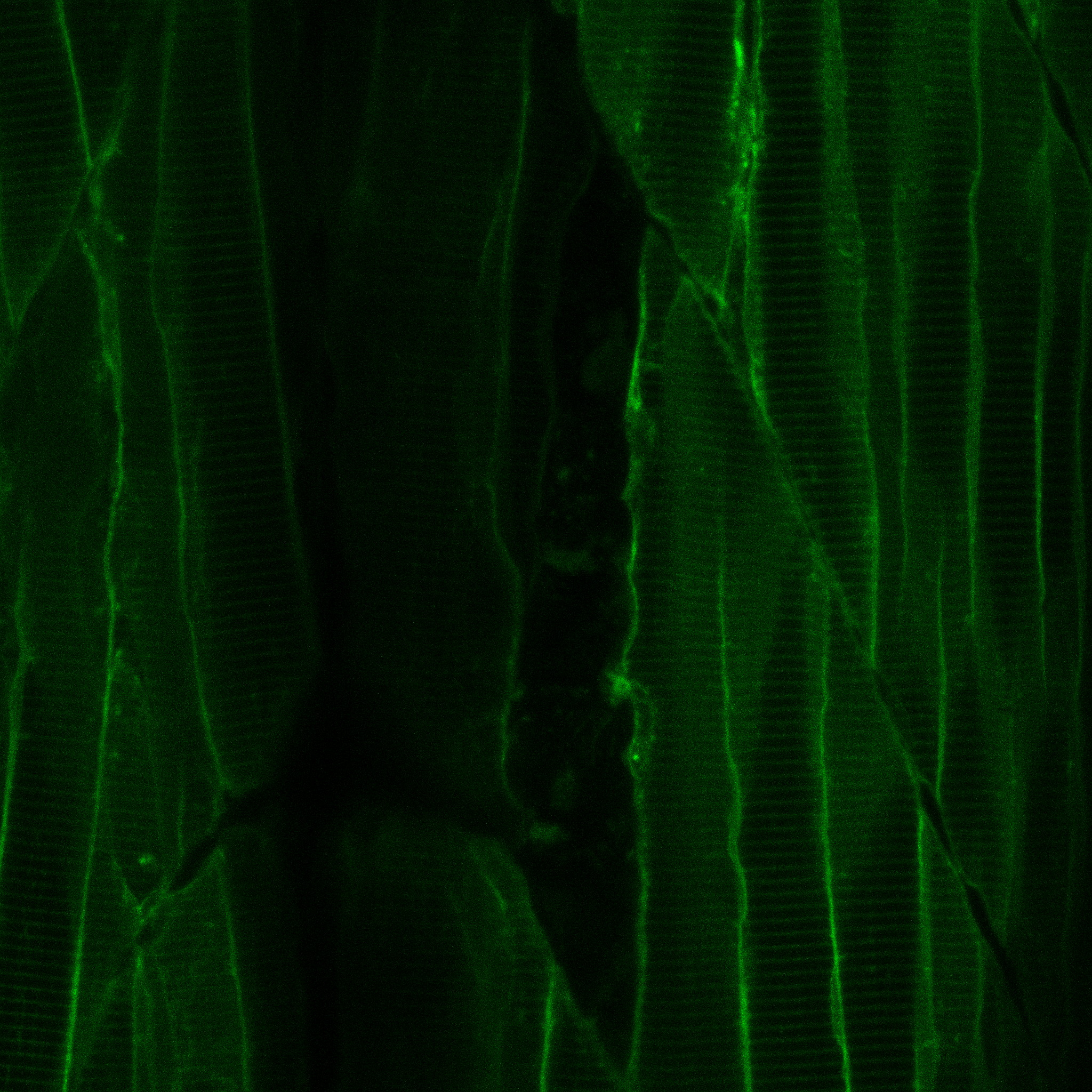

Supplement: Supplementary file 16 — Source data Fig. 7 [file 44318_2024_136_MOESM16_ESM.zip › Figure 7C/GC3AI-deformed myofiber-green channel.tif]

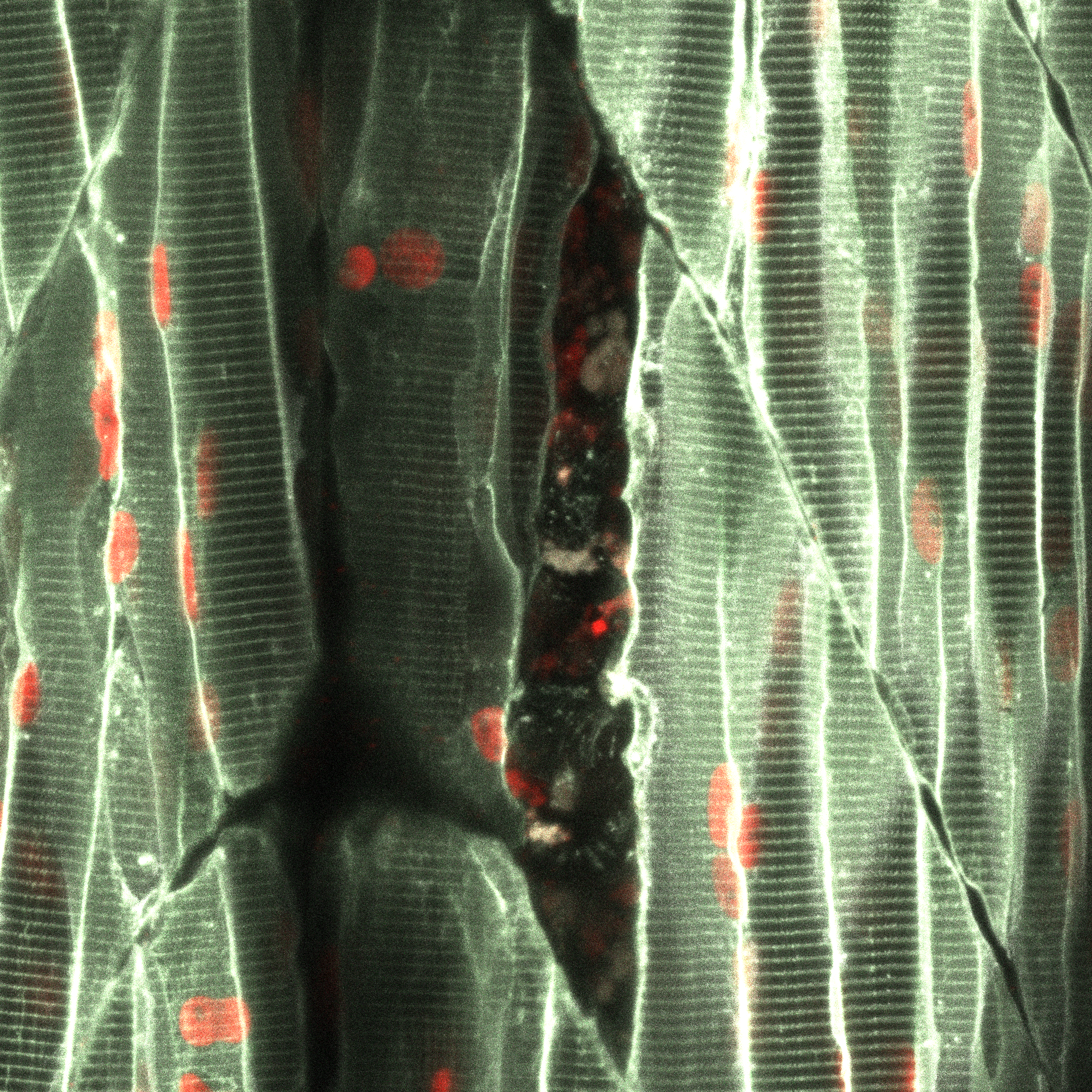

Supplement: Supplementary file 16 — Source data Fig. 7 [file 44318_2024_136_MOESM16_ESM.zip › Figure 7C/GC3AI-deformed myofiber-merged.tif]

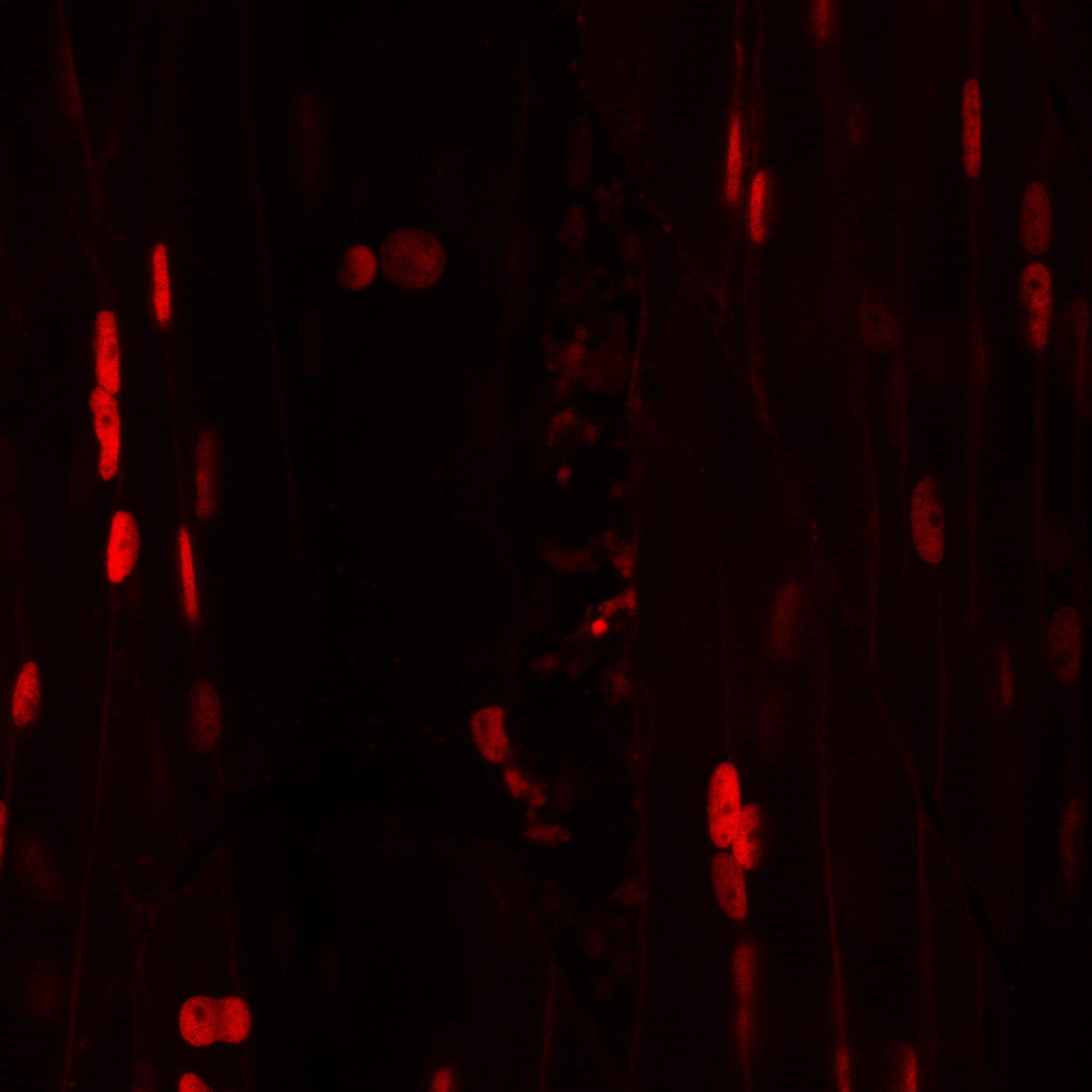

Supplement: Supplementary file 16 — Source data Fig. 7 [file 44318_2024_136_MOESM16_ESM.zip › Figure 7C/GC3AI-deformed myofiber-red channel.tif]

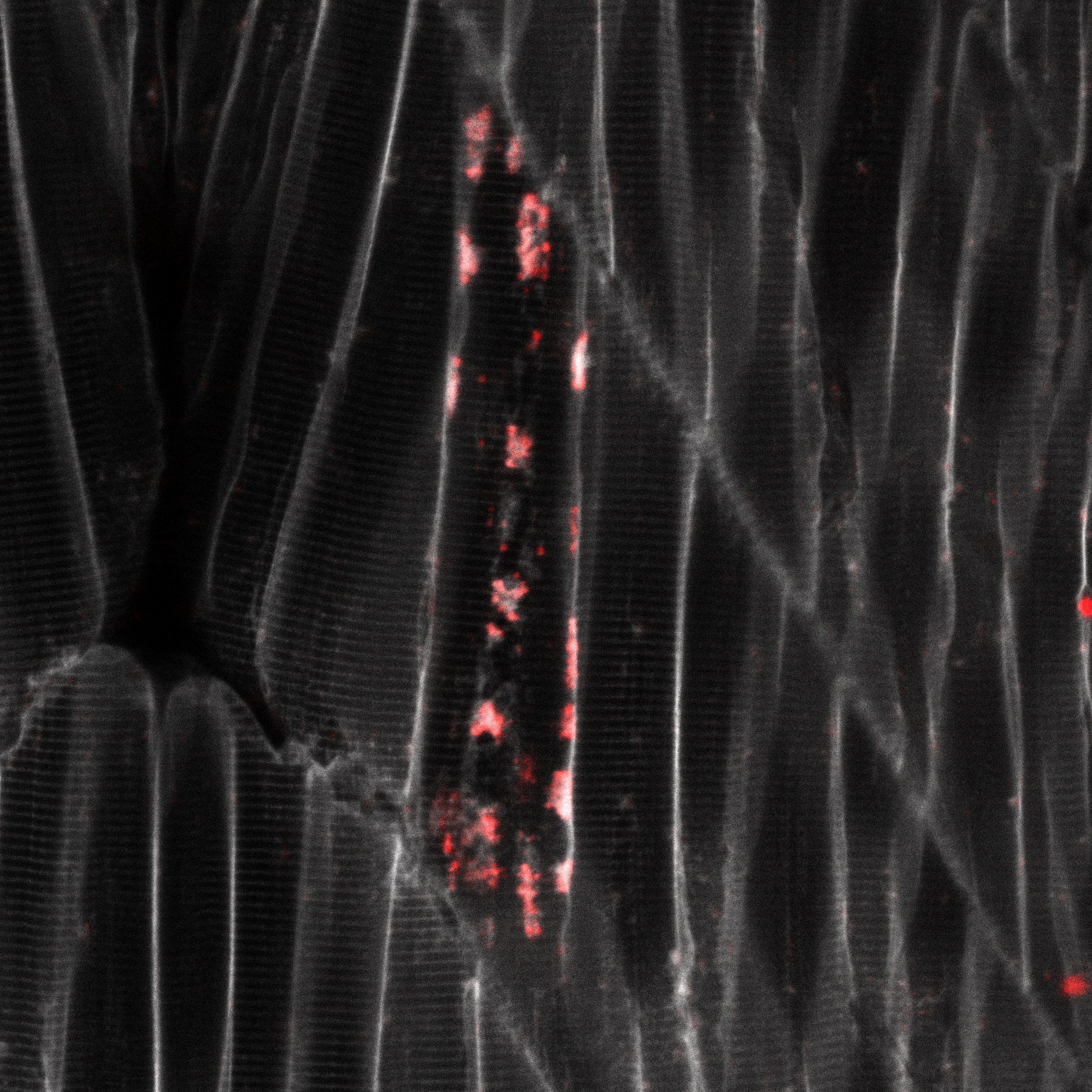

Supplement: Supplementary file 16 — Source data Fig. 7 [file 44318_2024_136_MOESM16_ESM.zip › Figure 7F/LysoTracker-deformed myofiber-merged.tif]

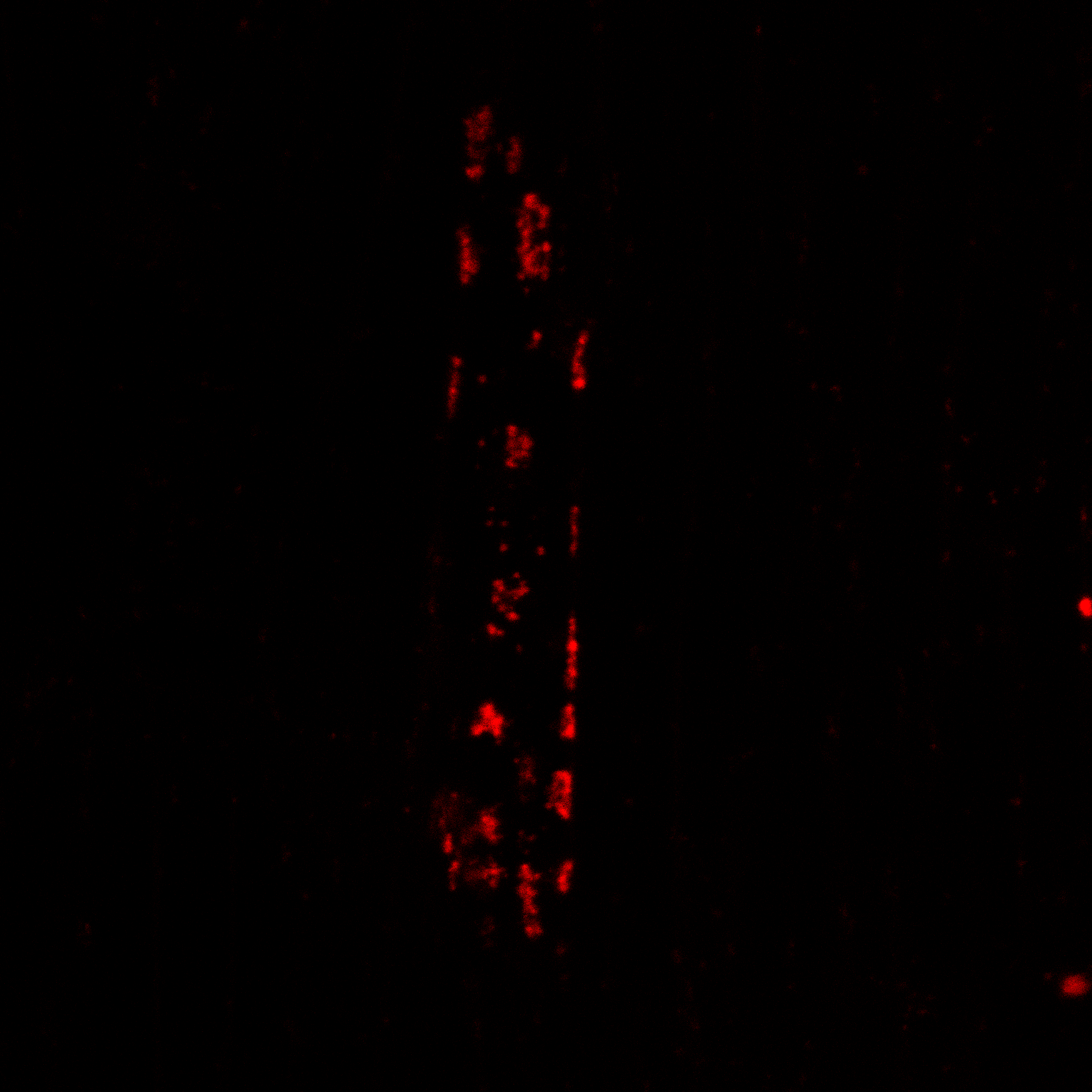

Supplement: Supplementary file 16 — Source data Fig. 7 [file 44318_2024_136_MOESM16_ESM.zip › Figure 7F/LysoTracker-deformed myofiber-red channel.tif]

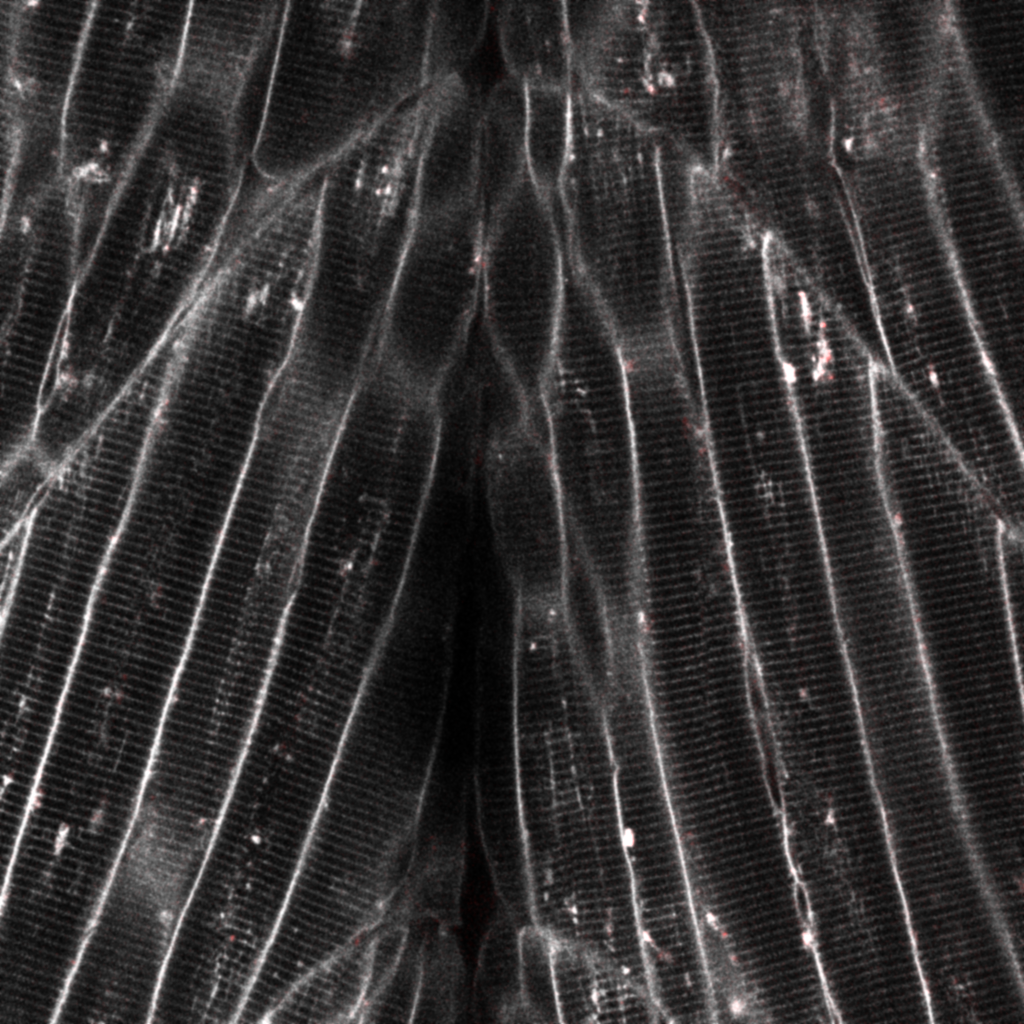

Supplement: Supplementary file 16 — Source data Fig. 7 [file 44318_2024_136_MOESM16_ESM.zip › Figure 7F/LysoTracker-normal myofiber-merged.tif]

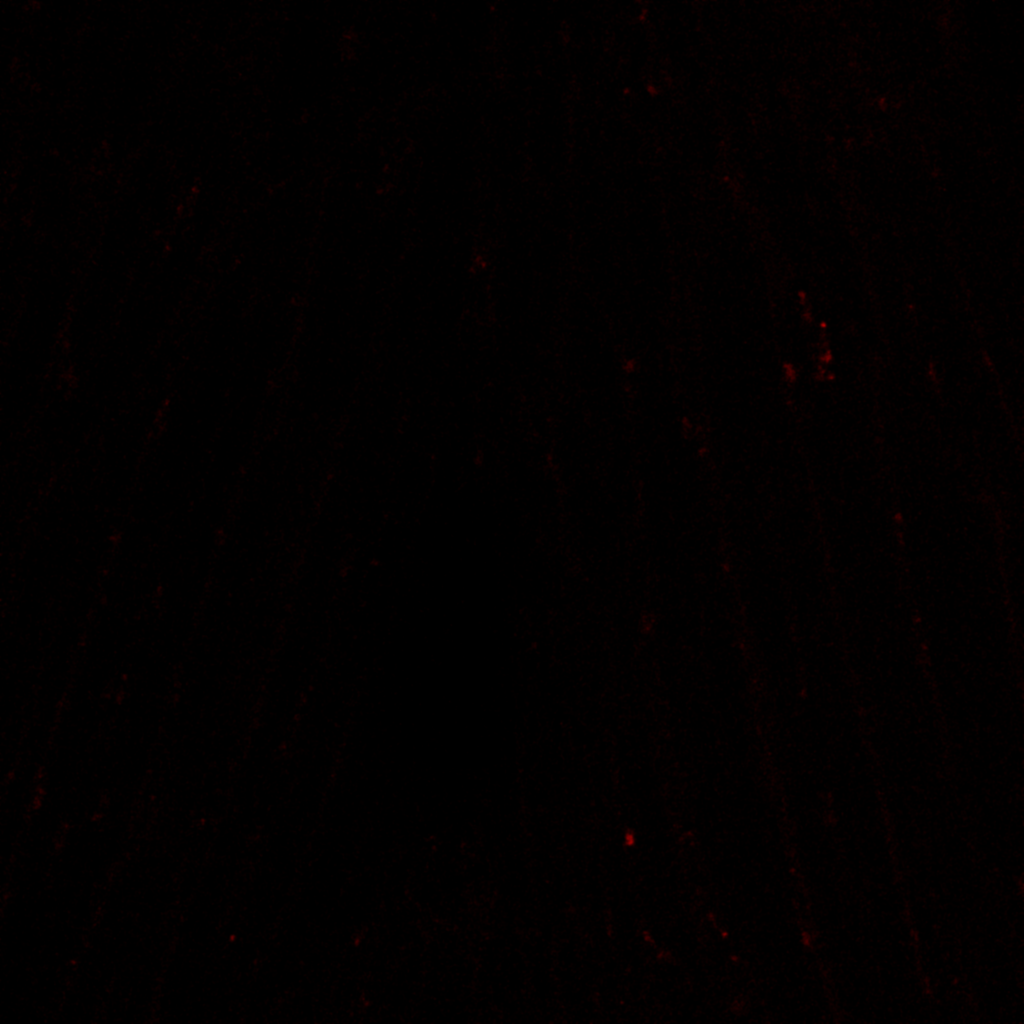

Supplement: Supplementary file 16 — Source data Fig. 7 [file 44318_2024_136_MOESM16_ESM.zip › Figure 7F/LysoTracker-normal myofiber-red channel.tif]

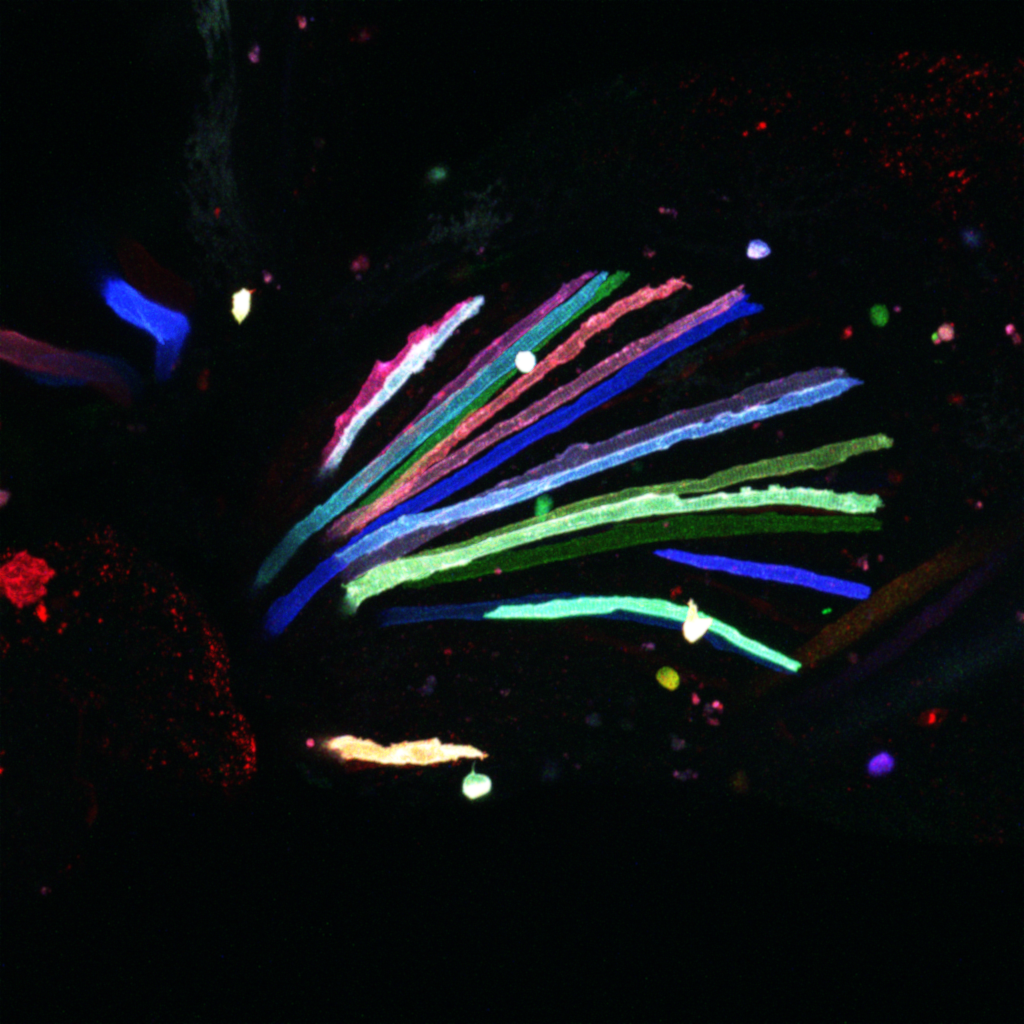

Supplement: Supplementary file 16 — Source data Fig. 7 [file 44318_2024_136_MOESM16_ESM.zip › Figure 7I/Pectoral fin-CQ-14 dpf.tif]

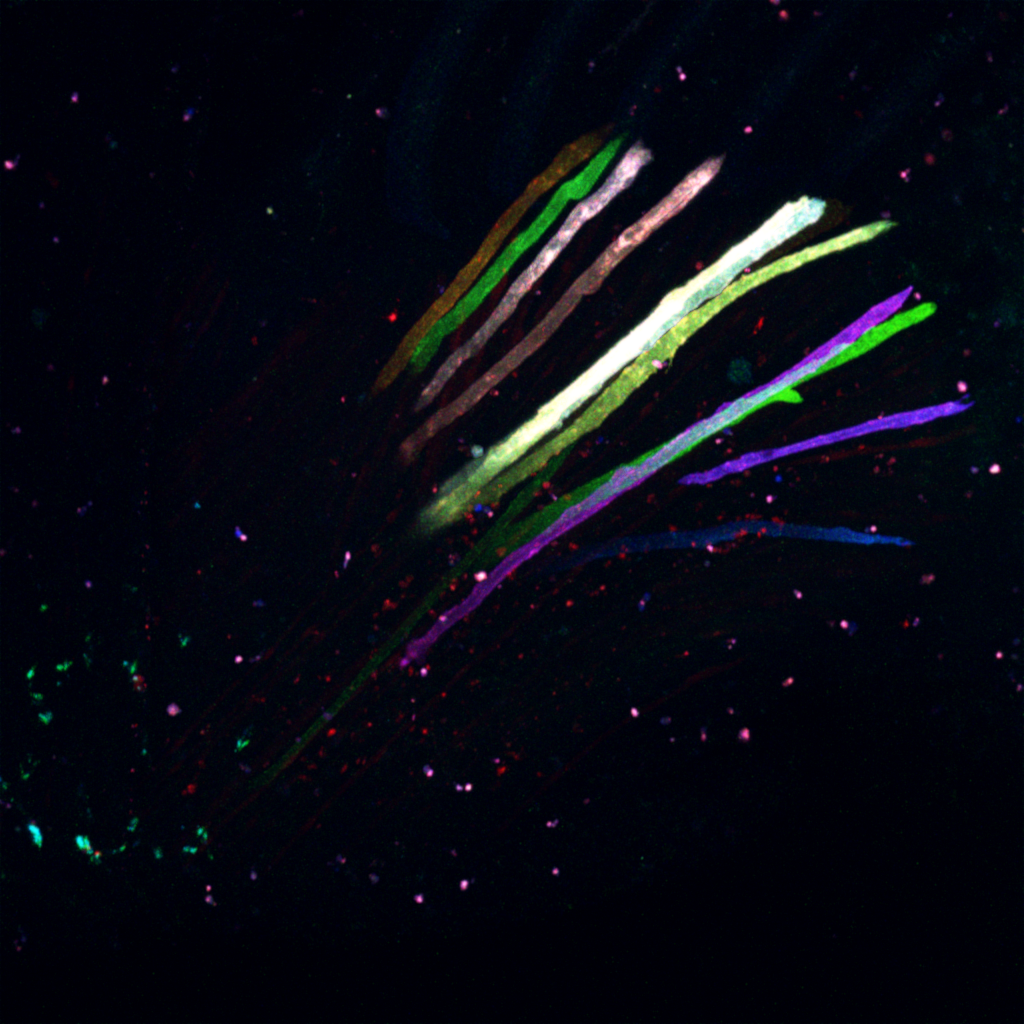

Supplement: Supplementary file 16 — Source data Fig. 7 [file 44318_2024_136_MOESM16_ESM.zip › Figure 7I/Pectoral fin-CQ-28 dpf.tif]

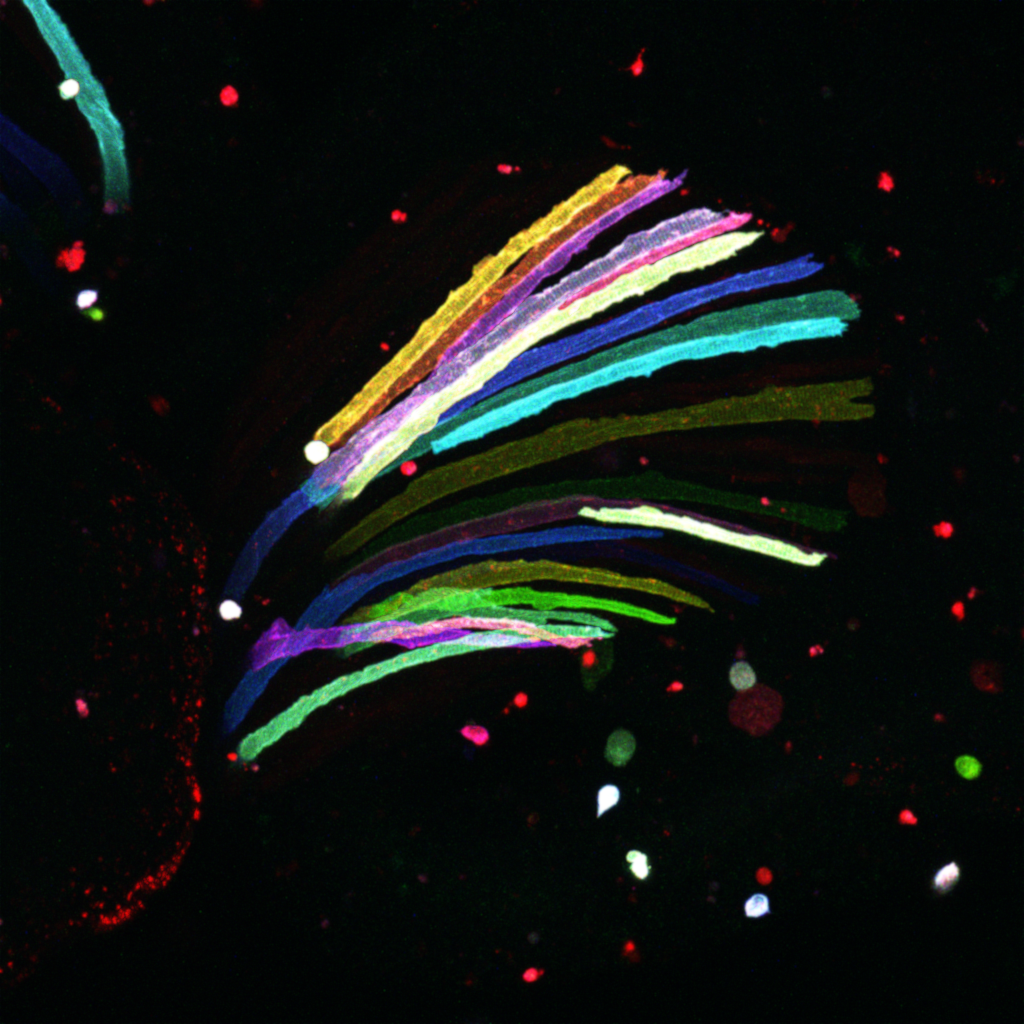

Supplement: Supplementary file 16 — Source data Fig. 7 [file 44318_2024_136_MOESM16_ESM.zip › Figure 7I/Pectoral fin-ctrl-14 dpf.tif]

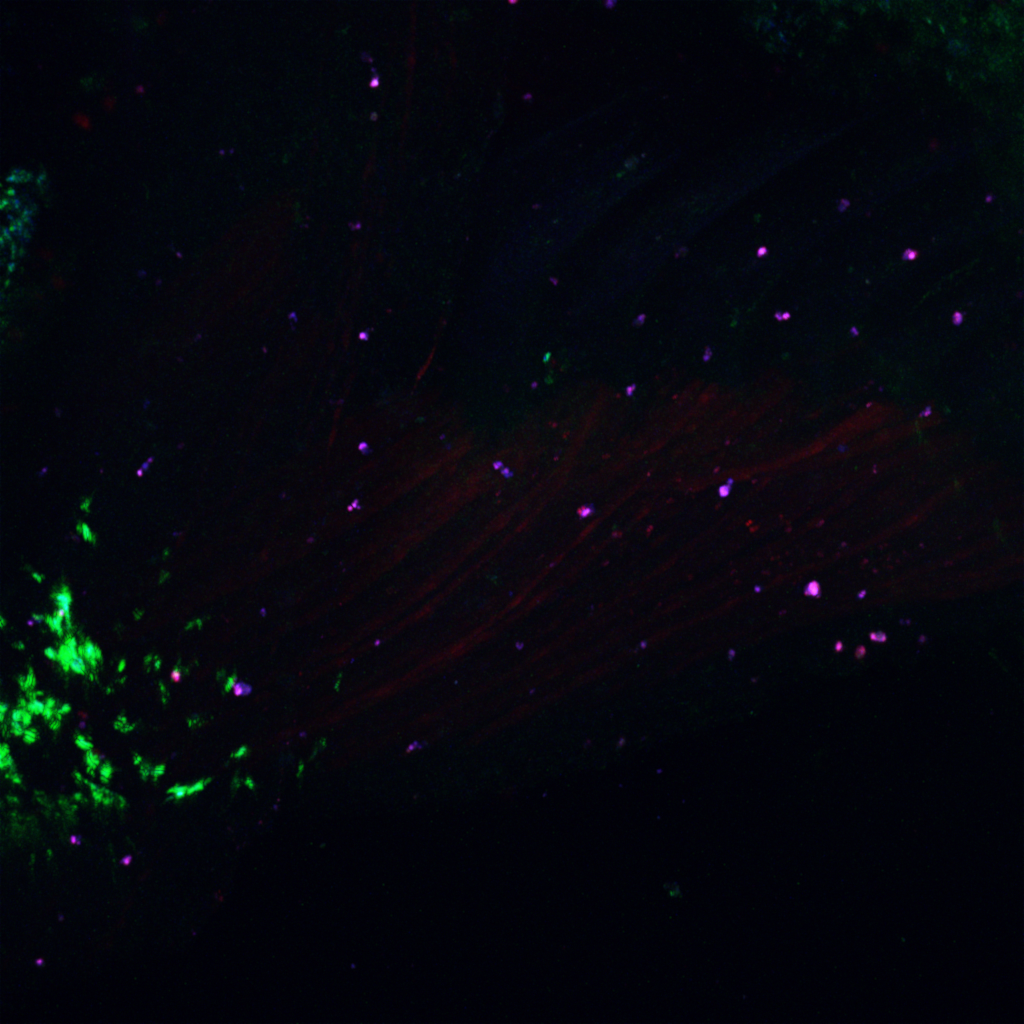

Supplement: Supplementary file 16 — Source data Fig. 7 [file 44318_2024_136_MOESM16_ESM.zip › Figure 7I/Pectoral fin-ctrl-28 dpf.tif]

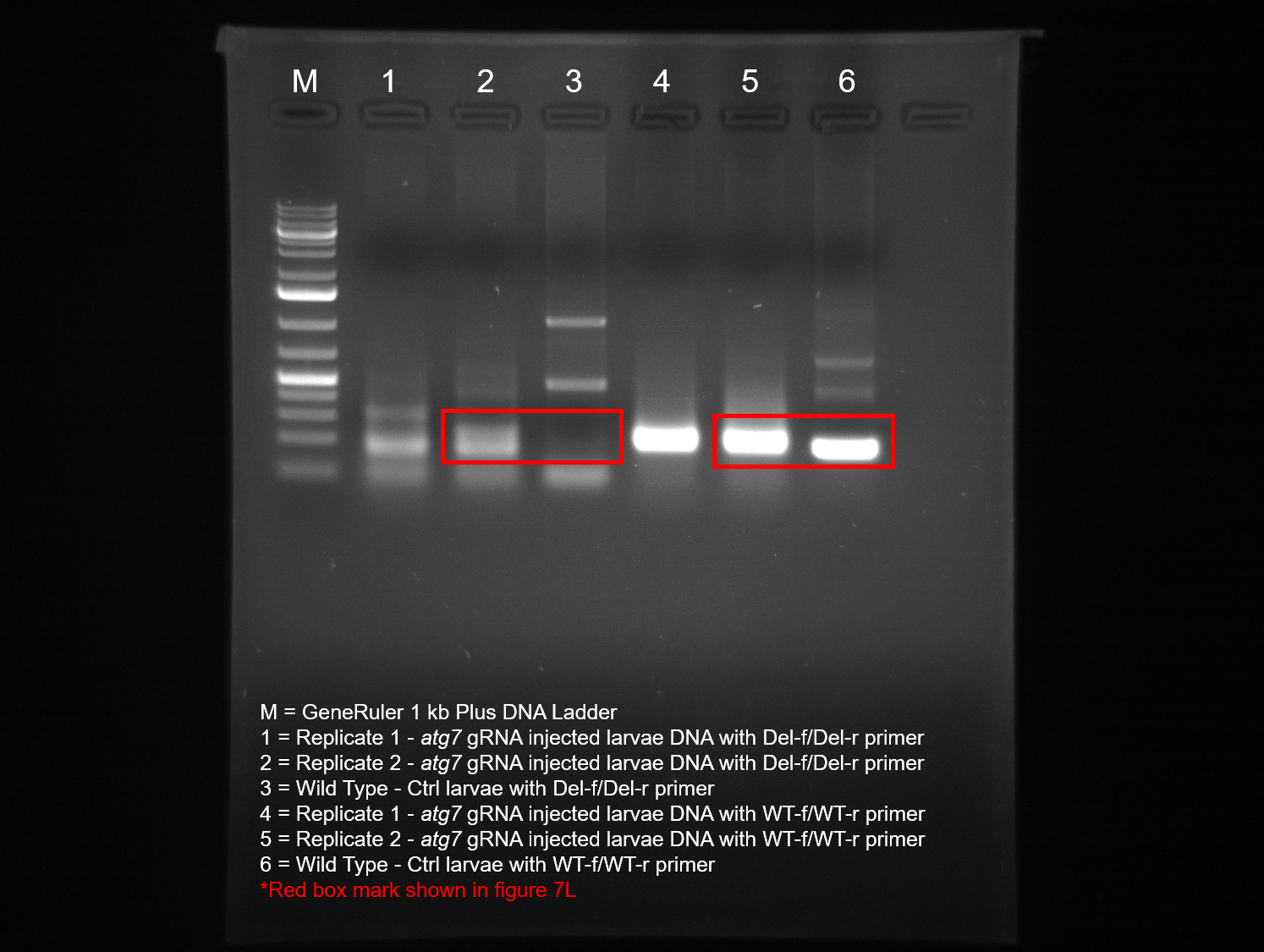

Supplement: Supplementary file 16 — Source data Fig. 7 [file 44318_2024_136_MOESM16_ESM.zip › Figure 7L/Gel image.tif]

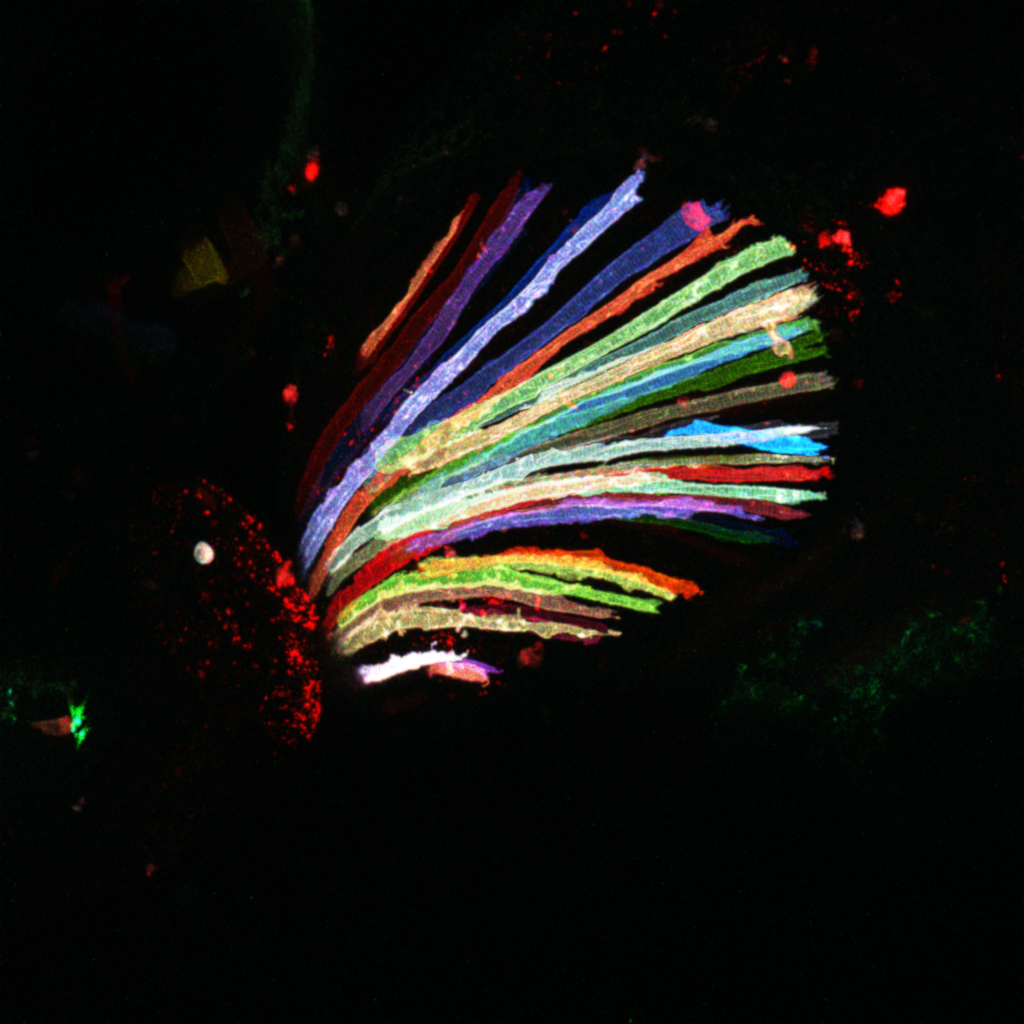

Supplement: Supplementary file 16 — Source data Fig. 7 [file 44318_2024_136_MOESM16_ESM.zip › Figure 7P/Pectoral fin-atg7 sgRNA-14 dpf.tif]

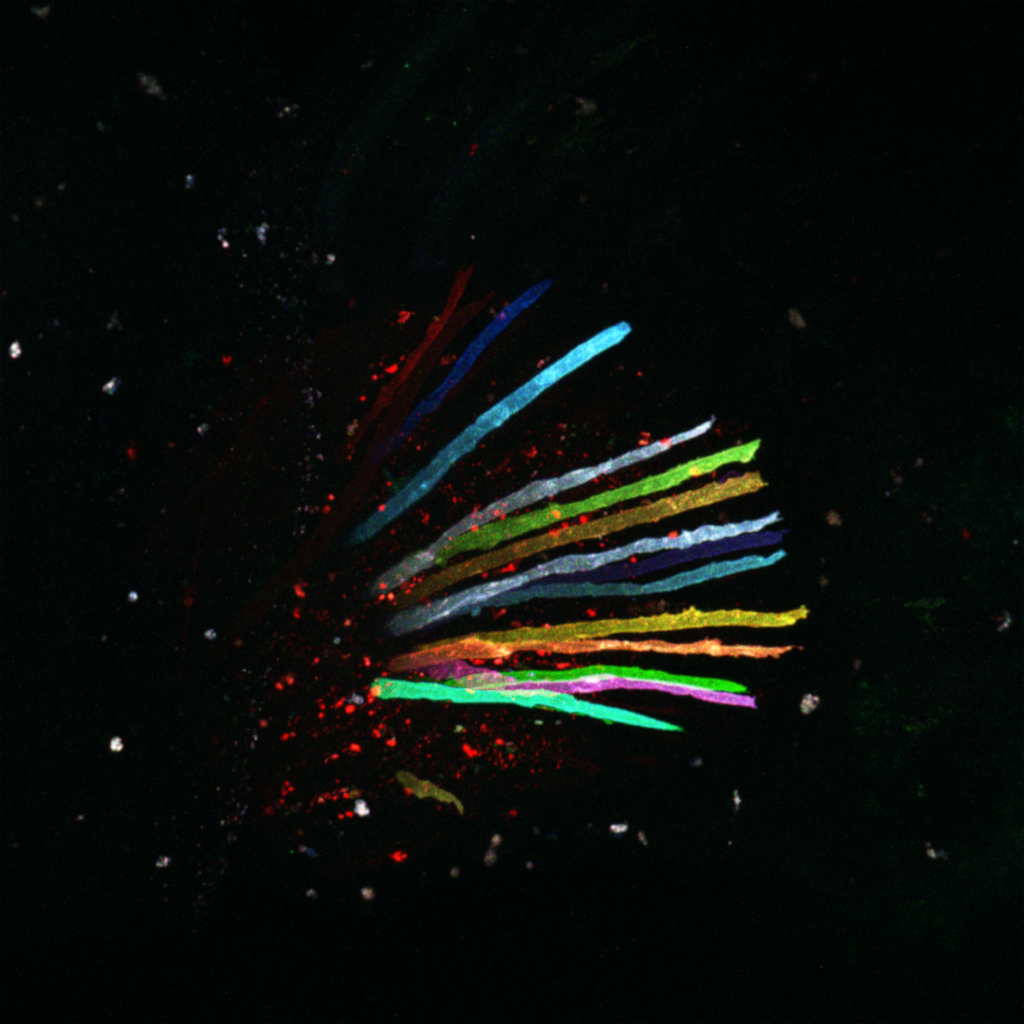

Supplement: Supplementary file 16 — Source data Fig. 7 [file 44318_2024_136_MOESM16_ESM.zip › Figure 7P/Pectoral fin-atg7 sgRNA-28 dpf.tif]

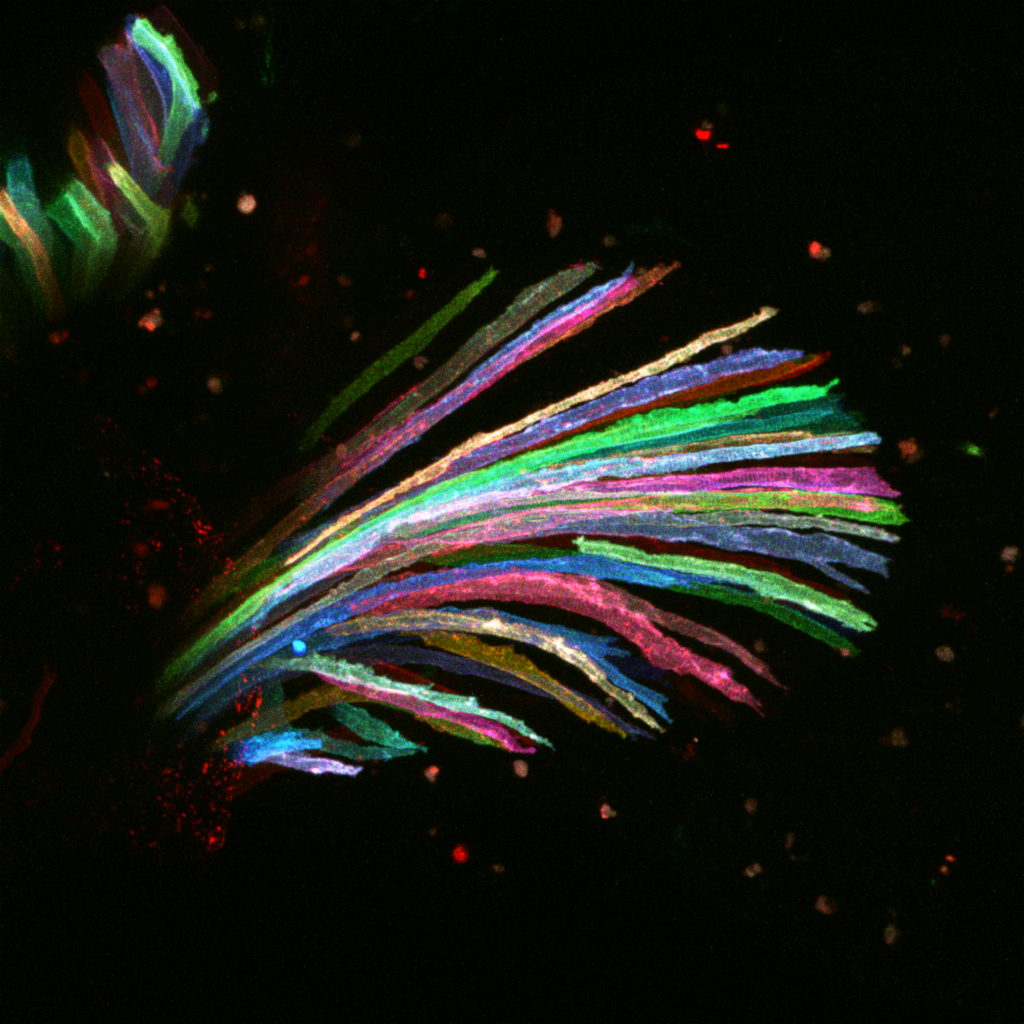

Supplement: Supplementary file 16 — Source data Fig. 7 [file 44318_2024_136_MOESM16_ESM.zip › Figure 7P/Pectoral fin-ctrl sgRNA-14 dpf.tif]

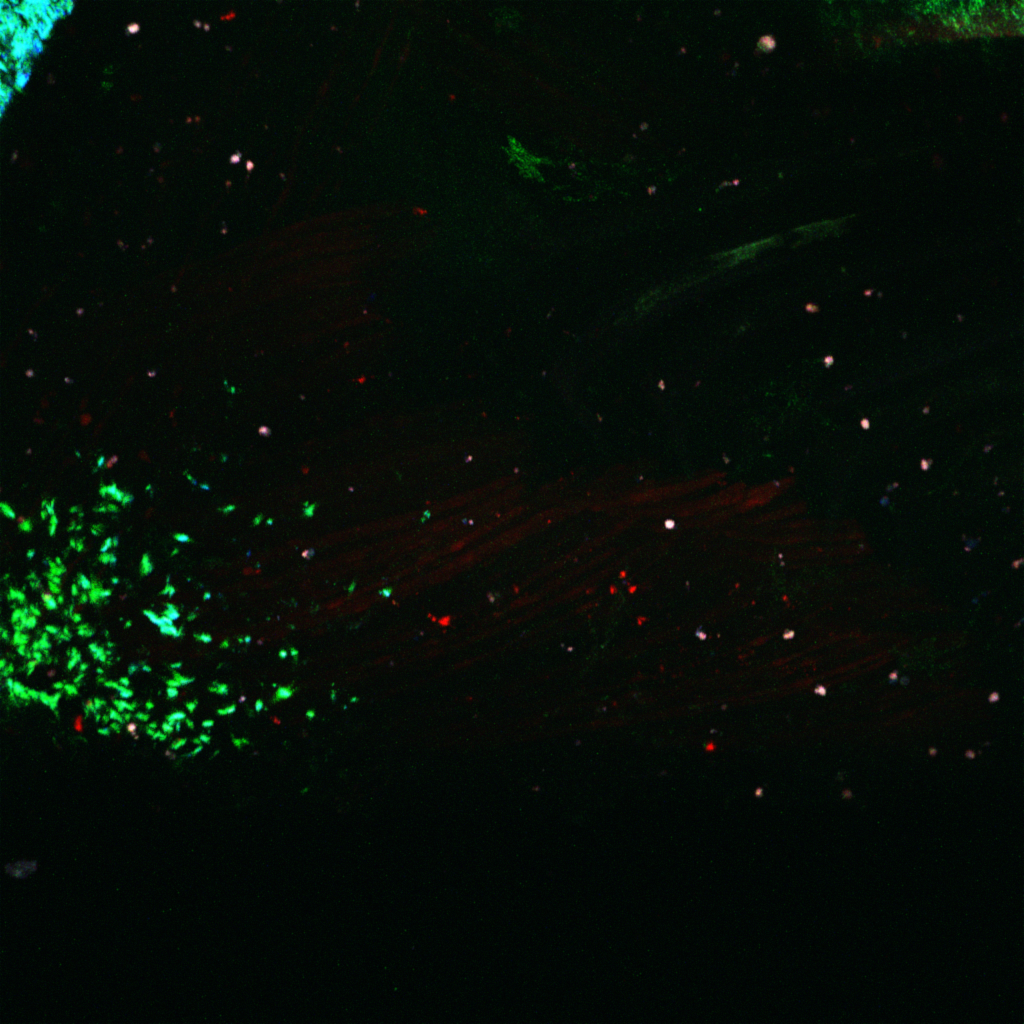

Supplement: Supplementary file 16 — Source data Fig. 7 [file 44318_2024_136_MOESM16_ESM.zip › Figure 7P/Pectoral fin-ctrl sgRNA-28 dpf.tif]
