# Supplementary figures and images for: Whole-body replacement of larval myofibers generates permanent adult myofibers in zebrafish (part 4 of 4)
Source: EMBO J. 2024 Jun 5;43(15):2. doi: 10.1038/s44318-024-00136-y (PMC11294464; doi:10.1038/s44318-024-00136-y)

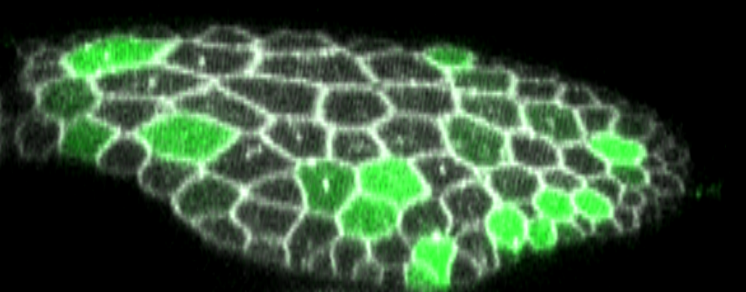

Supplement: Supplementary file 16 — Source data Fig. 7 [file 44318_2024_136_MOESM16_ESM.zip › Figure 7U/EGFP control-11 dpf.tif]

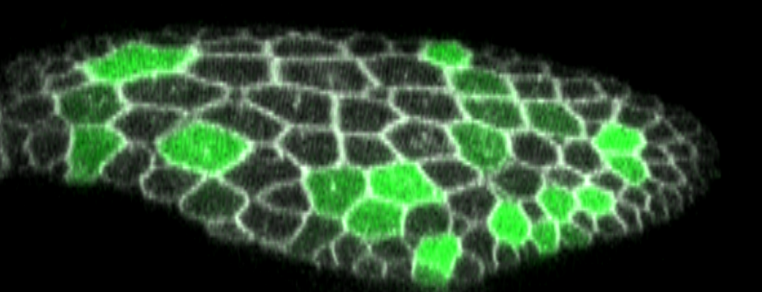

Supplement: Supplementary file 16 — Source data Fig. 7 [file 44318_2024_136_MOESM16_ESM.zip › Figure 7U/EGFP control-12 dpf.tif]

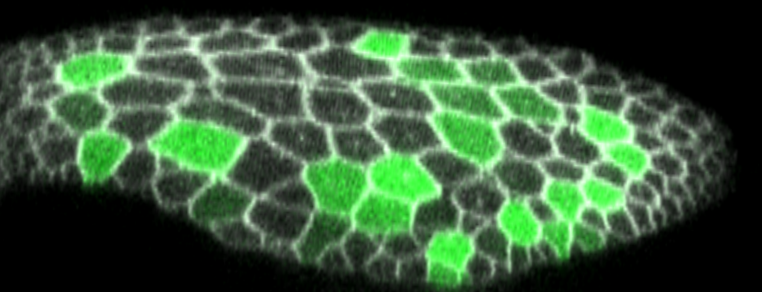

Supplement: Supplementary file 16 — Source data Fig. 7 [file 44318_2024_136_MOESM16_ESM.zip › Figure 7U/EGFP control-13 dpf.tif]

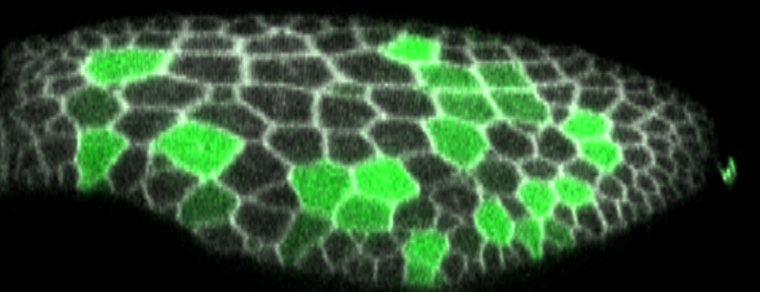

Supplement: Supplementary file 16 — Source data Fig. 7 [file 44318_2024_136_MOESM16_ESM.zip › Figure 7U/EGFP control-14 dpf.tif]

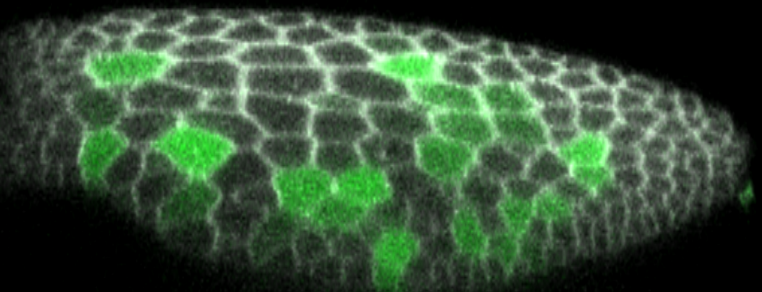

Supplement: Supplementary file 16 — Source data Fig. 7 [file 44318_2024_136_MOESM16_ESM.zip › Figure 7U/EGFP control-17 dpf.tif]

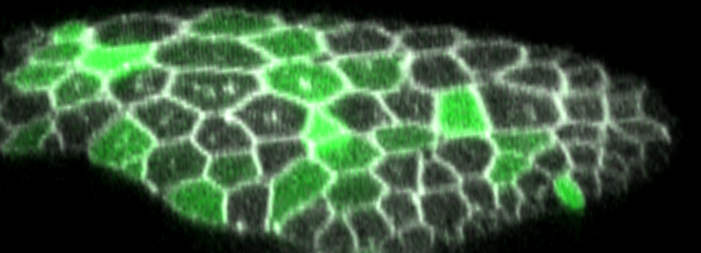

Supplement: Supplementary file 16 — Source data Fig. 7 [file 44318_2024_136_MOESM16_ESM.zip › Figure 7U/EGFP-2A-ATG7-11 dpf.tif]

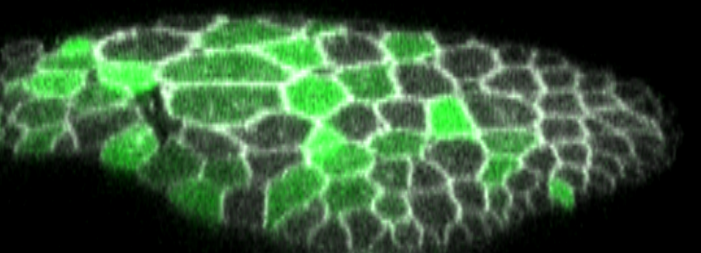

Supplement: Supplementary file 16 — Source data Fig. 7 [file 44318_2024_136_MOESM16_ESM.zip › Figure 7U/EGFP-2A-ATG7-12 dpf.tif]

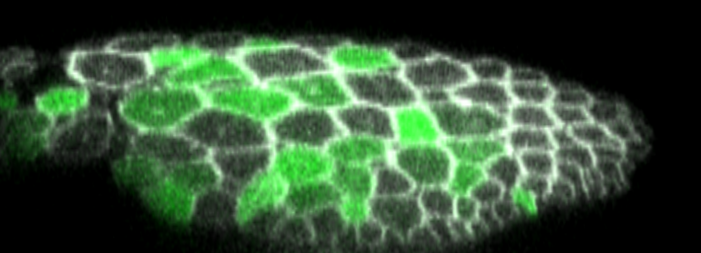

Supplement: Supplementary file 16 — Source data Fig. 7 [file 44318_2024_136_MOESM16_ESM.zip › Figure 7U/EGFP-2A-ATG7-13 dpf.tif]

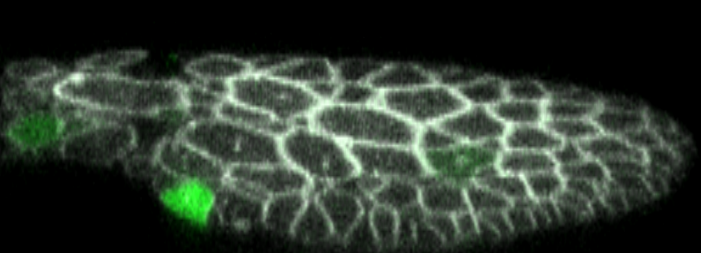

Supplement: Supplementary file 16 — Source data Fig. 7 [file 44318_2024_136_MOESM16_ESM.zip › Figure 7U/EGFP-2A-ATG7-14 dpf.tif]

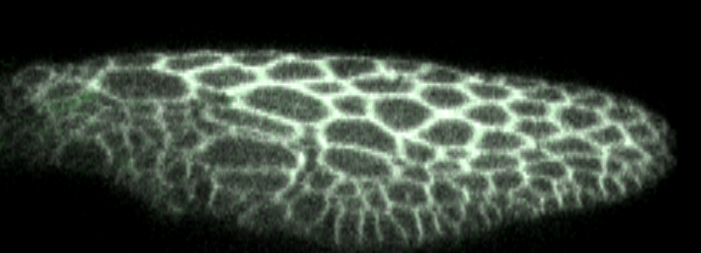

Supplement: Supplementary file 16 — Source data Fig. 7 [file 44318_2024_136_MOESM16_ESM.zip › Figure 7U/EGFP-2A-ATG7-17 dpf.tif]
