# Supplementary material for: Whole-body replacement of larval myofibers generates permanent adult myofibers in zebrafish
Source: EMBO J. 2024 Jun 5;43(15):2. doi: 10.1038/s44318-024-00136-y (PMC11294464; doi:10.1038/s44318-024-00136-y)
Supplement: Supplementary file 17 — Expanded View Figures [file 44318_2024_136_MOESM17_ESM.pdf]

Expanded View Figures

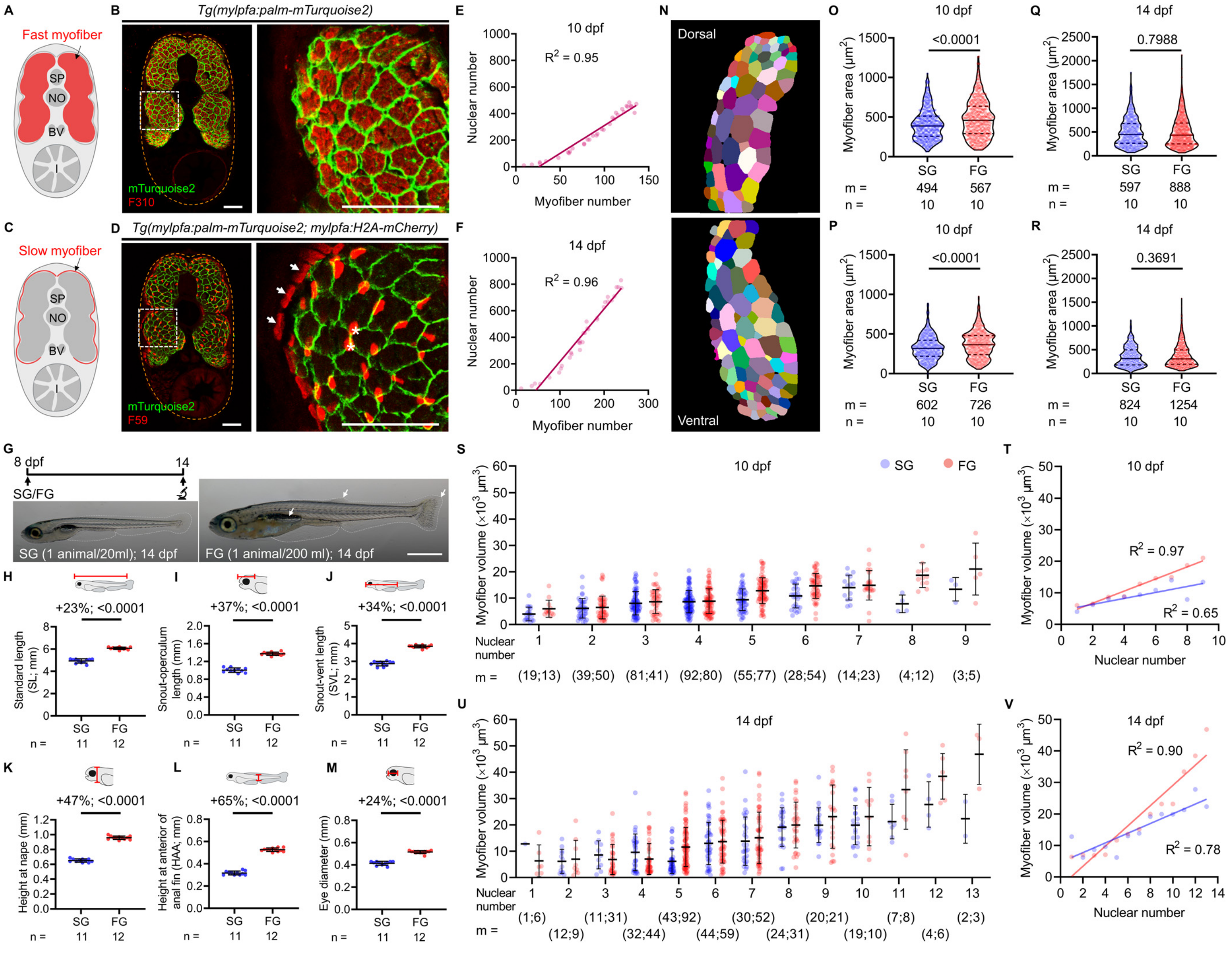

Figure EV1. Myofiber volume scales linearly with myofiber nuclear number regardless of the growth conditions.

(**A**) Schematic highlights the fast myofiber compartment. SP, spinal cord. NO, notochord. BV, blood vessels. I, intestine. (**B**) Histological examination of the labeled myofibers in the *Tg(mylpfa:palm-mTurquoise2)* transgenic line (left). White dashed box outlines the magnified region shown to the right. F310 Ab stains fast myofibers. (**C**) Schematic highlights the slow myofiber compartment. (**D**) Histological examination of the labeled myofibers in the *palmuscle-Dual*: *Tg(mylpfa:palm-mTurquoise2; mylpfa:H2A-mCherry)* double transgenic line (left). White dashed box marks the magnified region shown to the right. Orange dashed lines outline the entire trunk compartment. F59 Ab stains slow myofibers. White arrows point to slow myofibers. White asterisks mark the fast myofiber nuclei. (**E, F**) Linear regression coefficients analyses of myofiber number versus nuclear number across all 32 myotomes in individual animals at 10 dpf (**E**) and 14 dpf (**F**). (**G**) Timeline of the growth manipulation and tracking scheme. Representative images of fish grown under either slow growth (SG; 1 animal in 20 ml) or fast growth (FG; 1 animal in 200 ml) conditions at 14 dpf. White arrows point to the emergence of swim bladder, median fin fold, and caudal fin structures under the FG condition. White dashed lines mark the fin fold region. (**H–M**) Quantification of anatomical traits under SG and FG: Standard length, SL (**H**); Snout-operculum length (**I**); Snout-vent length (**J**); Height at nape (**K**); Height at anterior of anal fin, HAA (**L**) and Eye diameter (**M**). (**N**) A cross-sectional image of a myotome, individual myofibers are depicted in pseudocolor by Cellpose. (**O–R**) Quantitation of the myofiber cross-sectional area in either the dorsal or ventral compartment of Myotome #12 at 10 dpf (**O, P**) and 14 dpf (**Q, R**). (**S**) Quantification of individual myofiber volume at 10 dpf under either slow growth (SG) or fast growth (FG) conditions. (**T**) Linear regression coefficients were calculated for myofiber volume versus nuclear number (10 dpf). (**U**) Quantification of the individual myofiber volume at 14 dpf under either the SG or the FG conditions. (**V**) Linear regression coefficients were calculated for myofiber volume versus nuclear number (14 dpf). Data from biological replicates are shown as mean ± standard deviation (**H–M, S, U**) or violin plots (solid lines, median; dashed lines, quartiles; **O–R**). Significance was examined by examined by two-tailed Student's t-test (**H–L**) or two-tailed Mann–Whitney test (**M, O–R**). *P* values are shown above the horizontal lines for intergroup comparisons. *n* = number of animals (**H–M, O–R**) and m = number of myofibers (**O–S, U**). Four animals (**E, F**) and ten animals (**S, U**) were used in each condition. Scale bars, 50 µm (**B, D**); 1 mm (**G**).

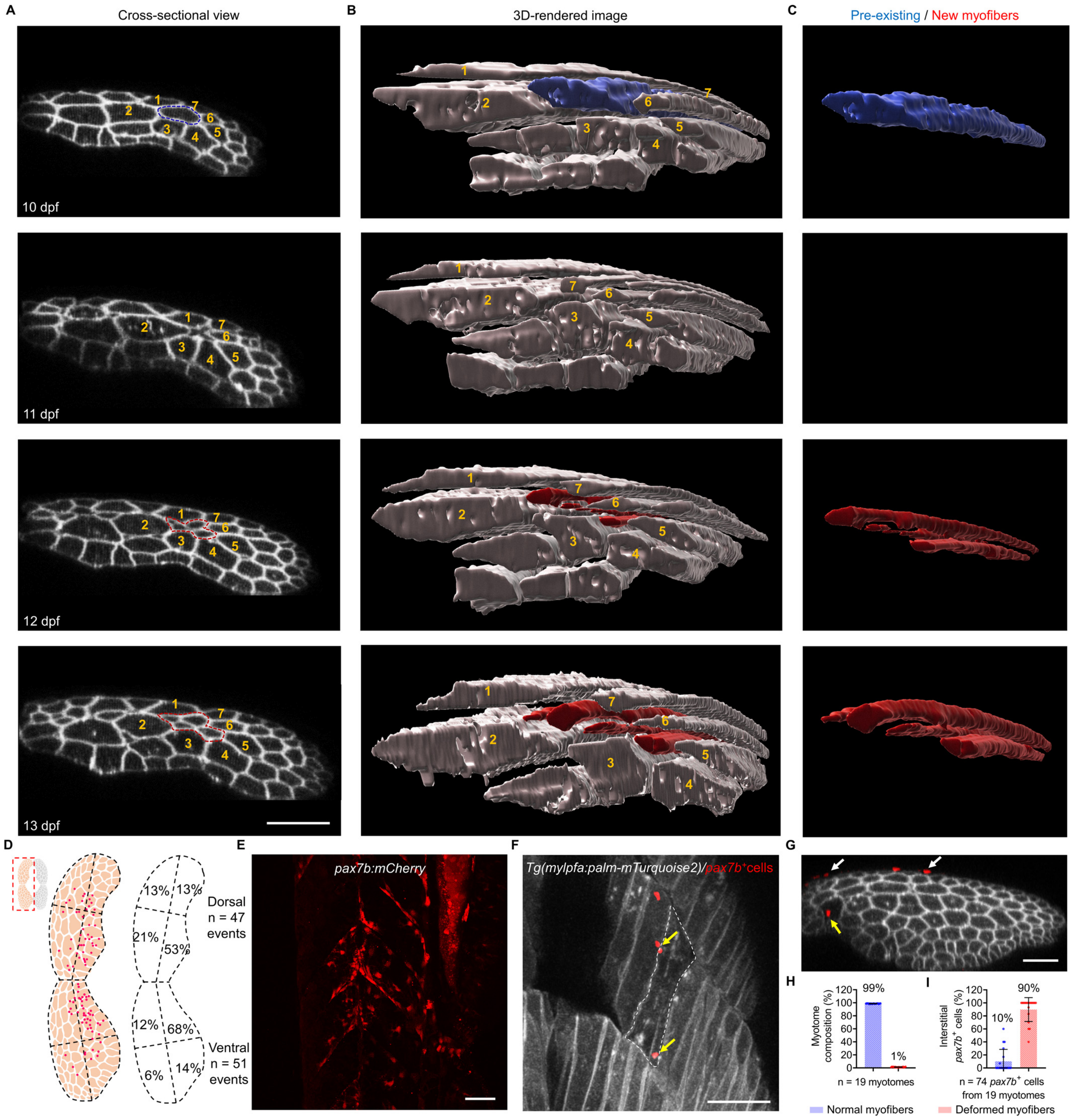

Figure EV2. Individual deformed myofibers are fully eliminated and replaced.

(**A–C**) Time-lapse images of the same myotome over a 3-day period, showing the myofiber elimination and the replacement processes in a cross-sectional view (**A**) or as a 3D rendering (**B**, **C**). Blue dashed lines outline a deformed myofiber. Red dashed lines outline newborn myofibers. Neighboring myofibers are labeled with respective numbers. (**D**) Spatial distribution of deformed myofibers in the dorsal and ventral myotomes. A total of 98 dissolution events were captured and mapped. (**E**) Representative image of *pax7b*-positive muscle stem cells expressing mCherry. (**F**) Representative image showing *pax7b*-positive cells (yellow arrows) in close proximity to a deformed myofiber. (**G**) Cross-sectional view of a myotome showing *pax7b*-positive cells located either at the myotome's periphery (white arrows) or within the interstitial space (yellow arrows). (**H**, **I**) Quantification of myofiber composition within a myotome (**H**) and the interstitial *pax7b*-positive cells closely associated with either normal or deformed myofibers (**I**). Data from biological replicates are shown as mean ± standard deviation (**H**, **I**). n = number of myotome (**H**) or cells (**I**). Scale bar, 50 µm (**A**, **E–G**).

 © The Author(s)

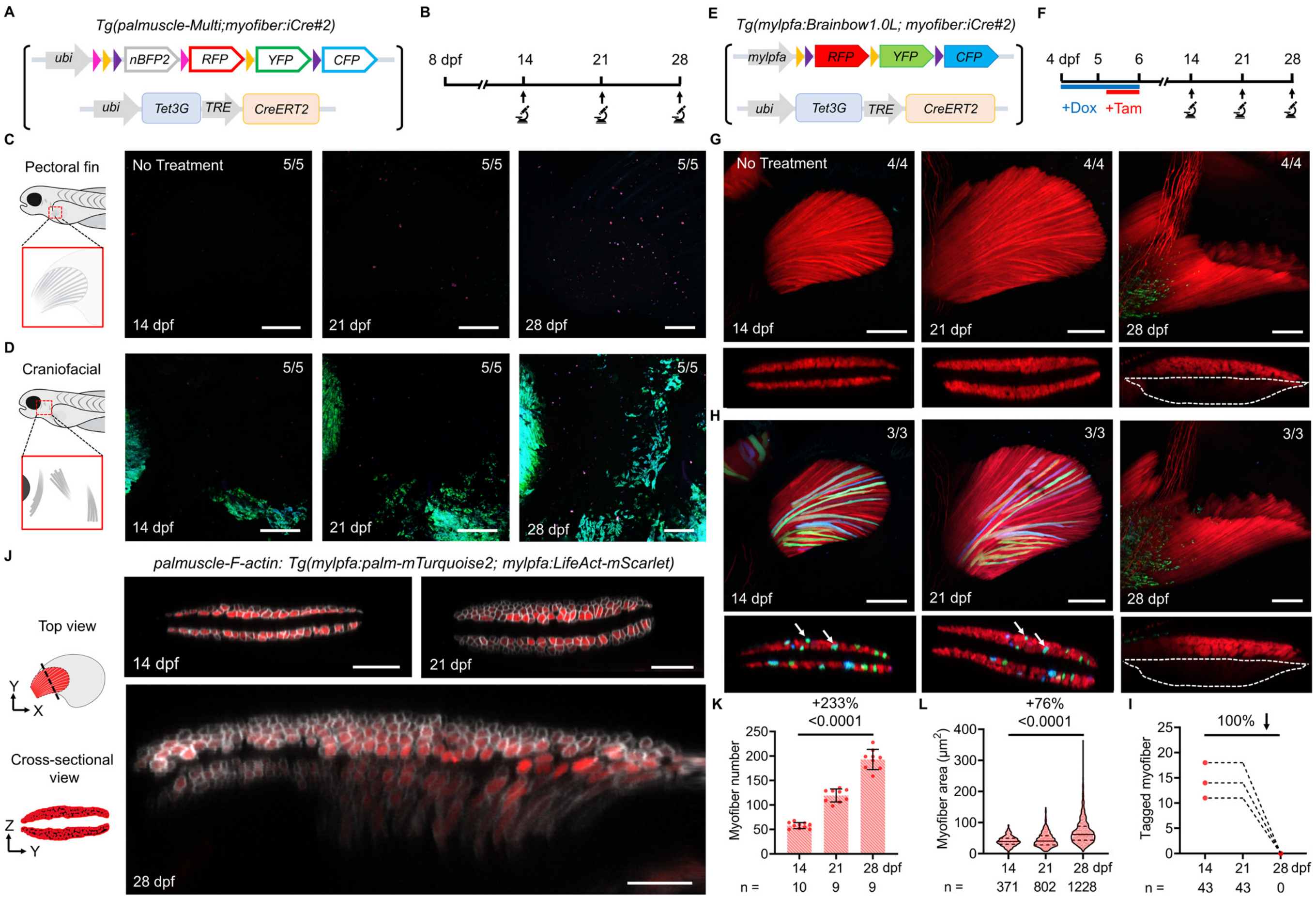

Figure EV3. Pre-existing pectoral fin myofibers are eliminated and replaced by a de novo source.

(**A**) The *palmuscle-Multi* and the *myofiber:iCre#2* transgenic constructs. (**B**) Timeline of the tracking scheme. (**C, D**) Long-term tracking of the *Tg(palmuscle-Multi; myofiber:iCre#2)* double transgenic line in different anatomical regions, including the pectoral fin region (**C**) and the craniofacial region (**D**). No leaky Cre activity was observed. (**E**) The *mylpfa:Brainbow1.0 L* and *myofiber:iCre#2* transgenic constructs. (**F**) Timeline of the treatment and tracking scheme. (**G**) Long-term tracking of the *Tg(mylpfa:Brainbow1.0 L; myofiber:iCre#2)* double transgenic line showed no leaky Cre activity. (**H**) Long-term time-lapse imaging of the same myofibers at 14, 21, and 28 dpf. White arrows highlight "disappearing myofibers". White dashed lines (bottom right, **G** and **H**) outline the bottom pectoral fin myofiber compartment, which becomes less visible at 28 dpf due to tissue thickening. (**I**) Quantification of tagged myofiber numbers. (**J**) Cross-sectional view of the pectoral fin myofibers showing both top and bottom layers at 14, 21 and 28 dpf. (**K, L**) Quantification of myofiber numbers (**K**) and myofiber areas (**L**). The entire top layer of fin myofibers were included in the quantification. Data from biological replicates are shown as mean ± standard deviation (**K**) or violin plots (solid lines, median; dashed lines, quartiles; **L**). Significance was examined by two-tailed Student's t-test (**K**) or two-tailed Mann-Whitney test (**L**). Percent differences and *P* values are shown above the horizontal lines for intergroup comparisons. *n* = number of animals (**C, D, G, H, K**) or myofibers (**I, L**). Scale bars, 50 μm (**J**) and 100 μm (**C, D, G, H**). dpf, days post-fertilization.

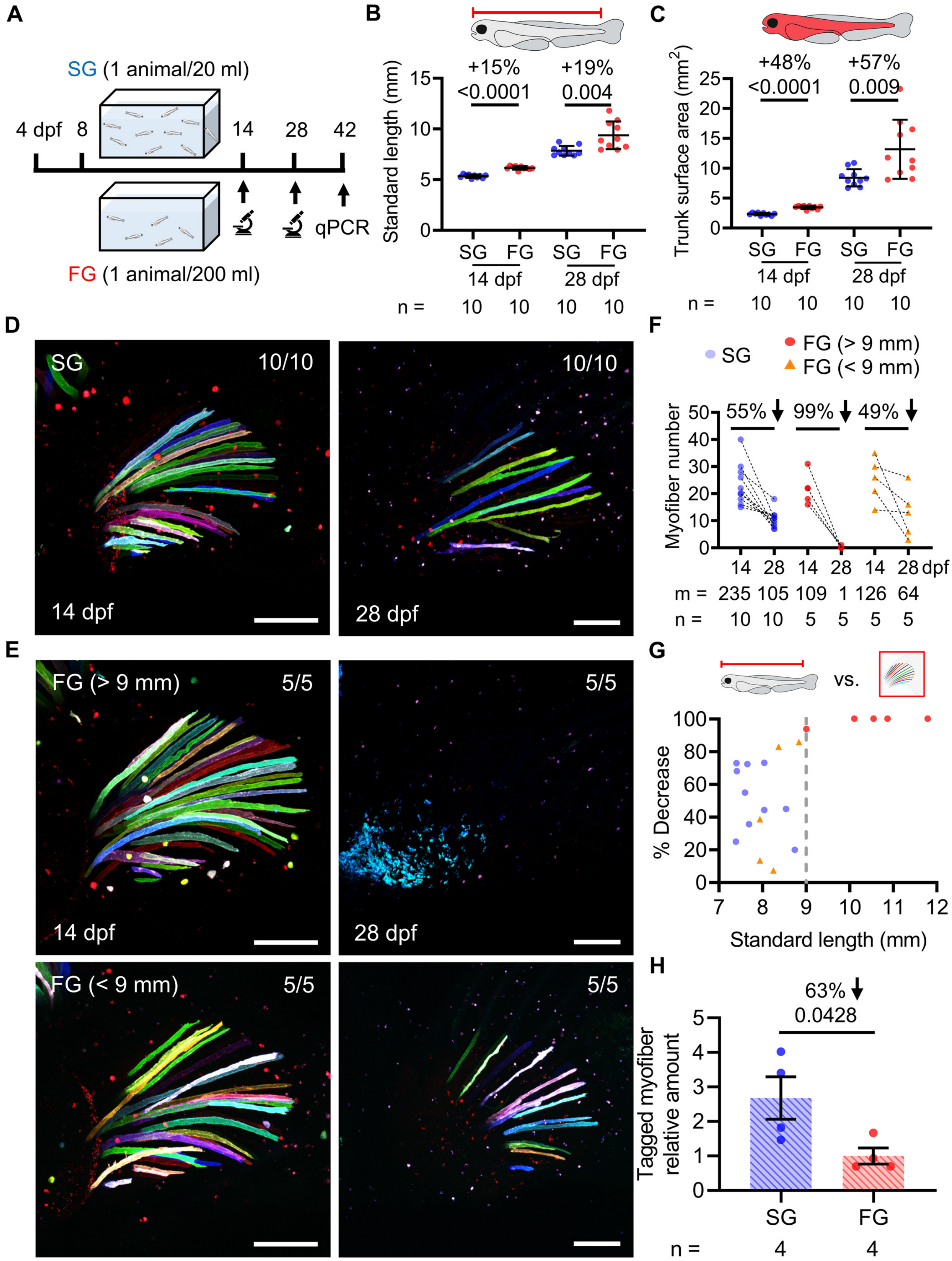
A
SG (1 animal/20 ml)
4 dpf 8 14 28 42
qPCR
FG (1 animal/200 ml)
B
+15% +19%
<0.0001 0.004
Standard length (mm)
SG FG SG FG
14 dpf 28 dpf
n = 10 10 10 10
C
+48% +57%
<0.0001 0.009
Trunk surface area (mm2)
SG FG SG FG
14 dpf 28 dpf
n = 10 10 10 10
D
SG 10/10
14 dpf
10/10
28 dpf
F
SG
FG (> 9 mm)
FG (< 9 mm)
55% 99% 49%
Myofiber number
14 28 14 28 14 28 dpf
m = 235 105 109 1 126 64
n = 10 10 5 5 5 5
E
FG (> 9 mm) 5/5
14 dpf
5/5
28 dpf
FG (< 9 mm) 5/5
5/5
G
vs.
% Decrease
Standard length (mm)
H
63%
0.0428
Tagged myofiber relative amount
SG FG
n = 4 4

 © The Author(s)

Figure EV4. Rapid growth condition promotes myofiber elimination.

(**A**) Timeline of larval growth manipulation and tracking scheme. SG, slow growth. FG, fast growth. (**B**, **C**) Larval growth under the SG and FG conditions as determined by standard length (**B**), and trunk surface area (**C**). (**D**, **E**) Long-term tracking of the *Tg(palmuscle-Multi; myofiber:iCre#2)* double transgenic line under SG (**D**) and FG (**E**) conditions. (**F**, **G**) Quantification of tagged myofiber numbers (**F**) and decreases in percentage (**G**). Gray dashed line highlights the standard length of 9 mm. (**H**) RT-qPCR analysis of whole-animal myofiber loss at 42 dpf under either SG or FG conditions. Data from biological replicates are shown as mean ± standard deviation (**B**, **C**) and mean ± standard error (**H**). Significance was examined by two-tailed Student's t-test. Percent differences and *P* values are shown above the horizontal lines for intergroup comparisons. n = number of animals (**B**–**F**) or biological replicates (**H**). m = number of myofibers (**F**). Scale bar, 100 μm (**D**, **E**). dpf, days post-fertilization.

© The Author(s)

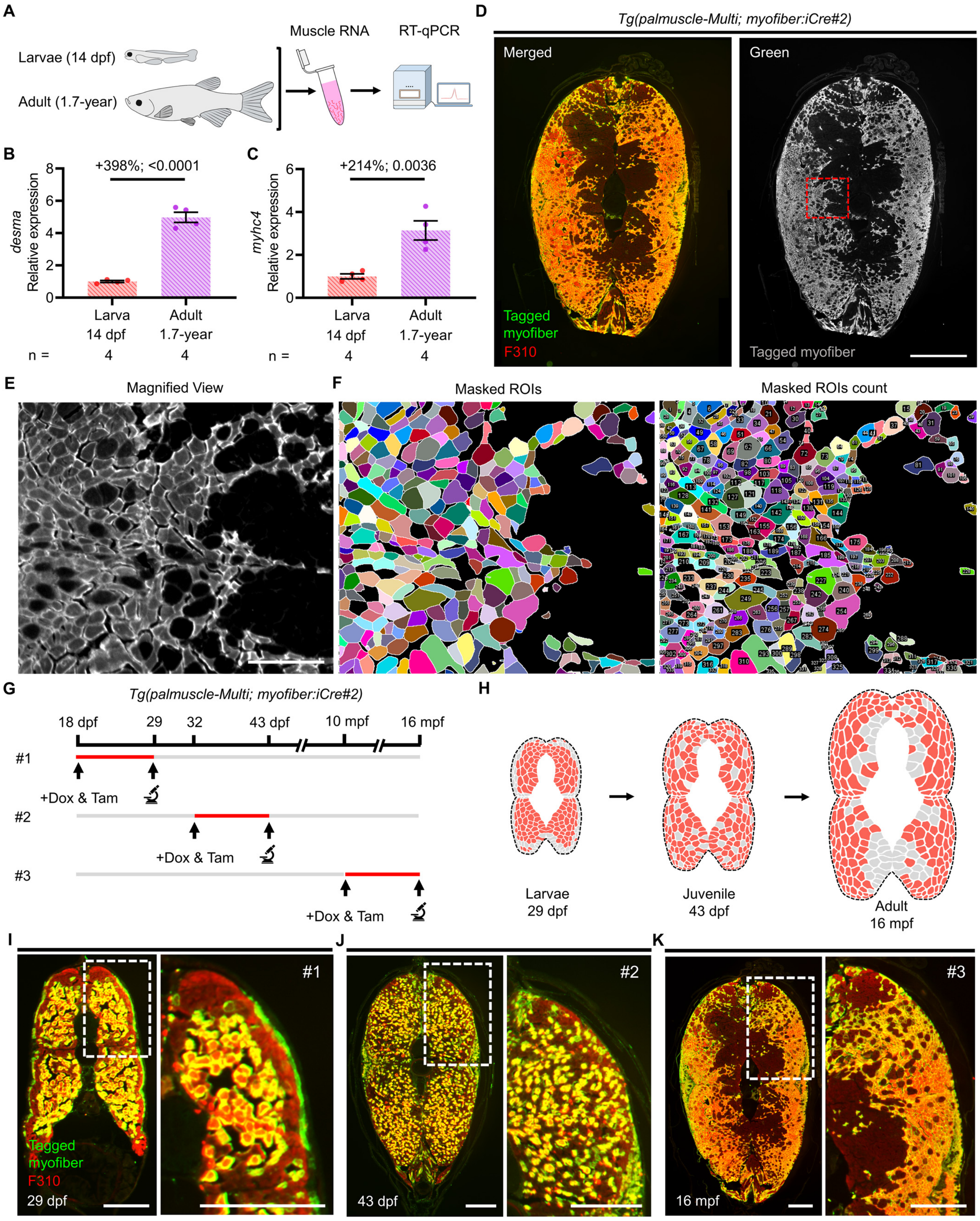
A
Larvae (14 dpf)
Adult (1.7-year)
Muscle RNA
RT-qPCR
B
+398%; <0.0001
desma
Relative expression
Larva
14 dpf
Adult
1.7-year
n = 4 4
C
+214%; 0.0036
myhc4
Relative expression
Larva
14 dpf
Adult
1.7-year
n = 4 4
D
Tg(palmuscle-Multi; myofiber:iCre#2)
Merged
Green
Tagged
myofiber
F310
Tagged myofiber
E
Magnified View
F
Masked ROIs
Masked ROIs count
G
Tg(palmuscle-Multi; myofiber:iCre#2)
18 dpf
29
32
43 dpf
10 mpf
16 mpf
#1
+Dox & Tam
#2
+Dox & Tam
#3
+Dox & Tam
H
Larvae
29 dpf
Juvenile
43 dpf
Adult
16 mpf
I
#1
Tagged
myofiber
F310
29 dpf
J
#2
43 dpf
K
#3
16 mpf

 © The Author(s)

Figure EV5. Larval myofibers are functionally distinct from adult myofibers.

(**A**) Illustration of zebrafish at larval and adult stages, reflecting their relative size differences. (**B**, **C**) RT-qPCR analyses of expression of the intermediate filament *desma* (**B**), and sarcomere myosin *myhc4* (**C**) in whole animals (14 dpf) or dissected muscle tissues (1.7 years of age). (**D**) Cross-sectional image of the middle-trunk region of the animal at 16 mpf with tagged myofibers. F310 Ab stains fast myofibers. (**E**) Magnified view of the trunk region indicated in (**D**) by red dashed box. (**F**) Individual tagged myofibers from cross-sectional images shown in pseudocolor (left) and with respective ROI numbers (right). (**G**) Timeline of the treatment and tracking scheme. (**H**) Schematic drawing of the progressive shift in hyperplastic growth zone across different developmental stages. (**I**–**K**) Histological examinations of the tagged myofibers in the middle-trunk region from larvae to adult stage, showing growth zone shift from the periphery at 29 dpf (**I**), to the interstitial space at 43 dpf (**J**), and ultimately to the deep region of the myotome at 16 mpf (**K**). White dashed boxes outline the magnified regions shown to the right of each panel. F310 Ab stains fast myofibers. Data from biological replicates are shown as mean ± standard error (**B**, **C**). Significance was examined by two-tailed Student's t-test. Percent differences and *P* values are shown above the horizontal lines for intergroup comparisons. n = the number of biological replicates. Scale bars, 1 mm (**D**); 200 μm (**E**, **I**); 300 μm (**J**); 500 μm (**K**). dpf, days post-fertilization; mpf, months post-fertilization.

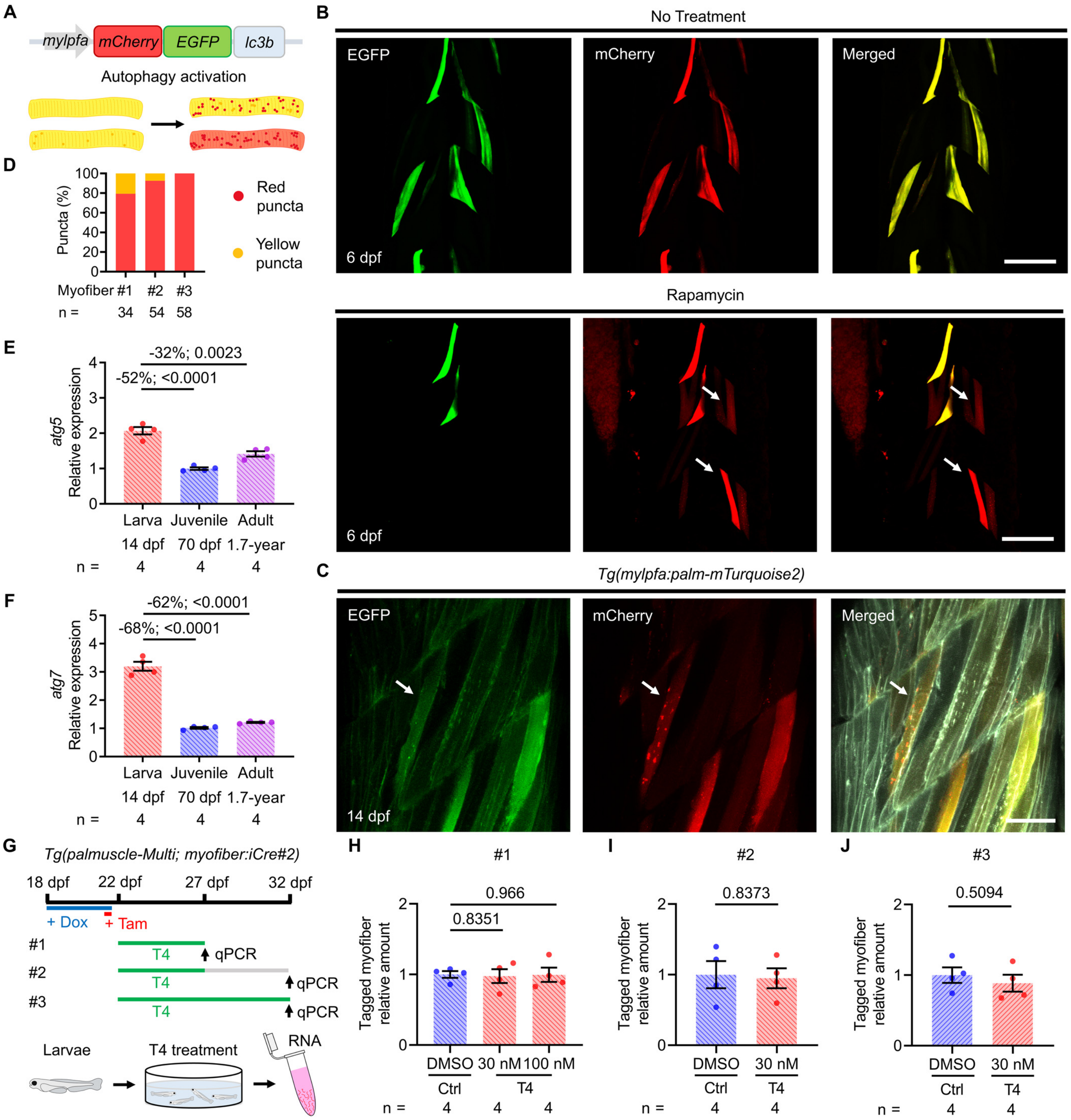

Figure EV6. Live monitoring of autophagic activation in deformed myofibers.

(**A**) The transgenic construct for live monitoring of autophagic activation in myofibers. (**B**) Treatment with rapamycin activates autophagic response in myofibers. White arrows point to activated, mCherry-positive myofibers. Of note, without the treatment of rapamycin, all 6 dpf myofibers were both EGFP- and mCherry-positive. (**C**) Deformed myofibers contain abundant red puncta. White arrow points to a deformed myofiber. (**D**) Percentages of red and yellow puncta in three individual deformed myofibers. (**E**, **F**) RT-qPCR was performed to analyze expression of the autophagic genes *atg5* (**E**) and *atg7* (**F**) in whole animals (14 dpf) or dissociated myofibers (70 dpf and 1.7 years of age). (**G**) Timeline and three different thyroxine hormone (T4) treatment schemes. (**H**–**J**) RT-qPCR analysis of the whole-animal myofiber loss for each of the schemes. Data from biological replicates are shown as mean ± standard error (**E**, **F**, **H**, **I**, **J**). Significance was examined by two-tailed Student's t-test. Percent differences and *P* values are shown above the horizontal lines for intergroup comparisons. n = number of puncta (**D**) or biological replicates (**E**, **F**, **H**, **I**, **J**). Scale bars, 100 μm (**B**); 50 μm (**C**). dpf, days post-fertilization.

 © The Author(s)
